# Automated Flow Synthesis of Tumor Neoantigen Peptides for Personalized Immunotherapy

**DOI:** 10.1038/s41598-019-56943-5

**Published:** 2020-01-20

**Authors:** Nicholas L. Truex, Rebecca L. Holden, Bin-You Wang, Pu-Guang Chen, Stephanie Hanna, Zhuting Hu, Keerthi Shetty, Oriol Olive, Donna Neuberg, Nir Hacohen, Derin B. Keskin, Patrick A. Ott, Catherine J. Wu, Bradley L. Pentelute

**Affiliations:** 1 Department of Chemistry, Massachusetts Institute of Technology, 77 Massachusetts Avenue, Cambridge, MA 02139 USA; 2 Department of Medical Oncology, Dana-Farber Cancer Institute, Boston, MA 02215 USA; 3 Translational Immunogenomics Laboratory, Dana-Farber Cancer Institute, Boston, MA 02215 USA; 4 Department of Biostatistics and Computational Biology, Dana-Farber Cancer Institute, Boston, MA 02215 USA; 5 Harvard Medical School, Boston, MA 02215 USA; 6 Broad Institute of MIT and Harvard, Cambridge, MA 02142 USA; 7 Center for Cancer Research, Massachusetts General Hospital, Boston, MA 02114 USA; 8 Department of Medicine, Brigham and Women’s Hospital, Boston, MA 02215 USA; 9 Koch Institute for Integrative Cancer Research, Massachusetts Institute of Technology, Cambridge, MA 02139 USA

**Keywords:** Peptides, Cancer immunotherapy, Chemical engineering

## Abstract

High-throughput genome sequencing and computation have enabled rapid identification of targets for personalized medicine, including cancer vaccines. Synthetic peptides are an established mode of cancer vaccine delivery, but generating the peptides for each patient in a rapid and affordable fashion remains difficult. High-throughput peptide synthesis technology is therefore urgently needed for patient-specific cancer vaccines to succeed in the clinic. Previously, we developed automated flow peptide synthesis technology that greatly accelerates the production of synthetic peptides. Herein, we show that this technology permits the synthesis of high-quality peptides for personalized medicine. Automated flow synthesis produces 30-mer peptides in less than 35 minutes and 15- to 16-mer peptides in less than 20 minutes. The purity of these peptides is comparable with or higher than the purity of peptides produced by other methods. This work illustrates how automated flow synthesis technology can enable customized peptide therapies by accelerating synthesis and increasing purity. We envision that implementing this technology in clinical settings will greatly increase capacity to generate clinical-grade peptides on demand, which is a key step in reaching the full potential of personalized vaccines for the treatment of cancer and other diseases.

## Introduction

Personalized medicine guided by genome-sequencing technology represents a new paradigm for disease treatment and prevention^1^. These therapies offer the promise of precision, but also present a formidable challenge—administering custom-made treatments on demand^2–4^. Providing these treatments in a rapid and affordable fashion remains a barrier that currently limits their potential^5,6^.

A salient example for personalized medicine is that of personalized neoantigen vaccines for cancer, in which on-demand manufacturing for individual patients is a challenge^7–13^. These vaccines are based on the array of somatic mutations that can form in a tumor, which encode novel, tumor-specific antigens, called ‘neoantigens’. Immune targeting of multiple neoantigens in concert is expected to promote selective immune activation against cancer cells and prevent immunologic escape. Indeed, five clinical trials testing personalized neoantigen vaccines in patients with melanoma and glioblastoma multiforme have shown that these treatments can generate immune responses in humans^12–16^. Synthetic long peptides are a mainstay of the treatments, which have also been administered in conjunction with adjuvants^17,18^. Four of the five clinical trials with personalized cancer vaccines have used synthetic peptides in the immunizing formulation^12–15^, while the fifth study used synthetic RNA^16^. All five trials also used several dozen shorter peptides per patient to perform *ex vivo* immune monitoring studies.

**Figure 1:**
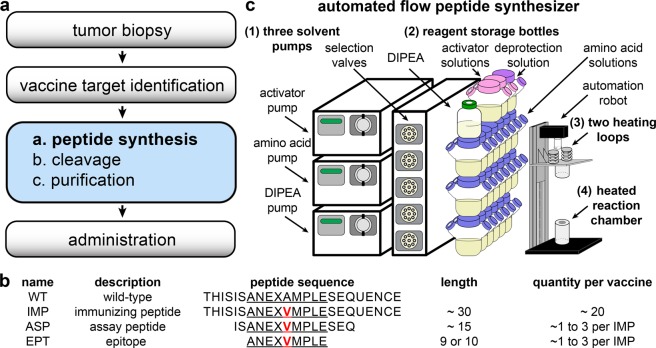
Peptide design and production for a personalized neoantigen vaccine. (**a**) Workflow for the design and production of neoantigen vaccines. (**b**) Example peptide sequences for a wildtype (WT), immunizing (IMP), immune monitoring assay (ASP), and epitope (EPT) peptides. (**c**) Schematic illustration of an automated flow peptide synthesizer (without connective capillary tubing and UV-vis module).

Personalized neoantigen-targeting vaccine studies use hundreds of peptides that range in length from 8 to 30 amino acids. In designing the peptides, several studies have adopted the following workflow: After a tumor biopsy, mutated epitopes were identified using whole-exome sequencing (WES) of tumor and normal cells in parallel; epitope peptides (EPTs) of 8 to 10 amino acids that can bind to personal human leukocyte antigen (HLA) alleles were then identified using class I binding predictive algorithms^19^; up to 20 minimal class I epitope peptides were chosen as neoantigen vaccine targets, and were included within synthetic long immunizing peptides (IMPs) of 15 to 30 amino acids; The peptides were then synthesized by a commercial peptide vendor, cleaved and purified under good manufacturing practice (GMP) conditions, and then administered to the patient as immunizing peptides (see Fig. 1a). The long peptides, IMPs, were synthesized for vaccine administration, because similar peptides have been shown to effectively stimulate antigen-specific CD4^+^ and CD8^+^ T cells^17,18^. In addition, shorter overlapping assay peptides (ASPs) were synthesized to evaluate immune responses. Figure 1b illustrates the EPTs, ASPs, and IMPs, and also summarizes the quantity of each set designed per patient. In the earlier studies, the average lead time to generate 20 IMPs ranged from 18 to 20 weeks, which was largely devoted to the time-consuming and expensive synthesis of clinical-grade peptides^12,13^. Minimizing this time and cost is vital to allow treatment of other cancer types, including metastatic cancers.

In 2017, we introduced an automated flow peptide synthesizer that accelerates the rate of peptide synthesis^20^. This synthesizer builds on our previous advances with flow chemistry of fluorenylmethyloxycarbonyl (Fmoc)-based solid-phase peptide synthesis^20–24^. The flow conditions can achieve quantitative amide-bond coupling in seconds, while standard microwave or batch peptide syntheses require minutes or even hours. Our automated flow synthesizer (see Fig. 1c) is composed of five main modules: (1) three solvent pumps, which continuously draw solutions of amino acids, activator base, deprotection agents, *N,N*-diisopropylethylamine (DIPEA), or *N,N*-dimethylformamide (DMF); (2) reagent storage bottles; (3) two heating loops, which preheat the solutions prior to flowing into the reaction vessel; (4) one heated reaction chamber, which stores the solid-support resin for synthesis; and (5) one UV-vis detector (not shown), which allows relative quantification of Fmoc removal during the deprotection step.

Here we describe how automated flow peptide synthesis can facilitate the production of neoantigen peptides for personalized cancer vaccines. We show that automated flow peptide synthesis can produce high-quality 30-mer IMPs in less than 35 minutes, while other peptide synthesis methods take several hours or days with comparable reaction equivalents. We also show that these peptides are equal or higher in quality when compared to peptides produced by microwave or batch synthesis, and that these peptides can be purified. Further, we demonstrate that automated flow synthesis technology enables high-throughput production of a set of 15- to 16-mer ASPs for immune-assessment assays. Our results illustrate how automated flow synthesis increases the rate and quality of peptide production. We envision that manufacturing neoantigen vaccines using this technology will greatly reduce turnaround time and increase availability, thereby enabling true on-demand administration of these personalized treatments.

## Results and Discussion

### Limitations of conventional peptide synthesis for personalized neoantigens

Generating the peptides needed for personalized neoantigen vaccines has been difficult using conventional peptide synthesis. Across two clinical studies, personalized neoantigen vaccines were designed, produced, and administered to 22 patients with either high-risk melanoma (NCT01970358) or newly diagnosed glioblastoma (NCT02287428)^12,13^. The peptide lengths varied from 13 to 34 amino acids, and averaged 23 amino acids. The peptides were synthesized by a commercial peptide vendor using a conventional batch synthesis method. The median turnaround time from design to synthesis ranged from 18 to 20 weeks. Although 598 immunizing peptides were designed and ordered for these studies, only 400 peptides (67%) could be synthesized and purified (≥95% purity).

As a representative test case to evaluate the limits of neoantigen peptide production by our automated flow synthesis technology, we selected a set of 29 IMPs that were particularly difficult and time consuming to synthesize, called IMPs **1**–**29** (see Table 1). These peptides originated from 19 different genes and had previously been designed for inclusion in a vaccine^12^.

**Table 1:** Sequences of IMPs **1**–**29** from a previous clinical trial.

	amino acid sequence	gene origin	IMP length	vendor purity (%)^*a,b*^	purified purity (%)^*a,c*^
IMP **1**	LTPLTLIQRMNLLMKISIHKLQKSEF	PTEN	26	94	95
IMP **2**	MNLLMKISIHKLQKSEFFFIKRDKT	PTEN	25	89	95
IMP **3**	DNEPDHYILTPLTLIQRMNLLMKISI	PTEN	26		95
IMP **4**	IQRMNLLMKISIHKLQKSEFFFIKRDKTP	PTEN	29		84
IMP **5**	RSSFIQHNMTHTRENPFYAKNVGKLFTTA	ZNF599	29	94	96
IMP **6**	THTRENPFYAKNVGKLFTTAPHLLNI	ZNF599	26	96	98
IMP **7**	KIKELLPDWGGQHHGLREVLAAALFAS	CPT1C	27	99	96
IMP **8**	DWGGQHHGLREVLAAALFASCLWGA	CPT1C	25		69
IMP **9**	SFKLENLEFPDMPLEEWQEIDEKINEMK	AXDND1	28	95	95
IMP **10**	FTLQIRGRERFEMYRELNEALELKD	TP53	25		56
IMP **11**	TLQIRGRERFEMYRELNEALELK	TP53	23		92
IMP **12**	RAELQASDHRPVMAIVEVEVQEVDVG	SYNJ2	26		47
IMP **13**	HRPVMAIVEVEVQEVDVGARERVF	SYNJ2	24		96
IMP **14**	YSLDSSGNQNLYAMYQLSHFQSISVL	PLEKHM3	26		53
IMP **15**	SSGNQNLYAMYQLSHFQSISVLG	PLEKHM3	23	92	83
IMP **16**	TMLVSSLRDHFPDLPLHIHTHDTS	PC	24	93	99
IMP **17**	HIRPLEKEKVIPLVTSFIEAL	UTP20	21	98	97
IMP **18**	KEKVIPLVTSFIEALFMTVDKGSFGK	UTP20	26		97
IMP **19**	DLNPLIKLSGAYLVDDYDPDTSL	IGF2R	23	96	97
IMP **20**	KLSGAYLVDDYDPDTSLFINVCR	IGF2R	23		98
IMP **21**	GDFSREWAEAQHMMRELRNRNFGKHL	LAMA3	26	90	95
IMP **22**	DPRWIRAWWGGFLLCGALLF	SLCO3A1	20		60
IMP **23**	SNLDITPDDPRWIRAWWGGFLLCGA	SLCO3A1	25		45
IMP **24**	MEKQDKWTRKNIKNTRLIHFGDIQA	PLBD1	25	99	75
IMP **25**	AHVIEDQHKFPNYFGKEIIGGMLDI	CWF19L2	25	93	87
IMP **26**	YLTTVELYRCLEARQQEKHFEVLIS	KIF18B	25	97	96
IMP **27**	YLTTVELYRCLEARQQEK	KIF18B	18	99	95
IMP **28**	RRSTECSIHLEVIVDRPLQVFHVD	PCDHAC2	24	98	88
IMP **29**	RLPGSSDCAASASKVVGITDDVFLPK	FAM193A	26	97	95

^*a*^Determined by analytical RP-HPLC by integrating the peptide and impurity peaks at 214 nm.

^*b*^Synthesized using batch peptide synthesis by the commercial vendor.

^*c*^Synthesized using automated flow peptide synthesis.

**Figure 2:**
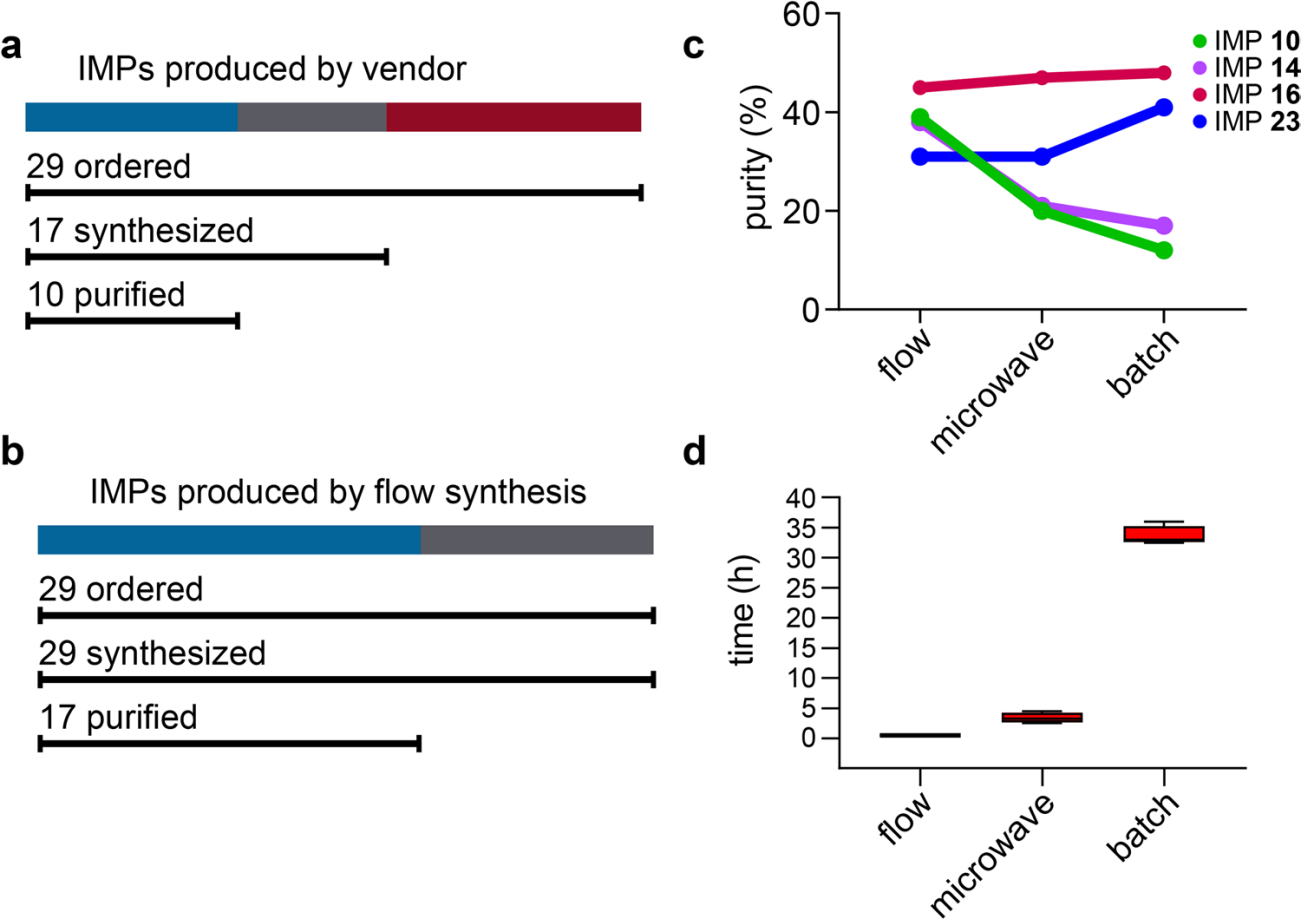
Comparison of peptide synthesis methods. (**a**) Summary of IMPs produced by a commercial peptide vendor. (**b**) Summary of IMPs produced by flow synthesis. (**c**) RP-HPLC purity of unpurified (crude) IMP **10**, IMP **14**, IMP **16**, and IMP **23** produced by flow, microwave, and batch synthesis. (**d**) Synthesis times of the four IMPs by flow, microwave, and batch methods. The upper, middle and lower hinges of the box plot indicate 75th, 50th and 25th quartiles, the whiskers extend to 1.5 × the interquartile range below and above the lower and upper hinge, respectively. The Kruskal-Wallis test was used for comparing the synthesis times, which indicated the synthesis times are significantly different for each method (*P* = 0.0002).

When the commercial vendor attempted to produce IMPs **1**–**29**, their efforts yielded successful syntheses for only 17 of the 29 peptides. Of these 17, only 10 passed purity analysis requirements after purification (>95% HPLC purity). The other 7 IMPs remained lower in purity (89–94% HPLC purity), even after two or three rounds of purification. Challenges encountered throughout the synthesis and purification led to substantial manufacturing delays, and ultimately resulted in only 10 of 29 IMPs returned, or a 66% failure rate (see Fig. 2a).

The following describes our synthesis and purification of IMPs **1**–**29** using automated flow peptide synthesis (see Fig. 2b). We envisioned that producing this set of 30-mer peptides would test the limits of this technology and establish whether flow synthesis can facilitate on-demand production of the immunizing peptides for personalized cancer vaccines.

### Comparison of flow, microwave, and traditional batch synthesis

First, we compared automated flow peptide synthesis with other methods. We synthesized four IMPs by flow, microwave, and batch peptide synthesis, using homologous coupling reagents and conditions for each method (see Materials and Methods, Supplementary Tables S1 and S2), then we compared the synthesis quality and time.

We selected IMP **10**, IMP **14**, IMP **16**, and IMP **23** to compare the flow, microwave, and batch peptide synthesis methods. We synthesized these IMPs on a 0.1 mmol scale by manually loading the *C*-terminal amino acid residue onto HMPB-ChemMatrix resin. We then coupled the subsequent amino acids with an excess of activated amino acid, according to previously published protocols for flow (10 equiv.)^20^, microwave (5 equiv.)^25^, and batch (12 equiv.)^26^ peptide synthesis. After completion of the syntheses, the peptides were cleaved from the resin and the protecting groups were removed with a trifluoroacetic acid cleavage cocktail. The peptides were then precipitated with diethyl ether (−80 °C), resuspended with CH_3_CN in H_2_O with 0.1% trifluoroacetic acid (TFA) and lyophilized. After lyophilization, we obtained the crude unpurified peptides as their TFA salts (white or yellow powder).

We then evaluated the quality of each synthesis by analyzing the unpurified IMPs using analytical RP-HPLC and LC/MS. The HPLC data were recorded using an Agilent Zorbax 5 µm 300SB-C3 column (2.1 × 150 mm) with a gradient of 5–65% CH_3_CN with 0.08% TFA in H_2_O with 0.1% TFA and a flow rate of 0.8 mL/min over 24 min (see Supplementary Fig. S1). We determined the peptide purity from each chromatogram by measuring the relative integrals of the peaks at 214 nm (see Fig. 2c). The average purity of the IMPs produced by flow synthesis was 38%, while the average purity of the IMPs produced by microwave and batch peptide synthesis was 30%. Corroboratory HPLC data were also recorded using a Phenomenex Aeris 3.6 µm WIDEPORE C4 column (see Supplementary Fig. S2). This analysis gave similar results: immunizing peptides produced by flow synthesis are comparable (IMPs **16** and **23**) or higher (IMPs **10** and **14**) in purity than those produced by microwave or batch synthesis. These results show that flow synthesis can generate peptides at a similar or higher purity compared to conventional synthesis methods (see Supplementary Fig. S3).

In addition, the automated flow technology substantially reduced the synthesis time. The markedly shorter synthesis time for flow synthesis reflects the efficiency of the technology rather than the reaction conditions. Each flow synthesis was complete in less than 35 min, and all four peptides were complete in less than three hours. By comparison, each microwave synthesis was complete after 4 to 6 h and each batch synthesis was complete after 24 to 48 h (see Fig. 2d, Supplementary Fig. S3).

### Automated flow peptide synthesis of a personalized neoantigen vaccine

We set out to produce at least 20 of 29 IMPs, which was the target number of peptides per vaccine in previous clinical trials^12,13^. We performed these syntheses on a 0.1 mmol scale by manually loading the *C*-terminal amino acid residue onto HMPB-ChemMatrix resin, then coupling the subsequent amino acids in flow. After the syntheses were complete (<35 min), we cleaved and lyophilized the peptides to obtain the unpurified peptides as the TFA salt (white or yellow powder). Mass spectrometry (ESI) analysis of the unpurified peptides showed that the desired mass was the main product for all 29 IMPs. Analytical RP-HPLC was then used to quantify the purity, which further indicated that automated flow peptide synthesis successfully produced all 29 IMPs. Supplementary Table S3 summarizes the purity and yield of each unpurified IMP. Supplementary Fig. S4 shows the corresponding RP-HPLC traces.

### Purification of the vaccine peptides

**Figure 3:**
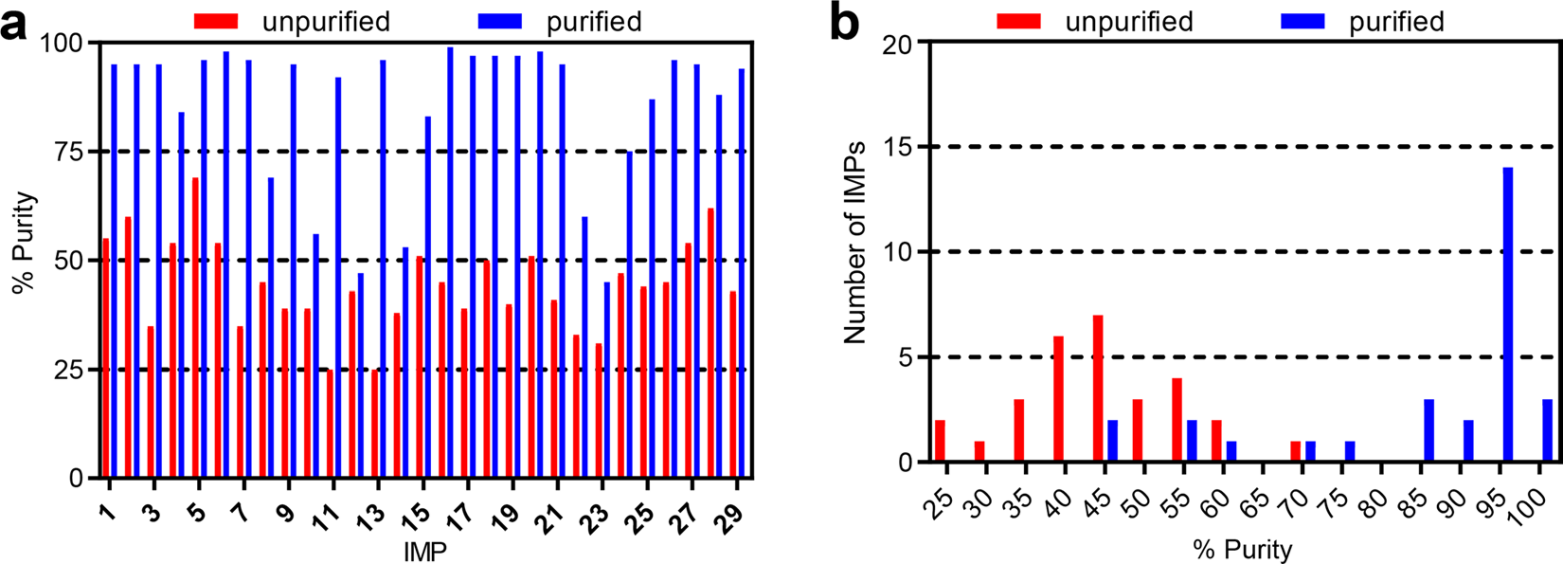
Characterization of IMPs produced by automated flow peptide synthesis. The graphs show (**a**) the individual purity and (**b**) the distribution of purities for IMPs **1**–**29**, before and after purification. The unpurified and purified purity is shown with red and blue bars, respectively.

We purified IMPs **1**–**29** to evaluate whether we could obtain these peptides in high purity for use in a vaccine (≥95%). The IMPs were purified by suspending them in a solution of CH_3_CN in H_2_O with 0.1% TFA, followed by preparative RP-HPLC and lyophilization of the clean fractions to obtain the IMPs as TFA salts (white powder). We then evaluated the purified IMPs by analytical RP-HPLC (see Supplementary Fig. S5), and confirmed the results with corroboratory RP-HPLC analysis (see Supplementary Fig. S6). Supplementary Table S3 summarizes the final purity, HPLC retention time, and isolated yield for each purified IMP, based on the cleavage of 0.05 mmol (50% of resin from a 0.1 mmol scale synthesis). Before purification, 3 IMPs were obtained with a purity of ≥60%, 7 with a purity between 50 and 59%, 9 with a purity between 40 and 49%, and 10 with a purity of <40% (see Fig. 3). After purification, 17 IMPs were obtained with a purity of ≥95%, 5 with a purity between 80 and 94%, and 7 with a purity of <80% (see Fig. 3). The yield based on the loading of the first amino acid varied from 2 to 27% (3 to 47 mg), and averaged 11% (19 mg).

These results illustrate that high-fidelity peptide synthesis can facilitate purification. For the 17 purified IMPs obtained with ≥95% purity, the unpurified precursors averaged 46% purity. For the 9 that remained lower in purity, the unpurified precursors averaged 43% purity. This observation shows that a higher quality peptide synthesis can facilitate purification and, in turn, accelerate production overall.

### High-throughput production of ASPs

Given the importance of shorter neoantigen peptides for immune monitoring, we determined whether we could rapidly produce ASPs using our automated flow synthesis technology. In patients vaccinated with 20 IMPs, approximately 40–50 of these peptides, 14–15 amino acids in length, are required for immune monitoring. Since these peptides are not used for immunizations, they can be produced with lower purity (>70%) and in lower amounts (1 mg). We selected a set of ASPs as a test case to synthesize by automated flow peptide synthesis, called ASPs **1**–**48**. Table 2 lists the sequences and lengths of the ASPs we produced by flow synthesis and also shows the final purity.

**Table 2:** Sequences from a set of ASPs for a personalized neoantigen vaccine.

	amino acid sequence	length	purity (%)
ASP **1**	ISTSSTIANILAAAV	15	86^*b*^
ASP **2**	IANILAAAVASISNQ	15	99^*b*^
ASP **3**	NNISNFFAKILFEEA	15	88^*a*^
ASP **4**	FAKILFEEANGRLVAS	16	92^*b*^
ASP **5**	SYEAYVLNIVRFLKK	15	88^*a*^
ASP **6**	YVLNIVRFLKKYKDSA	16	94^*a*^
ASP **7**	VRFLKKYKDSAQRDD	15	93^*a*^
ASP **8**	MEQGDWLIEGDLQVL	15	91^*b*^
ASP **9**	DWLIEGDLQVLDRVY	15	92^*a*^
ASP **10**	EGDLQVLDRVYWNDG	15	83^*a*^
ASP **11**	EQLRPLLASSLPLAV	15	73^*a*^
ASP **12**	LRPLLASSLPLAVRY	15	52^*a*^
ASP **13**^***c***^	YFQIGYMISLIAFFT	15	
ASP **14**^***c***^	ISLIAFFTNFYIQTY	15	
ASP **15**	HPSTVLDHKLEWVLY	15	78^*a*^
ASP **16**	HNLATYVFLHTMKGT	15	82^*a*^
ASP **17**	STVLDHKLEWVLYNE	15	78^*a*^
ASP **18**	RVTSAIHLIDSNTLQ	15	82^*a*^
ASP **19**	AIHLIDSNTLQVADI	15	65^*a*^
ASP **20**	IDSNTLQVADIDGST	15	82^*a*^
ASP **21**	TSISVHRYLGICHSL	15	54^*a*^
ASP **22**	HRYLGICHSLRALRW	15	88^*a*^
ASP **23**	ICHSLRALRWGRPRL	15	82^*a*^
ASP **24**	NPLYWNVVARWKHKT	15	90^*a*^
ASP **25**	NVVARWKHKTRKLSRA	16	82^*a*^
ASP **26**	KHKTRKLSRAFGSPY	15	95^*a*^
ASP **27**	ATYVFLHTMKGTPFE	15	65^*a*^
ASP **28**	VFLHTMKGTPFETPD	15	81^*a*^
ASP **29**	DRARREQERICLFSA	15	79^*a*^
ASP **30**	RREQERICLFSADPF	15	73^*a*^
ASP **31**	QERICLFSADPFDLE	15	96^*b*^
ASP **32**	SGSGVVSLHCLQHVV	15	76^*a*^
ASP **33**	VVSLHCLQHVVAVEA	15	86^*b*^
ASP **34**	HCLQHVVAVEAYTRE	15	84^*b*^
ASP **35**	LPHCSLIFPATNWIS	15	80^*a*^
ASP **36**	CSLIFPATNWISGGQ	15	97^*b*^
ASP **37**	IFPATNWISGGQNIT	15	86^*a*^
ASP **38**	SHEVLSHIFRYLSLQ	15	89^*a*^
ASP **39**	SHIFRYLSLQDIMCME	16	78^*a*^
ASP **40**	LSLQDIMCMESLSRK	15	64^*a*^
ASP **41**	RFNLIANQHLLAPGF	15	85^*a*^
ASP **42**	AAAFPSQRTSWEFLQ	15	84^*a*^
ASP **43**	SQRTSWEFLQSLVSIK	16	92^*a*^
ASP **44**	EFLQSLVSIKQEKPA	15	71^*a*^
ASP **45**^***c***^	DVFLSTTVFLMLSTT	15	
ASP **46**^***c***^	TVFLMLSTTCFLKYE	15	
ASP **47**	LHFIMPEKFSFWEDF	15	87^*a*^
ASP **48**	HFIMPEKFSFWEDFE	15	88^*a*^

^a^Determined by analytical RP-HPLC by integrating the peptide and impurity peaks at 214 nm.

^*b*^Determined by LC/MS by integrating the peptide and impurity ions observed in the mass spectrum.

^*c*^Efforts to purify this peptide were unsuccessful.

**Figure 4:**
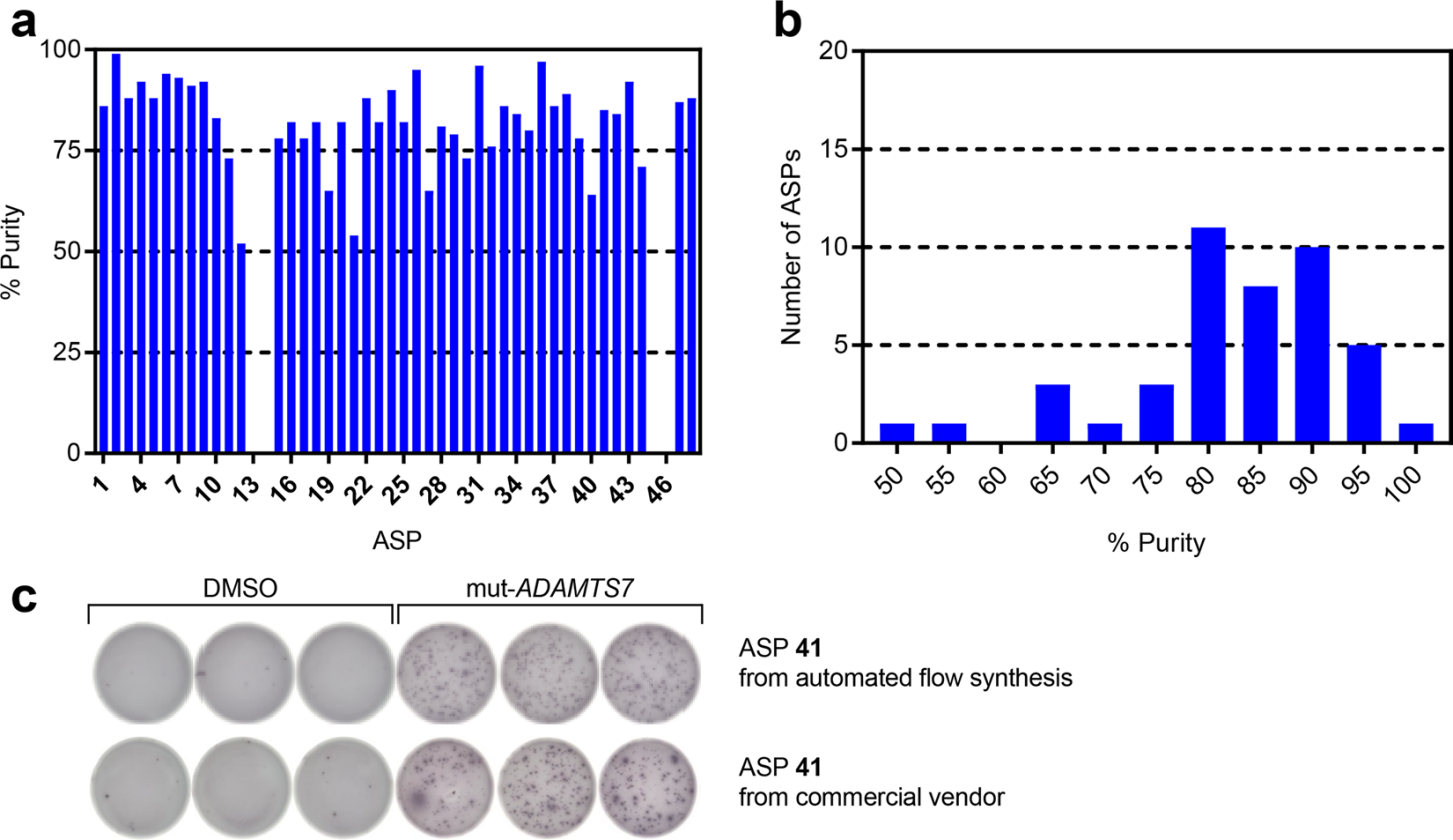
Characterization of purified ASPs produced by automated flow peptide synthesis. The graphs show (**a**) the individual purity and (**b**) the distribution of purity for ASPs **1**–**48** after purification. (**c**) IFN-γ secretion by neoantigen-specific T cells against mutated *ADAMTS7* (ASP **41**) peptide. The PBMCs were cultured with 2 μg/mL mut-*ADAMTS7* peptide for 14 days. 5 × 10^3^ T cells were then co-cultured overnight in ELISPOT wells with 1 × 10^4^ antigen presenting cells and with either DMSO or an mut-*ADAMTS7* peptide (10 μg/mL), followed by performing the IFN-γ ELISPOT assay.

We synthesized the ASPs in a similar fashion as the IMPs, but also developed an efficient workflow for isolating these peptides from resin in parallel. This workflow, combined with flow synthesis, permitted the production of ASPs in a remarkably high-throughput fashion. Figure 4 summarizes the ASP purity after synthesis and purification.

We were able to successfully produce all 48 ASPs by automated flow peptide synthesis. The syntheses were complete in less than 20 min. We then cleaved multiple ASPs from resin in parallel. The resin was rinsed with CH_2_Cl_2_ and aspirated under vacuum until dry. We transferred an aliquot (~100 mg) of the dried resin into a 24-well filter plate. Within the plate, each well contained a filter at the bottom and, underneath the plate, a syringe-like dispenser. After covering the dispensers with luer-lock syringe caps, we added a TFA cleavage solution (2 mL) to each well for 2 h. We precipitated the peptides by adding chilled (−80 °C) diethyl ether (5 mL) to each well, removing the syringe caps, and allowing the solution to drain. We performed two additional washes with diethyl ether, then added a solution of H_2_O/CH_3_CN (90:10) with 6 M guanidine at pH 2 (1 mL) to re-suspend the peptides in solution. We then used a vacuum manifold to drain the solutions into separate glass screw-top vials (1 mL) for immediate purification. We found that this procedure worked well for isolating ASPs in a high-throughput fashion on a small scale (100 mg of resin).

We then directly purified the ASPs by preparative RP-HPLC. After the purification, 39 of 48 ASPs were obtained in sufficient purity (>70%) and amounts (>1 mg) to use for *ex vivo* characterization of immune responses. The individual purity varied, where 10 were obtained with a purity >90%, 20 with a purity between 80 and 90%, 9 with a purity between 70 and 80%, and 5 with a purity <70%; only 4 peptides were not recovered (after purification).

We further evaluated the ASP quality by performing an immune monitoring assay. We selected ASP **41**, which was used in a previous clinical trial to analyze a patient immune response after administration of the corresponding neoantigen-targeting vaccine^13^. We performed an IFN-γ enzyme-linked immune absorbent spot (ELISPOT) assay to compare ASP **41** produced by flow synthesis with an identical peptide produced by a commercial peptide vendor. Patient-derived peripheral blood mononuclear cells (PBMCs) were stimulated with ASP **41** peptides from flow synthesis or the commercial vendor for 14 days. The ELISPOTs indicated that the ASP **41** from both flow synthesis and the commercial vendor generated an equivalent antigen-specific T cell response (see Fig. 4c, Supplementary Fig. S7). This finding establishes that the quality of ASPs produced by flow synthesis is sufficient for use in immune monitoring assays.

## Conclusion

We set out to facilitate production of peptides for personalized medicine, specifically in the context of neoantigen vaccines. We evaluated each step throughout the synthesis, cleavage, and purification of neoantigen peptides to establish a high-throughput workflow. Our efforts show that automated flow peptide synthesis can increase the rate and quality of peptide synthesis for IMP and ASP production. Flow synthesis produced IMPs with comparable or higher purity than either microwave or batch synthesis. Flow synthesis also permitted the production of a full set of neoantigen immunizing peptides, in addition to a full set of assay peptides that are of sufficient quality for use in immune monitoring assays.

Although automated flow peptide synthesis addresses the synthetic challenge of producing neoantigen peptides, obstacles in subsequent steps still prevent rapid production. The first challenge we encountered involved high-throughput cleavage of peptides from resin. Although we introduced a procedure for cleaving 24 peptides in parallel, further optimization is needed to implement this procedure on a larger scale. A second challenge is limited peptide solubility, which often delayed purification. A third challenge is peptide purification by preparative RP-HPLC, which sometimes requires multiple rounds to achieve high purity (≥95% purity). Although these challenges are a standard part of peptide production, they can delay the manufacturing of neoantigen vaccines and ultimately postpone patient treatment. Creative solutions to these challenges are urgently needed to fully address the peptide production and scalability problems for personalized neoantigen vaccines.

Our work illustrates how automated flow technology can enable rapid peptide synthesis for personalized neoantigen vaccines, which is useful in broader contexts. The advent of rapid and affordable genome-sequencing technology is likely to enable other classes of personalized medicine that use peptides, oligonucleotides, or artificial biopolymers. We anticipate that automated flow synthesis can be leveraged to produce these treatments by tailoring the solid-phase conditions to perform the corresponding coupling steps, which is part of ongoing work in our laboratory.

## Materials and Methods

### Materials

All reagents were purchased from commercial sources and used as received. *N*-α-Fmoc amino acids were purchased from CreoSalus or Novabiochem. *O*-(7-azabenzotriazol-1-yl)-*N,N,N*′*,N*′-tetramethyluronium hexafluorophosphate (HATU), (7-azabenzotriazol-1-yloxy)tripyrrolidinophosphonium hexafluorophosphate (PyAOP), *N*,*N*′-diisopropylcarbodiimide (DIC) were purchased from Chem-Impex. *N,N*-Dimethylformamide (DMF) was purchased from EMD Millipore. To each DMF bottle was added an AldraAmine trapping packet (Sigma-Aldrich) to minimize the accumulation of water and amine impurities. *N*,*N*-diisopropylethylamine (DIPEA), 4-(dimethylamino)pyridine (DMAP), piperidine, trifluoroacetic acid, triisopropylsilane, acetonitrile and 1,2-ethanedithiol (EDT) were purchased from Sigma-Aldrich. HMPB-ChemMatrix polyethylene glycol resin with a loading of ca. 0.5 mmol/g was purchased from Pcas Biomatrix.

### Resin loading

HMPB-ChemMatrix resin (200 mg, 0.5 mmol/g, 100–200 mesh) was suspended in ca. 5 mL of CH_2_Cl_2_ in a 6-mL fritted syringe and allowed to swell (15 min). The solution was drained, and the resin was rinsed three times with DMF and a solution was added of the first amino acid (1.0 mmol) with DIC (0.5 mmol, 78 µL) and DMAP (0.01 mmol, 50 µL of a 0.2 M solution in DMF) in 3.17 mL of DMF. The suspension was mixed gently and allowed to sit overnight (12–24 h). The solution was then drained and the resin was rinsed three times with DMF (5 mL).

### Automated flow peptide synthesis

We performed automated flow peptide synthesis on a ca. 0.1 mmol scale by manually loading the *C*-terminal amino acid residue onto HMPB-ChemMatrix resin, and by adding the subsequent amino acids by automated flow peptide synthesis^20^.

The reagent storage bottles on the synthesizer contain stock solutions in DMF of amino acids (0.4 M), activating agents (0.38 M HATU or PyAOP), and the deprotecting agent (40% piperidine), as well as the activating base (DIPEA, neat). The amino acid and activating agent stock solutions are mixed during each coupling step to deliver 10 equiv. of activated amino acid to the resin. The concentrations of these stock solutions can be reduced (0.2 M amino acid and 0.19 M activating agent (HATU or PyAOP)) to deliver 5 equiv. of activated amino acid, which does not extend the synthesis time and only marginally reduces the synthesis quality for ~30-mer peptides^27^.

The three pumps are Varian Prostar 210 HPLC pumps, of which two are fitted with 50 mL/min pump heads (400 μL of liquid per pump stroke) and deliver the amino acids and activating agents, whereas the third is fitted with a 5 mL/min pump head (40 μL of liquid per pump stroke) and delivers DIPEA. The two heating loops are a 10-ft stainless-steel loop at 90 °C and a 5-ft stainless-steel loop at 25 °C, which heat the solutions prior to flowing over the resin. The reactor is a stainless steel chamber for holding a fritted syringe and is heated to 90 °C. The UV-vis spectrophotometer monitors the absorbance at 312 nm, which allows relative quantitation of Fmoc removal during each coupling and deprotection step. The coupling and deprotection cycles are described in Supplementary Table S1.

The automated flow synthesis begins by prewashing the resin with DMF (80 mL/min, 20 s, 90 °C) and performing an initial deprotection of the first amino acid, followed by five automated steps that perform coupling, deprotection, and washing as follows: (1) This step primes the lines with the corresponding amino acid and coupling agent. Two pumps simultaneously flow at 40 mL/min for 5 strokes and a volume of 1.6 mL each (total volume of 3.2 mL); (2) This step performs the standard coupling. Amino acid, coupling agent, and activator base solution flow to the resin using three pumps, two pumps simultaneously flow amino acid (11 equiv.) and coupling agent (10 equiv.) solutions at 40 mL/min for 7 strokes and a volume of 2.8 mL each (total volume of 5.6 mL), and one pump delivers DIPEA at 4 mL/min for 7 strokes and a volume of 0.28 mL; (3) This step washes the lines with DMF. Two pumps simultaneously flow DMF through the lines at 40 mL/min for 35 strokes and a volume of 14 mL each (total volume of 28 mL); (4) This step performs the deprotection. Two pumps simultaneously flow at 40 mL/min for 13 strokes, one pump delivers a solution of 40% piperidine (5.2 mL) and the other delivers DMF (5.2 mL). These solutions combine to give 20% piperidine at a flow rate of 80 mL/min and a volume of 10.4 mL; (5) This step washes the lines with DMF (same as step 3). Two pumps simultaneously flow through the lines at 40 mL/min for 35 strokes and a volume of 14 mL each (total volume of 28 mL).

These five steps were repeated for each amino acid until the peptide total synthesis was complete. At the end of the synthesis, the resin was manually washed four times with DMF (5 mL) and four times with CH_2_Cl_2_ (5 mL).

### Microwave peptide synthesis

We performed the microwave peptide syntheses on a ca. 0.1 mmol scale by manually loading the *C*-terminal amino acid residue onto HMPB-ChemMatrix resin, and by adding the subsequent amino acids by microwave peptide synthesis^25^. The syntheses were performed on a CEM Liberty Blue peptide synthesizer with optimized conditions: amino acid solutions (0.2 M in DMF); activator base DIPEA (0.5 M in DMF); coupling reagent HATU (0.5 M in DMF), and washing solvent DMF. The deprotection and coupling steps were performed according to the recommended sequences from the CEM Corporation Liberty Blue User Guide (Rev. 4), which guided the amount of coupling reagents used for the amino acids (5 equiv.) and HATU (5 equiv.). The coupling and deprotection cycles are described in Supplementary Table S2. At the end of the synthesis, the resin was manually washed four times with DMF (5 mL) and four times with CH_2_Cl_2_ (5 mL).

### Batch peptide synthesis

We performed batch peptide synthesis on a ca. 0.1 mmol scale by manually loading the *C*-terminal amino acid residue onto HMPB-ChemMatrix resin, and by also manually adding the subsequent amino acids^26^. Each deprotection was performed twice, by adding a solution of 20% piperidine in DMF to the resin, stirring gently, and draining. The first deprotection treatment was performed for 1 min and the second for 10 min. Each coupling was also performed twice by adding a solution of the amino acid (0.6 mmol) and HBTU (0.6 mmol) in 2.4 mL DMF. We used HBTU for the batch syntheses, rather than HATU, because a previously reported procedure demonstrated that HBTU works well in batch peptide synthesis^26^. The first coupling treatment was performed by adding the solution of amino acid (6 equiv.) and DIPEA (1.2 mmol, 0.21 mL) to the resin, and by stirring gently. After 30 min, the amino acid solution was replaced for a second coupling (6 equiv.). The resin was washed three times with DMF after each deprotection and after each coupling step. At the end of the synthesis, the resin was washed four times with DMF (5 mL) and four times with CH_2_Cl_2_ (5 mL).

### Resin cleavage

After each synthesis was complete, we cleaved the peptides from the resin and removed acid-labile protecting groups under acidic conditions. The cleavages were performed on half of the resin from each synthesis (ca. 0.05 mmol of peptide) with the treatment of a 94/2.5/2.5/1 mixture (5 mL) of trifluoroacetic acid (TFA), water, ethane dithiol, and triisopropyl silane for 1 h at room temperature. More material can be obtained, if needed, after performing an additional resin cleavage and purification of the remaining ~0.05 mmol. The peptides were then washed three times by adding cold diethyl ether (40 mL, chilled to −80 °C in dry ice), mixing well, centrifuging (4000 rpm, 5 min), and decanting the supernatant. The remaining pellets were resuspended in 50% CH_3_CN in H_2_O with 0.1% TFA, filtered through a 0.2 µm nylon filter, frozen in liquid nitrogen, and lyophilized to give the unpurifed peptides as a white or yellow powder.

### RP-HPLC analysis of IMPs by the commercial peptide vendor

The IMPs previously produced by the commercial vendor for in-human use were analyzed at 0.4 mg/mL on an Agilent 1200 HPLC system with a Phenomenex Luna 5 µm C18(2) column (4.6 mm × 250 mm) with a 0–70% gradient of CH_3_CN in H_2_O with 0.1% TFA and a flow rate of 1.5 mL/min.

### RP-HPLC and LC/MS analysis of unpurified and purified IMPs

Lyophilized peptides were resuspended at 1 mg/mL in a 1:1 solution of H_2_O/CH_3_CN with 0.1% TFA, then analyzed on an Agilent 1200 HPLC system using an Agilent Zorbax 5 µm 300SB-C3 column (2.1 mm × 150 mm) with a 5–65% gradient of CH_3_CN with 0.08% TFA in H_2_O with 0.1% TFA and a flow rate of 0.8 mL/min. Representative IMPs were also characterized using two additional columns: Phenomenex Aeris 3.6 µm WIDEPORE C4 column (4.6 mm × 150 mm) with a 5–65% gradient of CH_3_CN with 0.08% TFA in H_2_O with 0.1% TFA and a flow rate of 0.8 mL/min; and Phenomenex Luna 5 µm C18(2) column (4.6 mm × 250 mm) with a 0–70% gradient of CH_3_CN in H_2_O with 0.1% TFA and a flow rate of 1.5 mL/min.

A 1:100 dilution of each 1 mg/mL peptide solution was prepared and analyzed by LC/MS on an Agilent 6550 ESI-Q-TOF mass spectrometer equipped with an Agilent Poroshell 5 µm 300SB-C3 column (1 mm × 75 mm) with a 1–91% gradient of CH_3_CN in H_2_O with 0.1% formic acid and a flow rate of 0.4 mL/min.

### RP-HPLC purification of IMPs

The peptides were purified with an Agilent Zorbax 7 µM SB-C18 Prep HT column (21.2 mm × 250 mm) with a 10–59% gradient over 98 min of CH_3_CN in H_2_O with 0.1% TFA and a flow rate of 15.0 mL/min. The pure fractions were combined, frozen in liquid nitrogen, and lyophilized to give the peptides as a white powder in 2–27% yield (3–47 mg) based on recovery from cleaving 50% of resin from a 0.1 mmol scale synthesis.

### Patient samples

Patients with high-risk melanoma provided informed consent and were enrolled between April 2014 and October 2015 to a single center, phase I clinical trial approved by the Dana-Farber/Harvard Cancer Center Institutional Review Board (NCT01970358)^13^. This study was conducted in accordance with the Declaration of Helsinki. Heparinized blood samples were obtained from study participants on Institutional Review Board-approved protocols at the DFCI. Patient PBMCs were isolated by Ficoll/Hypaque density-gradient centrifugation (GE Healthcare) and cryopreserved with 10% DMSO in FBS (Sigma-Aldrich). Cells from patients were stored in vapour-phase liquid nitrogen until the time of analysis.

### Generation and detection of patient neoantigen-specific T cells

These experiments were performed as described in a previous publication^13^. PBMCs were cultured in RPMI-1640 medium supplemented with L-glutamine, nonessential amino acids, HEPES, β-mercaptoethanol, sodium pyruvate, penicillin/streptomycin (Gibco), and 10% AB-positive heat-inactivated human serum (Gemini Bioproduct). For *in vitro* expansion of antigen-specific T cells, PBMCs were stimulated in 24-well cell culture plates at 5 × 10^6^ cells per well with individual peptides (each at 2 μg/mL) in the presence of IL-7 (20 ng/mL; R&D Systems). On day 3, low-dose IL-2 (20 U/mL; Amgen) was added. Half-medium change and supplementation of cytokines were performed every 3 days. After 14 days, T-cell specificity was tested against the peptide by interferon (IFN)-γ ELISPOT.

### IFN-γ ELISPOT assay

These experiments were also performed as described in a previous publication^13^. IFN-γ ELISPOT assays were performed using 96-well MultiScreen Filter Plates (Millipore), coated with 2 μg/mL anti-human IFN-γ mAb overnight (1-D1K, Mabtech). Plates were washed with PBS and blocked with complete RPMI before use. 5 × 10^3^ T cells were co-cultured with 1 × 10^4^ autologous CD4^+^ and CD8^+^ T cell-depleted PBMCs (APC). Peptides (10 μg/mL) were directly added to the ELISPOT wells with APCs and incubated with T cells overnight in complete RPMI at 37 °C. The plates were rinsed with PBS containing 0.05% Tween-20 and then 1 μg/mL anti-human IFN-γ mAb (7-B6-1-Biotin, Mabtech) was added, followed by Streptavidin-ALP (Mabtech). After rinsing, SIGMA FAST BCIP/NBT (5-bromo-4-chloro-3-indolyl phosphate/nitro blue tetrazolium; Sigma-Aldrich) was used to develop the immunospots, then the spots were imaged and counted (Cellular Technology Limited).

## Supplementary information

Supplementary information.
