# Supplementary material for: Automated Flow Synthesis of Tumor Neoantigen Peptides for Personalized Immunotherapy
Source: Sci Rep. 2020 Jan 20;10:723. doi: 10.1038/s41598-019-56943-5 (PMC6971261; doi:10.1038/s41598-019-56943-5)

Supplementary Information for

Automated Flow Synthesis of Tumor Neoantigen Peptides for Personalized Immunotherapy

Nicholas L. Truex,[1†] Rebecca L. Holden,[1†] Bin-you Wang,[1] Pu-guang Chen,[1] Stephanie Hanna,[1] Zhuting Hu,[2] Keerthi Shetty,[2,3] Oriol Olive,[2] Donna Neuberg,[4] Nir Hacohen,[5,6,7] Derin B. Keskin,[2,3,5,6,8] Patrick A. Ott,[2,5,8] Catherine J. Wu,[2,3,5,6,7,8]* and Bradley L. Pentelute[1,6,9]*

[†]N.L.T and R.L.H. contributed equally to this work.

[1]Department of Chemistry, Massachusetts Institute of Technology, 77 Massachusetts Avenue, Cambridge, MA 02139, USA, [2]Department of Medical Oncology, Dana-Farber Cancer Institute, Boston, MA 02215, USA, [3]Translational Immunogenomics Laboratory, Dana-Farber Cancer Institute, Boston, MA 02215, USA, [4]Department of Biostatistics and Computational Biology, Dana-Farber Cancer Institute, Boston, MA 02215, USA, [5]Harvard Medical School, Boston, MA 02215, USA, [6]Broad Institute of MIT and Harvard, Cambridge, MA 02142, USA, [7]Center for Cancer Research, Massachusetts General Hospital, Boston, MA 02114, USA, [8]Department of Medicine, Brigham and Women's Hospital, Boston, MA 02215, USA. [9]Koch Institute for Integrative Cancer Research, Massachusetts Institute of Technology, Cambridge, MA 02139, USA.

*To whom correspondence should be addressed: cwu@partners.org; blp@mit.edu

I. SUPPLEMENTARY FIGURES AND TABLES

Supplementary Table S1. Summary of automated flow peptide synthesis conditions.

Supplementary Table S2. Summary of microwave peptide synthesis conditions.

Supplementary Figure S1. Analytical RP-HPLC traces of unpurified IMP **10**, IMP **14**, IMP **16**, and IMP **23** using an Agilent Zorbax column.

Supplementary Figure S2. Analytical RP-HPLC traces of unpurified IMP **10**, IMP **14**, IMP **16**, and IMP **23** using a Phenomenex Aeris column.

Supplementary Figure S3. Synthesis time, mass, purity, and yield data for each unpurified IMP **10**, IMP **14**, IMP **16**, and IMP **23**.

Supplementary Table S3. Automated flow synthesis purity and yield data for IMPs **1**–**29**.

Supplementary Figure S4. Analytical RP-HPLC traces of unpurified IMPs **1**–**29** using an Agilent Zorbax column.

Supplementary Figure S5. Analytical RP-HPLC traces of purified IMPs **1**–**29** using an Agilent Zorbax column.

Supplementary Figure S6. Analytical RP-HPLC traces of purified IMP **10**, IMP **14**, and IMP **16** using Aeris, Luna, and Zorbax columns.

Supplementary Figure S7. IFN-γ secretion by neoantigen-specific T cell lines against mutated *ADAMTS7* (ASP **41**) peptide.

II. CHARACTERIZATION DATA

CHARACTERIZATION OF IMPS FROM AUTOMATED FLOW PEPTIDE SYNTHESIS

UV absorbance trace from the automated flow peptide synthesis
RP-HPLC chromatograms of the unpurified and purified peptides
Mass spectrum (ESI) of the purified peptide

IMP **1** 13
IMP **2** 14
IMP **3** 15
IMP **4** 16
IMP **5** 17
IMP **6** 18
IMP **7** 19
IMP **8** 20
IMP **9** 21
IMP **10** 22
IMP **11** 23
IMP **12** 24
IMP **13** 25
IMP **14** 26
IMP **15** 27
IMP **16** 28
IMP **17** 29
IMP **18** 30
IMP **19** 31
IMP **20** 32
IMP **21** 33
IMP **22** 34
IMP **23** 35
IMP **24** 36
IMP **25** 37
IMP **26** 38
IMP **27** 39
IMP **28** 40
IMP **29** 41

CHARACTERIZATION OF IMPS FROM COMMERCIAL VENDER

RP-HPLC chromatogram of the purified peptide
Mass spectrum (ESI) of the purified peptide

IMP **1** 42
IMP **2** 42
IMP **5** 43
IMP **6** 43
IMP **7** 44
IMP **9** 44
IMP **15** 45
IMP **16** 45
IMP **17** 46
IMP **19** 46
IMP **21** 47
IMP **24** 47
IMP **25** 48
IMP **26** 48
IMP **27** 49
IMP **28** 49
IMP **29** 50

CHARACTERIZATION OF ASPS FROM AUTOMATED FLOW PEPTIDE SYNTHESIS

RP-HPLC chromatogram of the purified peptide
Mass spectrum (ESI) of the purified peptide

ASP **1** 51
ASP **2** 51
ASP **3** 52
ASP **4** 52
ASP **5** 53
ASP **6** 53
ASP **7** 54
ASP **8** 54
ASP **9** 55
ASP **10** 55
ASP **11** 56
ASP **12** 56
ASP **15** 57
ASP **16** 57
ASP **17** 58
ASP **18** 58
ASP **19** 59
ASP **20** 59

CHARACTERIZATION OF ASPS FROM AUTOMATED FLOW PEPTIDE SYNTHESIS (CONTINUED)

ASP **21** 60
ASP **22** 60
ASP **23** 61
ASP **24** 61
ASP **25** 62
ASP **26** 62
ASP **27** 63
ASP **28** 63
ASP **29** 64
ASP **30** 64
ASP **31** 65
ASP **32** 65
ASP **33** 66
ASP **34** 66
ASP **35** 67
ASP **36** 67
ASP **37** 68
ASP **38** 68
ASP **39** 69
ASP **40** 69
ASP **41** 70
ASP **42** 70
ASP **43** 71
ASP **44** 71
ASP **47** 72
ASP **48** 72

I. SUPPLEMENTAL FIGURES AND TABLES

Supplementary Table S1. Summary of coupling and deprotection steps performed during automated flow peptide synthesis.

step #	standard coupling and deprotection	arginine[a] deprotection and coupling	histidine[b] deprotection and coupling
**1**	amino acid (5 strokes, 1.6 mL)<br>HATU (5 strokes, 1.6 mL)<br>90 °C heating loop; 90 °C reactor<br>80 mL/min	arginine (5 strokes, 1.6 mL)<br>PyAOP (5 strokes, 1.6 mL)<br>90 °C heating loop; 90 °C reactor<br>40 mL/min	amino acid (5 strokes, 1.6 mL)<br>HATU (5 strokes, 1.6 mL)<br>25 °C heating loop; 90 °C reactor<br>40 mL/min
**2**	amino acid (7 strokes, 2.8 mL)<br>HATU (7 strokes, 2.8 mL)<br>DIPEA (7 strokes, 0.28 mL)<br>90 °C heating loop; 90 °C reactor<br>80 mL/min	arginine (14 strokes, 5.6 mL)<br>PyAOP (14 strokes, 5.6 mL)<br>DIPEA (14 strokes, 0.56 mL)<br>90 °C heating loop; 90 °C reactor<br>40 mL/min	amino acid (7 strokes, 2.8 mL)<br>HATU (7 strokes, 2.8 mL)<br>DIPEA (7 strokes, 0.28 mL)<br>25 °C heating loop; 90 °C reactor<br>40 mL/min
**3**	DMF (35 strokes, 28 mL)<br>90 °C heating loop; 90 °C reactor<br>80 mL/min	DMF (35 strokes, 28 mL)<br>90 °C heating loop; 90 °C reactor<br>80 mL/min	DMF (35 strokes, 28 mL)<br>90 °C heating loop; 90 °C reactor<br>80 mL/min
**4**	40% Piperidine (13 strokes, 5.2 mL)<br>DMF (13 strokes, 5.2 mL)<br>90 °C heating loop; 90 °C reactor<br>80 mL/min	40% Piperidine (13 strokes, 5.2 mL)<br>DMF (13 strokes, 5.2 mL)<br>90 °C heating loop; 90 °C reactor<br>80 mL/min	40% Piperidine (13 strokes, 5.2 mL)<br>DMF (13 strokes, 5.2 mL)<br>90 °C heating loop; 90 °C reactor<br>80 mL/min
**5**	DMF (35 strokes, 28 mL)<br>90 °C heating loop; 90 °C reactor<br>80 mL/min	DMF (35 strokes, 28 mL)<br>90 °C heating loop; 90 °C reactor<br>80 mL/min	DMF (35 strokes, 28 mL)<br>90 °C heating loop; 90 °C reactor<br>80 mL/min

[a]Arginine was coupled using a double coupling cycle, because the coupling efficiency of arginine is lower upon its formation of a γ-lactam.
[b]Histidine was coupled at lower temperatures to minimize epimerization.

Supplementary Table S2. Summary of coupling and deprotection steps performed during microwave peptide synthesis.

step #	standard deprotection and coupling	arginine[a] deprotection and coupling	histidine[b] deprotection and coupling
**1**	20% Piperidine (4 mL)<br>75 °C for 15 s; 90 °C for 50 s	20% Piperidine (4 mL)<br>75 °C for 30 s	20% Piperidine (4 mL)<br>75 °C for 15 s; 90 °C for 50 s
**2**	DMF (4 mL × 4)	20% Piperidine (4 mL)<br>75 °C for 180 s	DMF (4 mL × 4)
**3**	amino acid (2.5 mL)<br>HATU (1 mL); DIPEA (1 mL)<br>75 °C for 15 s; 90 °C for 110 s	DMF (4 mL × 4)	amino acid (2.5 mL)<br>HATU (1 mL); DIPEA (1 mL)<br>25 °C for 120 s; 50 °C for 480 s
**4**	DMF (4 mL × 2)	amino acid (2.5 mL)<br>HATU (1 mL); DIPEA (1 mL)<br>75 °C for 15 s; 90 °C for 110 s	DMF (4 mL × 2)
**5**		DMF (4 mL)	
**6**		amino acid (2.5 mL)<br>HATU (1 mL); DIPEA (1 mL)<br>75 °C for 15 s; 90 °C for 110 s	

[a]Arginine was coupled using a double coupling cycle, because the coupling efficiency of arginine is lower upon formation of a γ-lactam.
[b]Histidine was coupled at lower temperatures to minimize epimerization.

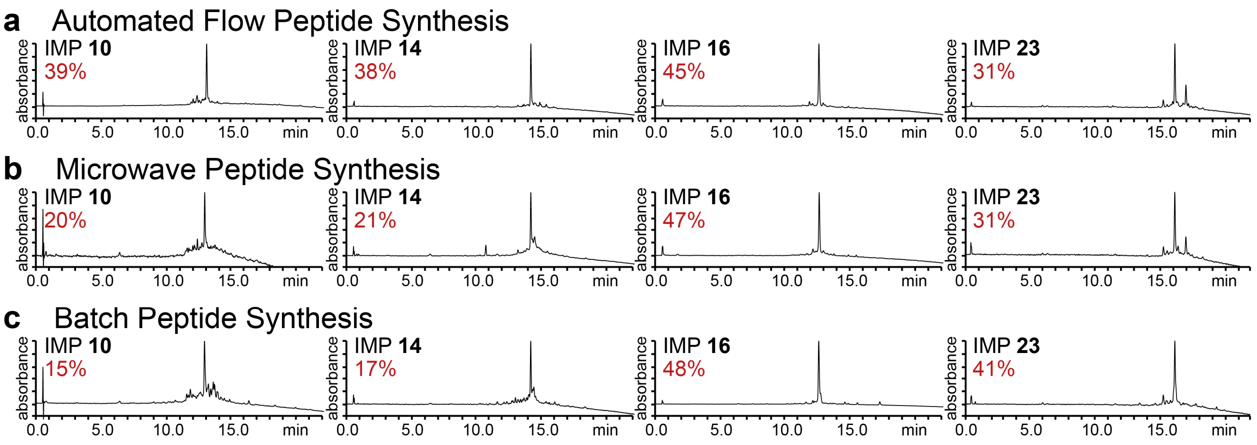

Supplementary Figure S1. Analytical RP-HPLC traces of IMP **10**, IMP **14**, IMP **16**, and IMP **23** produced by (a) automated flow, (b) microwave, and (c) batch synthesis. Conditions: Agilent Zorbax 5 µm 300SB-C3 column (2.1 × 150 mm) with a gradient of 5–65% CH_3CN with 0.08% TFA in H_2O with 0.1% TFA and a flow rate of 0.8 mL/min over 24 min.

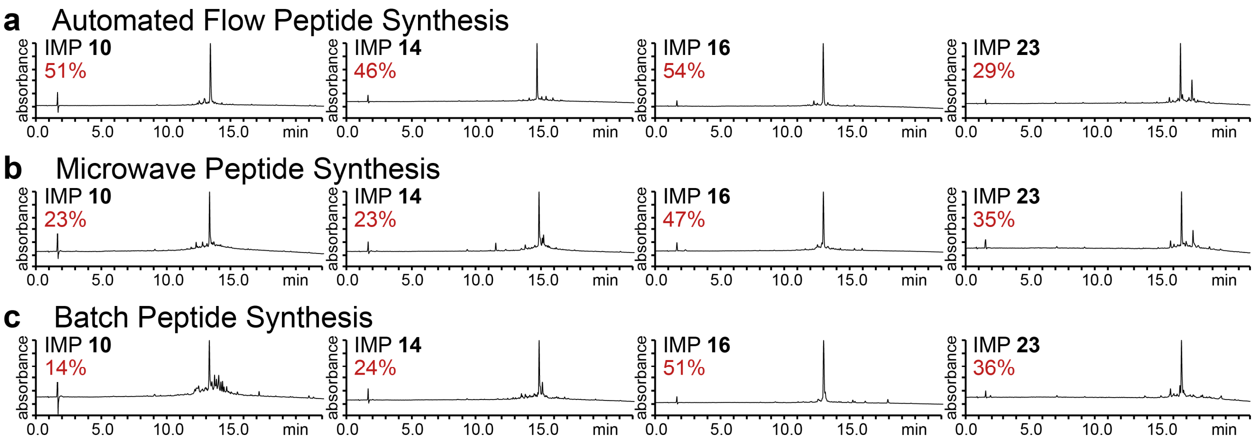

Supplementary Figure S2. Analytical RP-HPLC traces of IMP **10**, IMP **14**, IMP **16**, and IMP **23** produced by (a) automated flow, (b) microwave, and (c) batch synthesis. Conditions: Phenomenex Aeris 3.6 µm WIDEPORE C4 column (4.6 × 150 mm) with a gradient of 5–65% CH_3CN with 0.08% TFA in H_2O with 0.1% TFA and a flow rate of 0.8 mL/min over 24 min.

IMP **10**

synthesis method	synthesis time (hh:mm)	mass (mg)	purity (%)	yield (%)
**flow**	00:31	107	39	22
**microwave**	04:30	55	20	6
**batch**	32:48	45	15	4

IMP **14**

synthesis method	synthesis time (hh:mm)	mass (mg)	purity (%)	yield (%)
**flow**	00:31	123.5	38	29
**microwave**	02:30	27	21	4
**batch**	36:18	124	17	13

IMP **16**

synthesis method	synthesis time (hh:mm)	mass (mg)	purity (%)	yield (%)
**flow**	00:30	93	45	24
**microwave**	03:06	82	47	22
**batch**	33:00	95	48	26

IMP **23**

synthesis method	synthesis time (hh:mm)	mass (mg)	purity (%)	yield (%)
**flow**	00:30	59	31	11
**microwave**	03:30	12	31	2
**batch**	32:54	59	41	15

Supplementary Figure S3. Summary of synthesis data for unpurified IMPs **10**, **14**, **16**, and **23** synthesized by flow, microwave, and batch peptide synthesis. Each graph shows synthesis time, isolated mass (mg), unpurified purity (%), and isolated yield (%). Purity (%) was determined with analytical RP-HPLC using an Agilent Zorbax 5 µm 300SB-C3 column (2.1 × 150 mm) by integrating the chromatogram peaks at 214 nm. Yield (%) was calculated based on the mass and purity of recovered peptide from a resin cleavage on a 0.05 mmol scale.

Supplementary Table S3. Summary of yield and purity data for unpurified and purified IMPs **1–29** synthesized by automated flow peptide synthesis**.**

	average M.W. (g/mol) [a]	automated flow synthesis time (min)	unpurified yield (mg)[b,c]	unpurified purity (%)[d,e]	purified yield (mg) [b,c]	purified yield (%)[c]	ret. time by RP-HPLC	purified purity (%)[d,e]	commercial vendor purity (%)[d,f]
IMP **1**	3096.82	31	101.1	55	18.1	10	17.5	95	94
IMP **2**	3096.76	30	95.9	60	31.5	16	13.5	95	89
IMP **3**	3082.67	31	93.5	35	26.3	15	18.2	95	
IMP **4**	3591.36	35	141.4	54	36.6	16	13.7	84	
IMP **5**	3396.78	35	100.5	69	50.8	24	11.7	96	94
IMP **6**	2983.37	33	109.6	54	30.4	17	13.5	98	96
IMP **7**	2957.41	32	77.8	35	7.3	4	14.4	96	99
IMP **8**	2666.00	30	26.6	45	8.0	5	17.0	69	
IMP **9**	3482.88	33	103.8	39	46.6	24	15.9	95	95
IMP **10**	3157.56	31	107.3	39	6.9	4	13.0	56	
IMP **11**	2895.30	34	100.2	25	25.7	14	12.5	92	
IMP **12**	2877.21	32	73.8	43	5.1	3	13.0	47	
IMP **13**	2765.16	30	86.9	25	3.3	2	14.2	96	
IMP **14**	2966.26	31	123.5	38	3.5	2	14.3	53	
IMP **15**	2544.81	27	47.7	51	16.1	12	13.6	83	92
IMP **16**	2770.12	30	93.1	45	47.2	27	12.6	99	93
IMP **17**	2432.91	25	59.9	39	9.3	6	15.8	97	98
IMP **18**	2885.41	31	51.6	50	9.7	6	19.0	97	
IMP **19**	2537.80	27	44.8	40	8.3	6	14.2	97	96
IMP **20**	2604.90	27	53.0	51	7.0	5	13.5	98	
IMP **21**	3216.59	33	108.1	41	30.6	15	13.6	95	90
IMP **22**	2377.83	24	35.1	33	12.4	9	18.7	60	
IMP **23**	2860.24	30	59.1	31	5.5	3	16.4	45	
IMP **24**	3071.52	31	82.3	47	6.2	3	10.7	75	99
IMP **25**	2872.26	30	80.7	44	32.1	19	14.0	87	93
IMP **26**	3069.48	34	65.9	45	18.8	10	13.5	96	97
IMP **27**	2243.53	22	78.2	54	21.5	16	11.9	95	99
IMP **28**	2849.25	30	91.3	62	13.3	8	13.4	88	98
IMP **29**	2634.00	31	34.7	43	11.3	7	12.2	95	97

[a]Calculated molecular weight.
[b]Determined gravimetrically by weighing the peptide on an analytical balance.
[c]Based on recovery from cleaving 50% of resin from a 0.1 mmol peptide synthesis.
[d]Determined by analytical RP-HPLC by integrating the peptide and impurity peaks at 214 nm.
[e]Zorbax 5 µm 300SB-C3 column (2.1 × 150 mm), 5–65% of CH_3CN (0.08% TFA) in H_2O (0.1% TFA) at 0.8 mL/min over 24 min.
[f]Phenomenex Luna 5 µm C18(2) column (4.6 × 250 mm), 0–70% of CH_3CN (0.1% TFA) in H_2O (0.1% TFA) at 1.5 mL/min over 20 min.

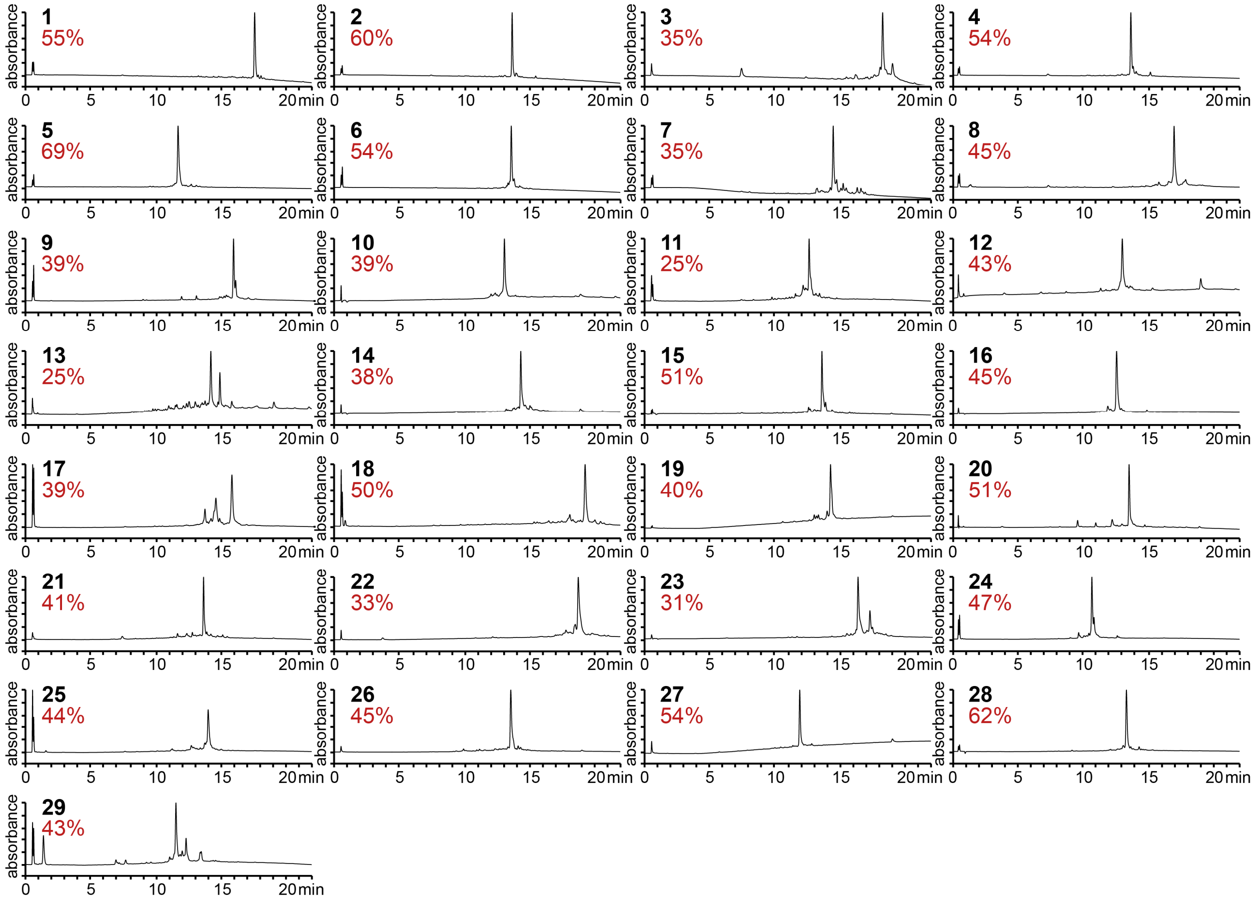

Supplementary Figure S4. Unpurified neoantigen peptides produced by automated flow peptide synthesis. Analytical RP-HPLC traces of the unpurified IMPs **1**–**29**. The relative integration of each IMP is shown on its corresponding trace (red), and was determined by integrating the peptide and impurity peaks at 214 nm. Conditions: Agilent Zorbax 5 µm 300SB-C3 column (2.1 × 150 mm) with a gradient of 5–65% CH_3CN with 0.08% TFA in H_2O with 0.1% TFA and a flow rate of 0.8 mL/min over 24 min.

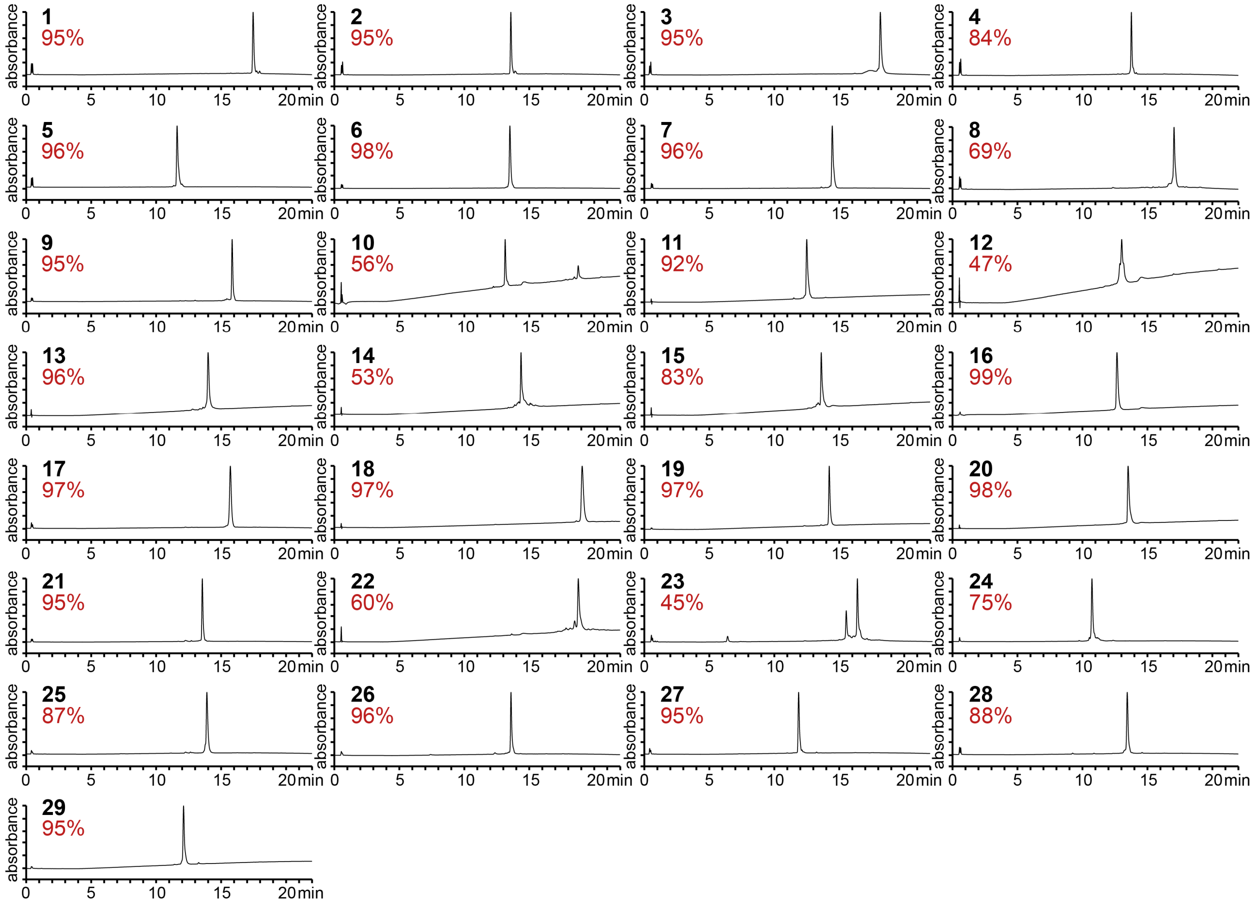

Supplementary Figure S5. Purified neoantigen peptides produced by automated flow peptide synthesis. Analytical HPLC traces of the purified IMPs **1**–**29**. The relative integration of each IMP is shown on its corresponding trace (red), and was determined by integrating the peptide and impurity peaks at 214 nm. Conditions: Agilent Zorbax 5 µm 300SB-C3 column (2.1 × 150 mm) with a gradient of 5–65% CH_3CN with 0.08% TFA in H_2O with 0.1% TFA and a flow rate of 0.8 mL/min over 24 min.

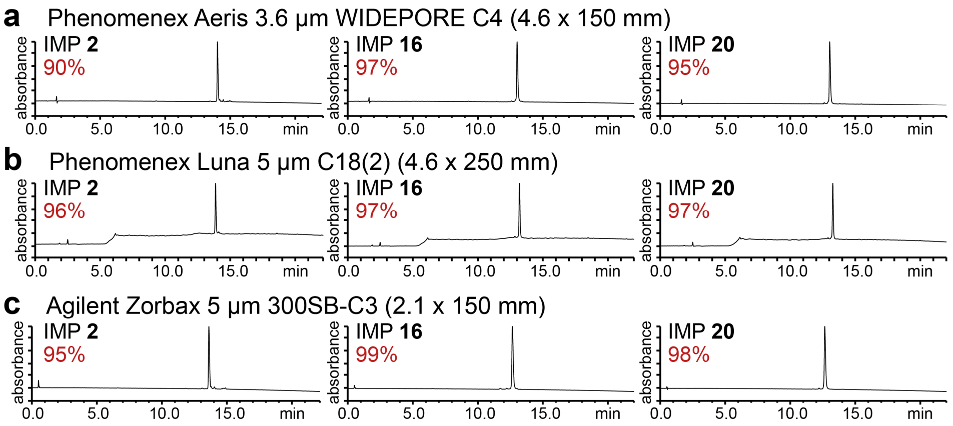

Supplementary Figure S6. Analytical RP-HPLC traces of purified IMP **2**, IMP **16**, and IMP **20**. Conditions: (a) Phenomenex Aeris 3.6 µm WIDEPORE C4 column (4.6 × 150 mm) with a gradient of 5–65% CH_3CN with 0.08% TFA in H_2O with 0.1% TFA and a flow rate of 0.8 mL/min over 24 min; (b) Phenomenex Luna 5 µm C18(2) column (4.6 × 250 mm) with a gradient of 0–70% CH_3CN in H_2O with 0.1% TFA and a flow rate of 1.5 mL/min over 20 min; and (c) Agilent Zorbax 5 µm 300SB-C3 column (2.1 × 150 mm) with a gradient of 5–65% CH_3CN with 0.08% TFA in H_2O with 0.1% TFA and a flow rate of 0.8 mL/min over 24 min.

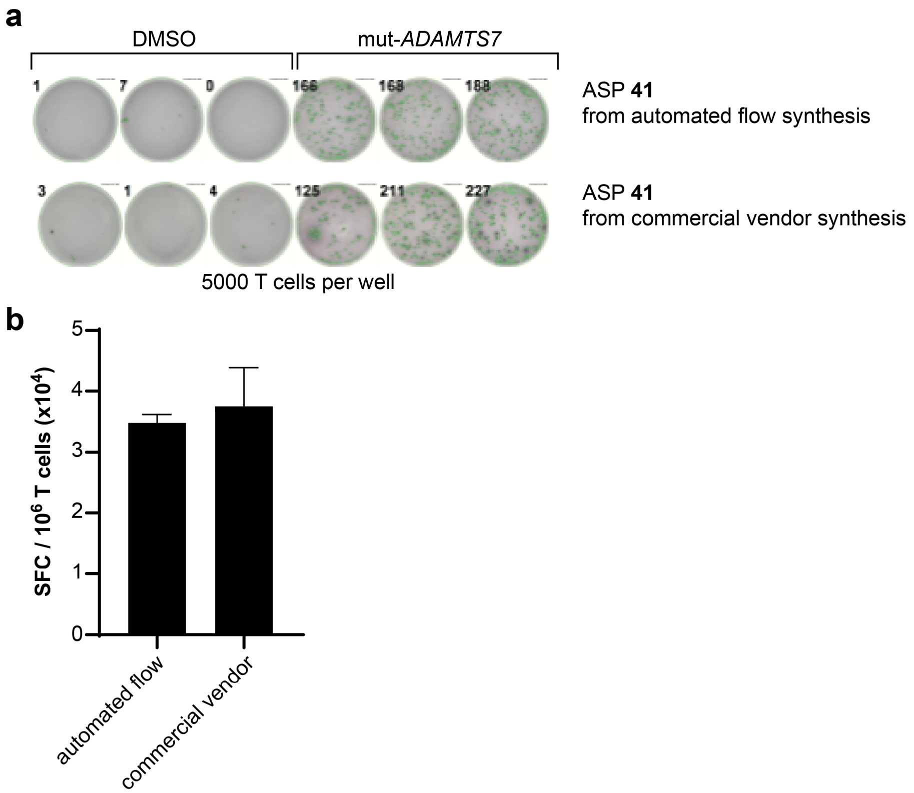

Supplementary Figure S7. IFN-γ secretion by neoantigen-specific T cell lines against mutated *ADAMTS7* (ASP 41) peptide. (a) The PBMCs were cultured with 2 µg/ml mut-*ADAMTS7* peptide for 14 days. 5×10^3 T cells were co-cultured with 1×10^4 antigen presenting cells with DMSO or mut-*ADAMTS7* peptide (10 µg/ml in ELISPOT wells overnight followed by IFN-γ ELISPOT assay. (b) Graph of the enumerated spot forming cells (SFCs) per 1 million cells. A two-sample two-tailed *t*-test with Welch correction indicated no statistical difference ($P > 0.05$).

Characterization Data for IMP **1**

UV absorbance trace from flow peptide synthesis

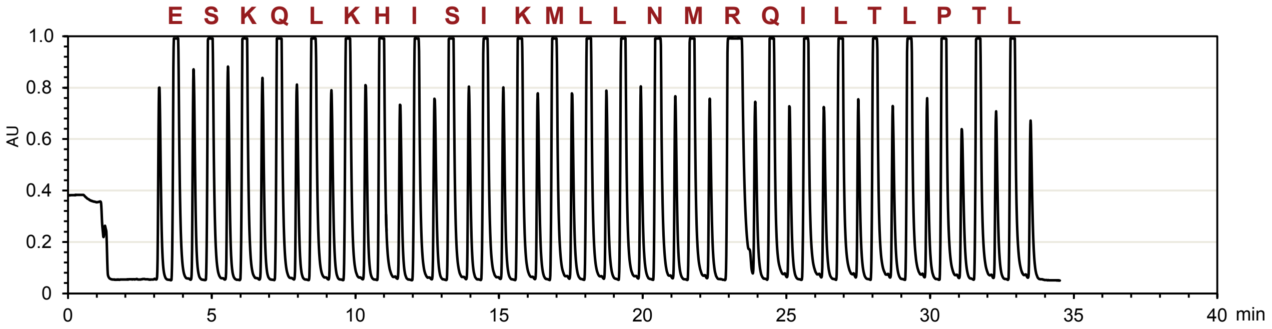

RP-HPLC trace of the unpurified peptide

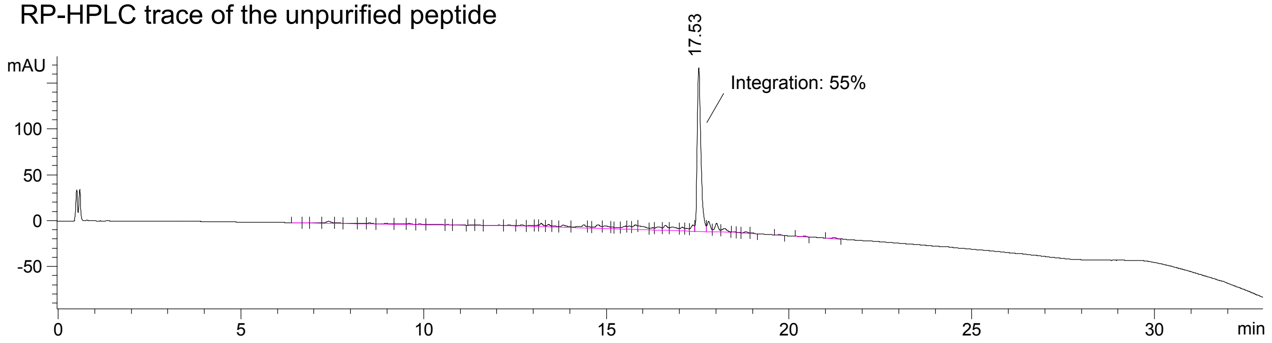

RP-HPLC trace of the purified peptide

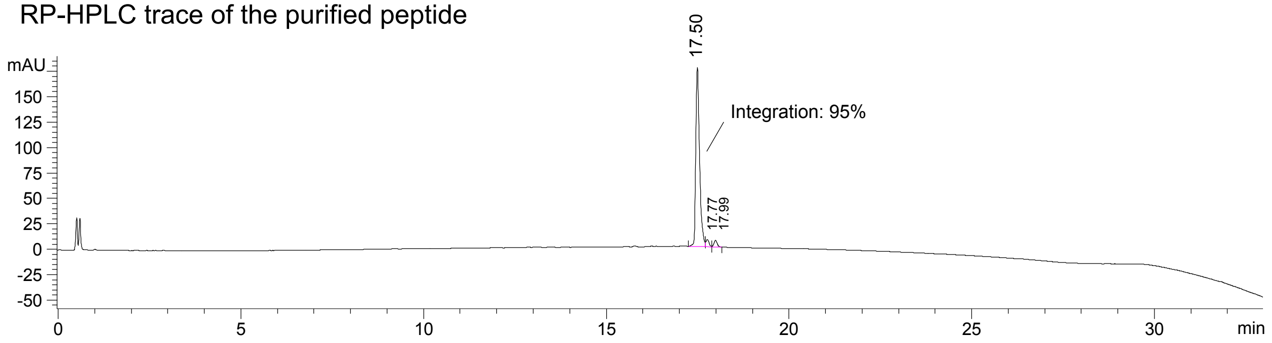

mass spectrum (ESI) of the purified peptide

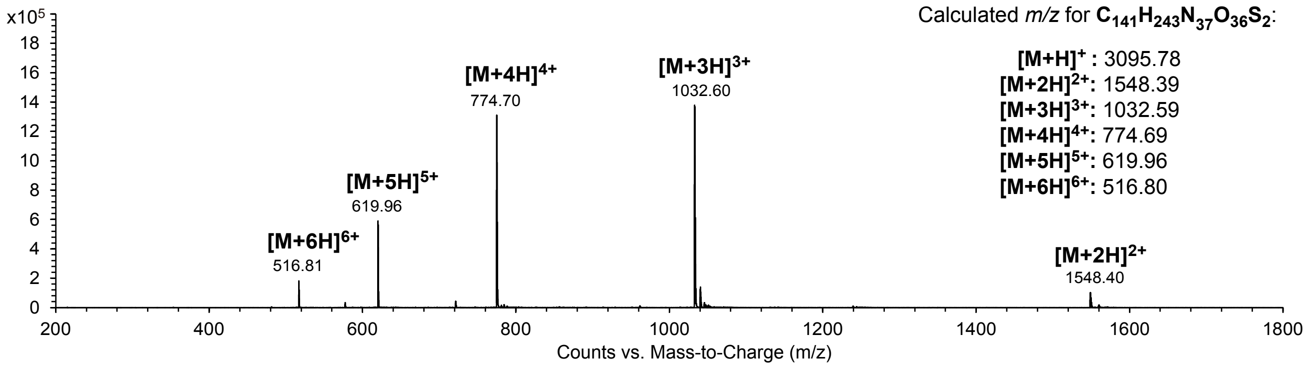

Characterization Data for IMP **2**

UV absorbance trace from flow peptide synthesis

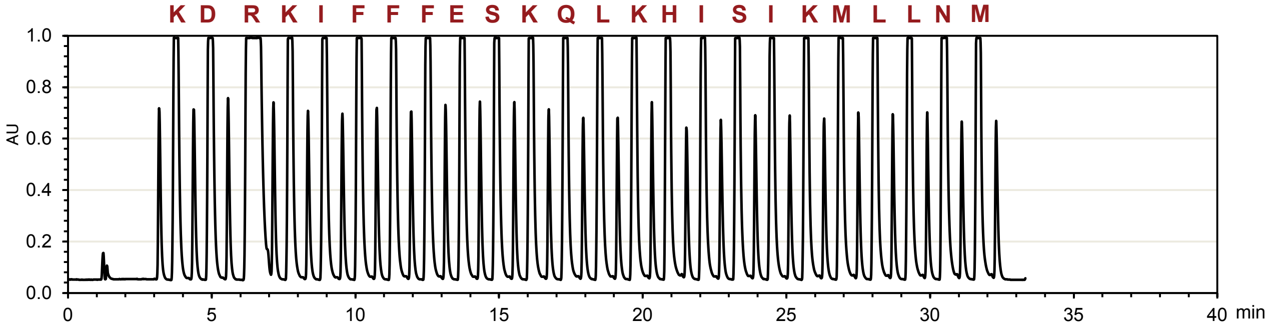

RP-HPLC trace of the unpurified peptide

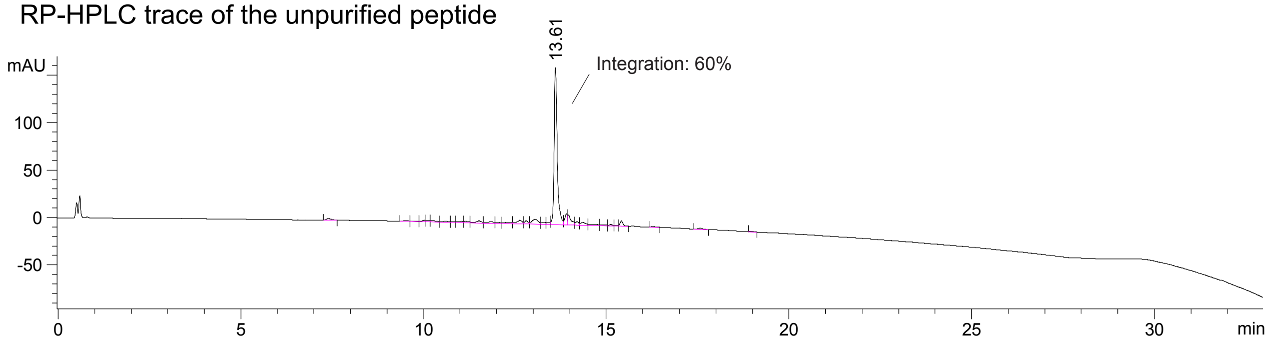

RP-HPLC trace of the purified peptide

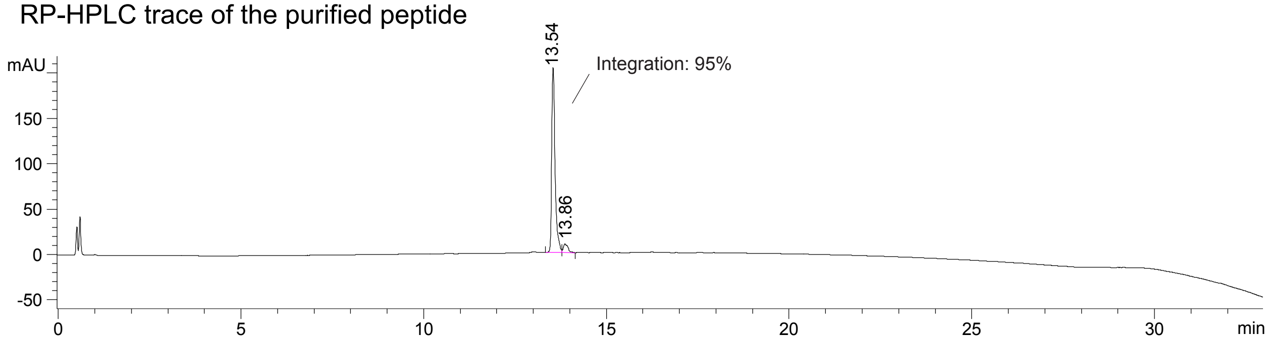

mass spectrum (ESI) of the purified peptide

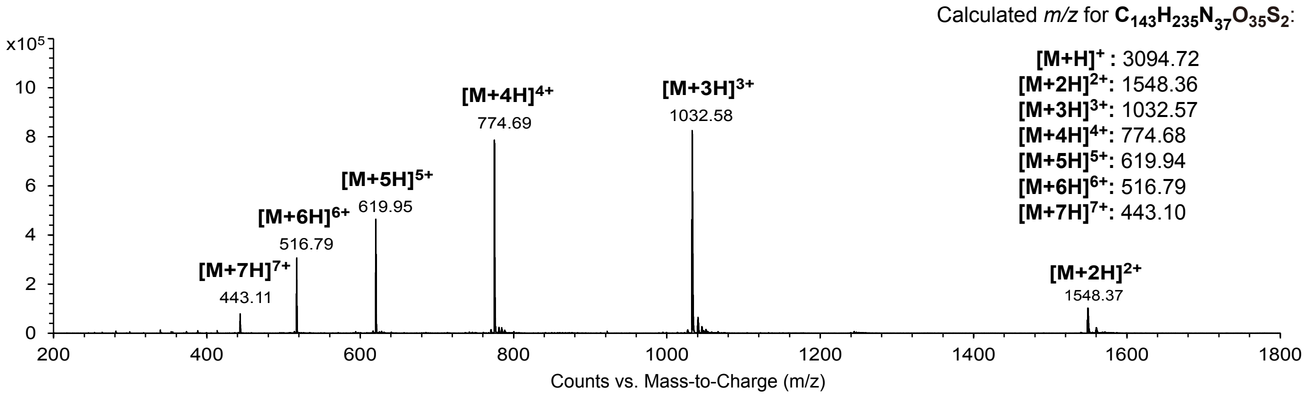

Characterization Data for IMP **3**

UV absorbance trace from flow peptide synthesis

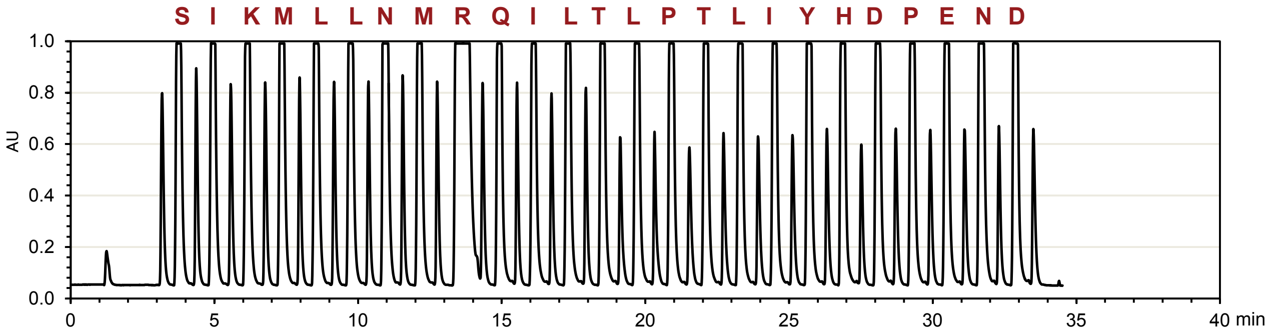

RP-HPLC trace of the unpurified peptide

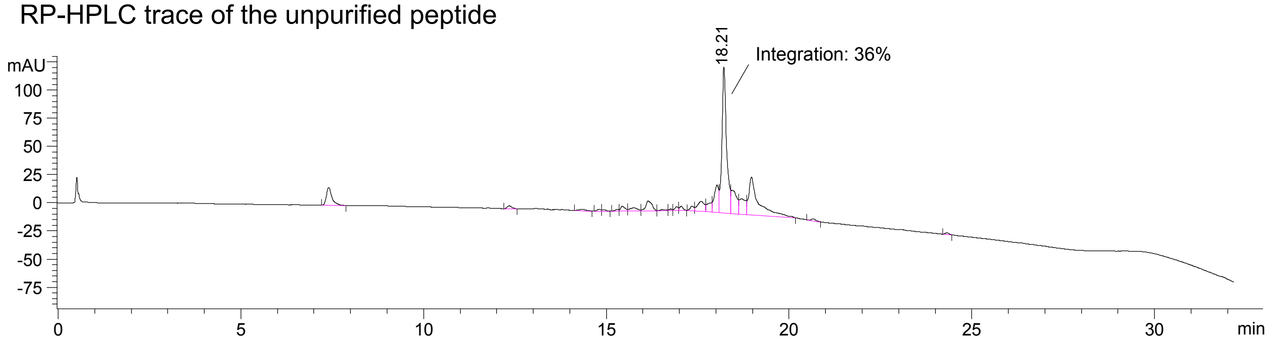

RP-HPLC trace of the purified peptide

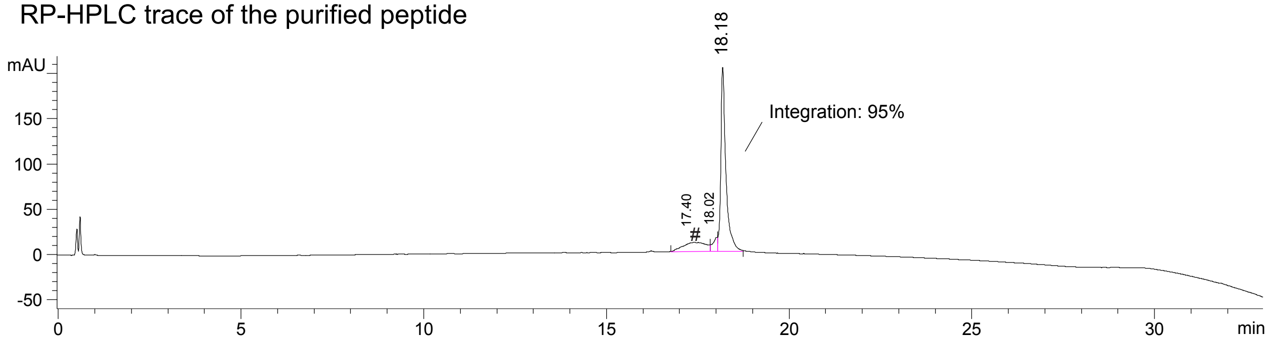

The number sign (#) indicates an uncharacterized oligomer of this peptide that forms.

mass spectrum (ESI) of the purified peptide

Calculated *m/z* for $C_{138}H_{229}N_{35}O_{40}S_2$:

$[M+H]^+$: 3080.64
$[M+2H]^{2+}$: 15441.32
$[M+3H]^{3+}$: 1027.88
$[M+4H]^{4+}$: 771.16

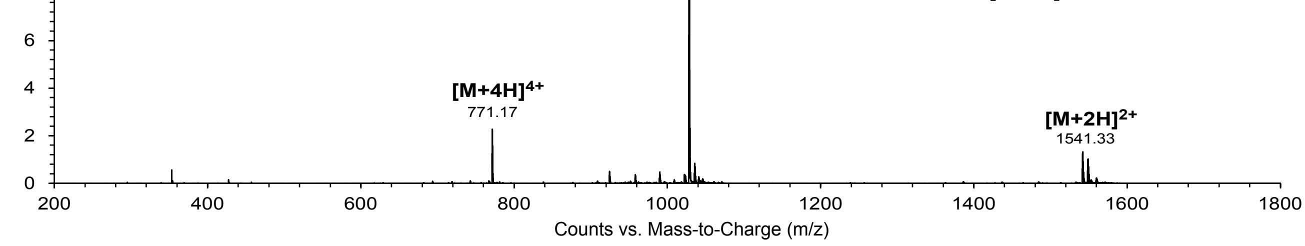

Characterization Data for IMP **4**

UV absorbance trace from flow peptide synthesis

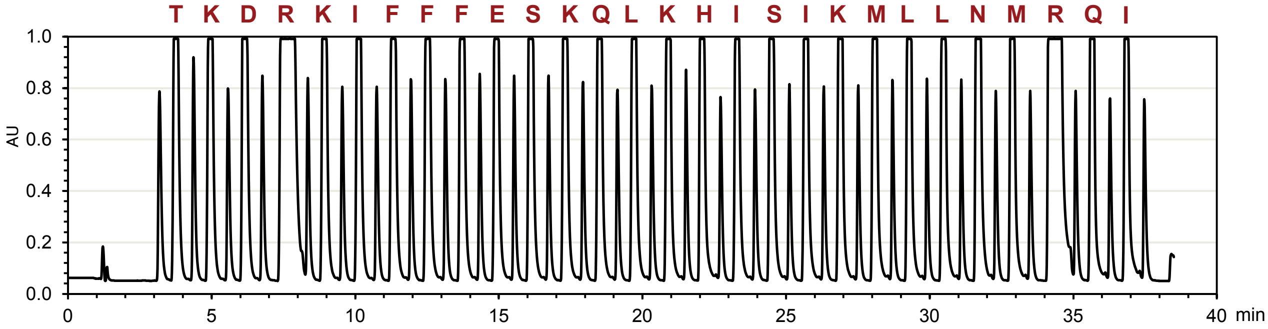

RP-HPLC trace of the unpurified peptide

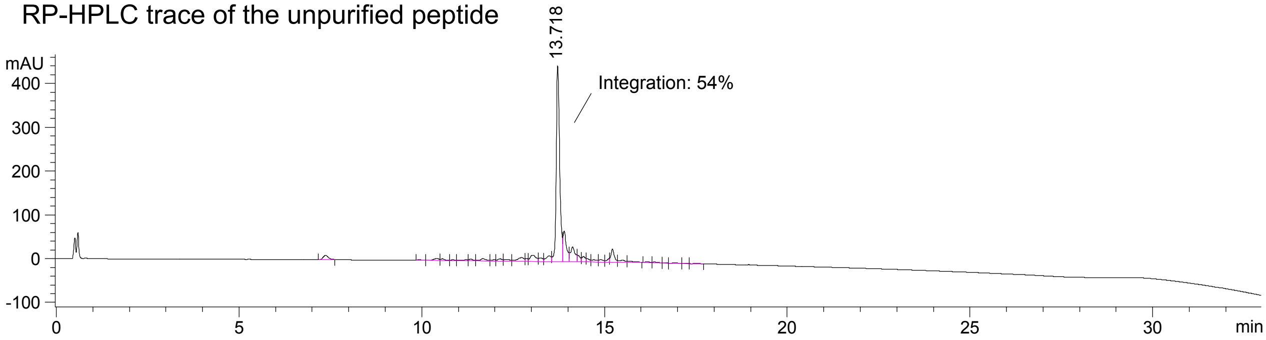

RP-HPLC trace of the purified peptide

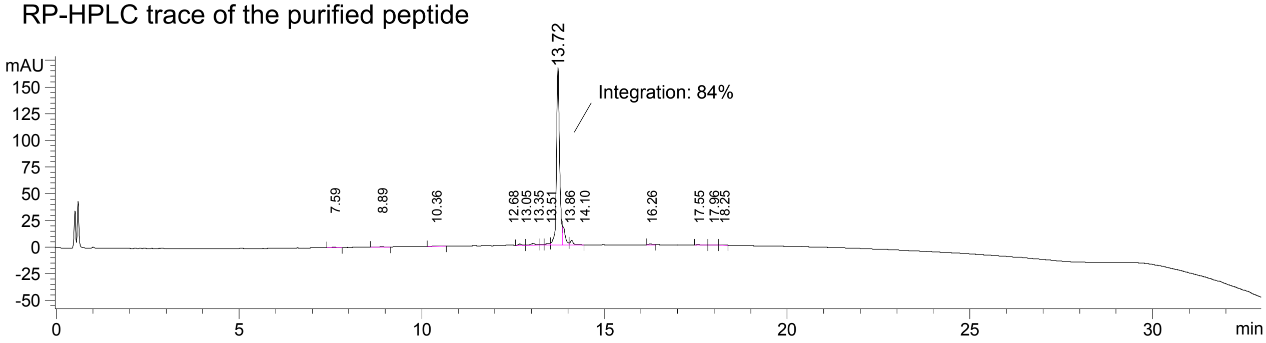

mass spectrum (ESI) of the purified peptide

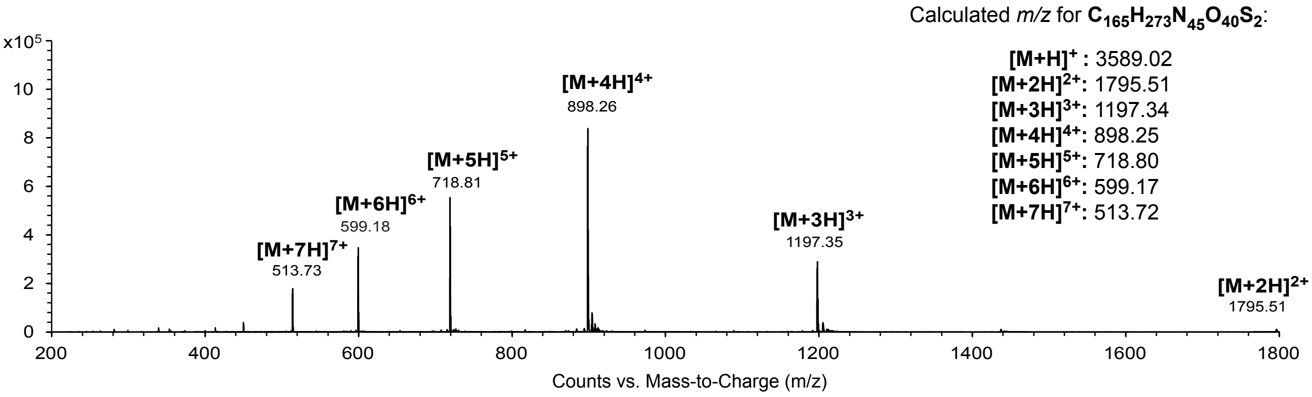

Characterization Data for IMP **5**

UV absorbance trace from flow peptide synthesis

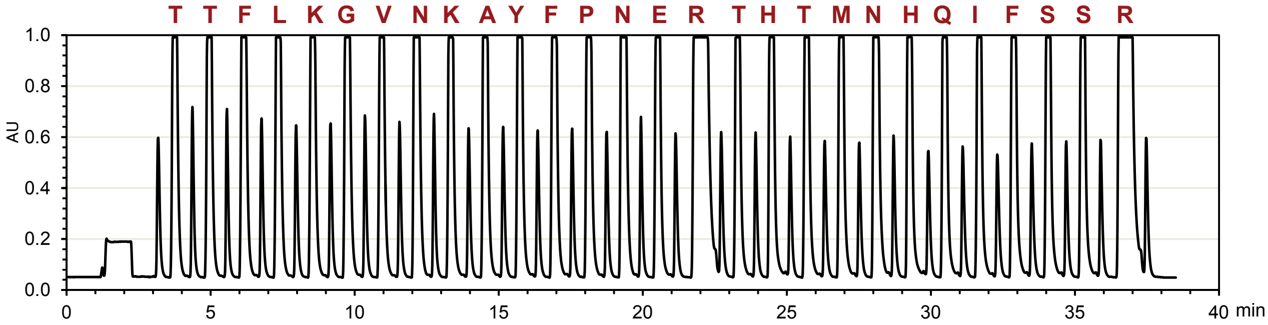

RP-HPLC trace of the unpurified peptide

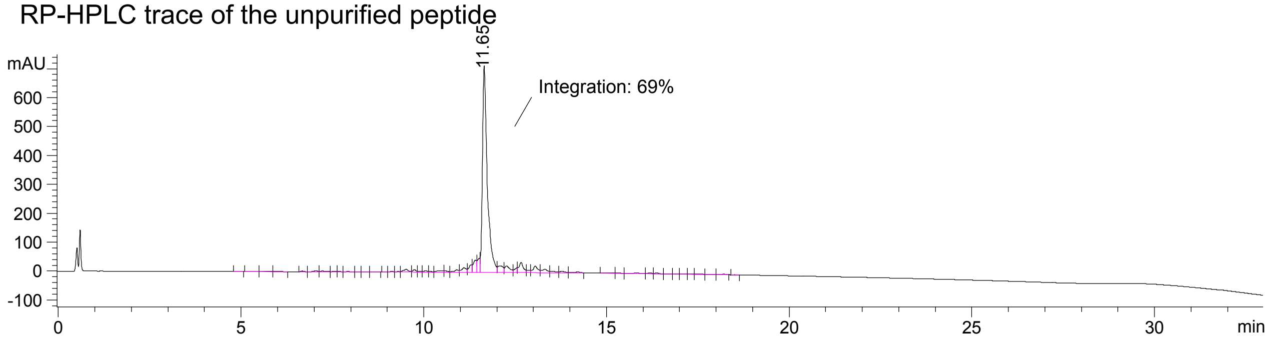

RP-HPLC trace of the purified peptide

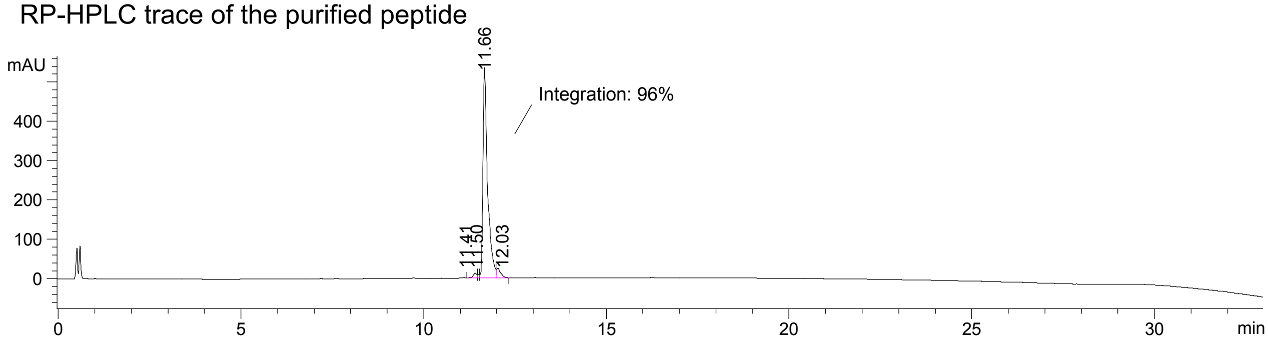

mass spectrum (ESI) of the purified peptide

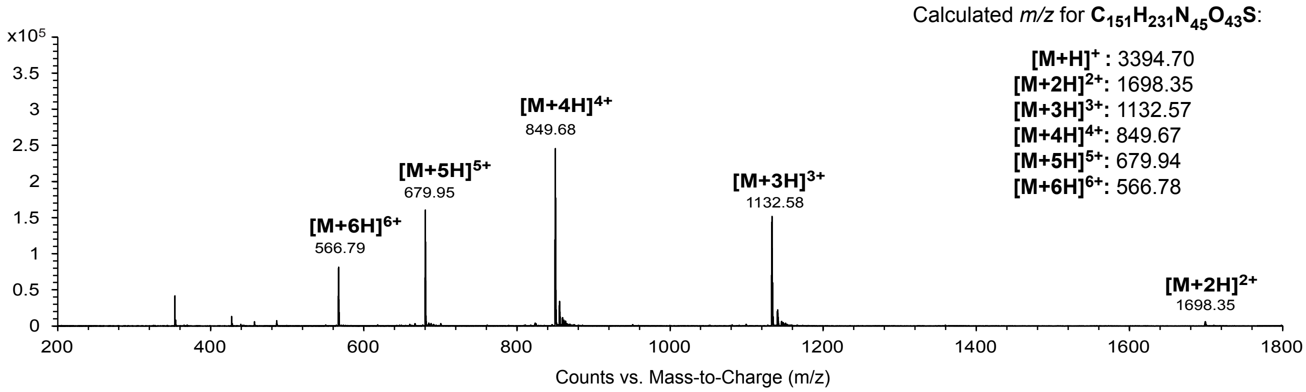

Characterization Data for IMP **6**

UV absorbance trace from flow peptide synthesis

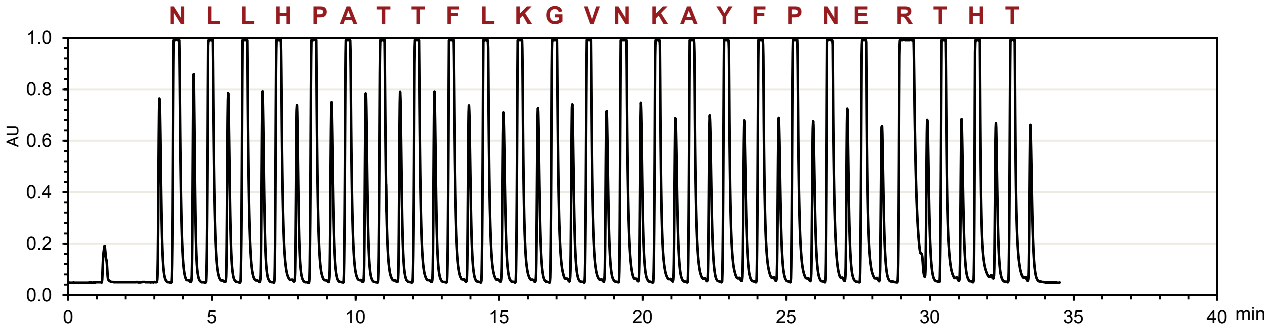

RP-HPLC trace of the unpurified peptide

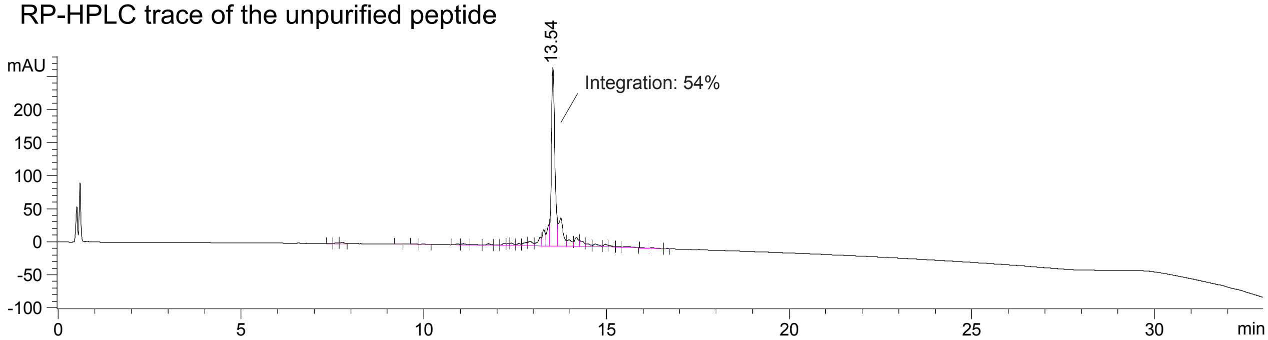

RP-HPLC trace of the purified peptide

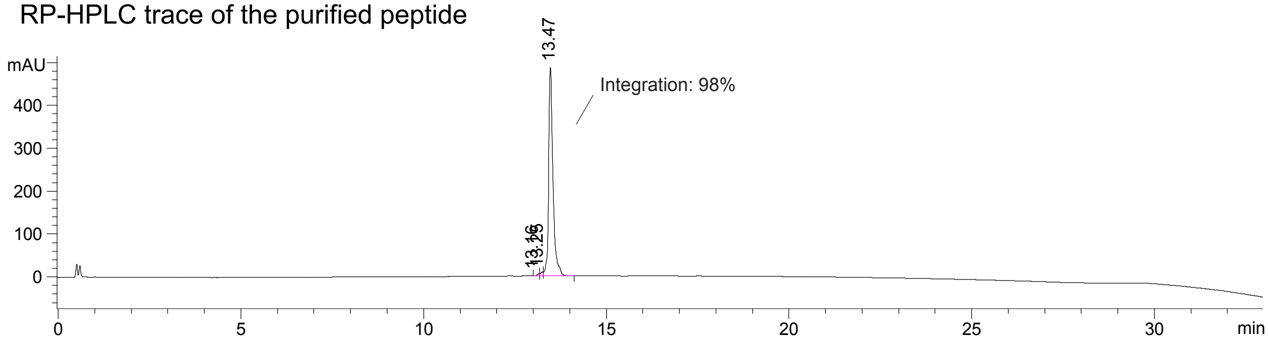

mass spectrum (ESI) of the purified peptide

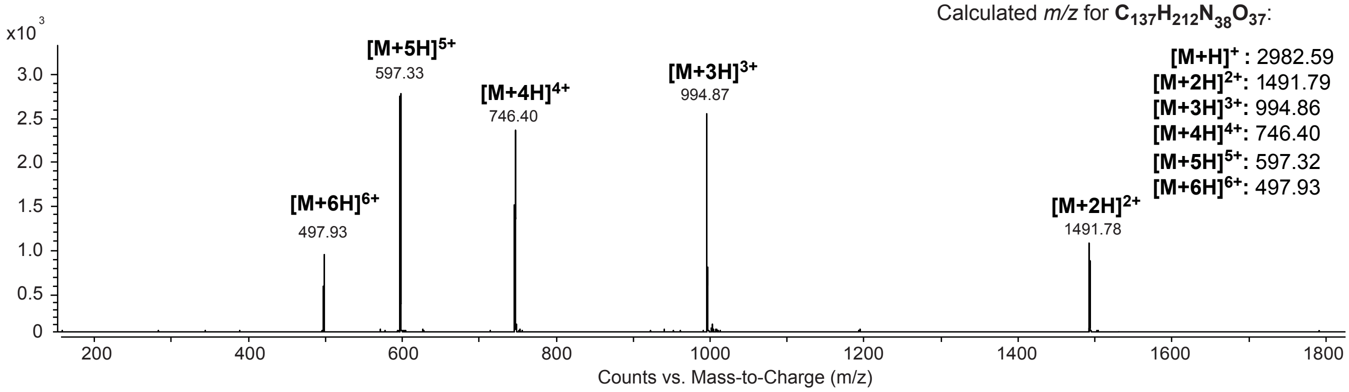

Characterization Data for IMP **7**

UV absorbance trace from flow peptide synthesis

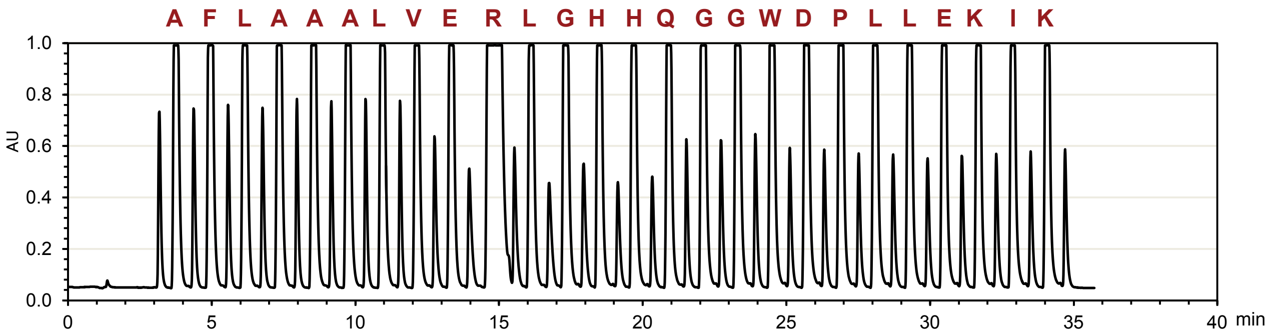

RP-HPLC trace of the unpurified peptide

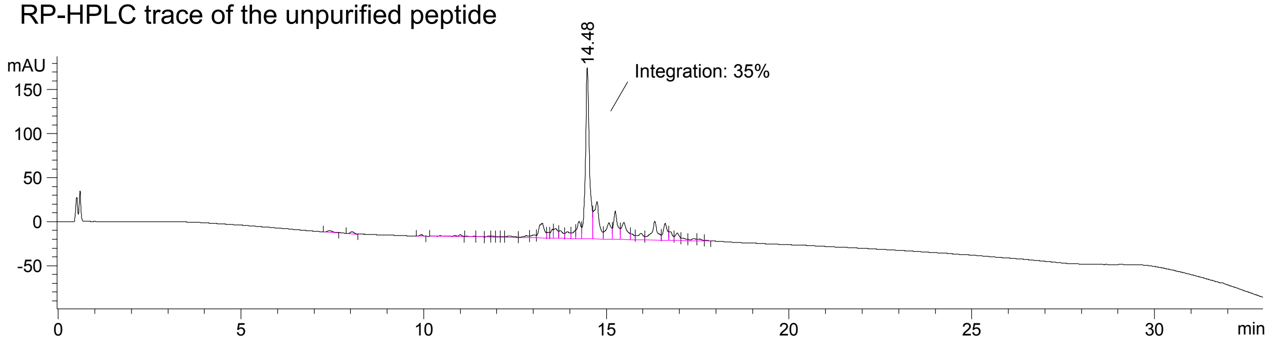

RP-HPLC trace of the purified peptide

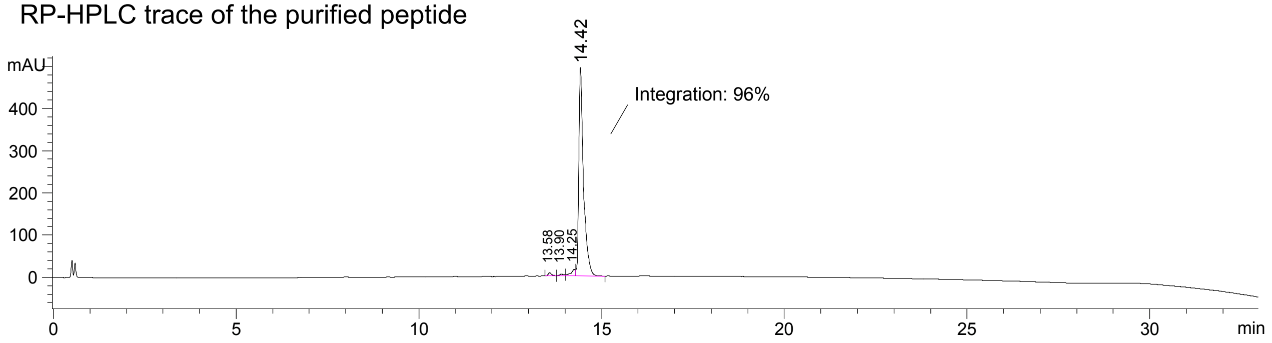

mass spectrum (ESI) of the purified peptide

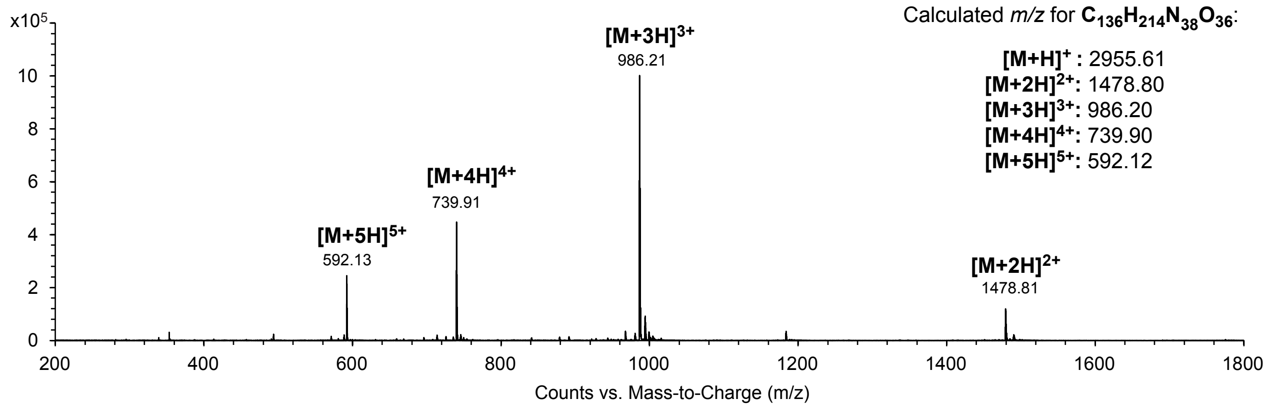

Characterization Data for IMP **8**

UV absorbance trace from flow peptide synthesis

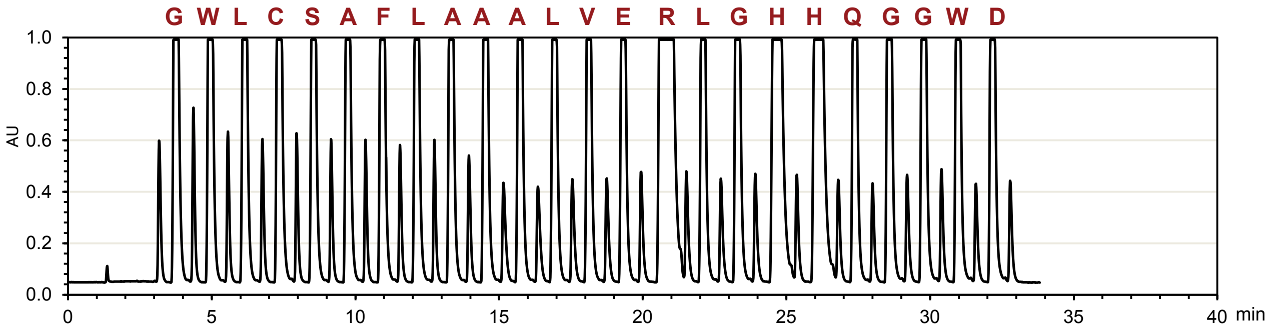

RP-HPLC trace of the unpurified peptide

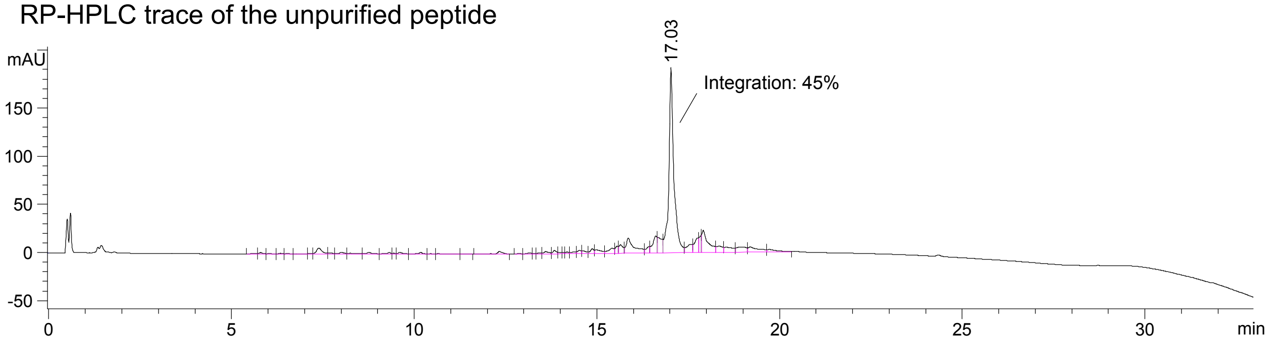

RP-HPLC trace of the purified peptide

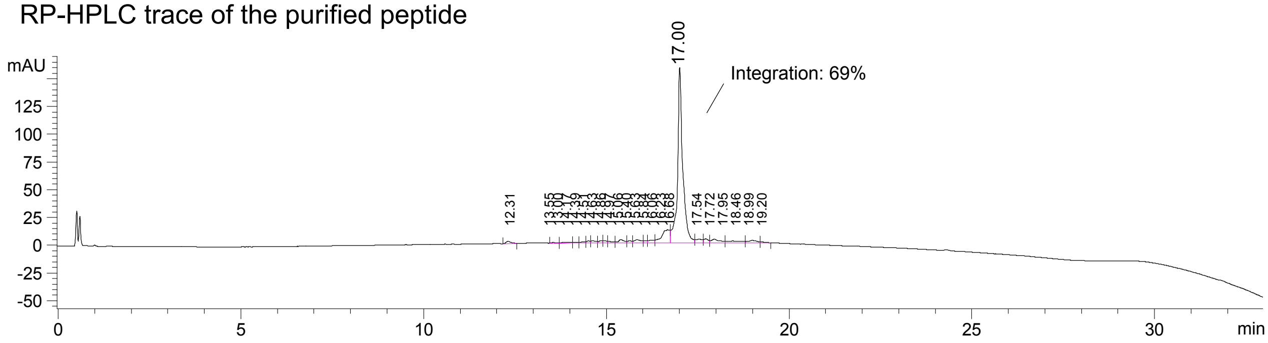

mass spectrum (ESI) of the purified peptide

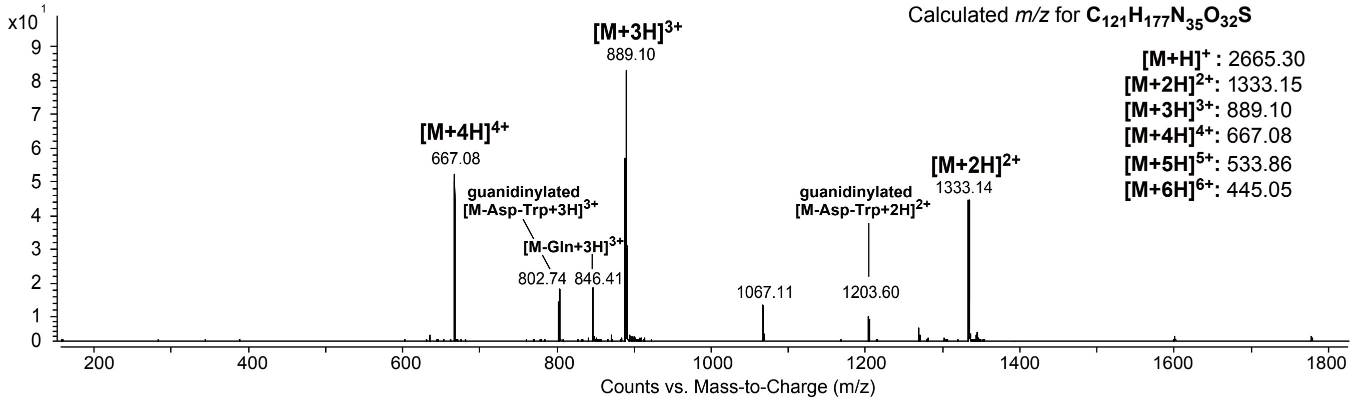

Characterization Data for IMP **9**

UV absorbance trace from flow peptide synthesis

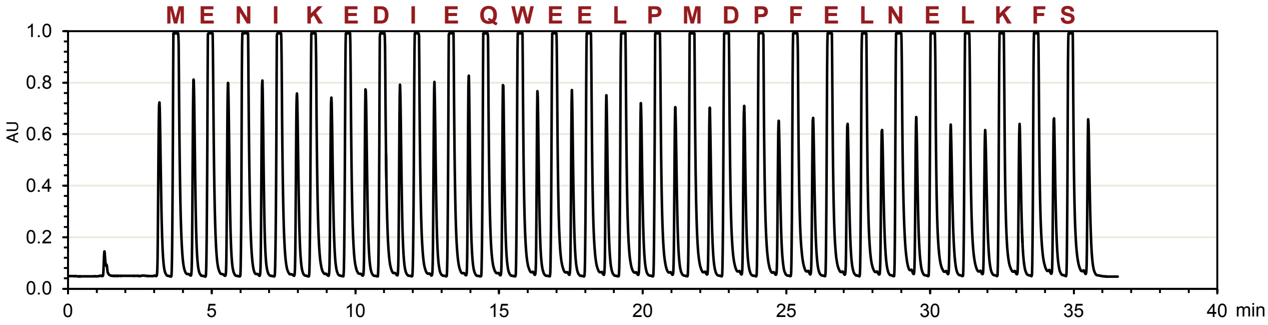

RP-HPLC trace of the unpurified peptide

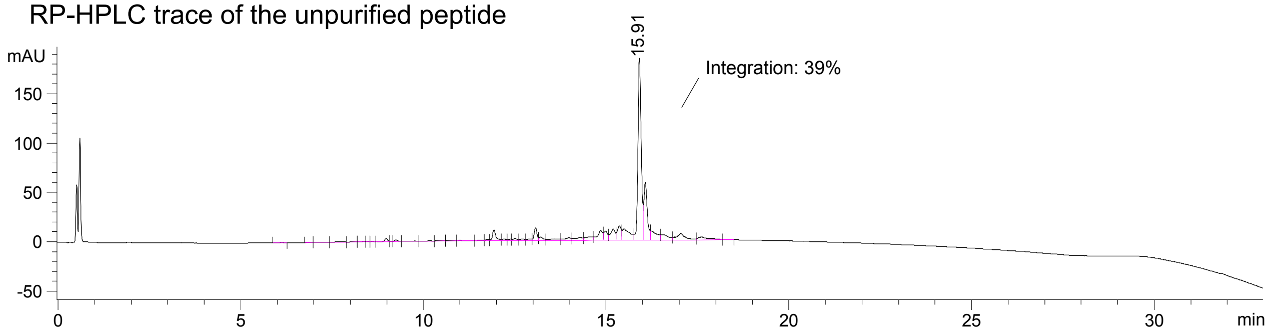

RP-HPLC trace of the purified peptide

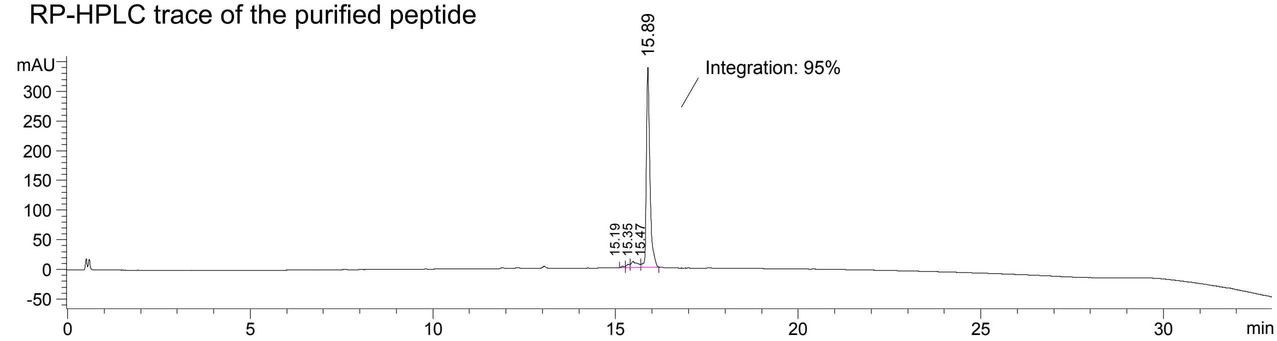

mass spectrum (ESI) of the purified peptide

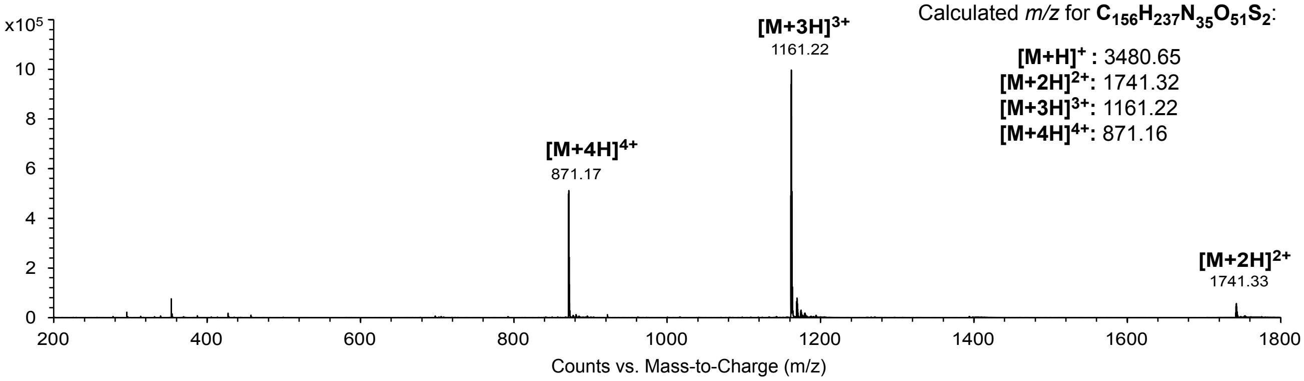

Calculated *m/z* for $C_{156}H_{237}N_{35}O_{51}S_2$:

$[M+H]^+$: 3480.65
$[M+2H]^{2+}$: 1741.32
$[M+3H]^{3+}$: 1161.22
$[M+4H]^{4+}$: 871.16

Characterization Data for IMP **10**

UV absorbance trace from flow peptide synthesis

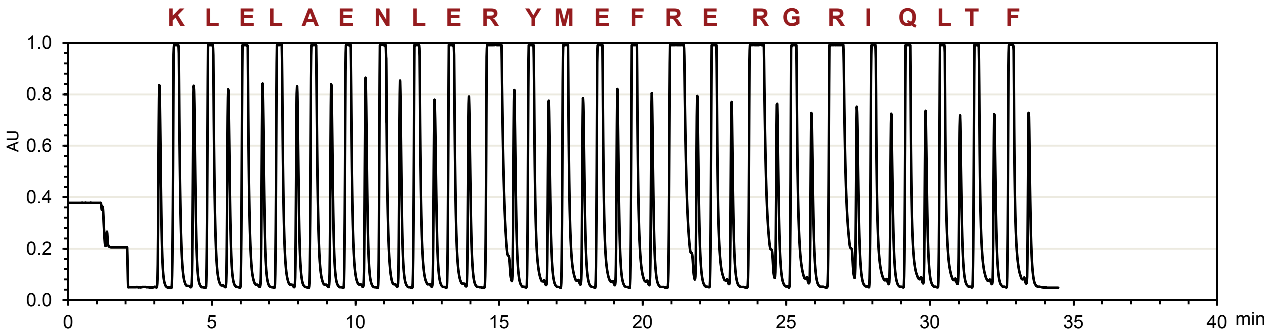

RP-HPLC trace of the unpurified peptide

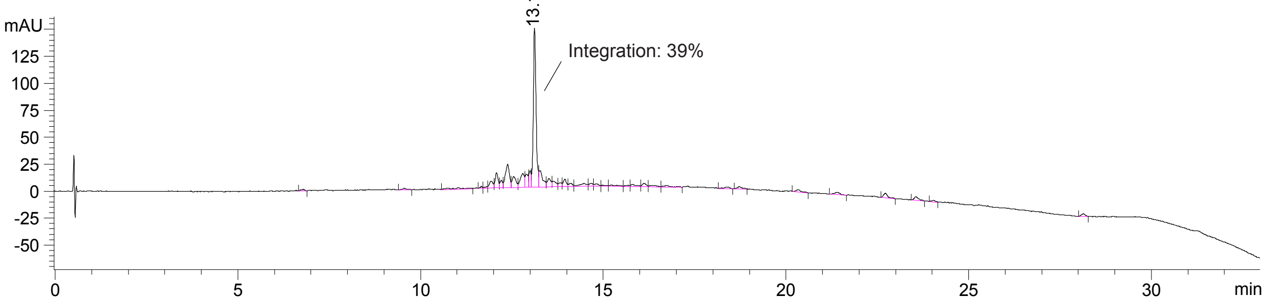

RP-HPLC trace of the purified peptide

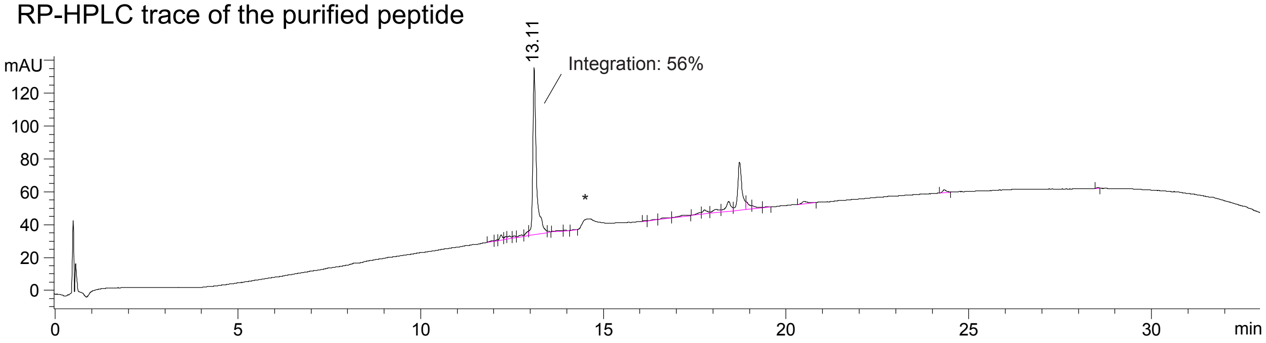

The asterisk (*) indicates an impurity from the HPLC column.

mass spectrum (ESI) of the purified peptide

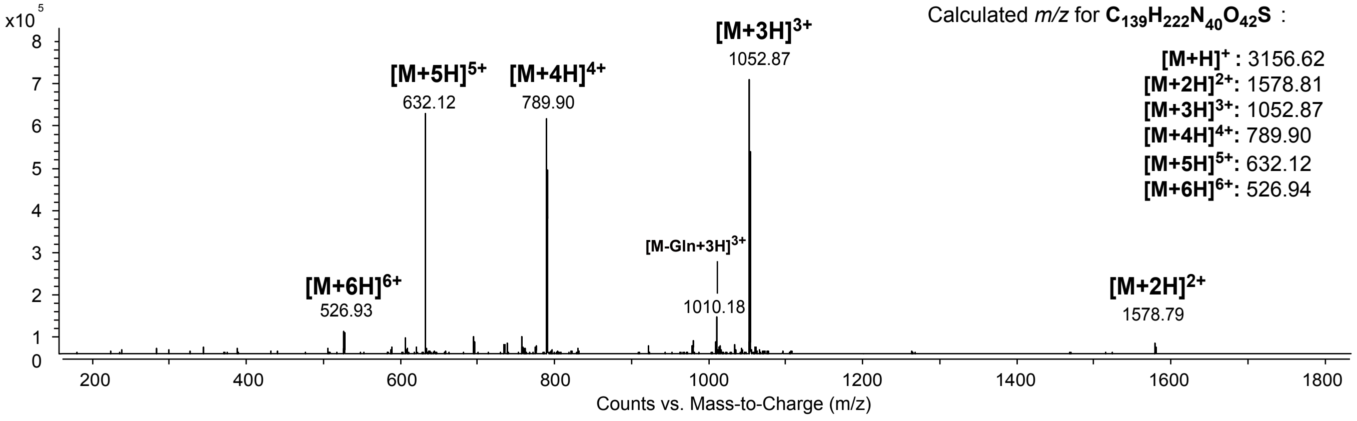

Characterization Data for IMP **11**

UV absorbance trace from flow peptide synthesis

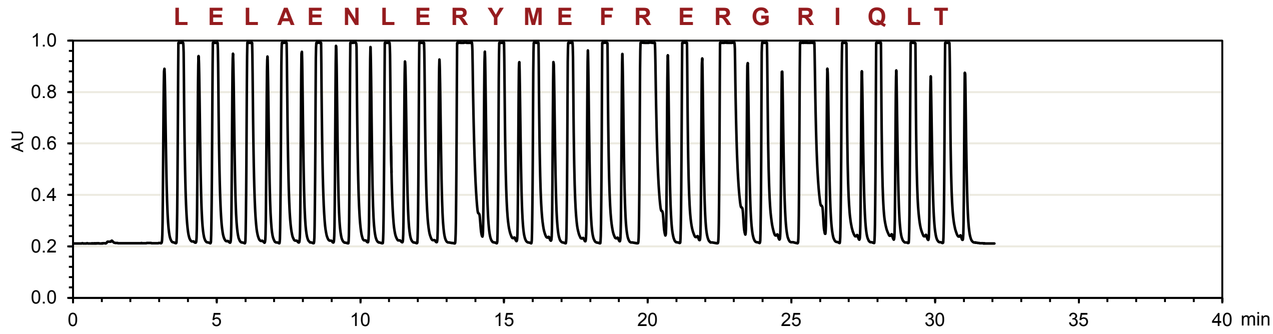

RP-HPLC trace of the unpurified peptide

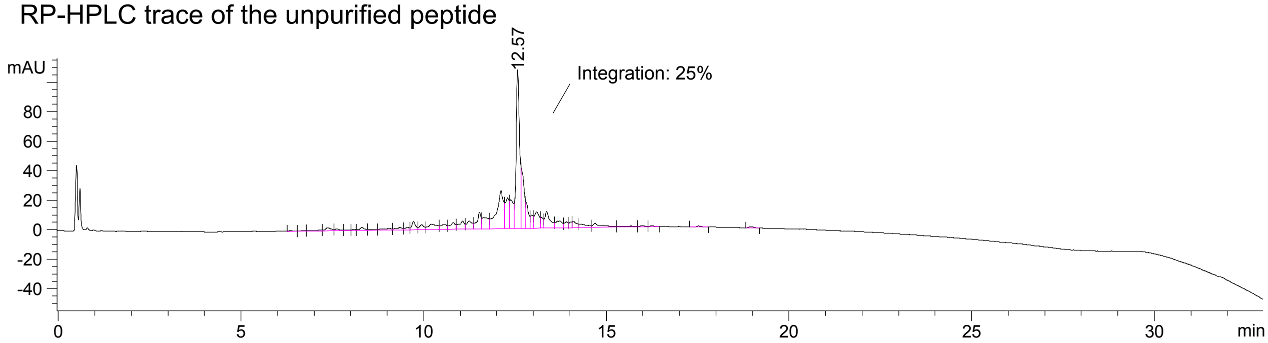

RP-HPLC trace of the purified peptide

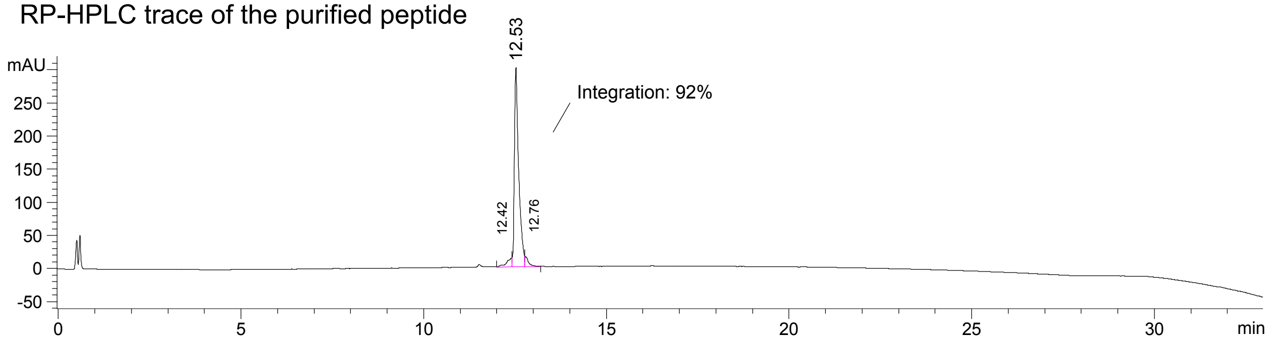

mass spectrum (ESI) of the purified peptide

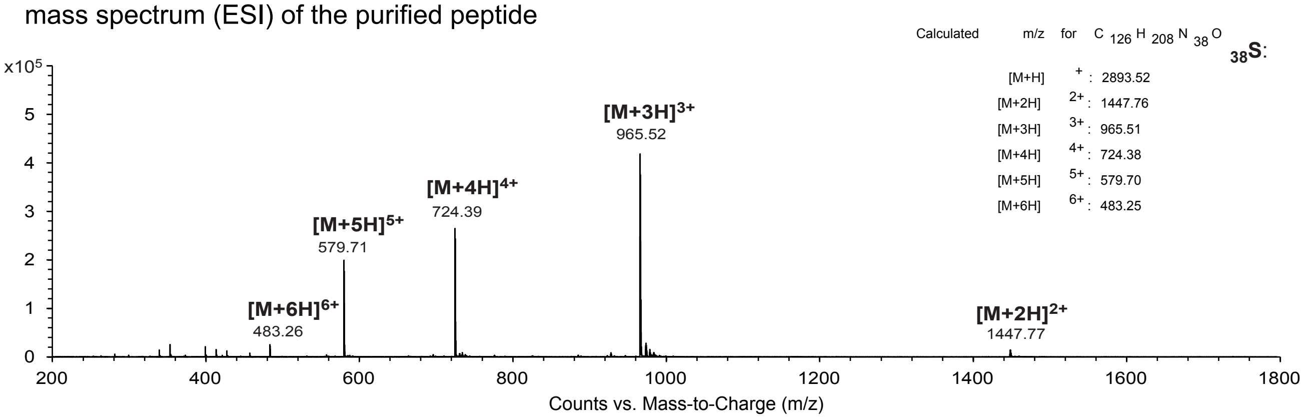

Characterization Data for IMP **12**

UV absorbance trace from flow peptide synthesis

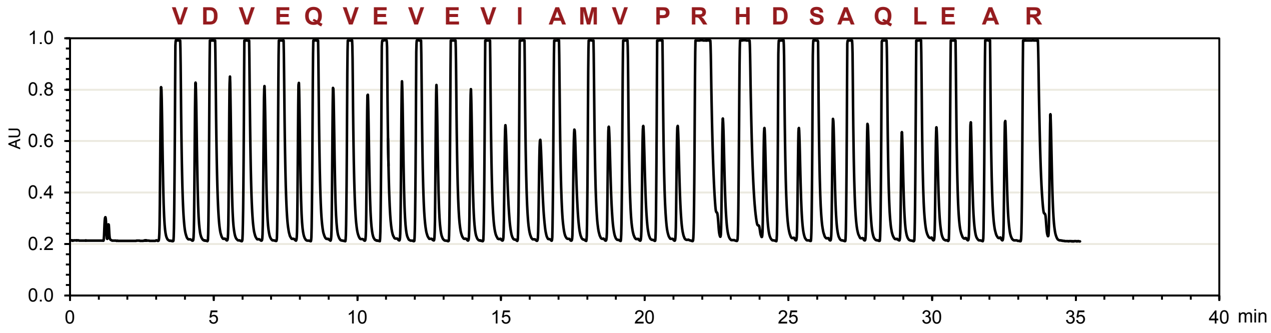

RP-HPLC trace of the unpurified peptide

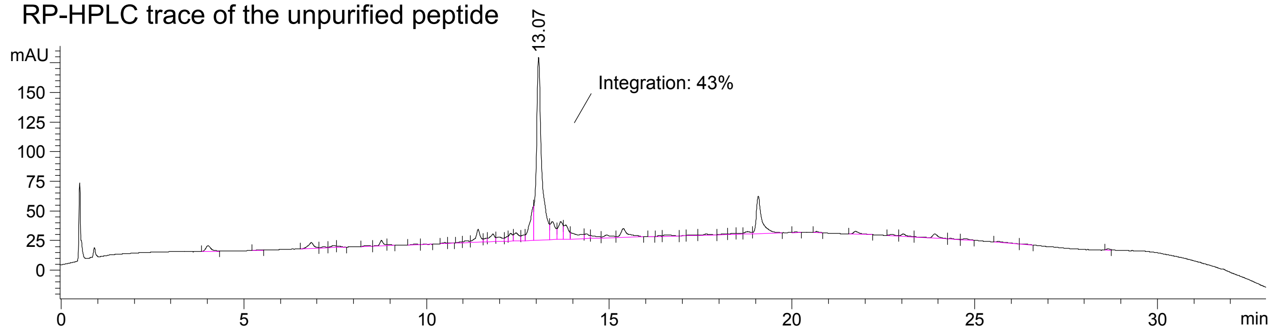

RP-HPLC trace of the purified peptide

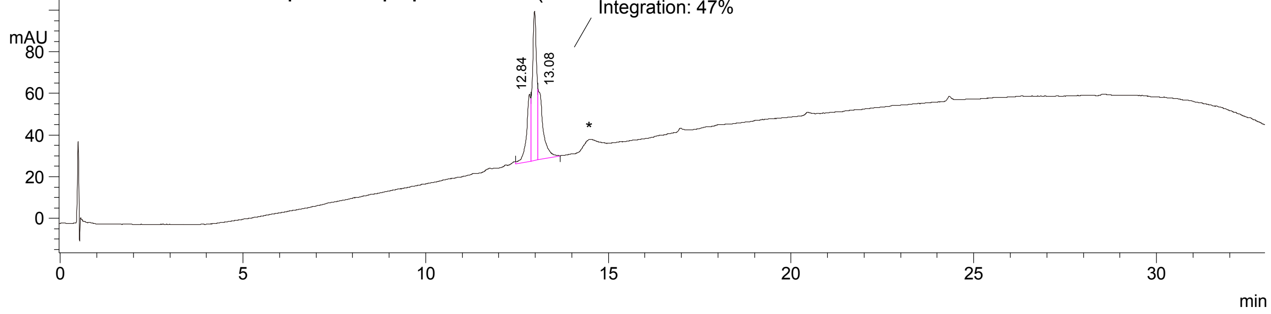

The asterisk (*) indicates an impurity from the HPLC column.

mass spectrum (ESI) of the purified peptide

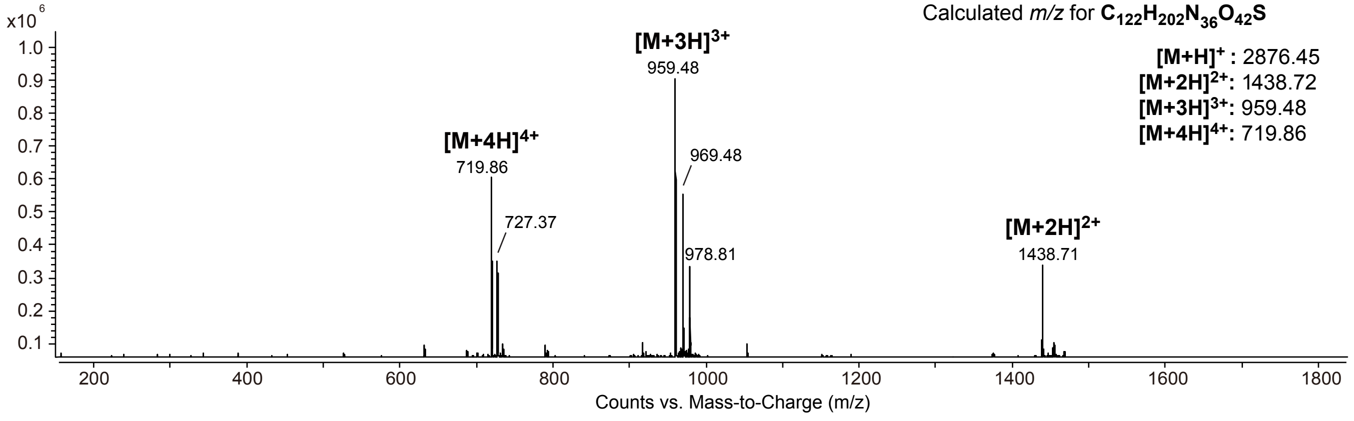

Characterization Data for IMP **13**

UV absorbance trace from flow peptide synthesis

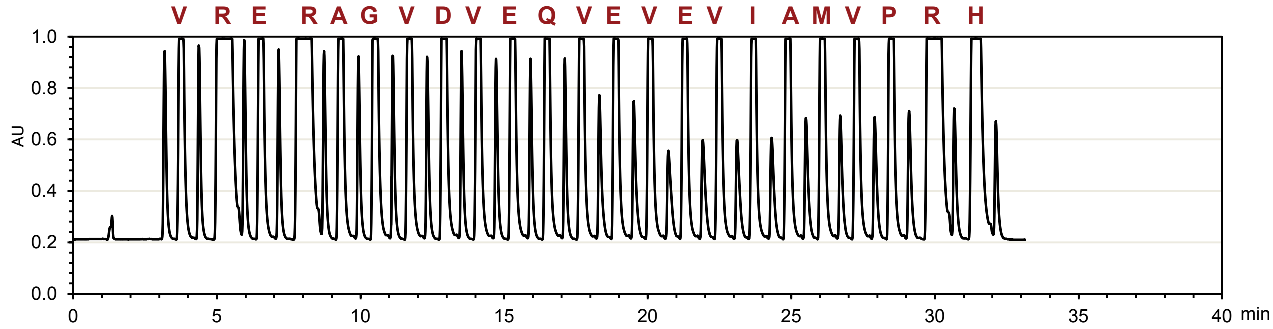

RP-HPLC trace of the unpurified pept

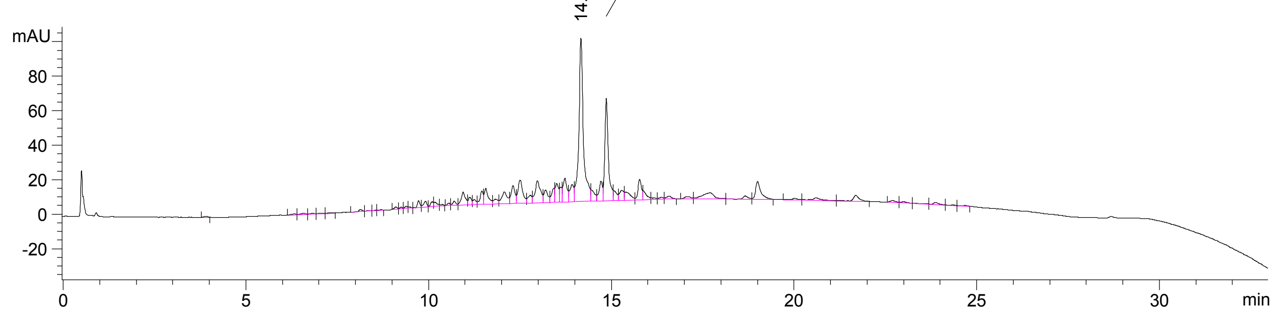

RP-HPLC trace of the purified peptide

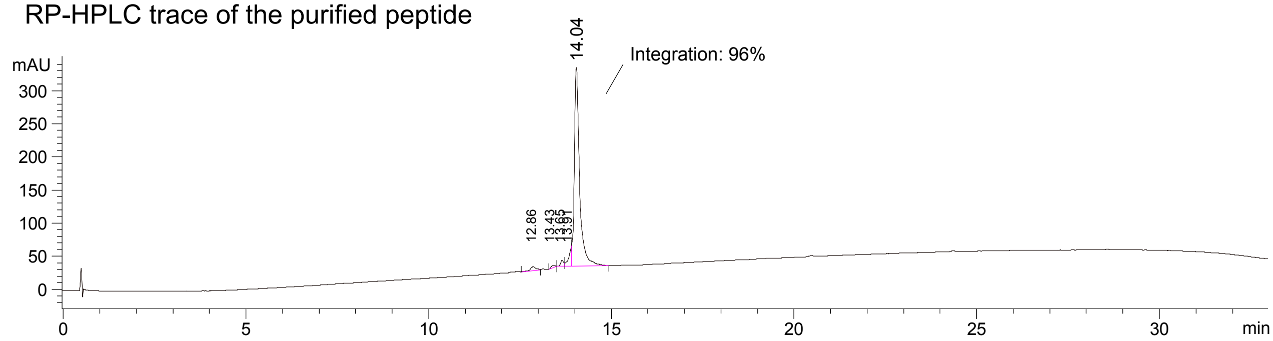

mass spectrum (ESI) of the purified peptide

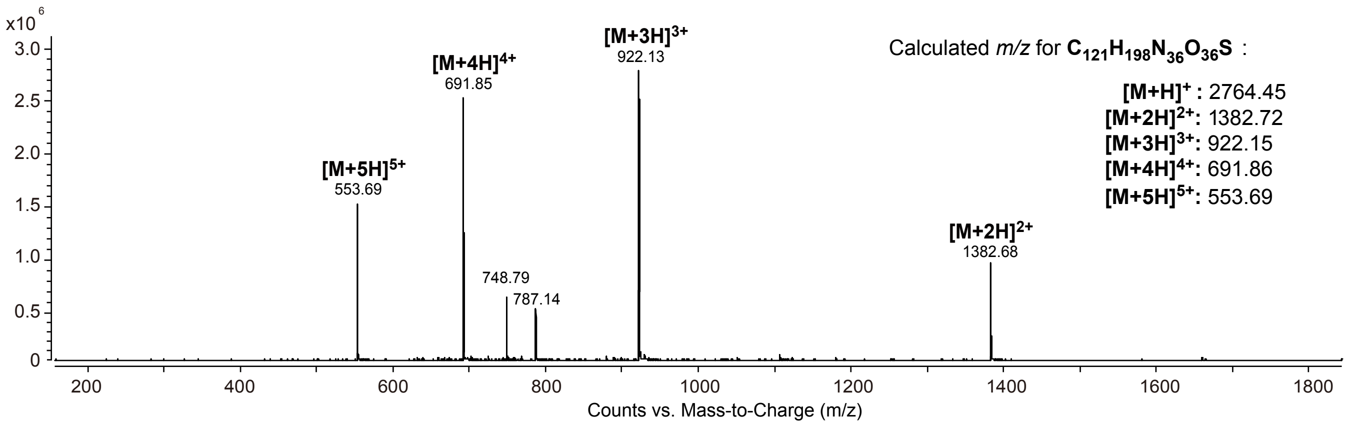

Characterization Data for IMP **14**

UV absorbance trace from flow peptide synthesis

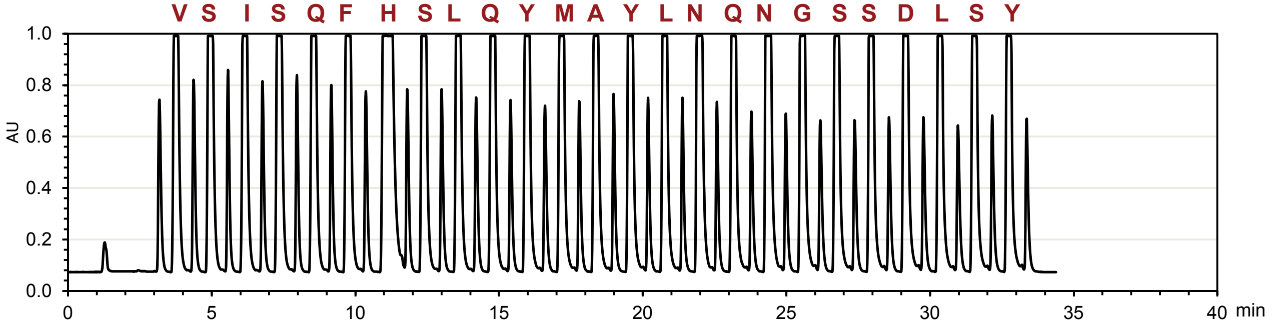

RP-HPLC trace of the unpurified peptide

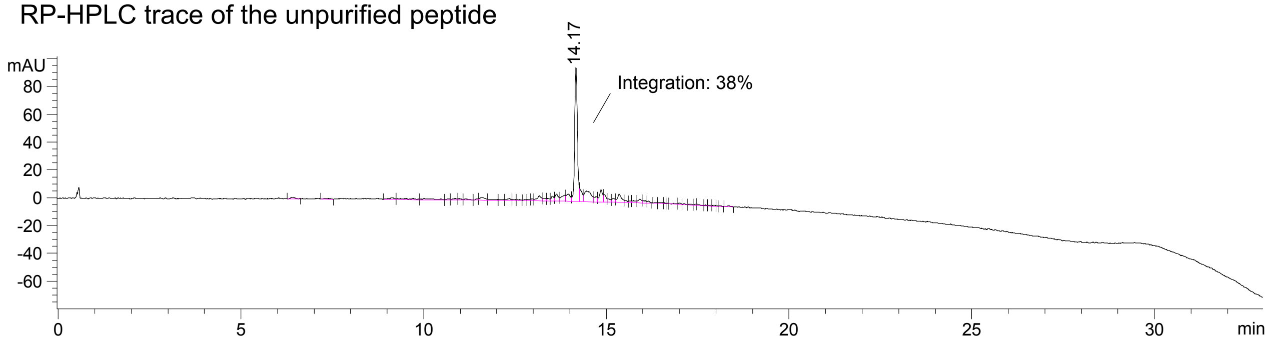

RP-HPLC trace of the purified peptide

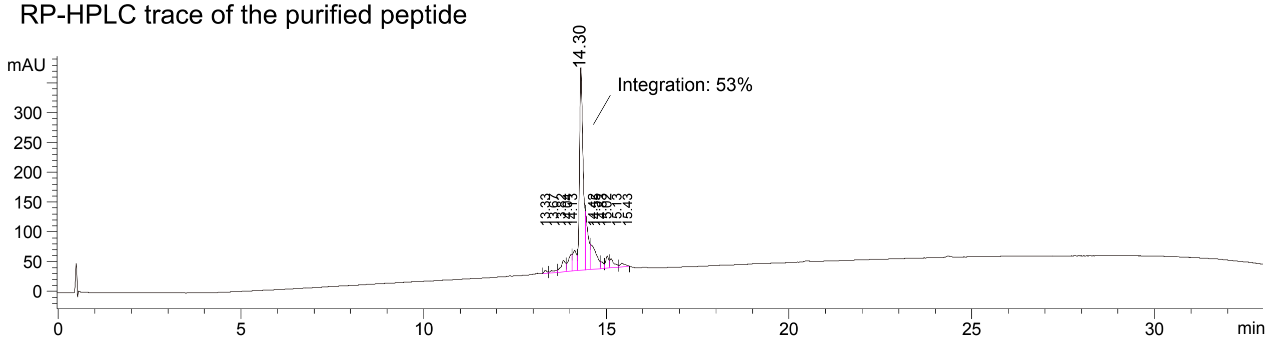

mass spectrum (ESI) of the purified peptide

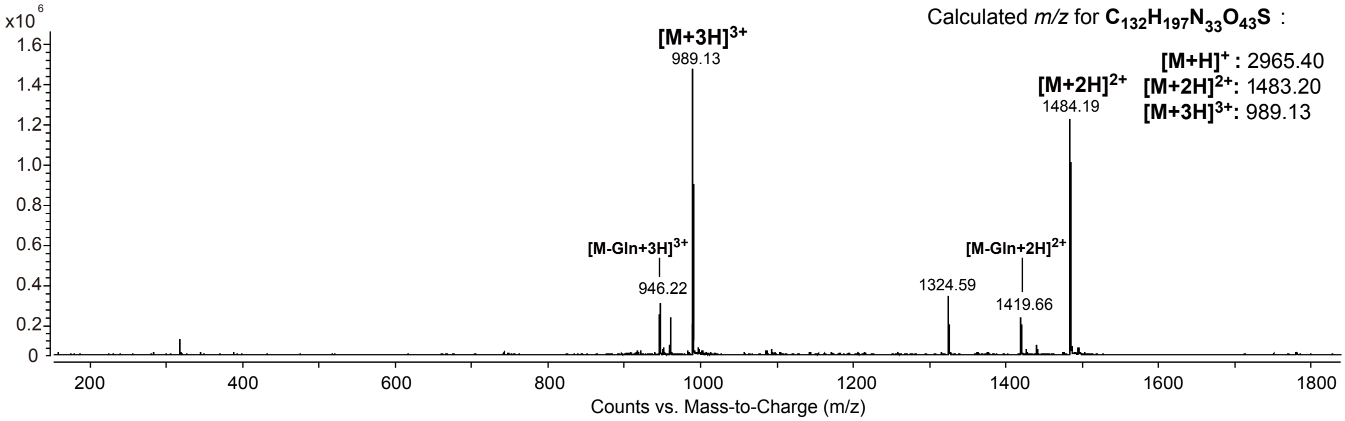

Calculated *m/z* for $C_{132}H_{197}N_{33}O_{43}S$:

$[M+H]^+$: 2965.40
$[M+2H]^{2+}$: 1483.20
$[M+3H]^{3+}$: 989.13

Characterization Data for IMP **15**

UV absorbance trace from flow peptide synthesis

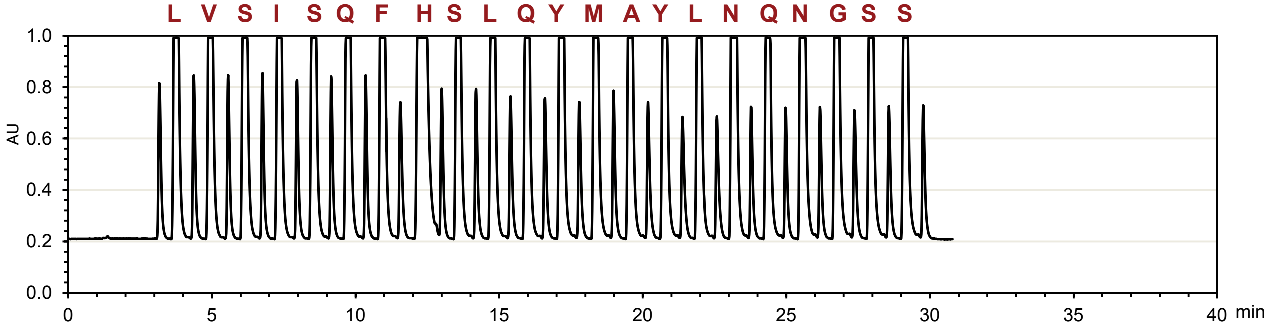

RP-HPLC trace of the unpurified peptide

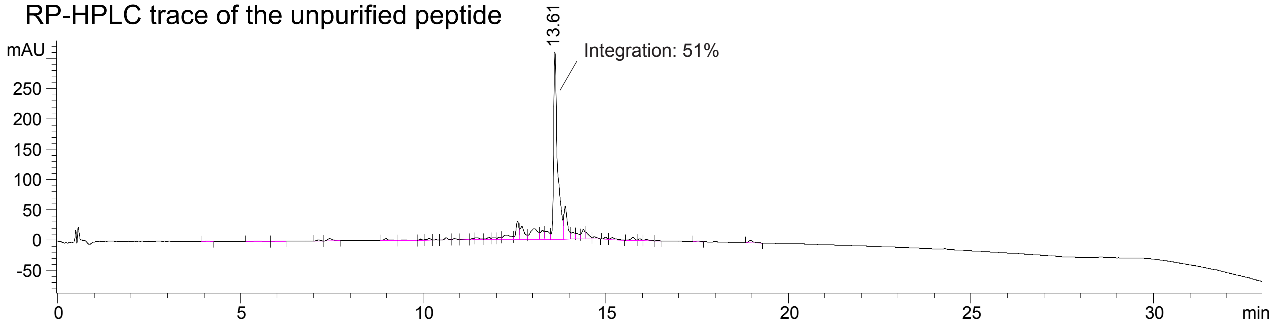

RP-HPLC trace of the purified peptide

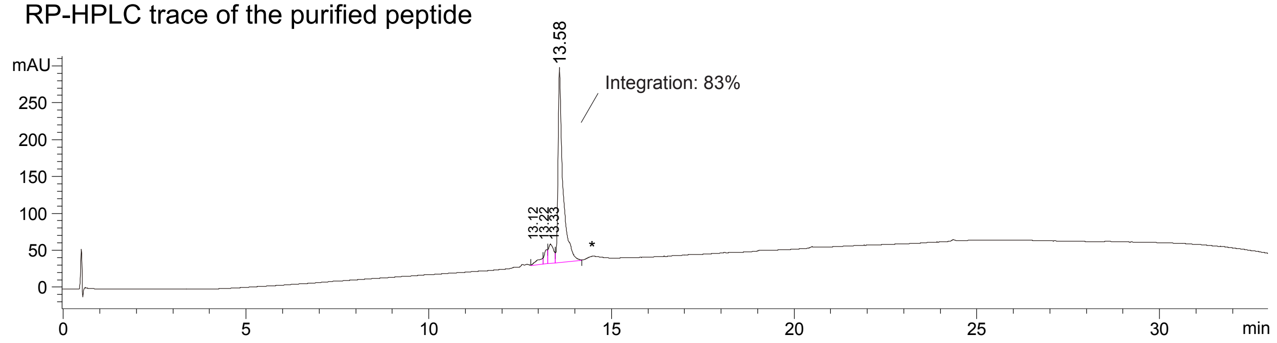

The asterisk (*) indicates an impurity from the HPLC column.

mass spectrum (ESI) of the purified peptide

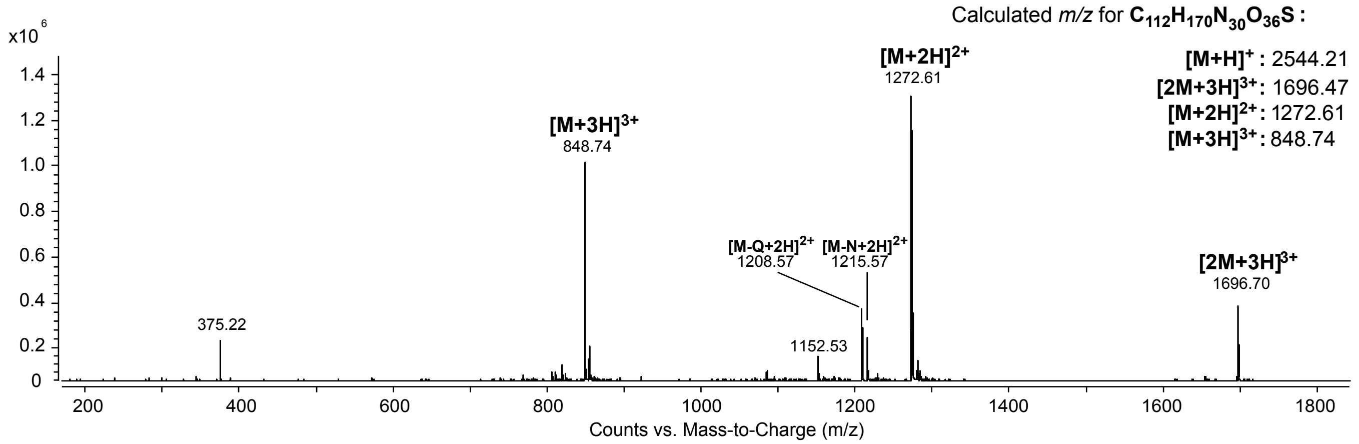

Characterization Data for IMP **16**

UV absorbance trace from flow peptide synthesis

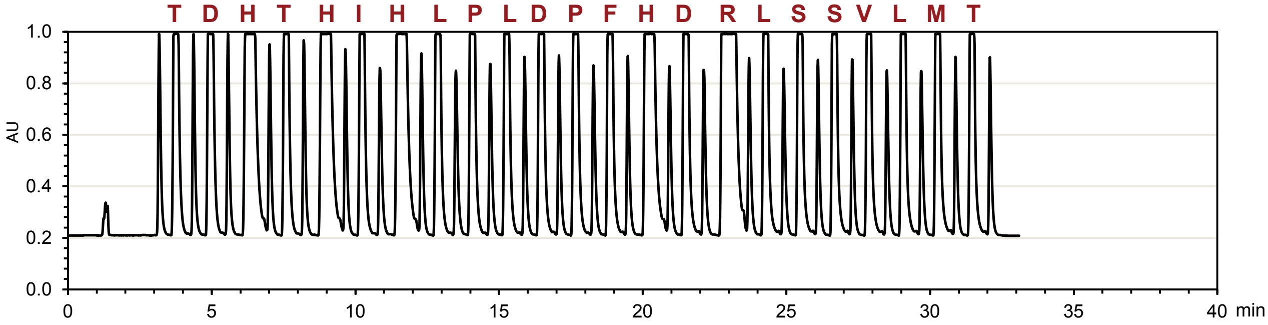

RP-HPLC trace of the unpurified peptide

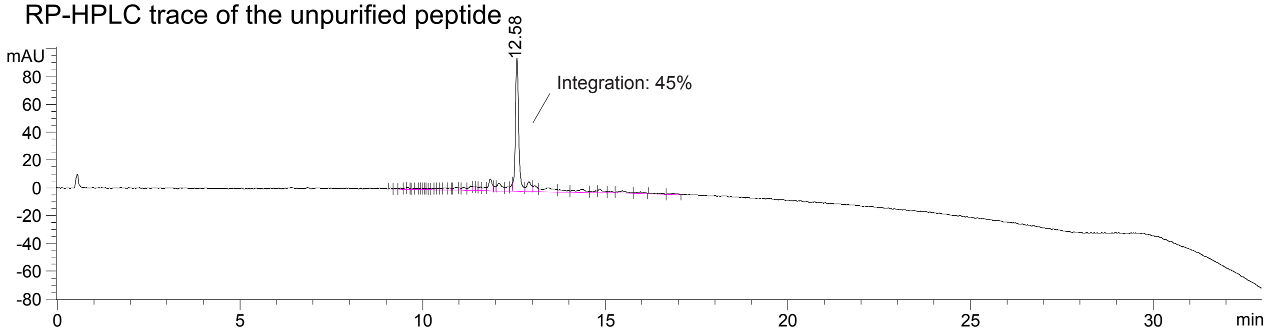

RP-HPLC trace of the purified peptide

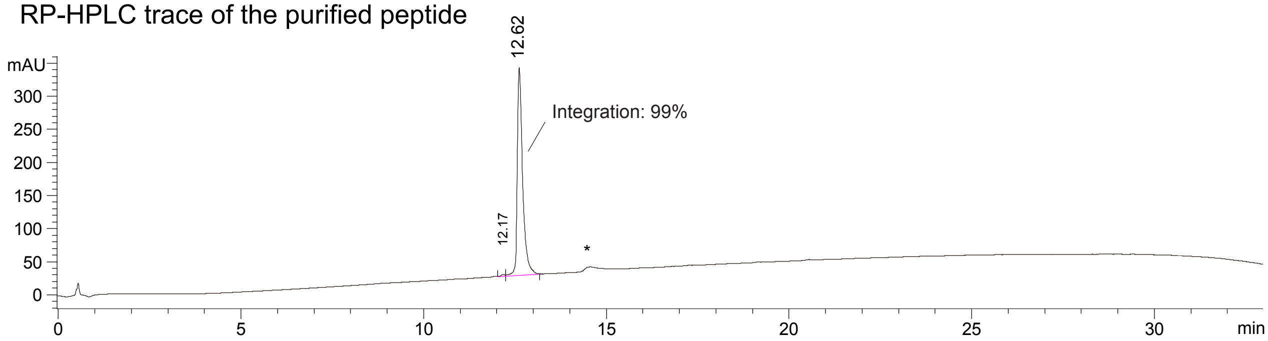

The asterisk (*) indicates an impurity from the HPLC column.

mass spectrum (ESI) of the purified peptide

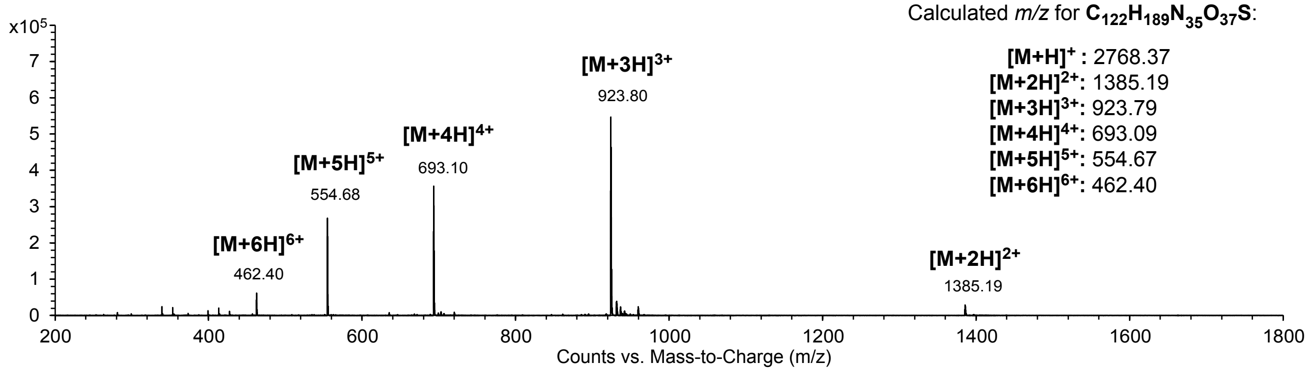

Calculated *m/z* for $C_{122}H_{189}N_{35}O_{37}S$:

$[M+H]^{+}$: 2768.37
$[M+2H]^{2+}$: 1385.19
$[M+3H]^{3+}$: 923.79
$[M+4H]^{4+}$: 693.09
$[M+5H]^{5+}$: 554.67
$[M+6H]^{6+}$: 462.40

Characterization Data for IMP **17**

UV absorbance trace from flow peptide synthesis

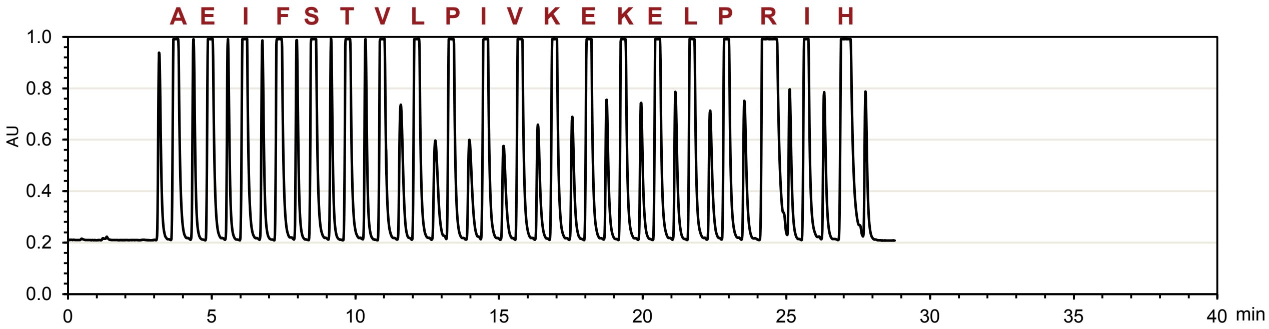

RP-HPLC trace of the unpurified peptide

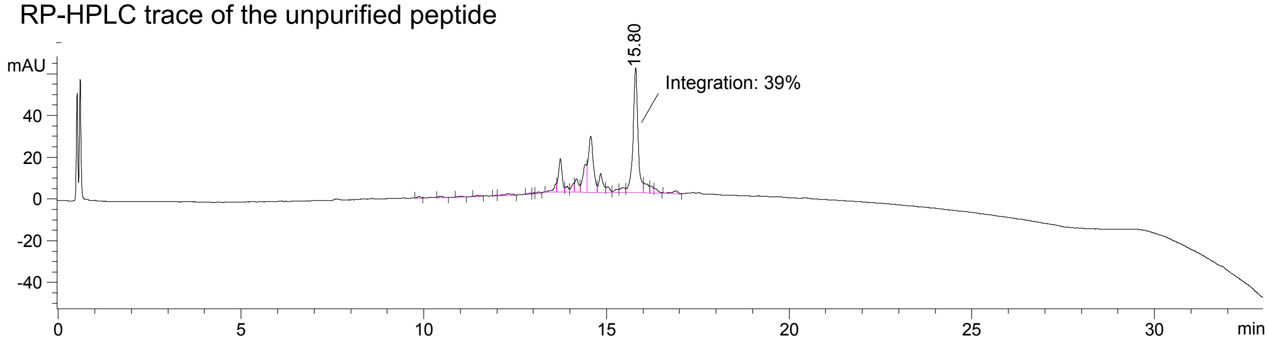

RP-HPLC trace of the purified peptide

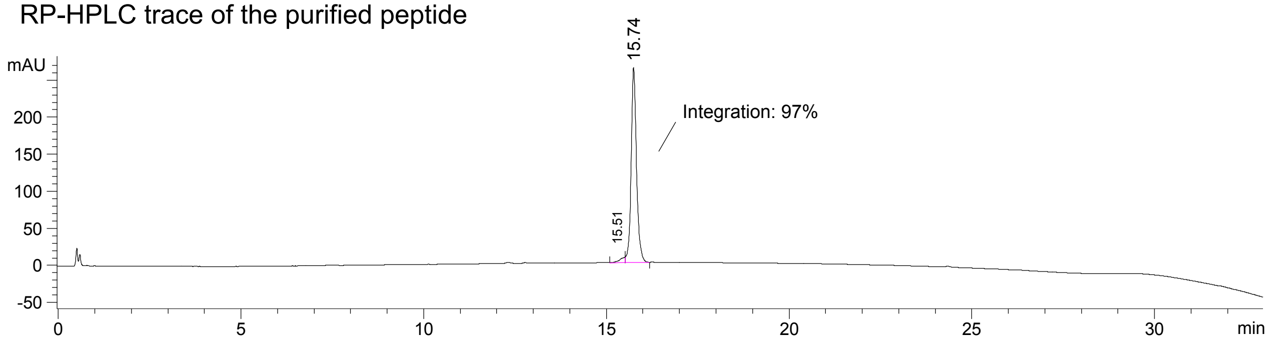

mass spectrum (ESI) of the purified peptide

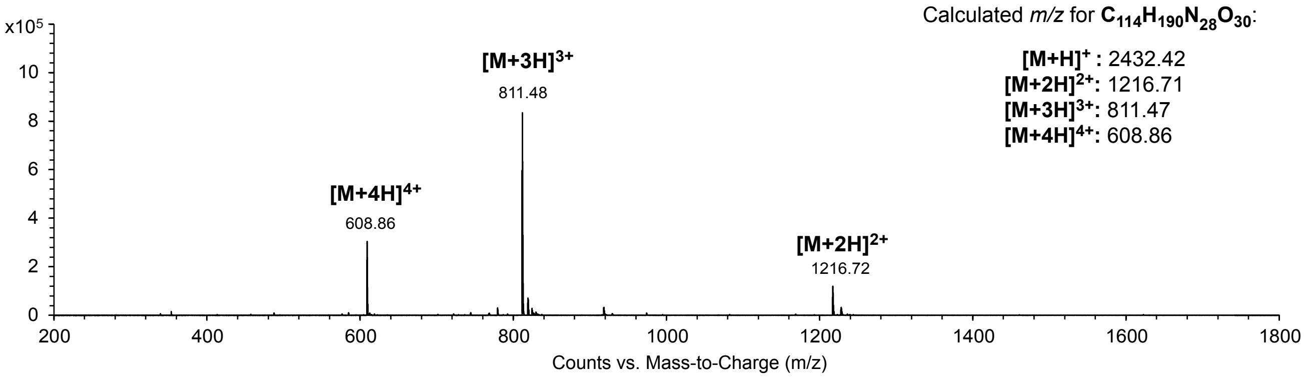

Characterization Data for IMP **18**

UV absorbance trace from flow peptide synthesis

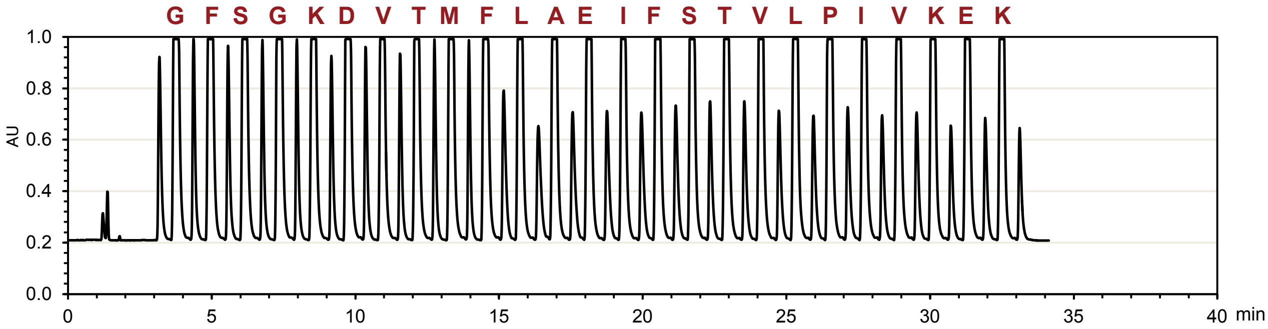

RP-HPLC trace of the unpurified peptide

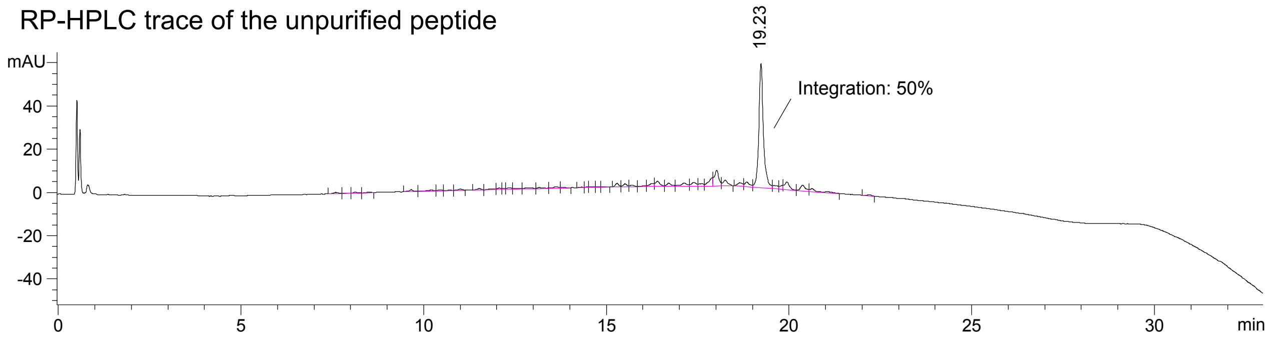

RP-HPLC trace of the purified peptide

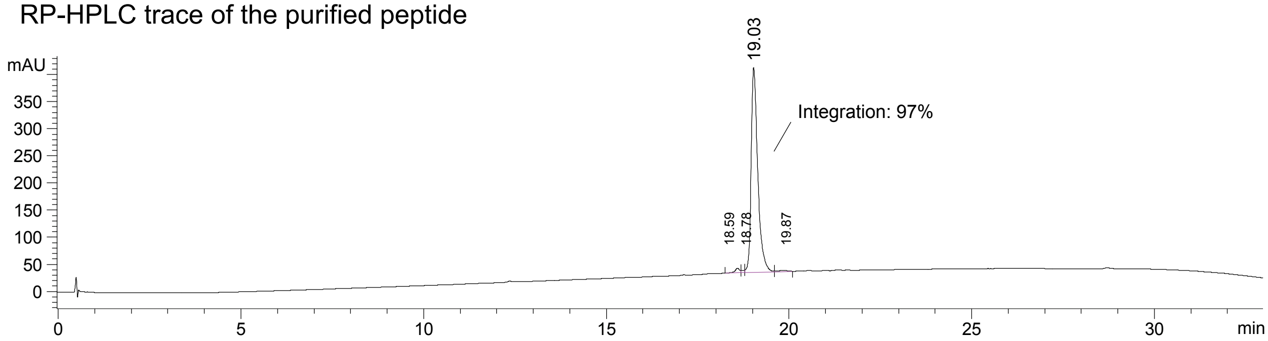

mass spectrum (ESI) of the purified peptide

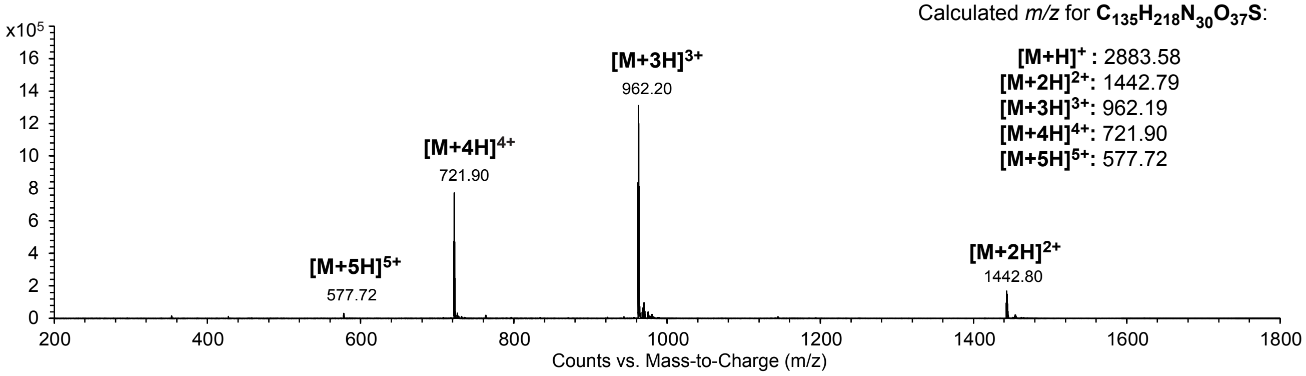

Characterization Data for IMP **19**

UV absorbance trace from flow peptide synthesis

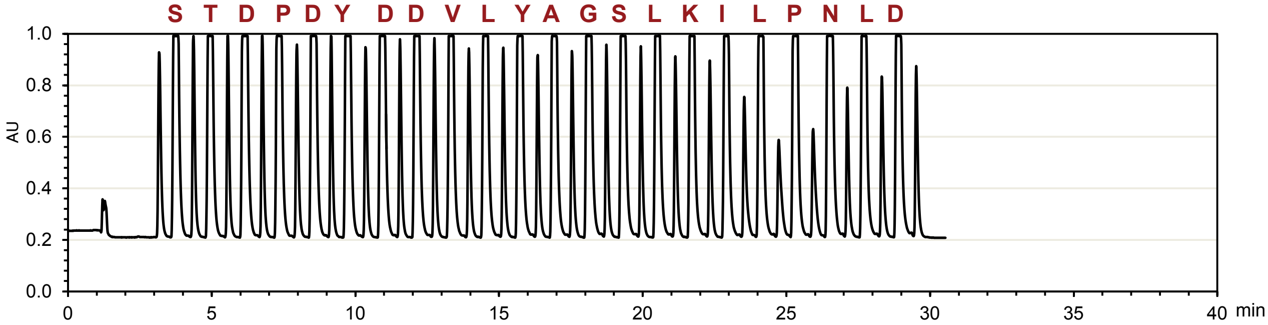

RP-HPLC trace of the unpurified peptide

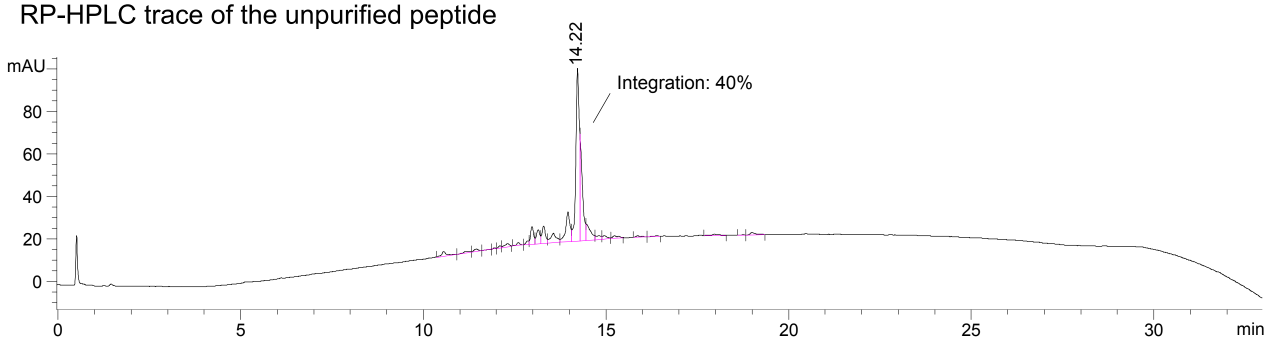

RP-HPLC trace of the purified peptide

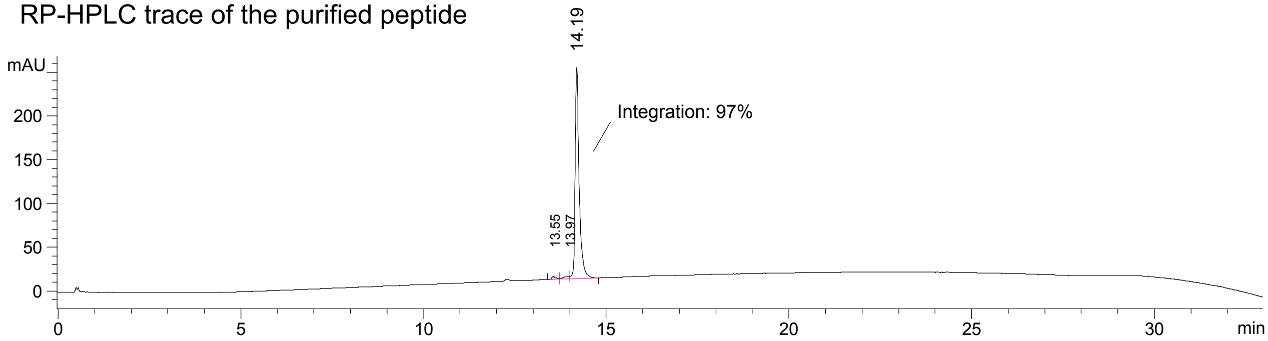

mass spectrum (ESI) of the purified peptide

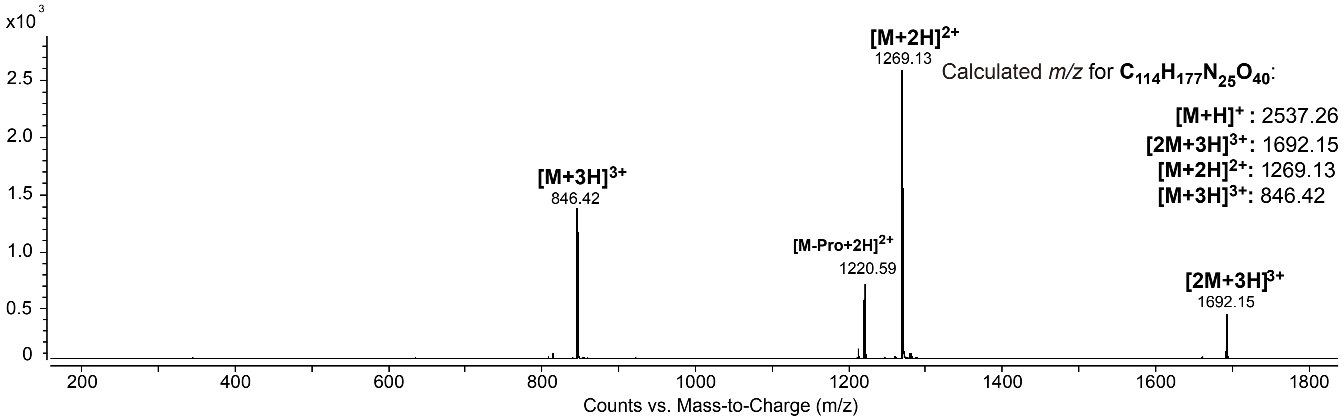

Characterization Data for IMP **20**

UV absorbance trace from flow peptide synthesis

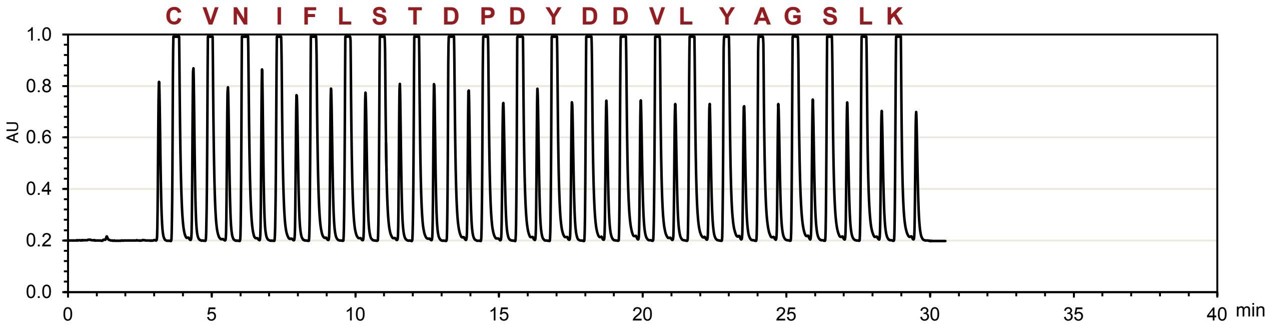

RP-HPLC trace of the unpurified peptide

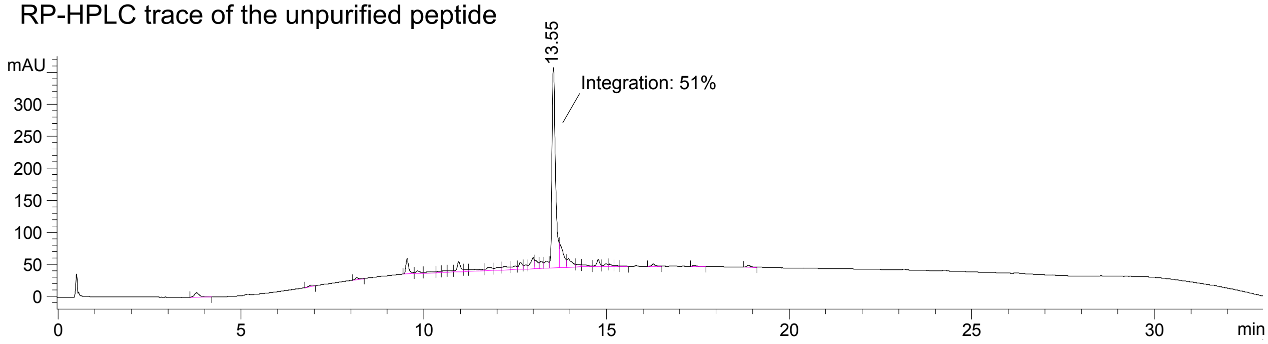

RP-HPLC trace of the purified peptide

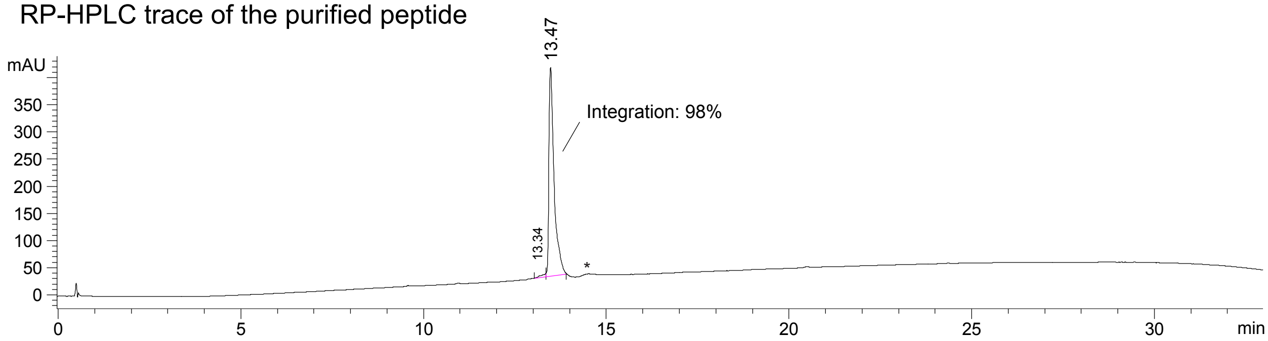

The asterisk (*) indicates an impurity from the HPLC column.

mass spectrum (ESI) of the purified peptide

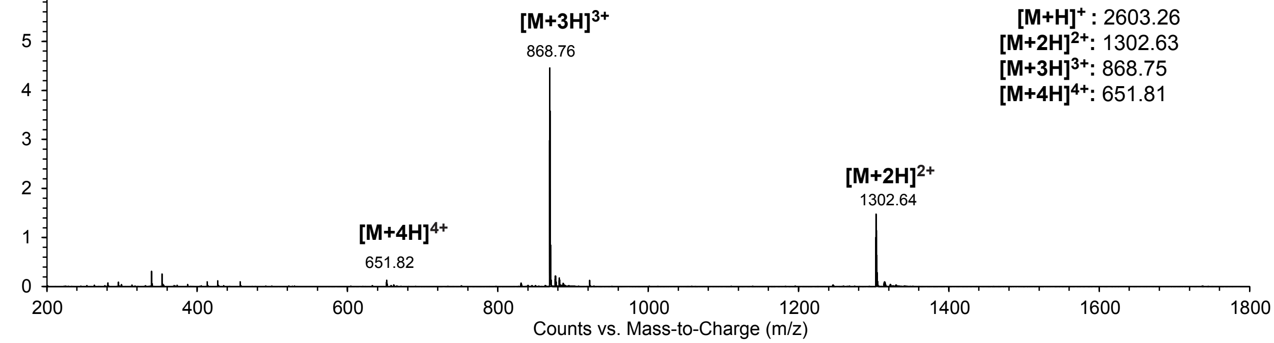

Calculated *m/z* for $C_{116}H_{178}N_{28}O_{38}S$:

$[M+H]^{+}$: 2603.26
$[M+2H]^{2+}$: 1302.63
$[M+3H]^{3+}$: 868.75
$[M+4H]^{4+}$: 651.81

Characterization Data for IMP **21**

UV absorbance trace from flow peptide synthesis

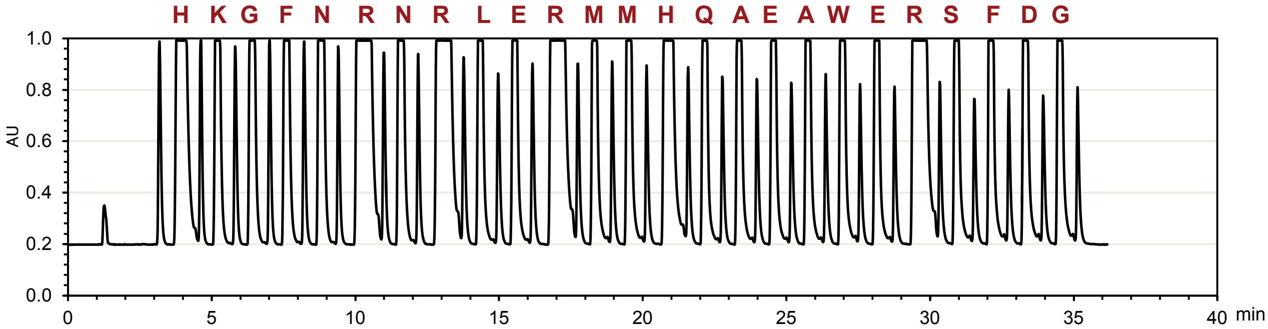

RP-HPLC trace of the unpurified peptide

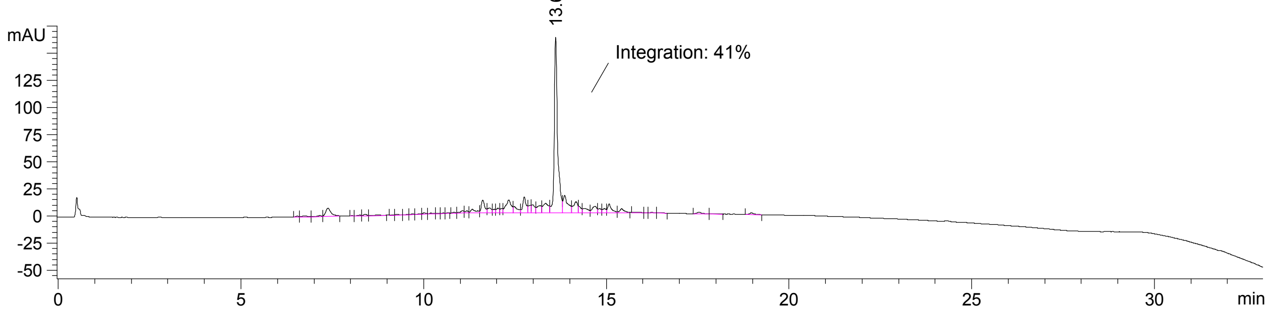

RP-HPLC trace of the purified peptide

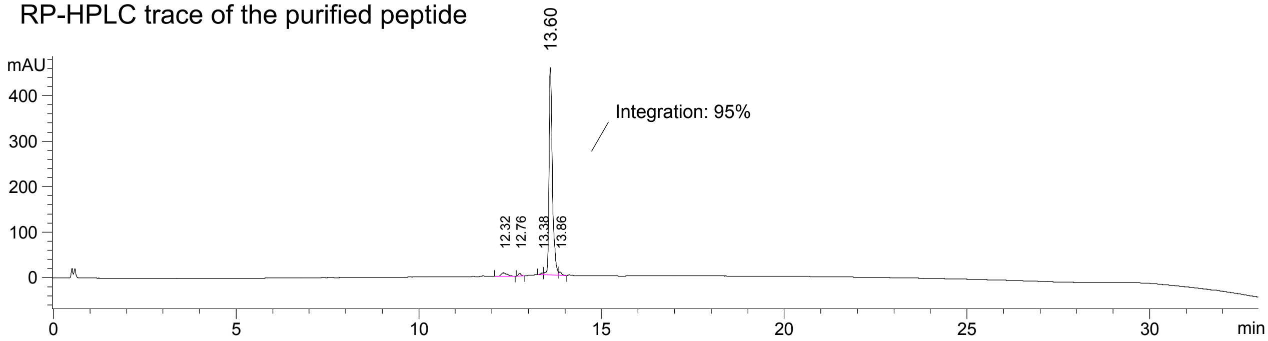

mass spectrum (ESI) of the purified peptide

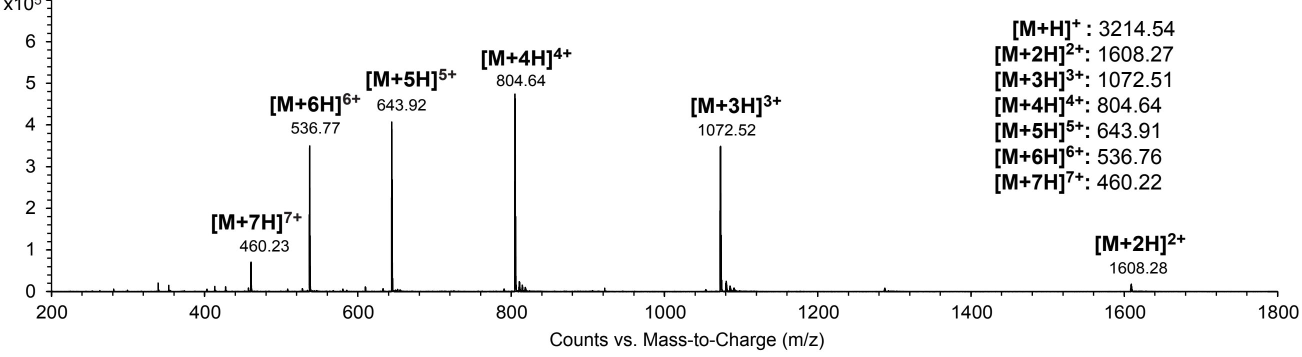

Calculated *m/z* for $C_{138}H_{211}N_{47}O_{39}S_2$:

[M+H]$^+$: 3214.54
[M+2H]$^{2+}$: 1608.27
[M+3H]$^{3+}$: 1072.51
[M+4H]$^{4+}$: 804.64
[M+5H]$^{5+}$: 643.91
[M+6H]$^{6+}$: 536.76
[M+7H]$^{7+}$: 460.22

Characterization Data for IMP **22**

UV absorbance trace from flow peptide synthesis

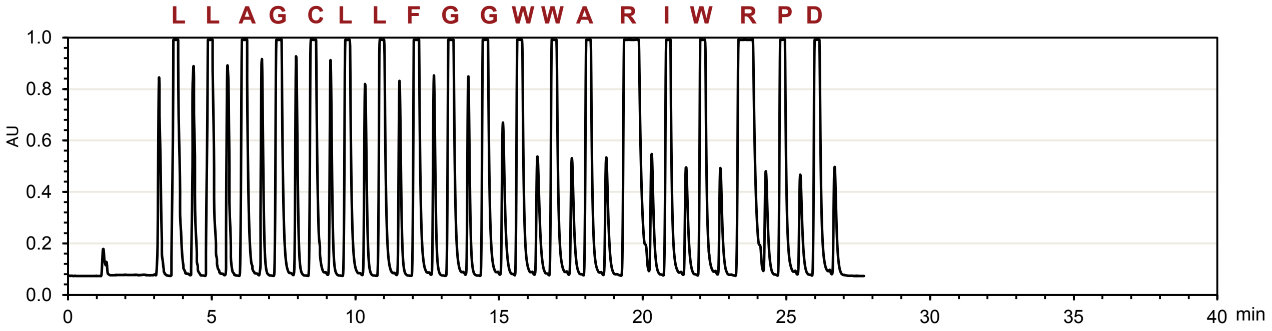

RP-HPLC trace of the unpurified peptide

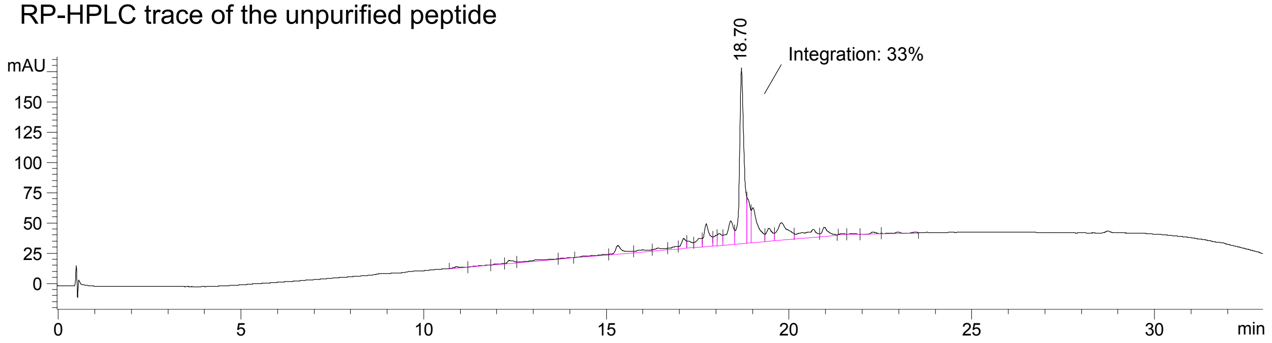

RP-HPLC trace of the purified peptide

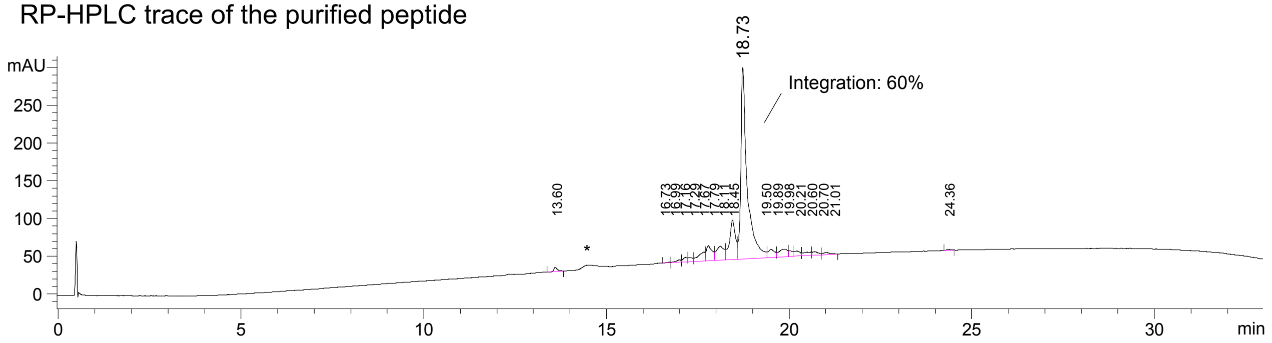

The asterisk (*) indicates an impurity from the HPLC column.

mass spectrum (ESI) of the purified peptide

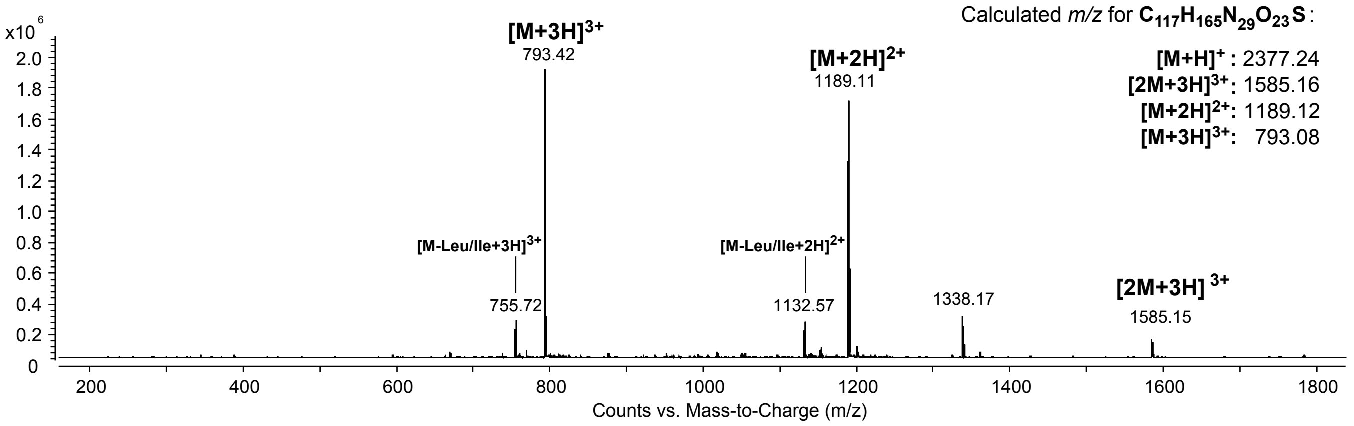

Characterization Data for IMP **23**

UV absorbance trace from flow peptide synthesis

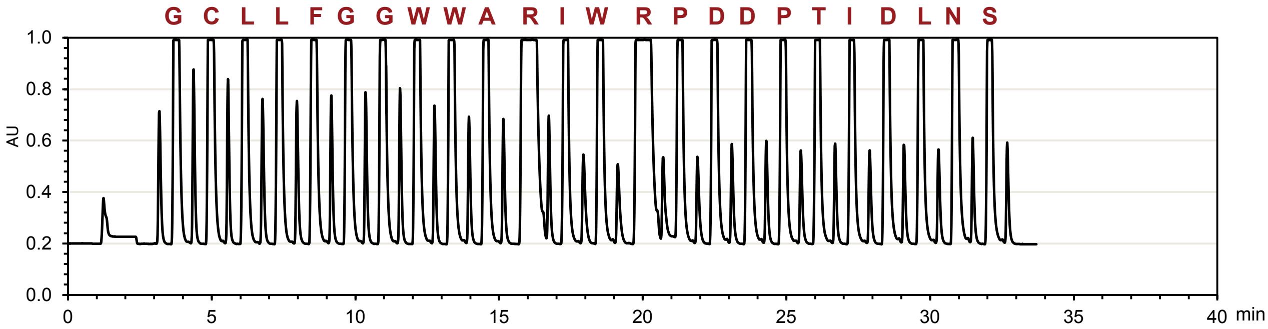

RP-HPLC trace of the unpurified peptide

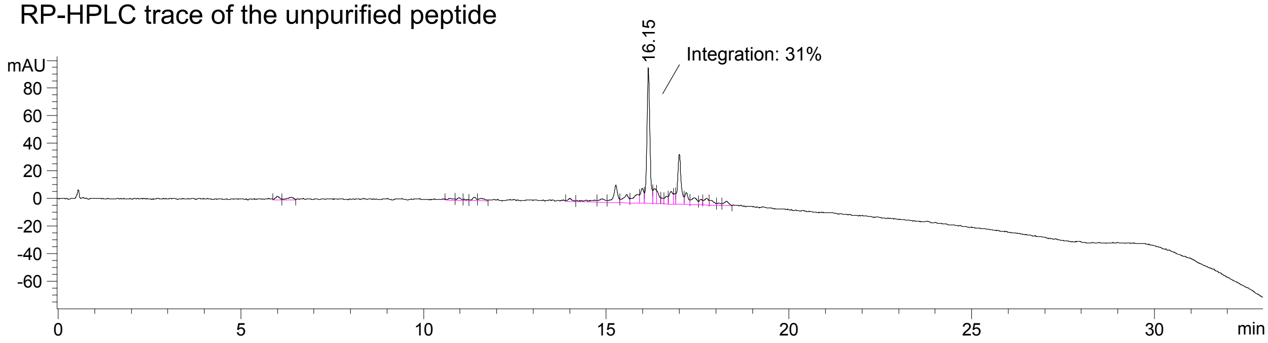

RP-HPLC trace of the purified peptide

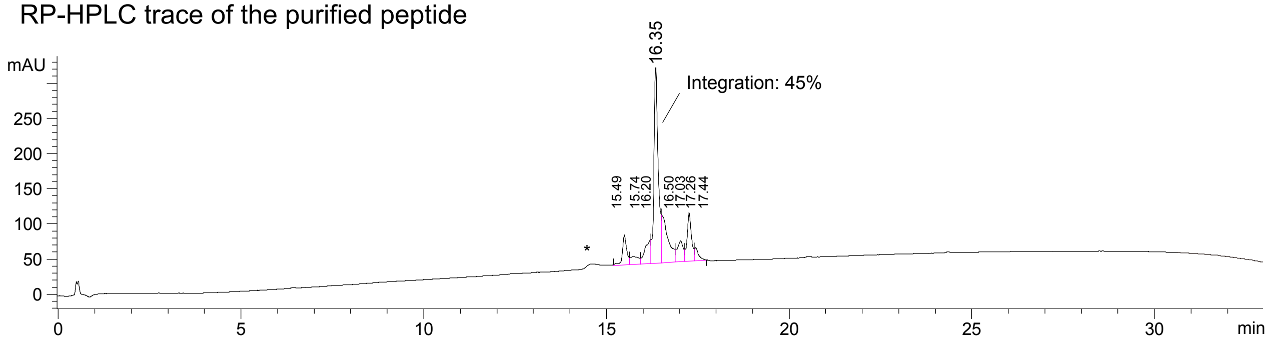

The asterisk (*) indicates an impurity from the HPLC column.

mass spectrum (ESI) of the purified peptide

Calculated *m/z* for $C_{132}H_{191}N_{35}O_{35}S$:

$[M+H]^+$: 2858.40
$[M+2H]^{2+}$: 1430.20
$[M+3H]^{3+}$: 953.80
$[M+4H]^{4+}$: 715.60

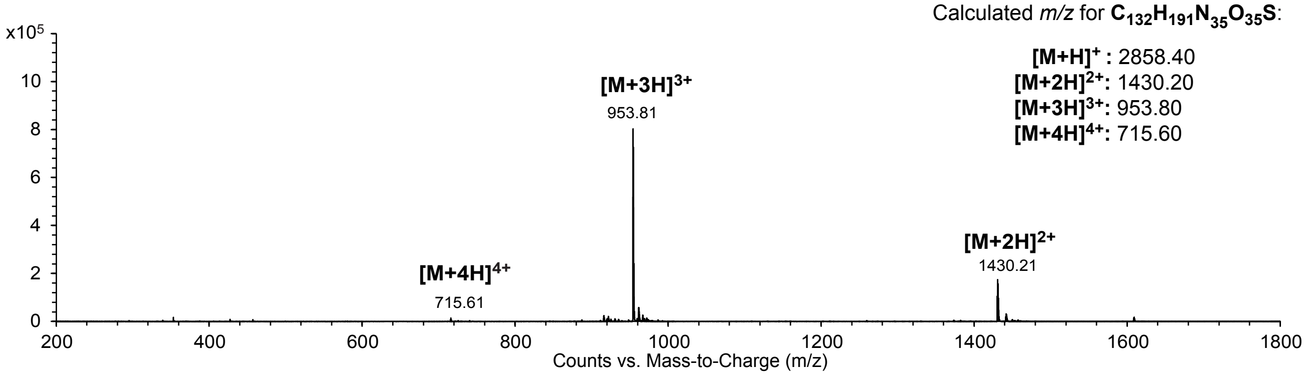

Characterization Data for IMP **24**

UV absorbance trace from flow peptide synthesis

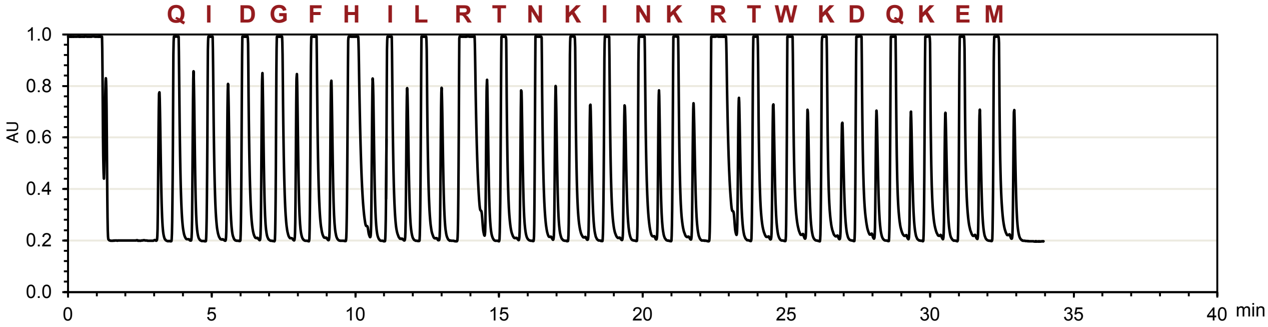

RP-HPLC trace of the unpurified peptide

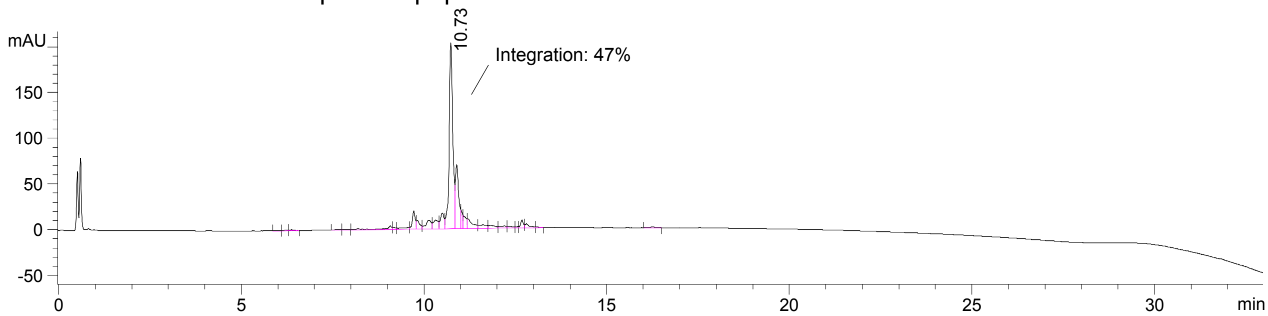

RP-HPLC trace of the purified peptide

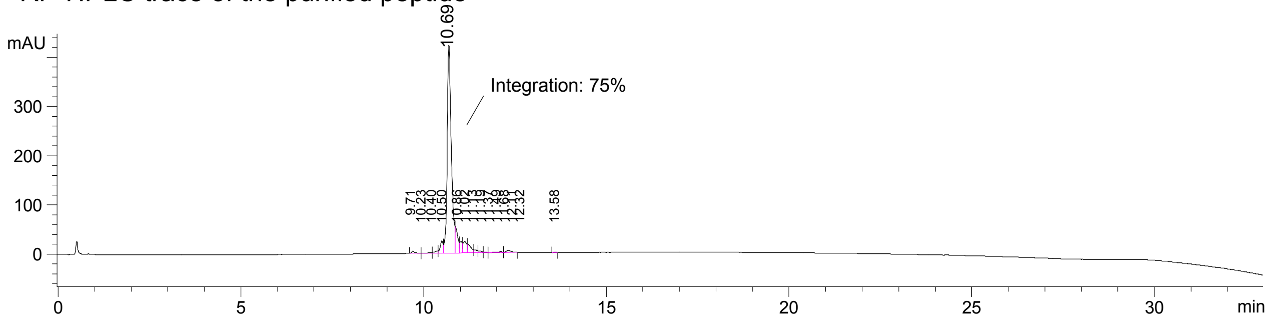

mass spectrum (ESI) of the purified peptide

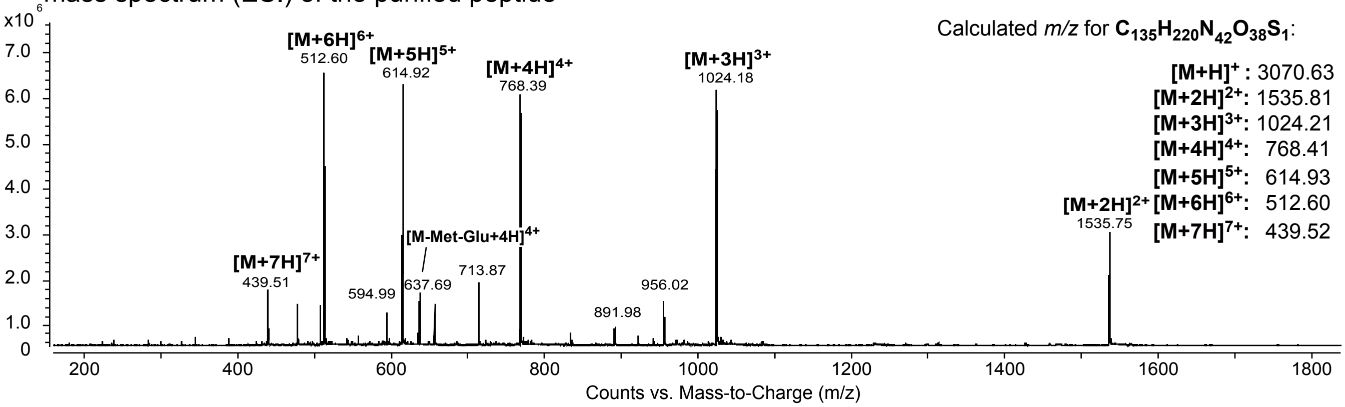

Calculated *m/z* for $\mathbf{C_{135}H_{220}N_{42}O_{38}S_1}$:

[M+H]$^+$: 3070.63
[M+2H]$^{2+}$: 1535.81
[M+3H]$^{3+}$: 1024.21
[M+4H]$^{4+}$: 768.41
[M+5H]$^{5+}$: 614.93
[M+6H]$^{6+}$: 512.60
[M+7H]$^{7+}$: 439.52

Characterization Data for IMP **25**

UV absorbance trace from flow peptide synthesis

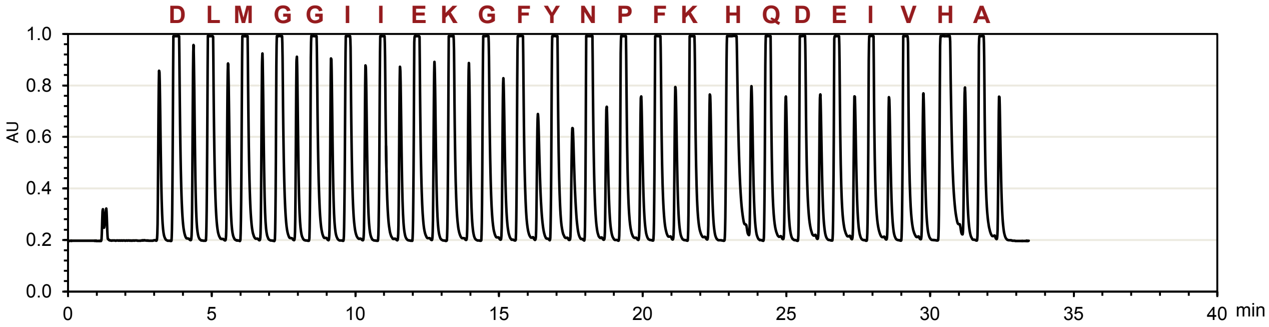

RP-HPLC trace of the unpurified peptide

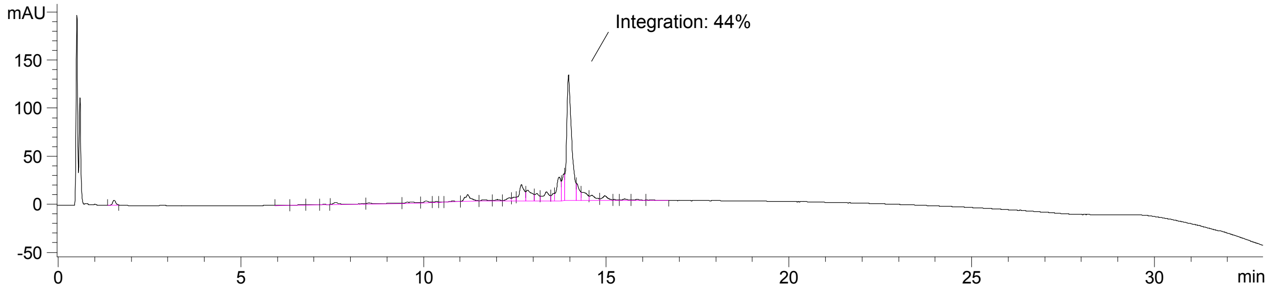

RP-HPLC trace of the purified peptide

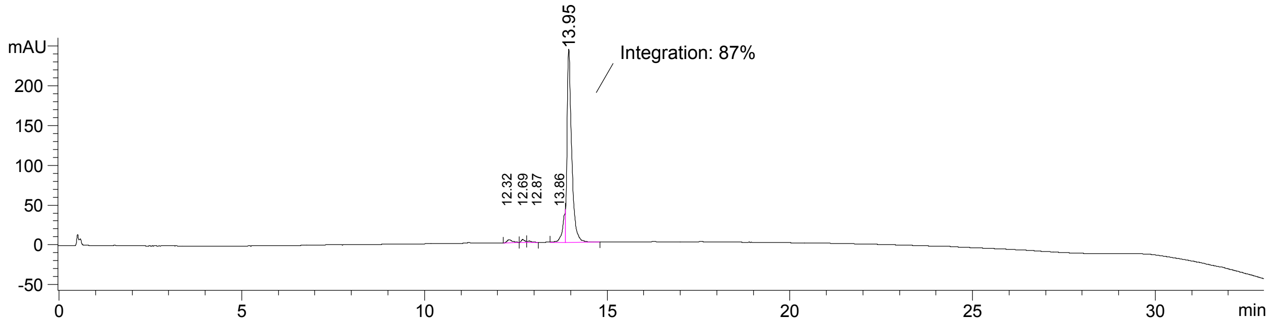

mass spectrum (ESI) of the purified peptide

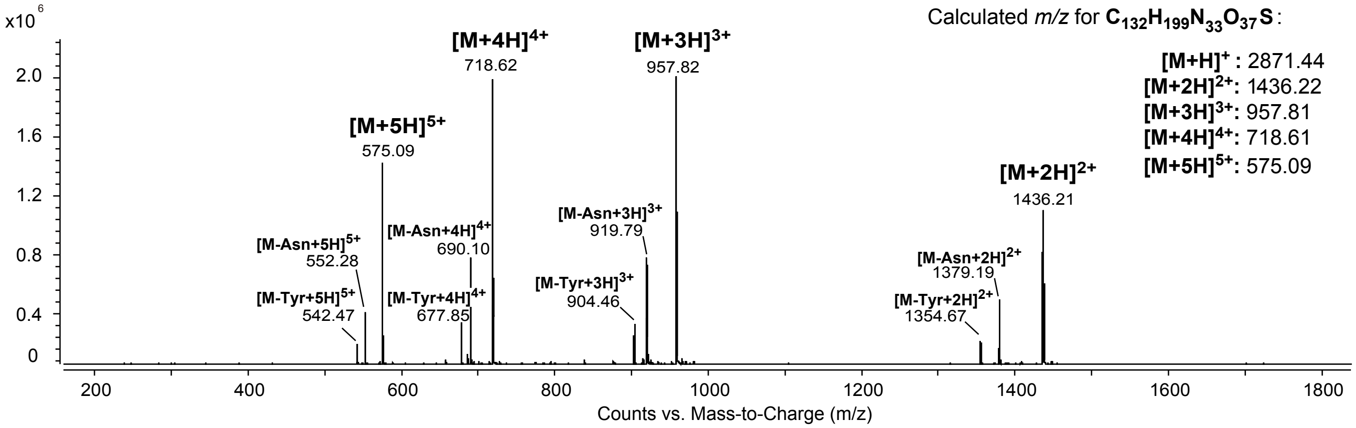

Characterization Data for IMP **26**

UV absorbance trace from flow peptide synthesis

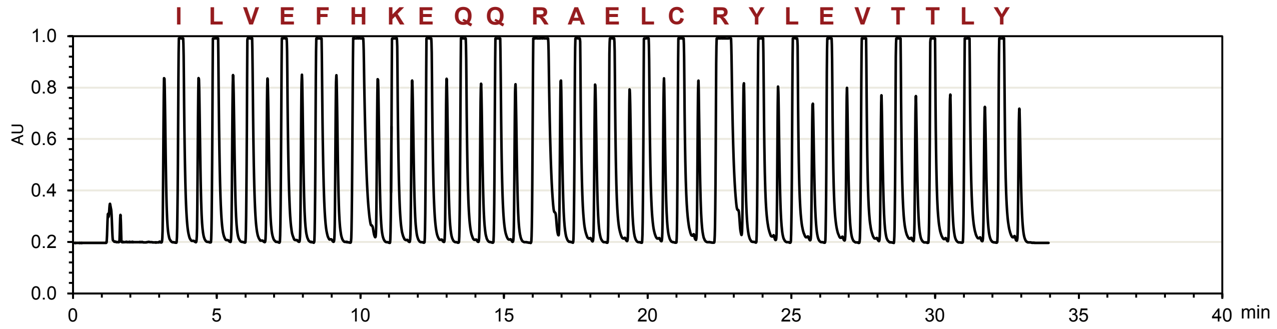

RP-HPLC trace of the unpurified peptide

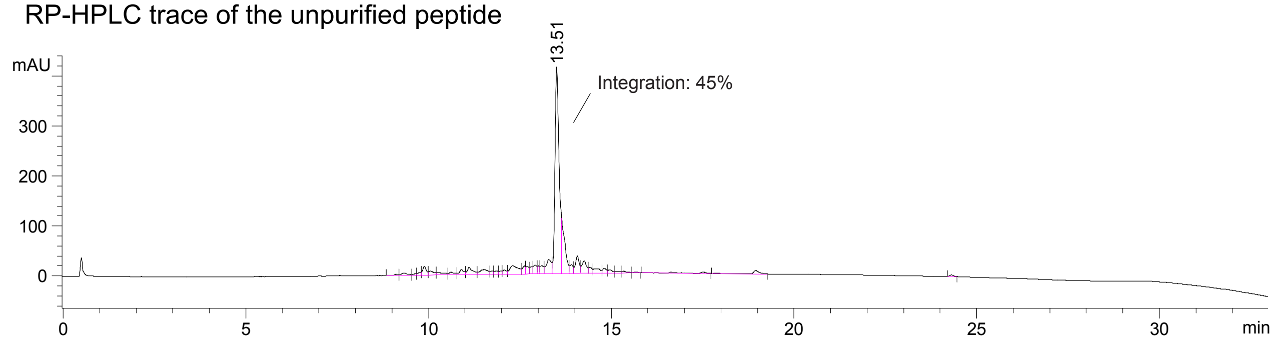

RP-HPLC trace of the purified peptide

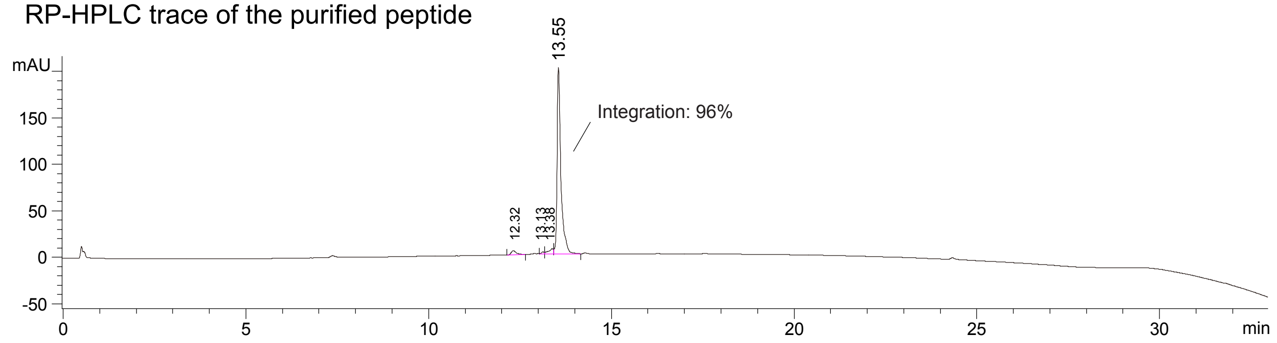

mass spectrum (ESI) of the purified peptide

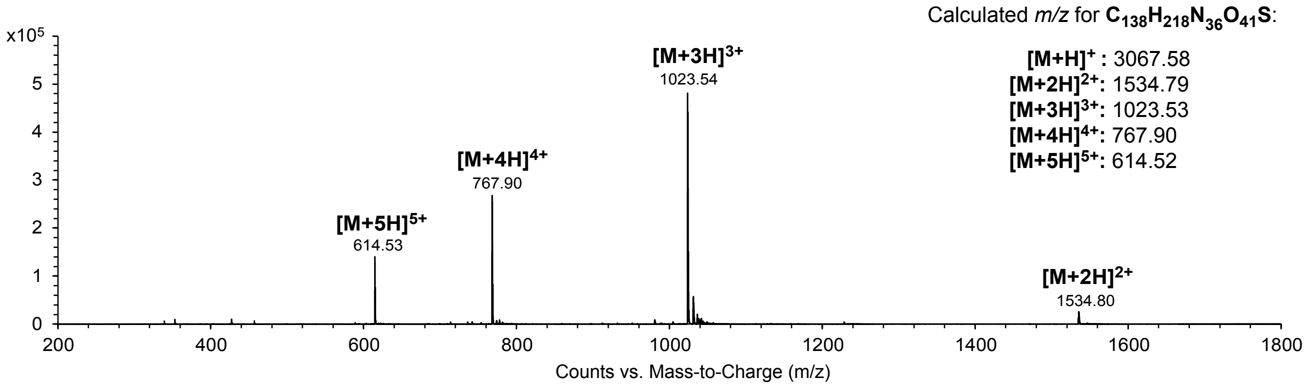

Characterization Data for IMP **27**

UV absorbance trace from flow peptide synthesis

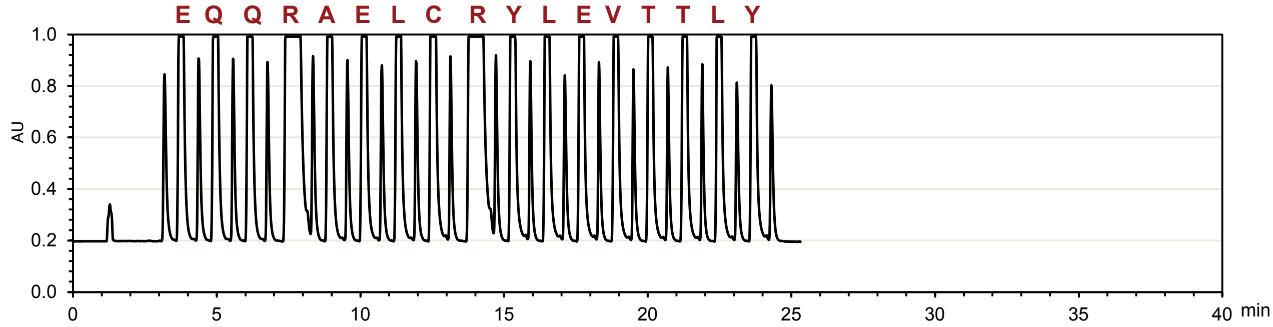

RP-HPLC trace of the unpurified peptide

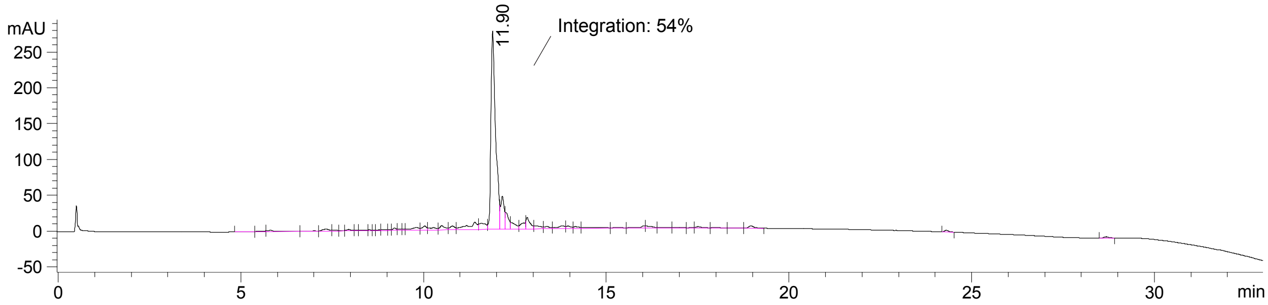

RP-HPLC trace of the purified peptide

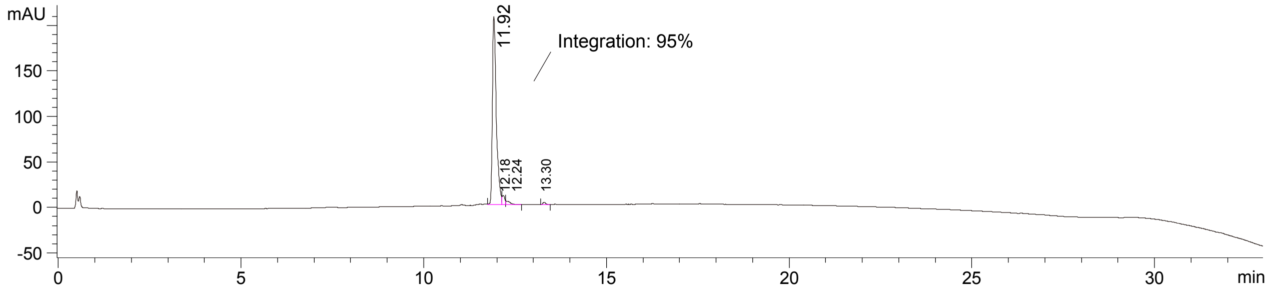

mass spectrum (ESI) of the purified peptide

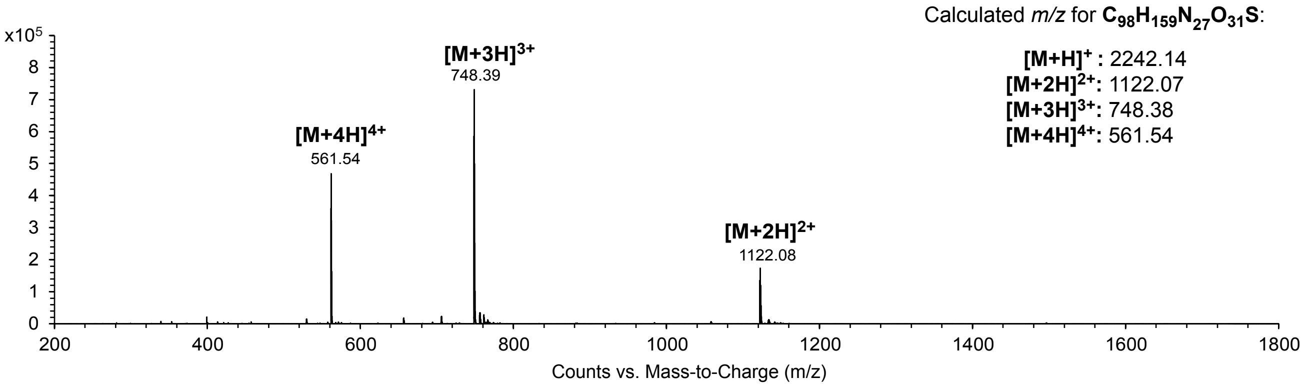

Characterization Data for IMP **28**

UV absorbance trace from flow peptide synthesis

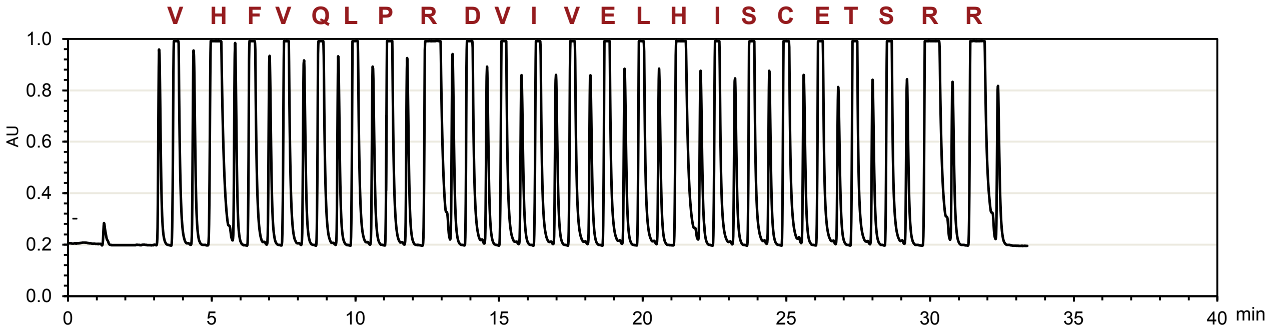

RP-HPLC trace of the unpurified peptide

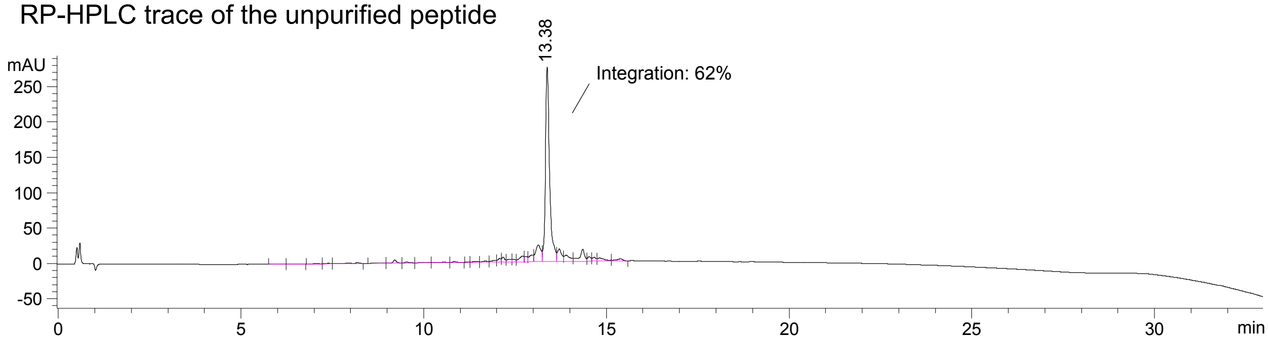

RP-HPLC trace of the purified peptide

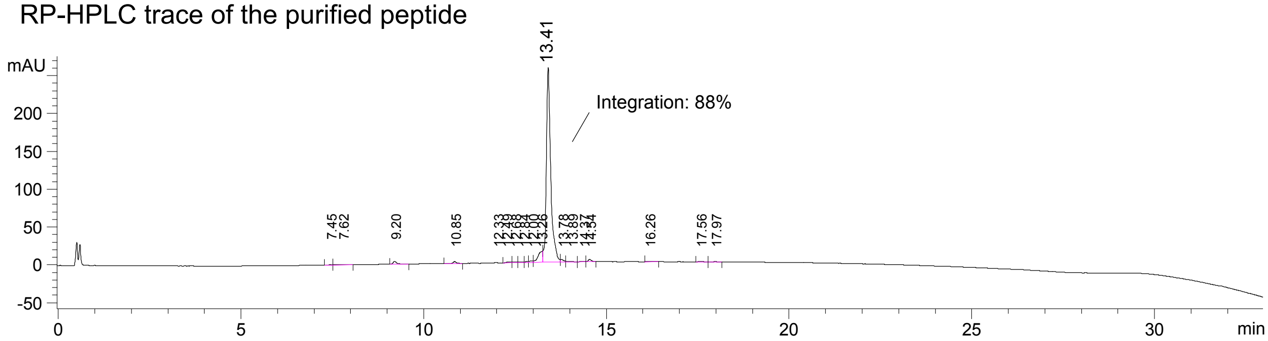

mass spectrum (ESI) of the purified peptide

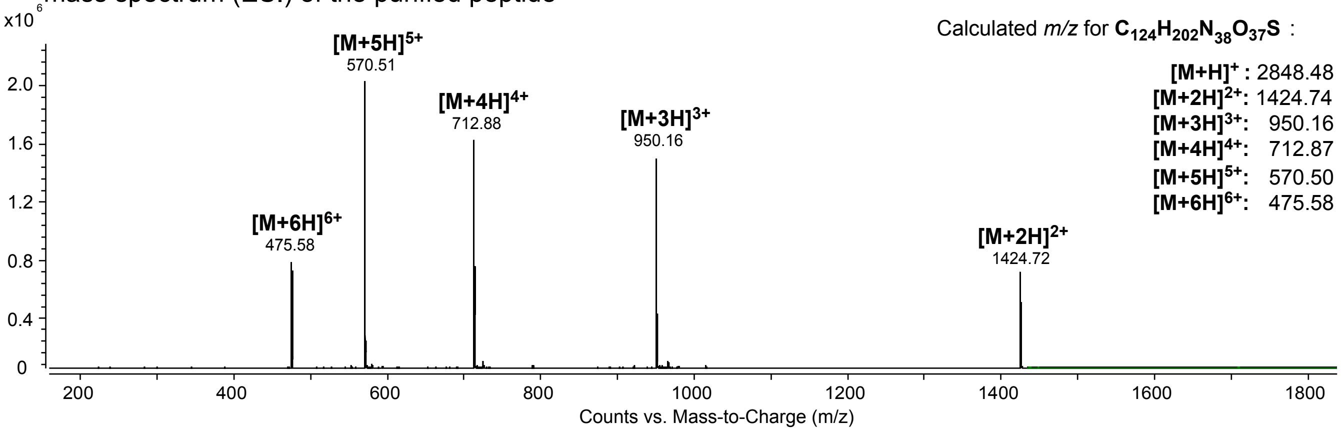

Calculated *m/z* for $C_{124}H_{202}N_{38}O_{37}S$:

$[M+H]^+$: 2848.48
$[M+2H]^{2+}$: 1424.74
$[M+3H]^{3+}$: 950.16
$[M+4H]^{4+}$: 712.87
$[M+5H]^{5+}$: 570.50
$[M+6H]^{6+}$: 475.58

Characterization Data for IMP **29**

UV absorbance trace from flow peptide synthesis

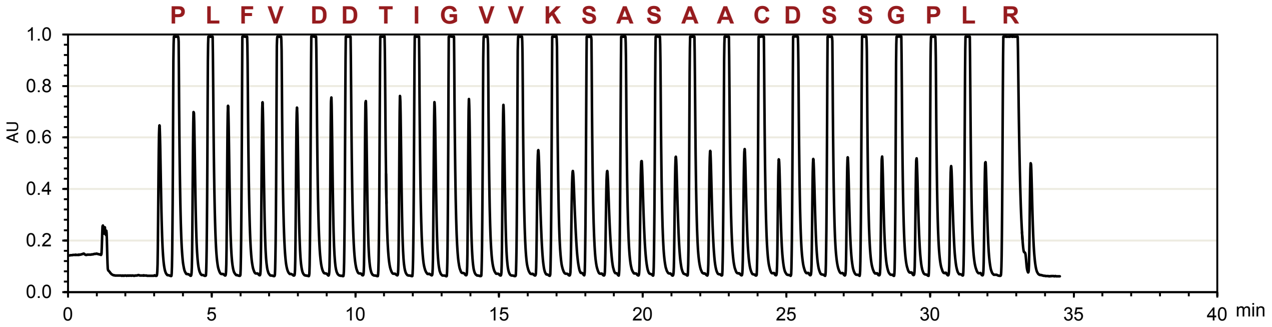

RP-HPLC trace of the unpurified peptide

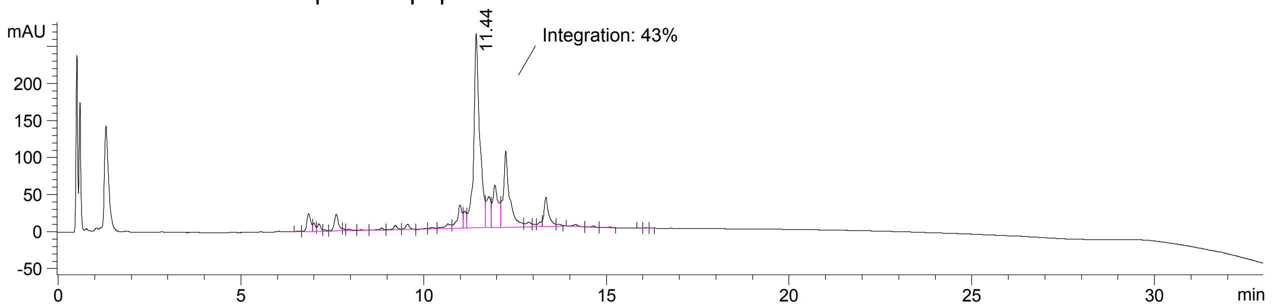

RP-HPLC trace of the purified peptide

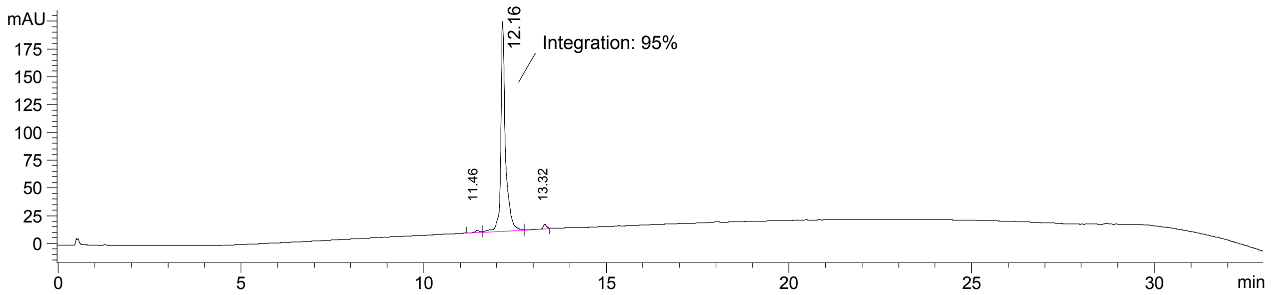

mass spectrum (ESI) of the purified peptide

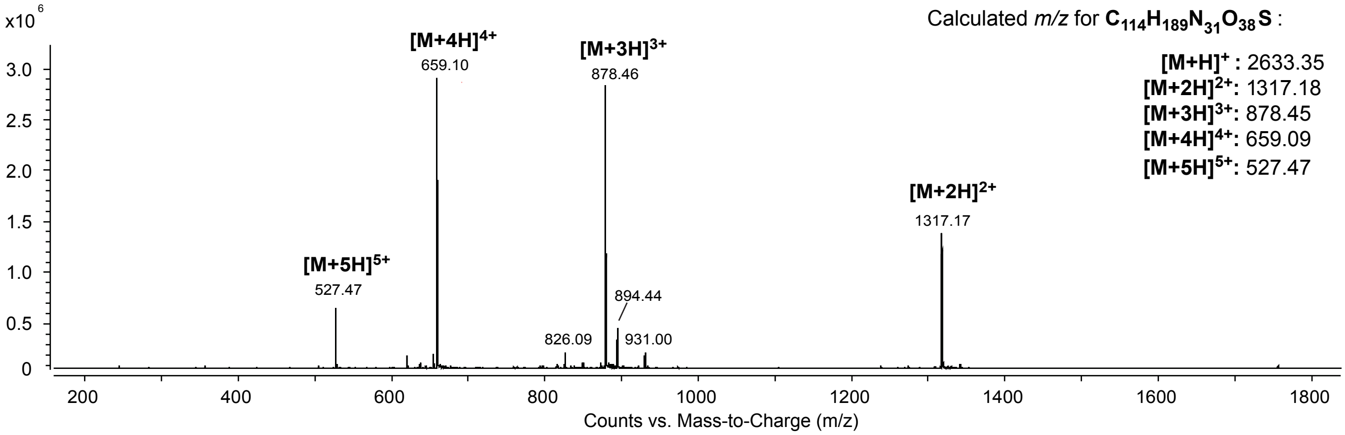

Calculated *m/z* for $C_{114}H_{189}N_{31}O_{38}S$:

$[M+H]^{+}$: 2633.35
$[M+2H]^{2+}$: 1317.18
$[M+3H]^{3+}$: 878.45
$[M+4H]^{4+}$: 659.09
$[M+5H]^{5+}$: 527.47

Characterization Data for IMP **1** from Commercial Vendor
RP-HPLC trace of the purified peptide

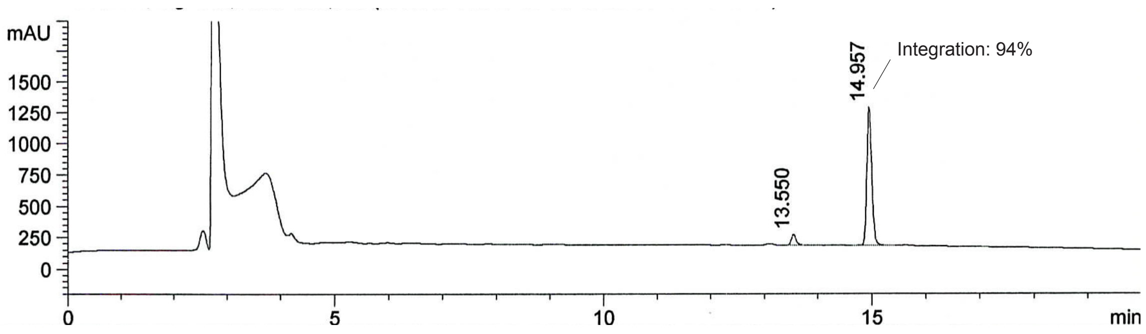

mass spectrum (ESI) of the purified peptide

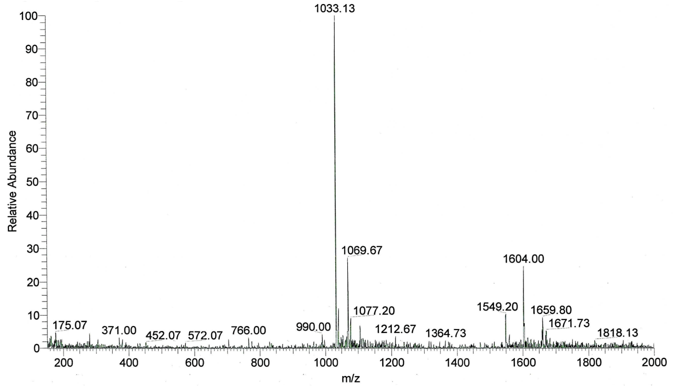

Characterization Data for IMP **2** from Commercial Vendor
RP-HPLC trace of the purified peptide

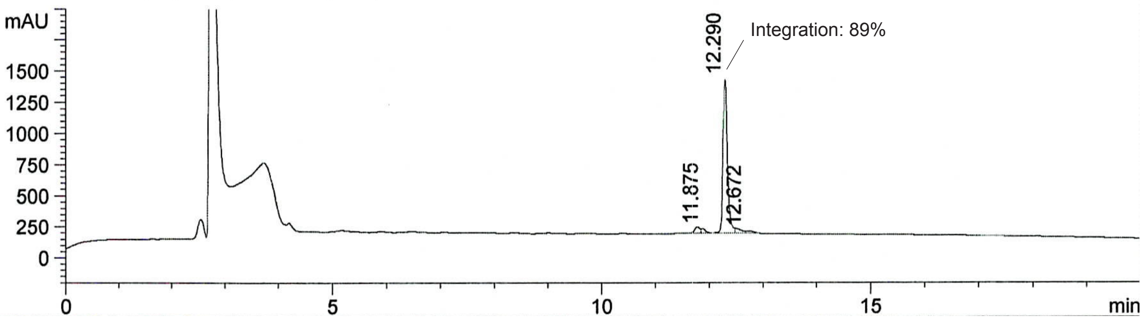

mass spectrum (ESI) of the purified peptide

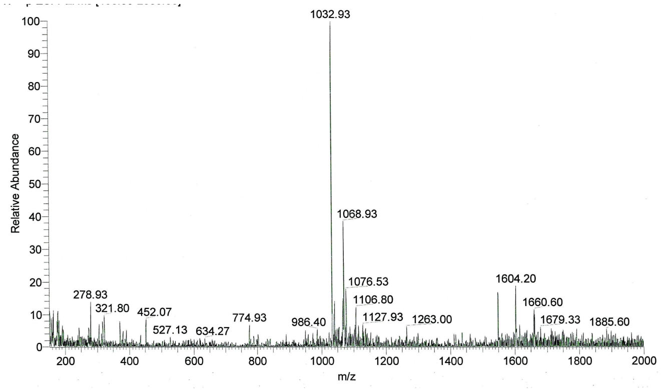

Characterization Data for IMP **5** from Commercial Vendor
RP-HPLC trace of the purified peptide

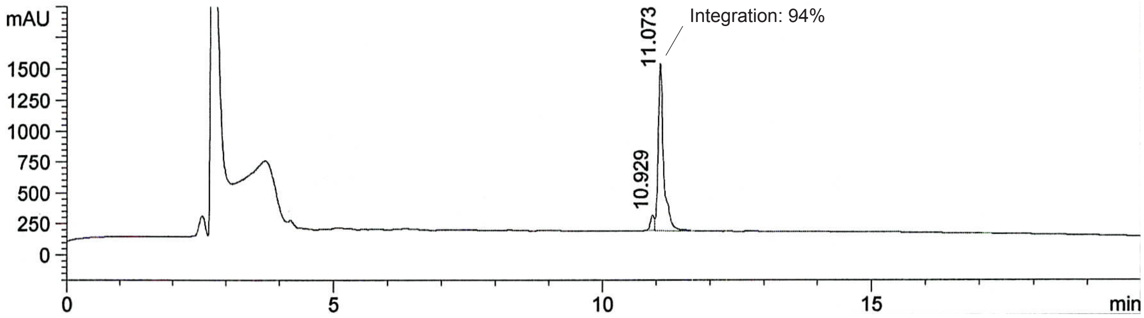

mass spectrum (ESI) of the purified peptide

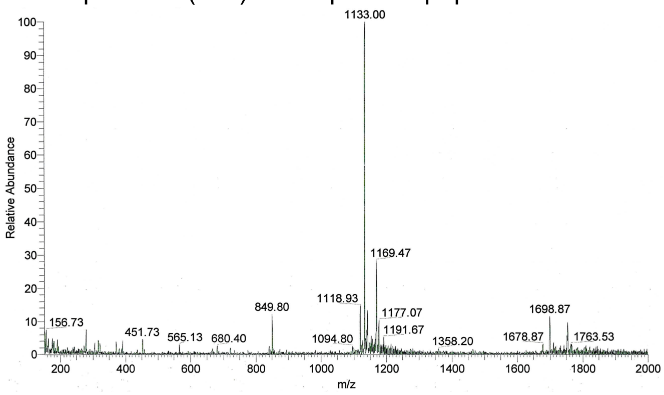

Characterization Data for IMP **6** from Commercial Vendor
RP-HPLC trace of the purified peptide

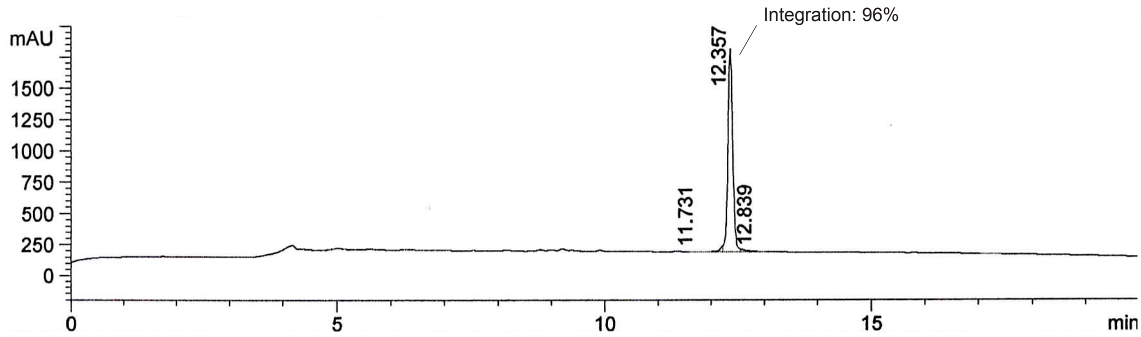

mass spectrum (ESI) of the purified peptide

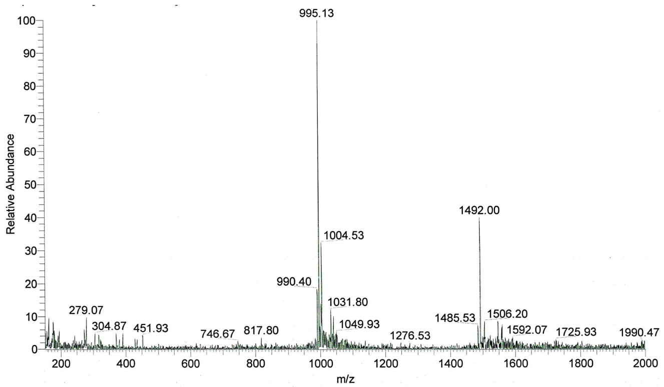

Characterization Data for IMP **07** from Commercial Vendor

RP-HPLC trace of the purified peptide

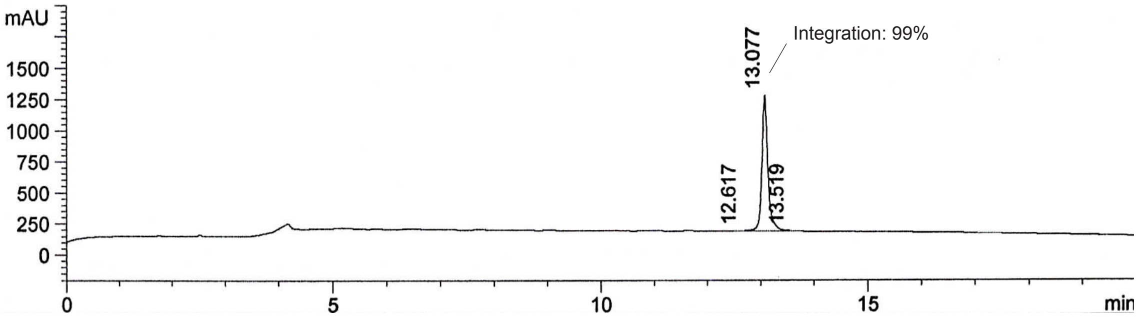

mass spectrum (ESI) of the purified peptide

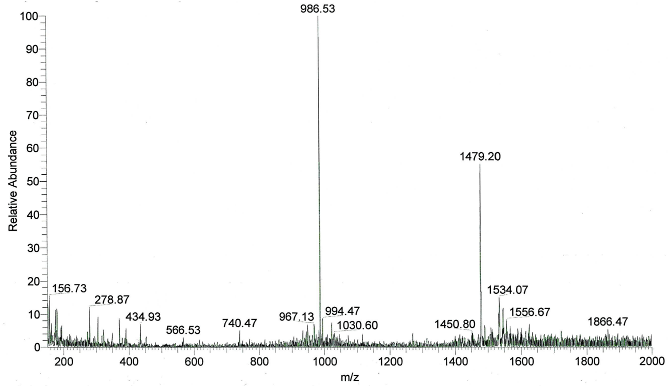

Characterization Data for IMP **09** from Commercial Vendor

RP-HPLC trace of the purified peptide

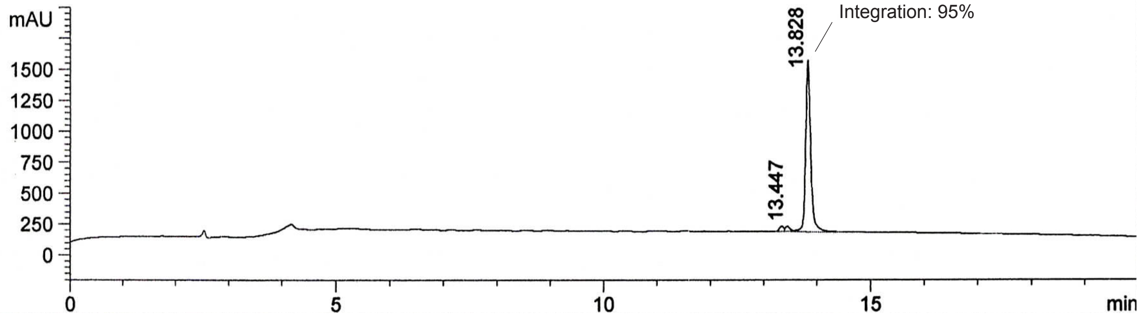

mass spectrum (ESI) of the purified peptide

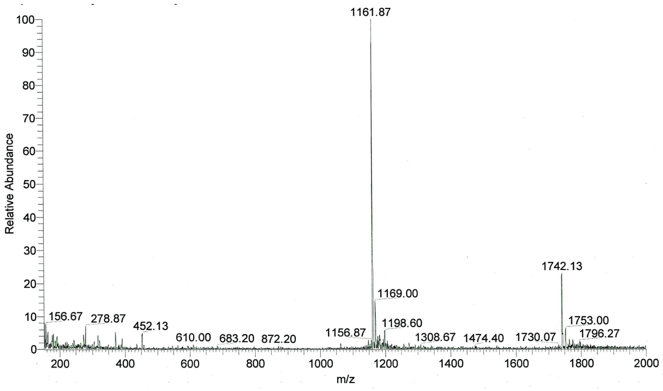

Characterization Data for IMP **15** from Commercial Vendor

RP-HPLC trace of the purified peptide

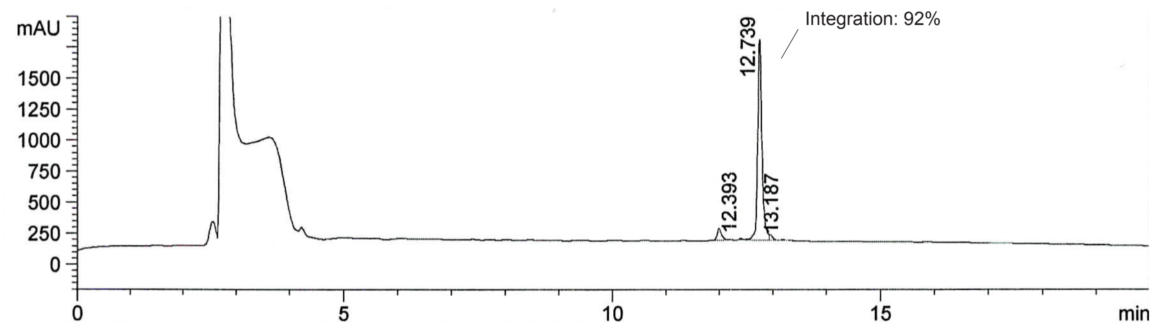

mass spectrum (ESI) of the purified peptide

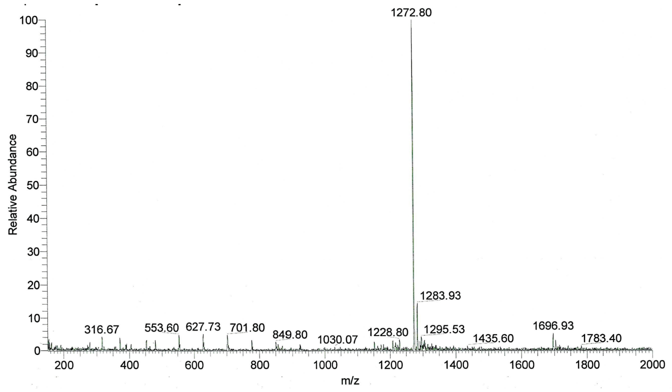

Characterization Data for IMP **16** from Commercial Vendor

RP-HPLC trace of the purified peptide

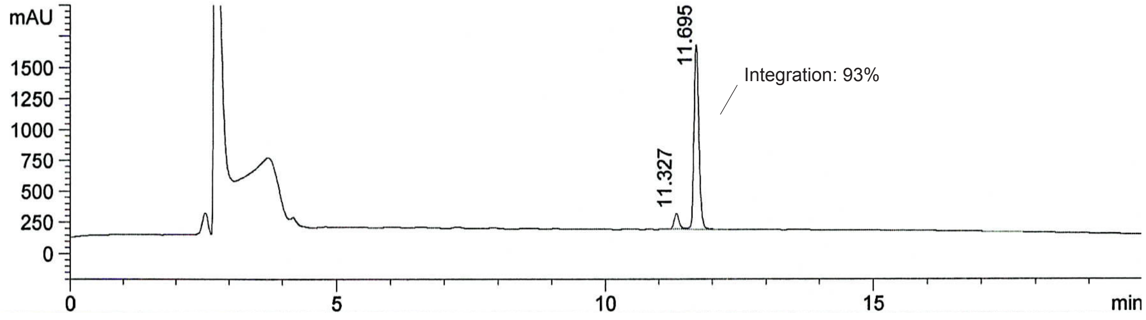

mass spectrum (ESI) of the purified peptide

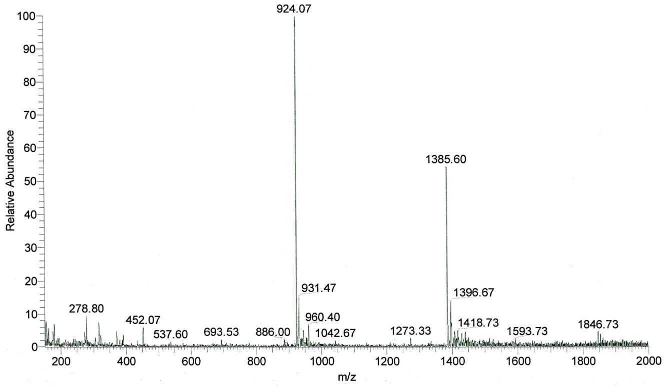

Characterization Data for IMP **17** from Commercial Vendor

RP-HPLC trace of the purified peptide

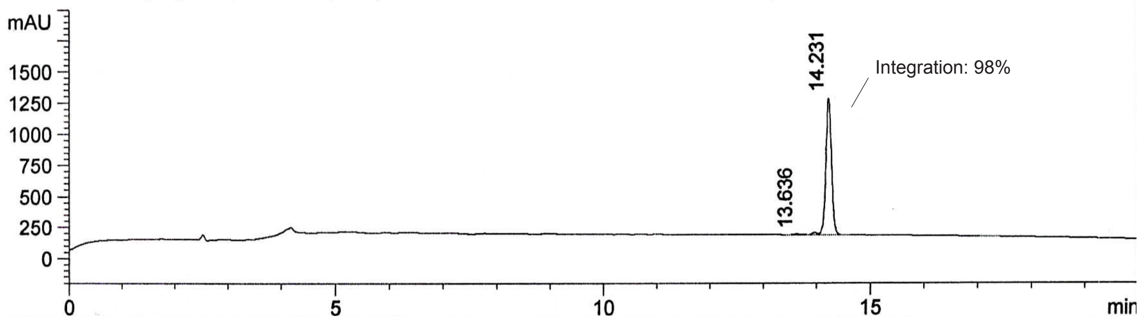

mass spectrum (ESI) of the purified peptide

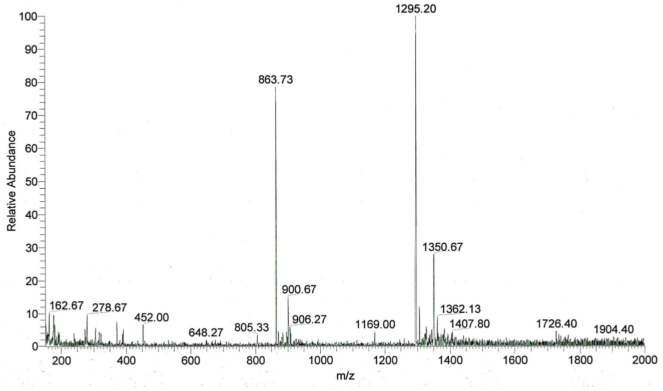

Characterization Data for IMP **19** from Commercial Vendor

RP-HPLC trace of the purified peptide

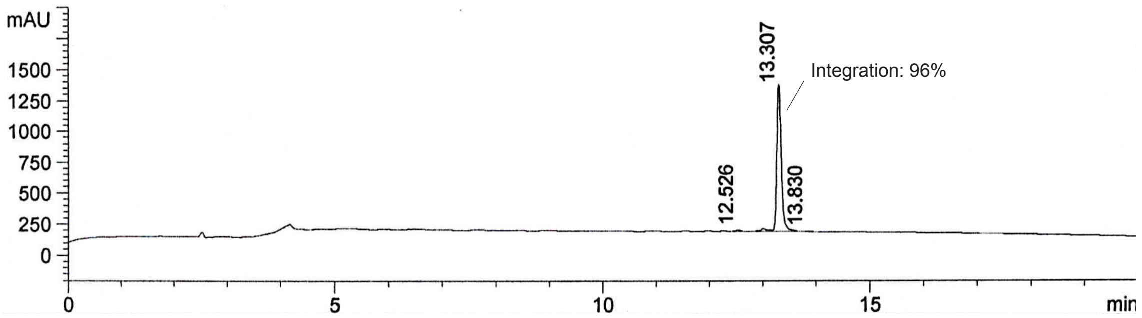

mass spectrum (ESI) of the purified peptide

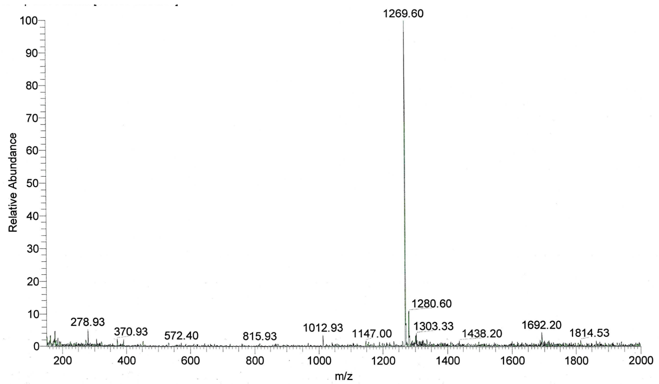

Characterization Data for IMP **21** from Commercial Vendor
RP-HPLC trace of the purified peptide

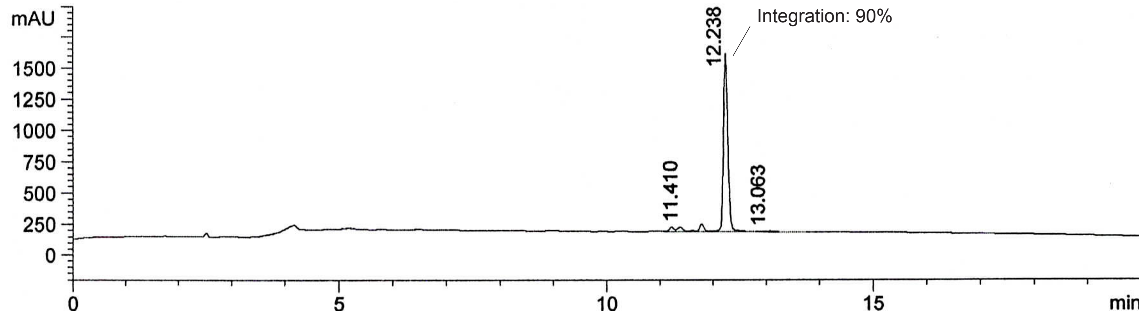

mass spectrum (ESI) of the purified peptide

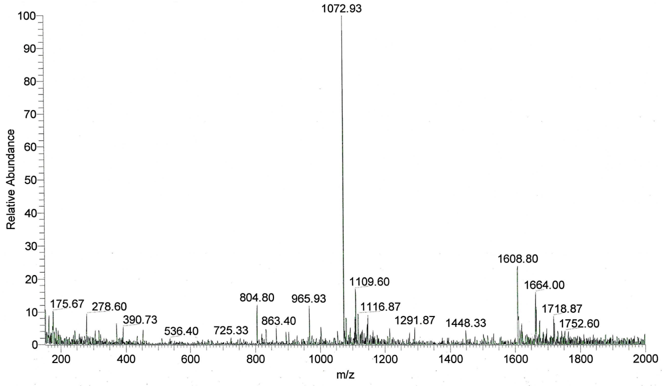

Characterization Data for IMP **24** from Commercial Vendor
RP-HPLC trace of the purified peptide

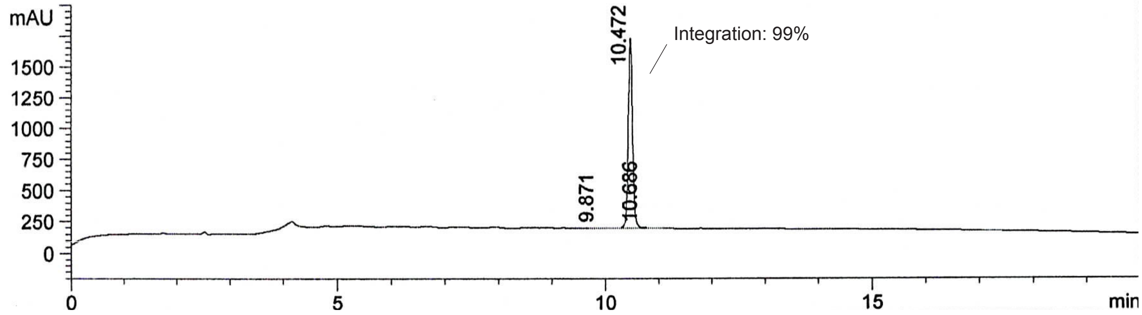

mass spectrum (ESI) of the purified peptide

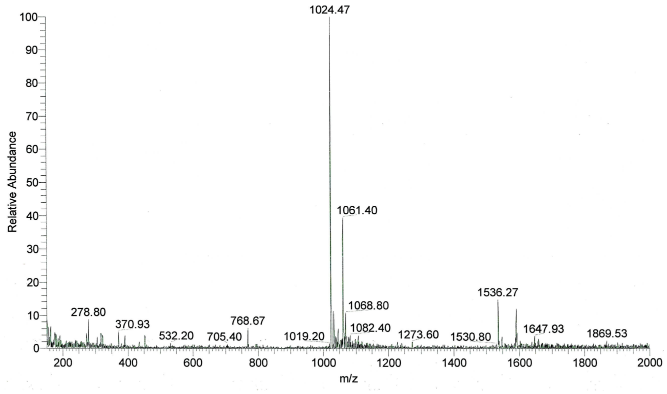

Characterization Data for IMP **25** from Commercial Vendor

RP-HPLC trace of the purified peptide

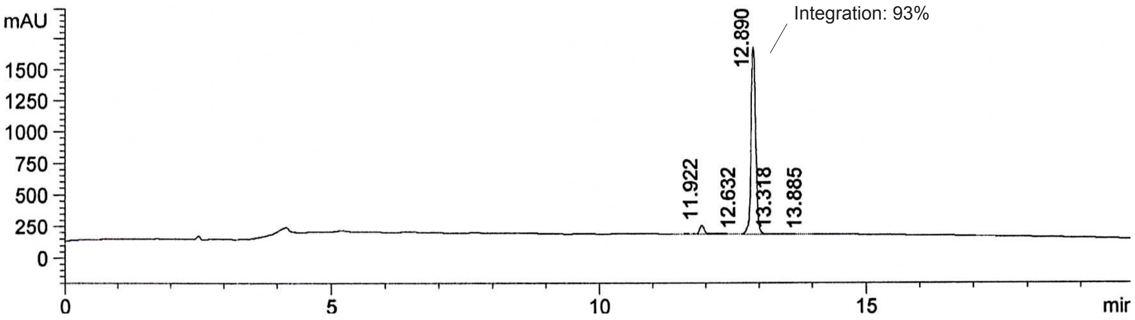

mass spectrum (ESI) of the purified peptide

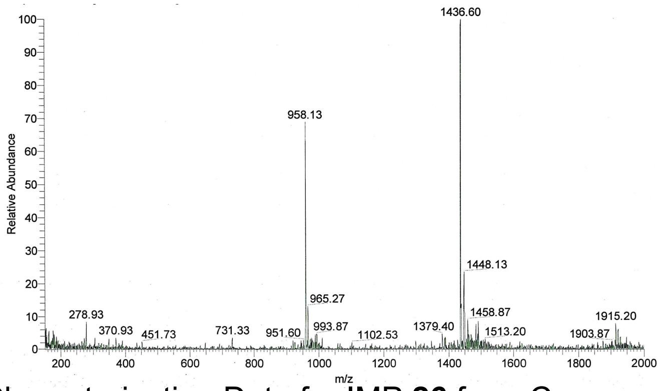

Characterization Data for IMP **26** from Commercial Vendor

RP-HPLC trace of the purified peptide

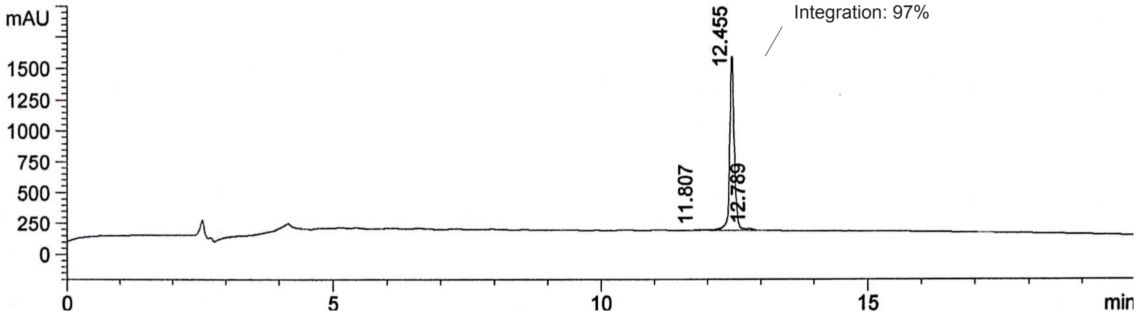

mass spectrum (ESI) of the purified peptide

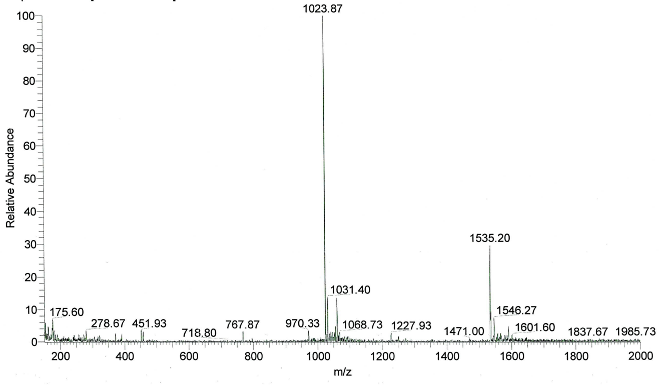

Characterization Data for IMP **27** from Commercial Vendor
RP-HPLC trace of the purified peptide

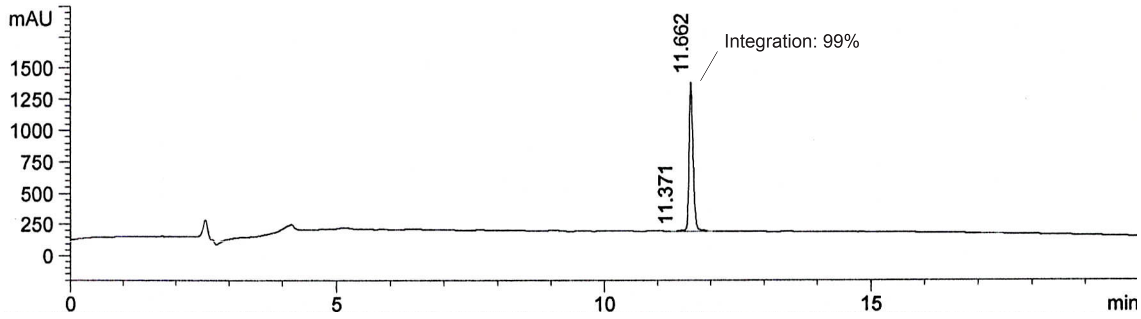

mass spectrum (ESI) of the purified peptide

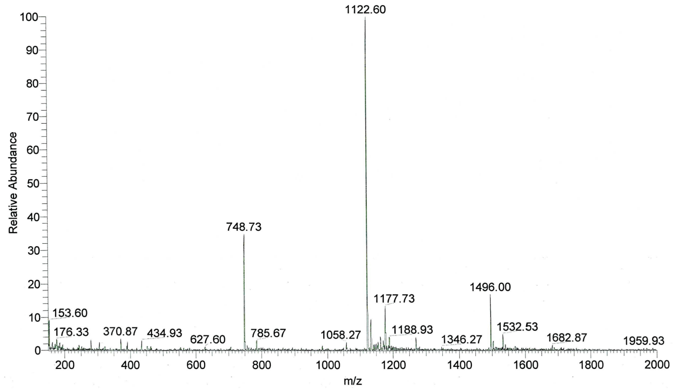

Characterization Data for IMP **28** from Commercial Vendor
RP-HPLC trace of the purified peptide

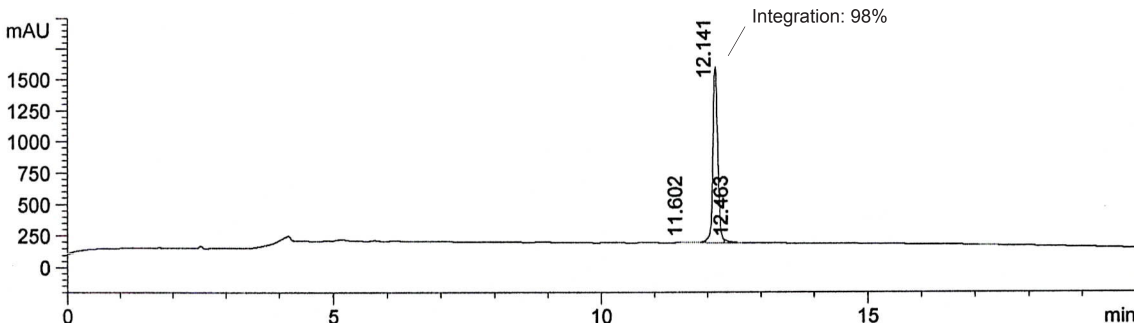

mass spectrum (ESI) of the purified peptide

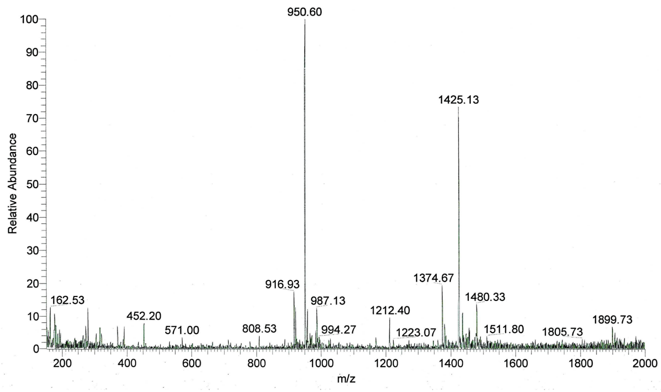

Characterization Data for IMP **29** from Commercial Vendor
RP-HPLC trace of the purified peptide

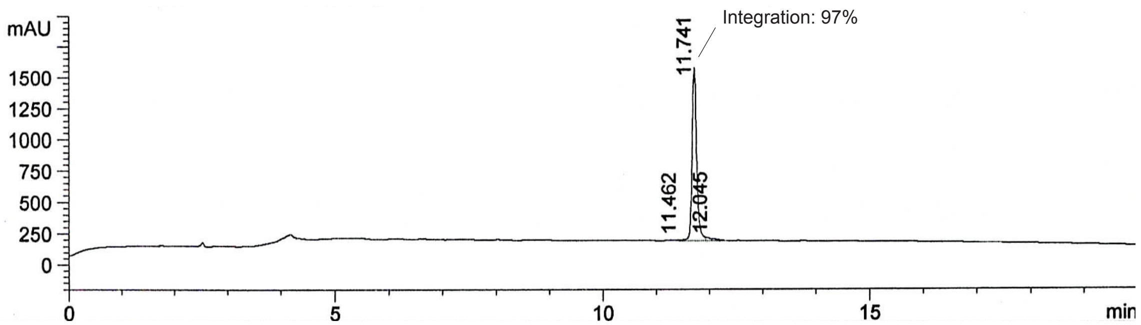

mass spectrum (ESI) of the purified peptide

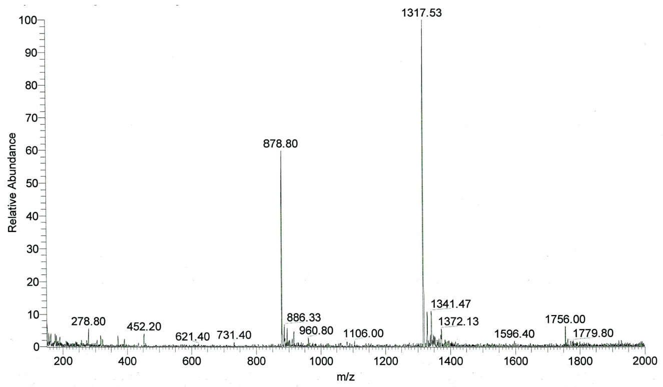

Characterization Data for ASP **01**

RP-HPLC trace of the purified peptide

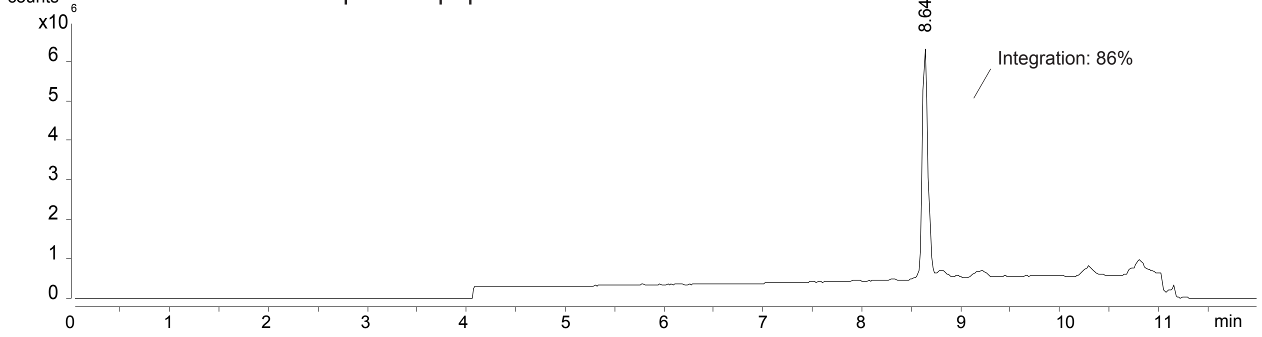

mass spectrum (ESI) of the purified peptide

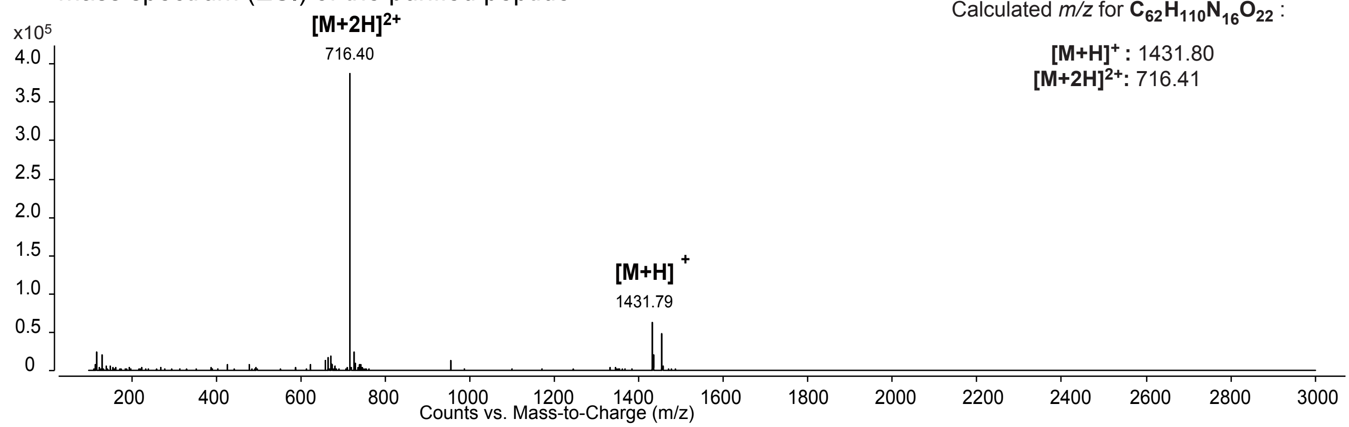

Characterization Data for ASP **02**

RP-HPLC trace of the purified peptide

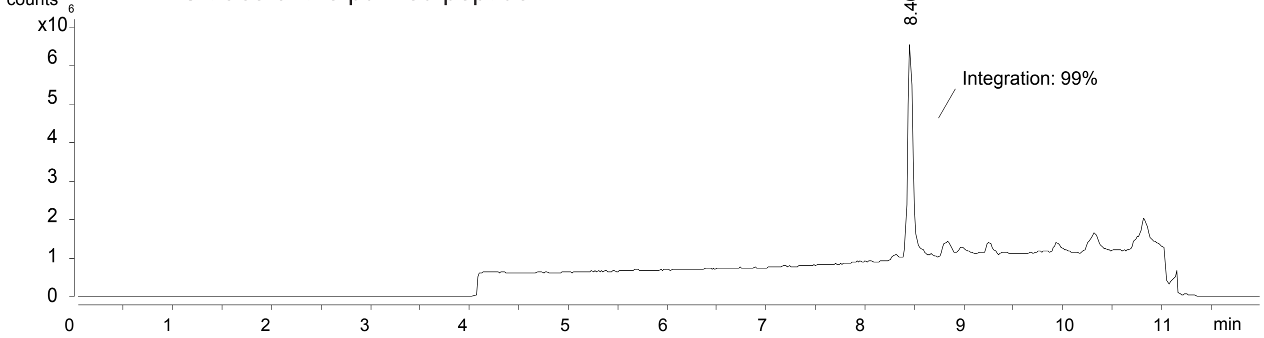

mass spectrum (ESI) of the purified peptide

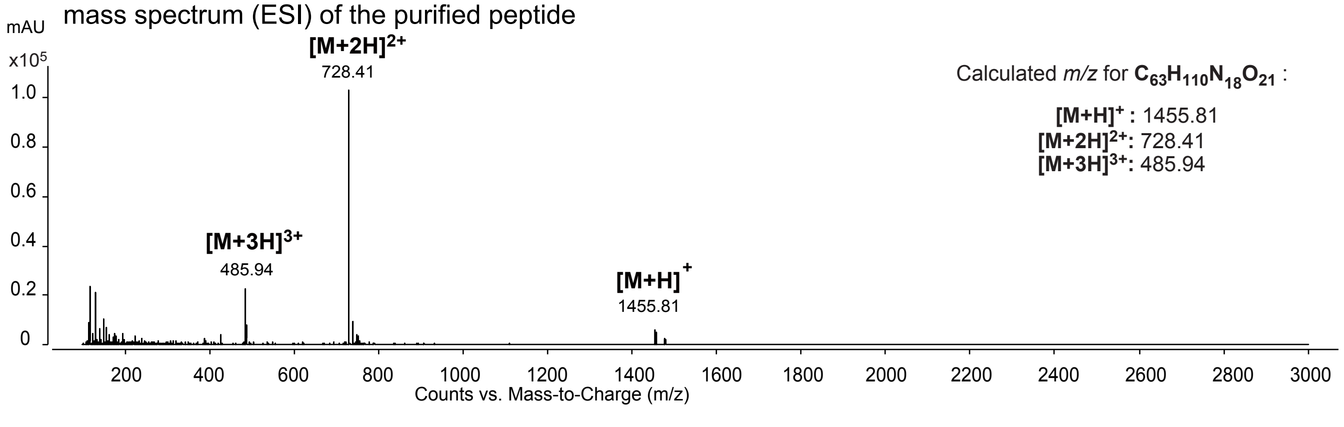

Characterization Data for ASP **03**

RP-HPLC trace of the purified peptide

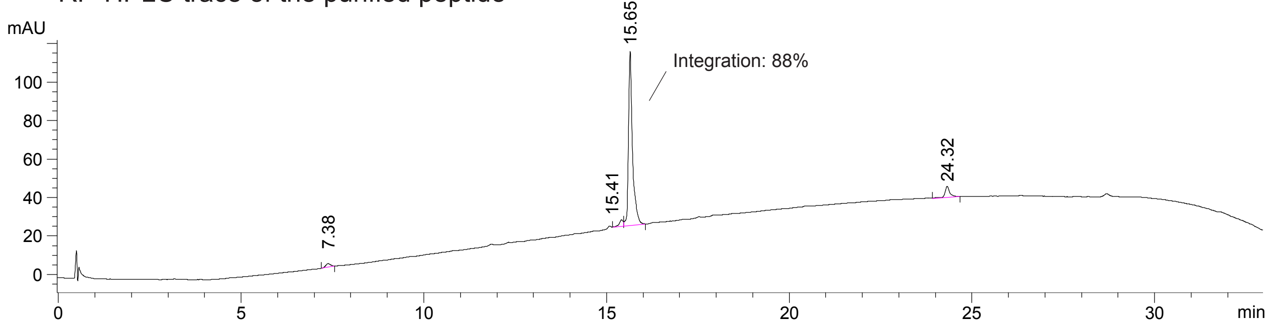

mass spectrum (ESI) of the purified peptide

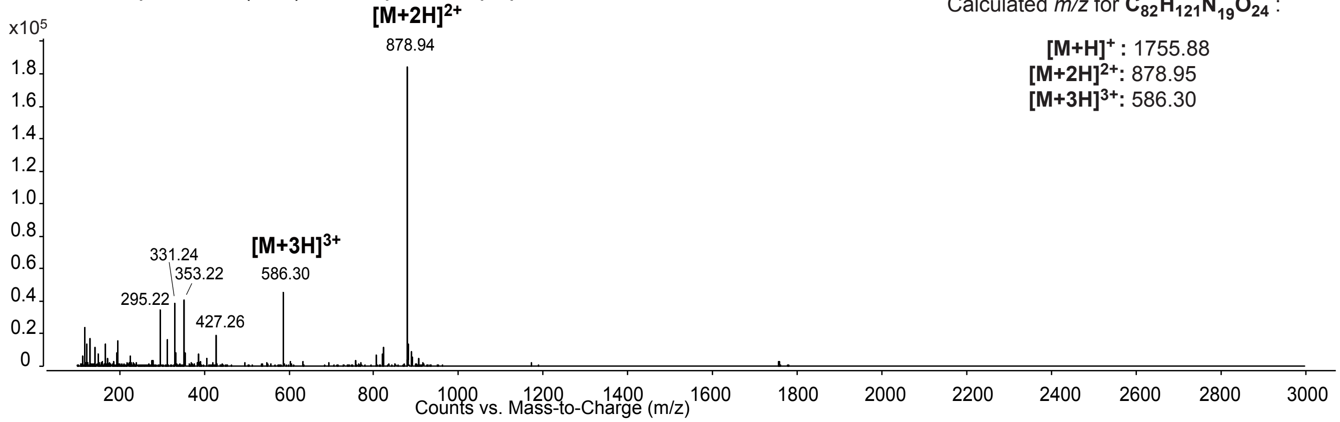

Calculated *m/z* for $C_{82}H_{121}N_{19}O_{24}$:

[M+H]$^+$: 1755.88
[M+2H]$^{2+}$: 878.95
[M+3H]$^{3+}$: 586.30

Characterization Data for ASP **04**

RP-HPLC trace of the purified peptide

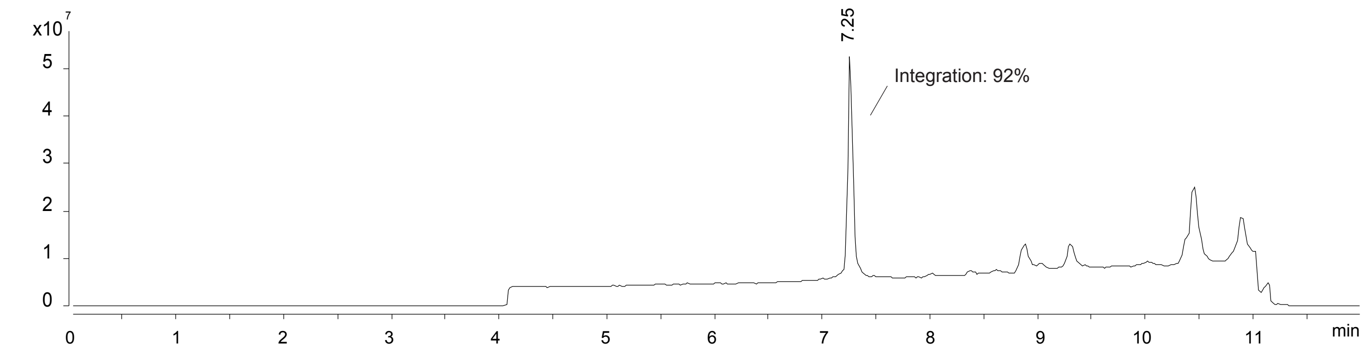

mass spectrum (ESI) of the purified peptide

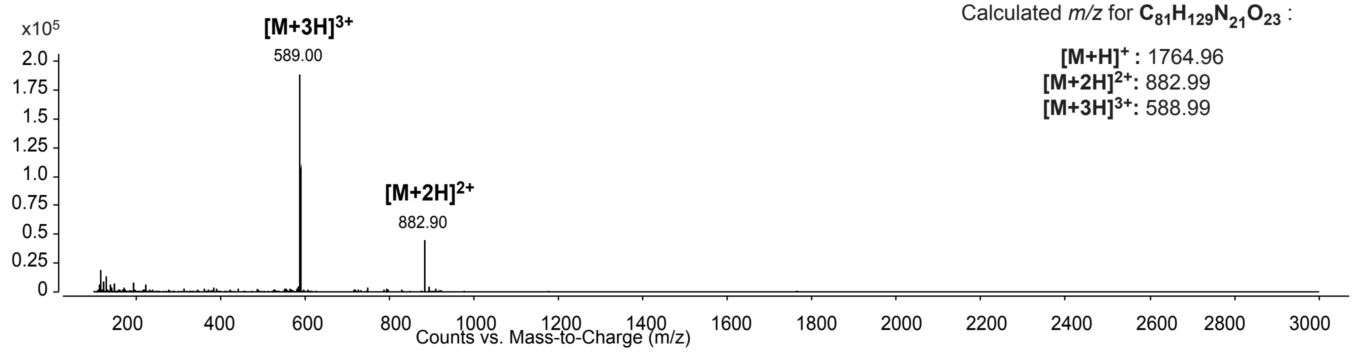

Calculated *m/z* for $C_{81}H_{129}N_{21}O_{23}$:

[M+H]$^+$: 1764.96
[M+2H]$^{2+}$: 882.99
[M+3H]$^{3+}$: 588.99

Characterization Data for ASP **05**

RP-HPLC trace of the purified peptide

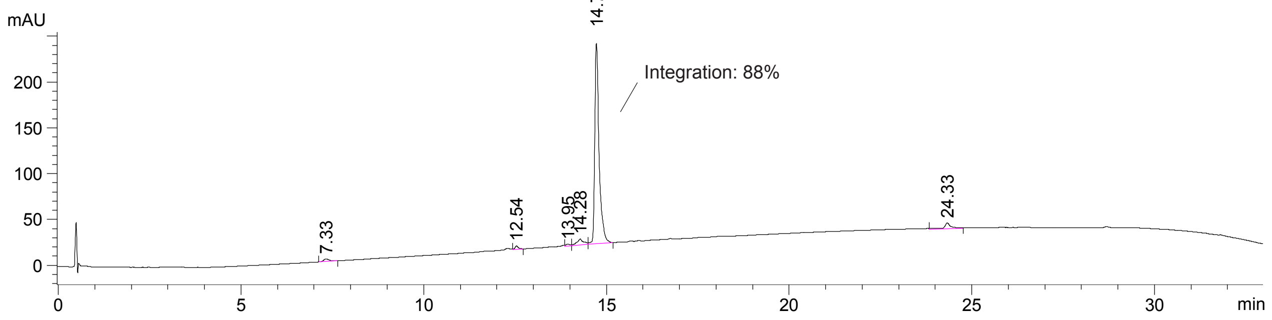

mass spectrum (ESI) of the purified peptide

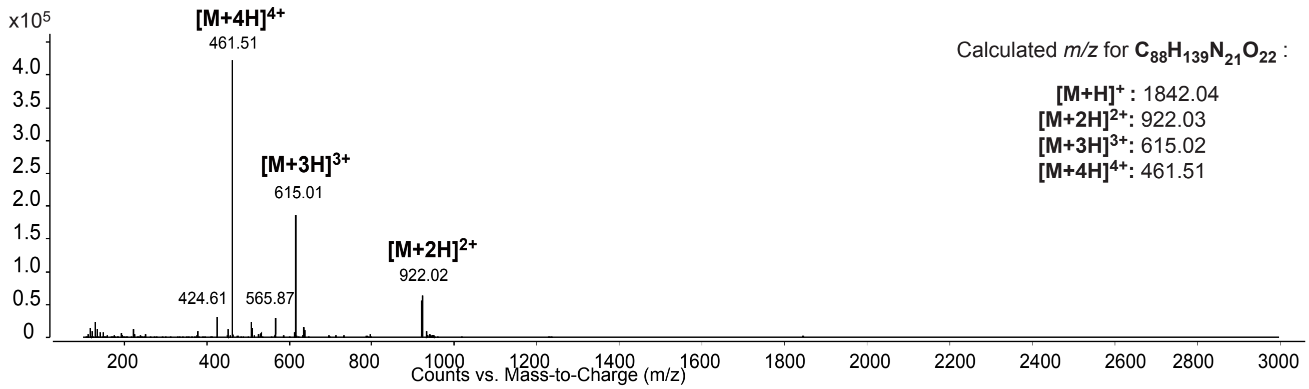

Characterization Data for ASP **06**

RP-HPLC trace of the purified peptide

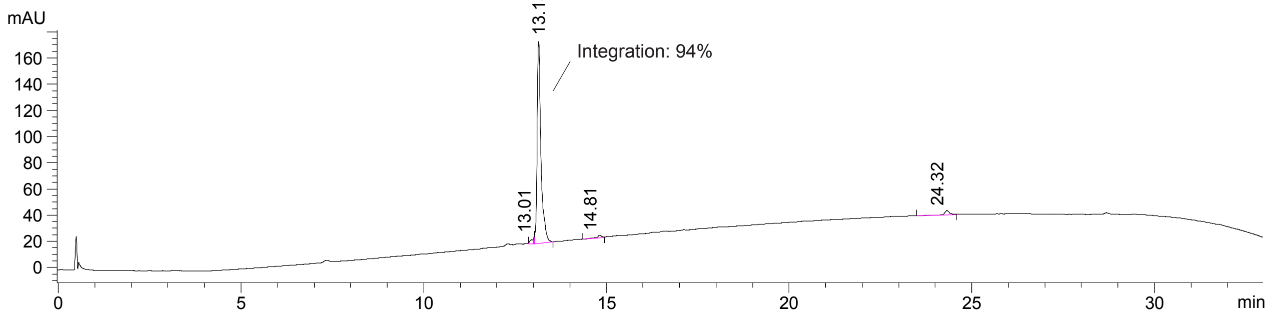

mass spectrum (ESI) of the purified peptide

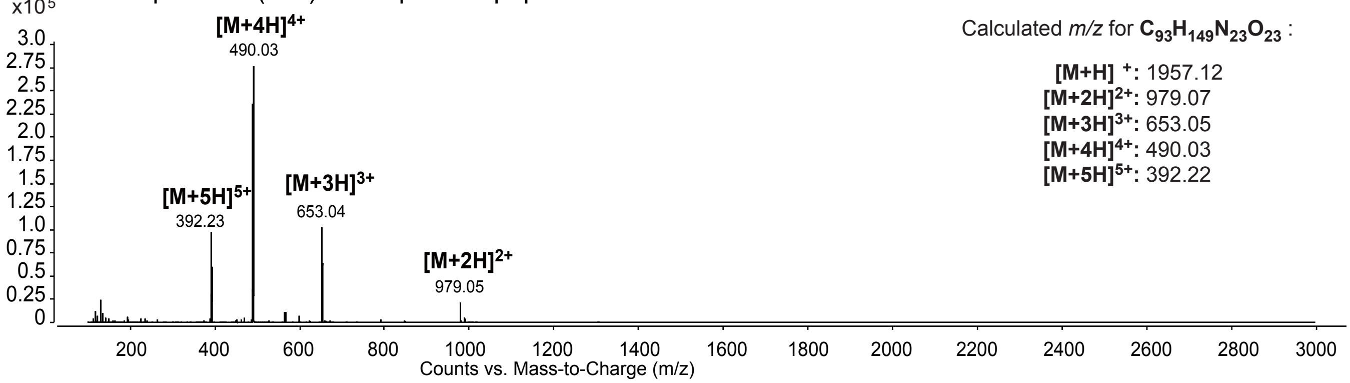

Characterization Data for ASP **07**

RP-HPLC trace of the purified peptide

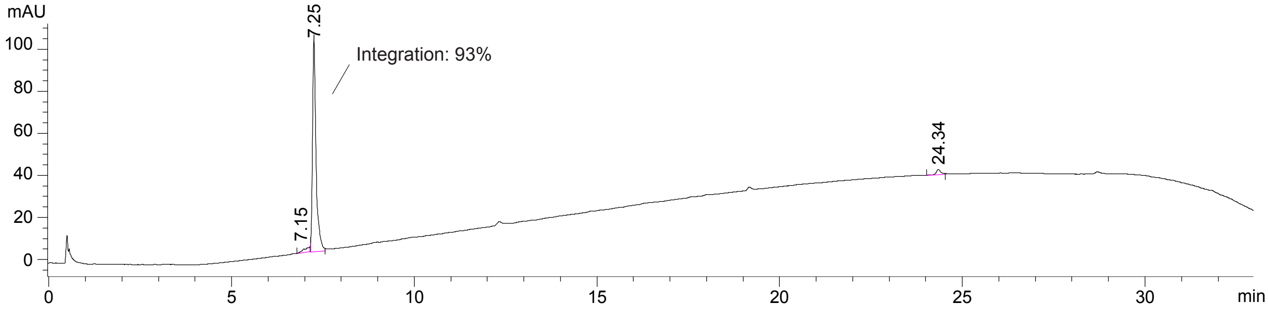

mass spectrum (ESI) of the purified peptide

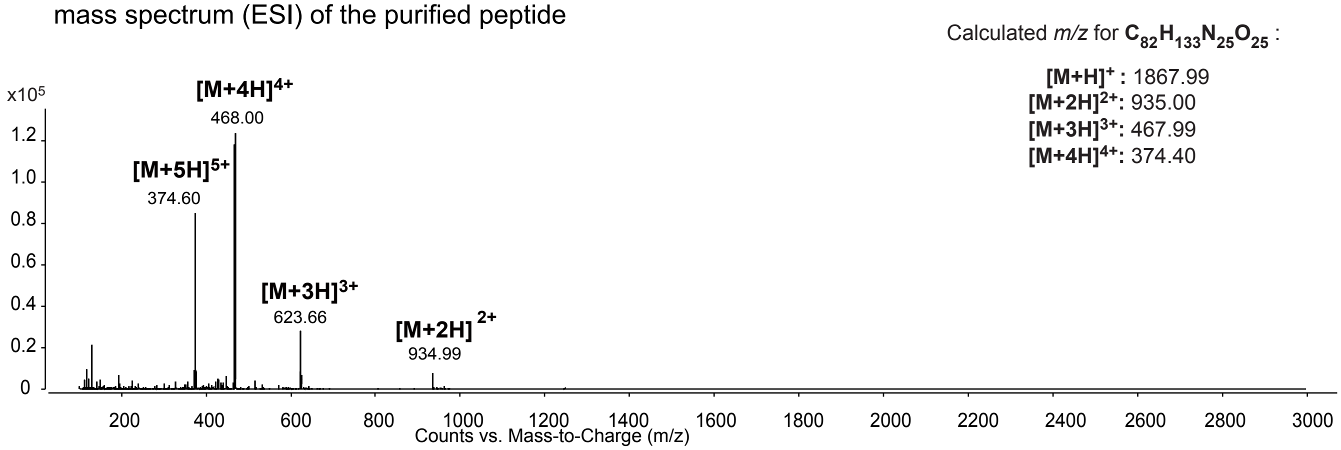

Characterization Data for ASP **08**

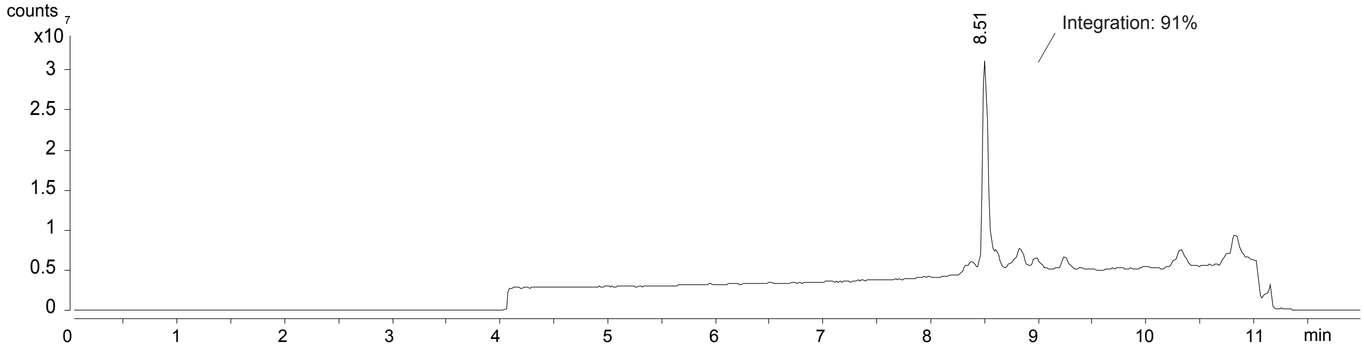

mass spectrum (ESI) of the purified peptide

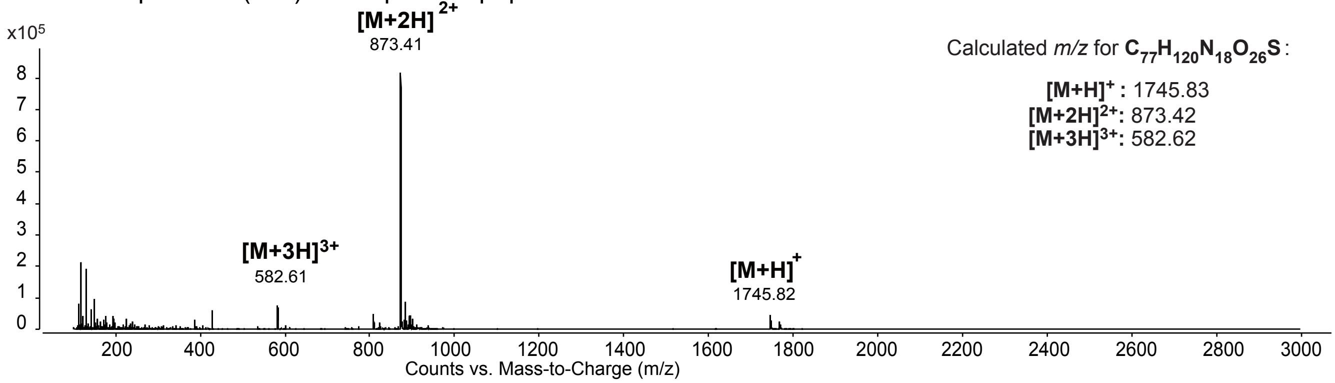

Characterization Data for ASP **09**

RP-HPLC trace of the purified peptide

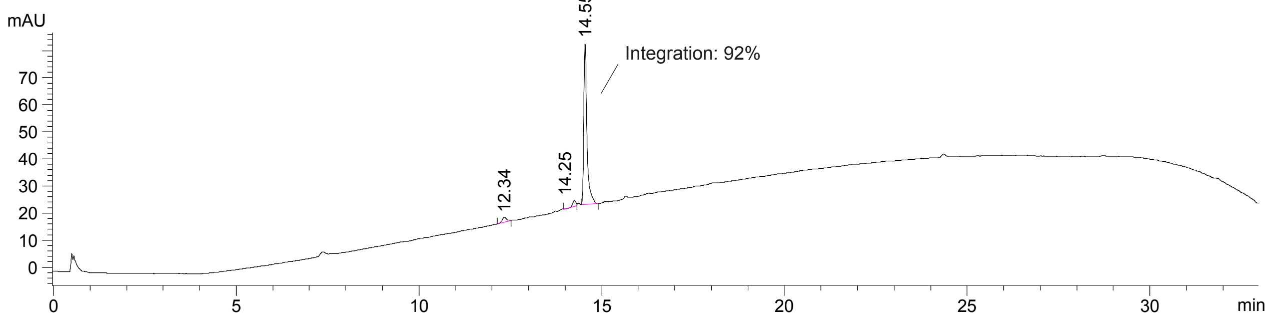

mass spectrum (ESI) of the purified peptide

Calculated *m/z* for $\mathbf{C_{84}H_{128}N_{20}O_{26}}$:

$[M+H]^+$: 1833.94
$[2M+3H]^{3+}$: 1222.96
$[M+2H]^{2+}$: 917.47
$[M+3H]^{3+}$: 611.98

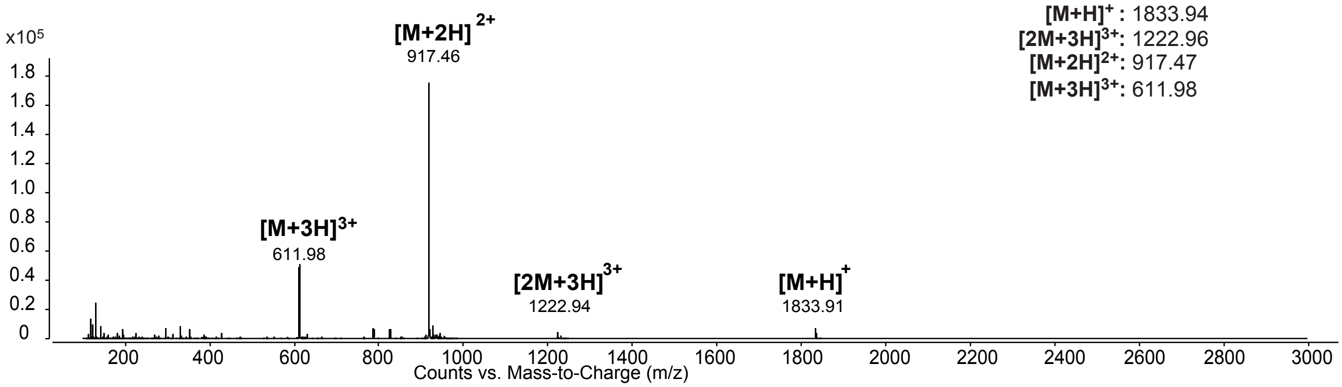

Characterization Data for ASP **10**

RP-HPLC trace of the purified peptide

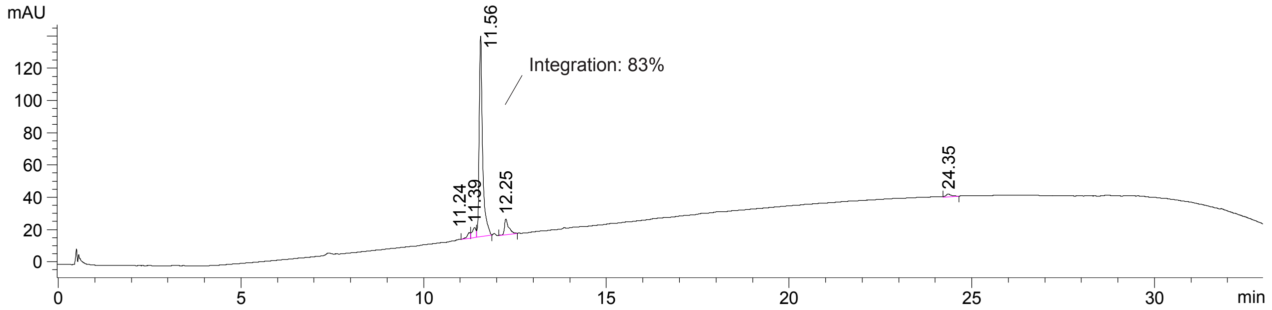

mass spectrum (ESI) of the purified peptide

Calculated *m/z* for $\mathbf{C_{78}H_{115}N_{21}O_{27}}$:

$[M+H]^+$: 1778.83
$[M+2H]^{2+}$: 889.92
$[M+3H]^{3+}$: 593.62

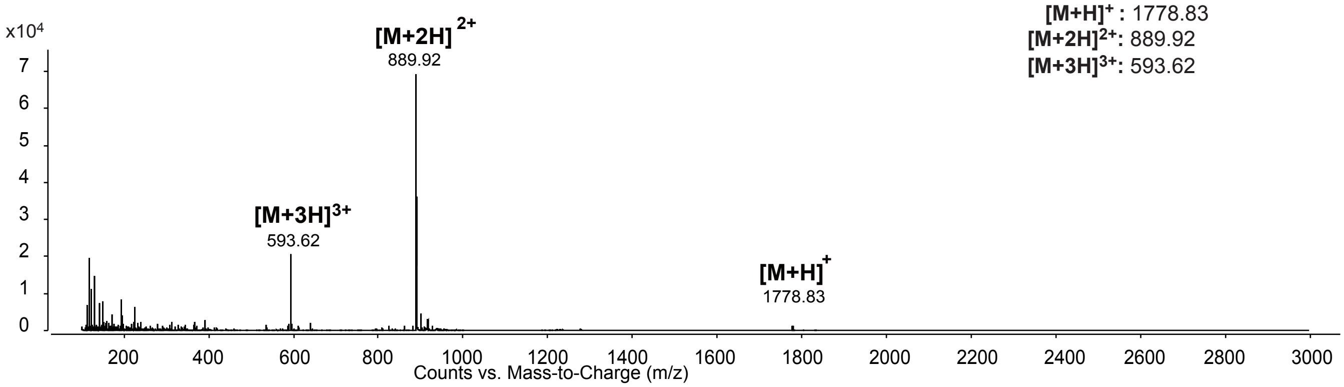

Characterization Data for ASP **11**

RP-HPLC trace of the purified peptide

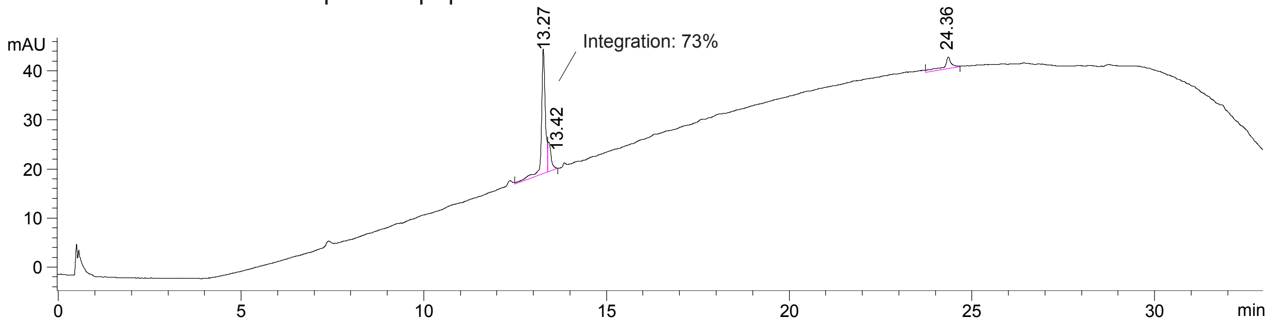

mass spectrum (ESI) of the purified peptide

Calculated *m/z* for $\mathbf{C_{73}H_{127}N_{19}O_{21}}$:

$[M+H]^+$: 1606.95
$[M+2H]^{2+}$: 803.98
$[M+3H]^{3+}$: 536.32

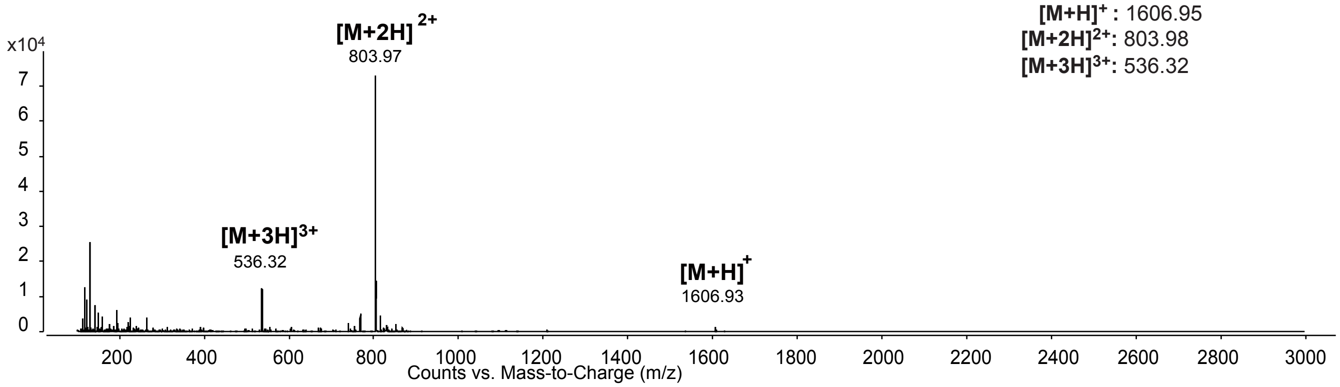

Characterization Data for ASP **12**

RP-HPLC trace of the purified peptide

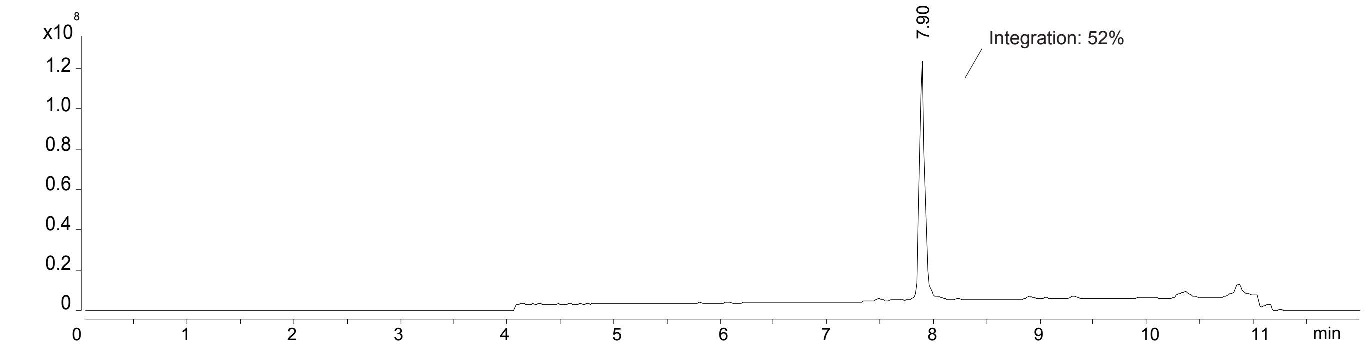

mass spectrum (ESI) of the purified peptide

Calculated *m/z* for $\mathbf{C_{78}H_{133}N_{21}O_{19}}$:

$[M+H]^+$: 1669.01
$[M+2H]^{2+}$: 835.01
$[M+3H]^{3+}$: 557.01

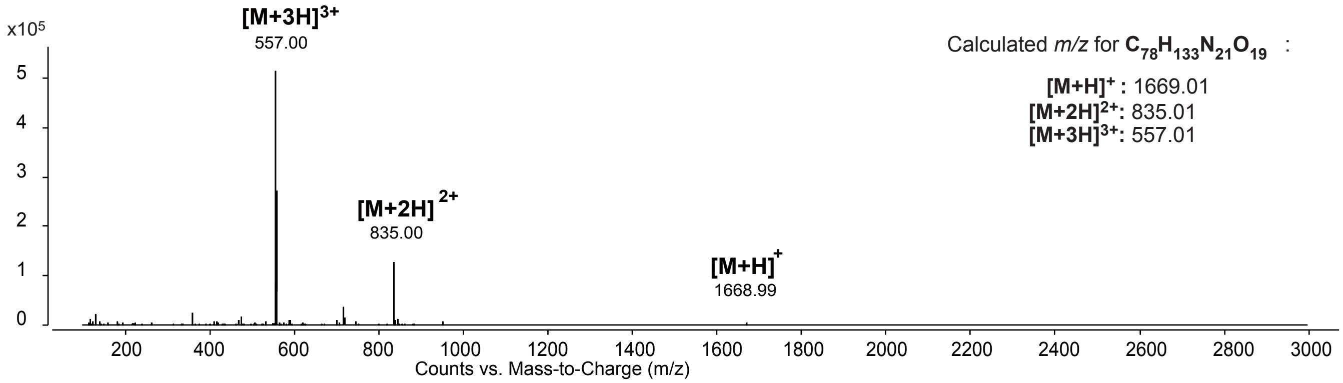

Characterization Data for ASP **15**

RP-HPLC trace of the purified peptide

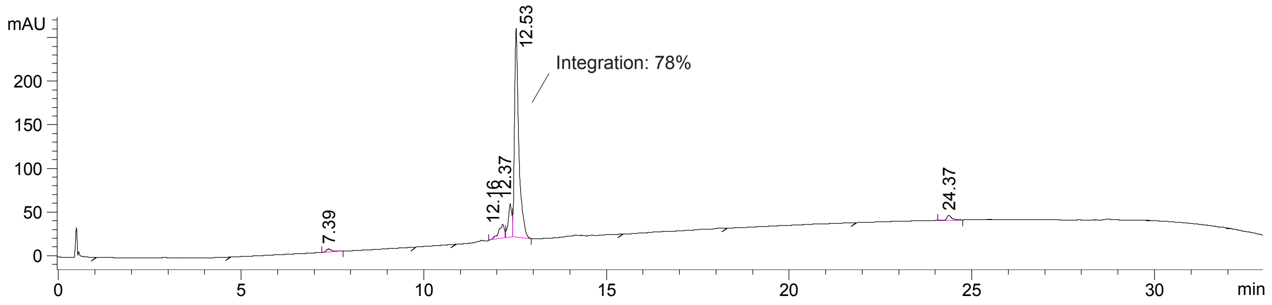

mass spectrum (ESI) of the purified peptide

Calculated *m/z* for $\mathbf{C_{87}H_{129}N_{21}O_{23}}$:

[M+H]$^{+}$: 1836.95
[M+2H]$^{2+}$: 918.99
[M+3H]$^{3+}$: 612.99
[M+4H]$^{4+}$: 459.99

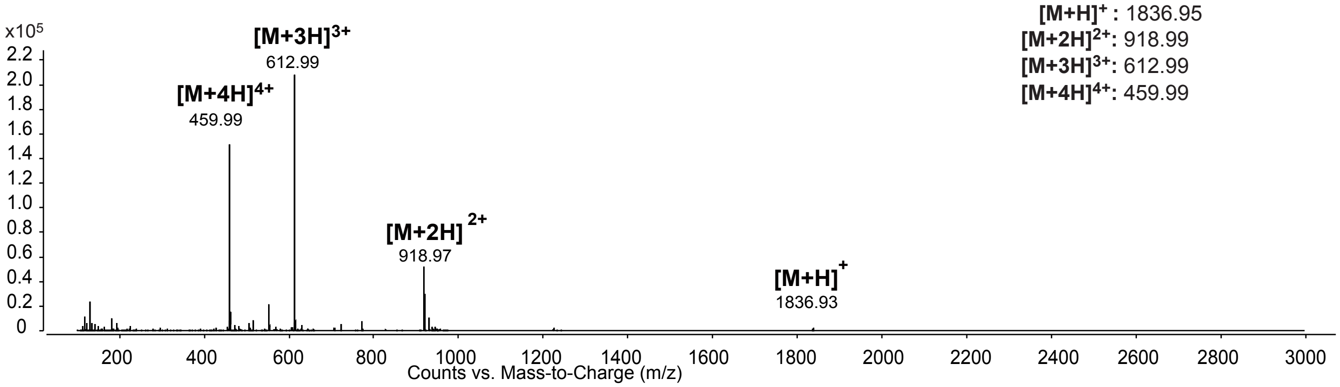

Characterization Data for ASP **16**

RP-HPLC trace of the purified peptide

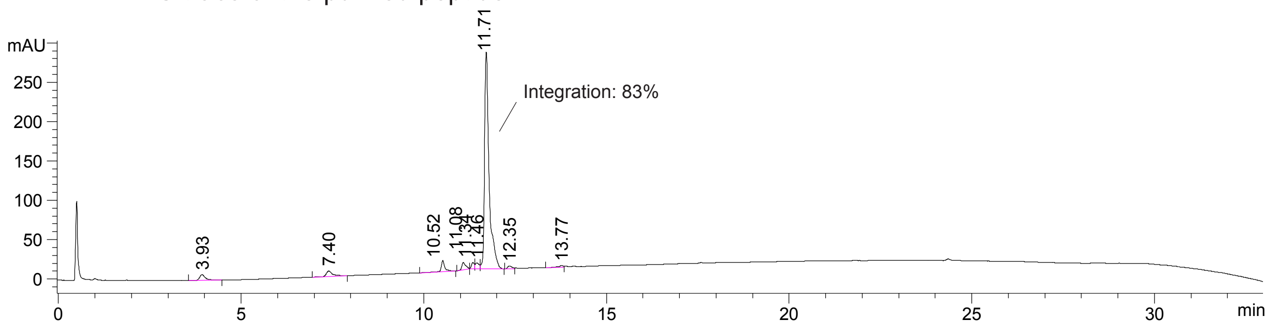

mass spectrum (ESI) of the purified peptide

Calculated *m/z* for $\mathbf{C_{79}H_{121}N_{21}O_{21}S_{1}}$:

[M+H]$^{+}$: 1732.88
[M+2H]$^{2+}$: 866.95
[M+3H]$^{3+}$: 578.30
[M+4H]$^{4+}$: 433.98

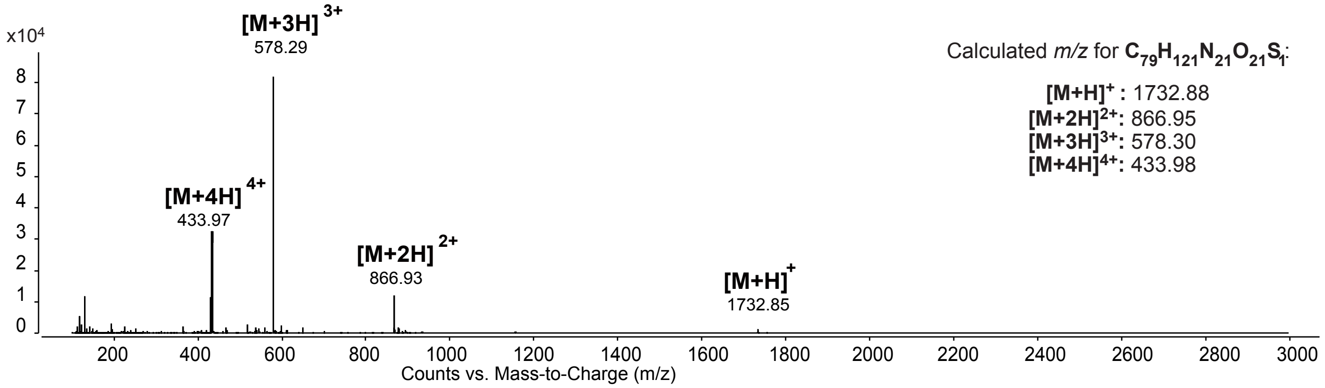

Characterization Data for ASP **17**

RP-HPLC trace of the purified peptide

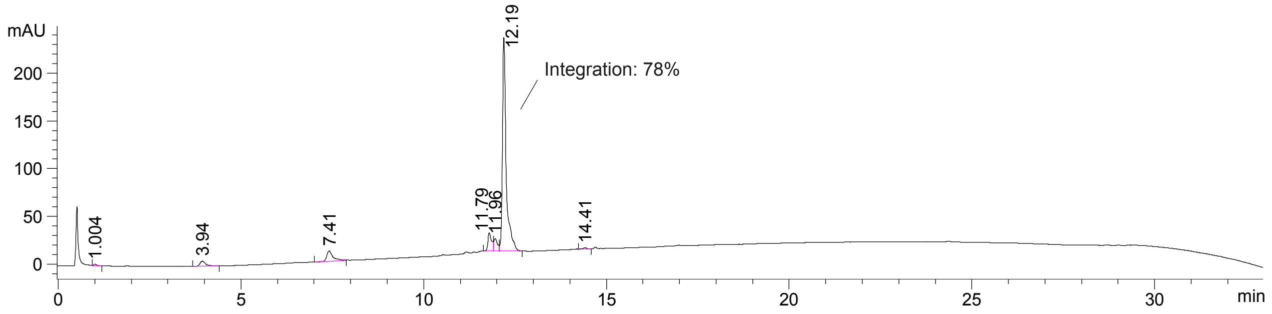

Calculated *m/z* for $C_{85}H_{128}N_{20}O_{26}$:

[M+H]$^+$: 1845.93
[2M+3H]$^{3+}$: 1230.96
[M+2H]$^{2+}$: 923.47
[M+3H]$^{3+}$: 615.98
[M+4H]$^{4+}$: 462.23

mass spectrum (ESI) of the purified peptide

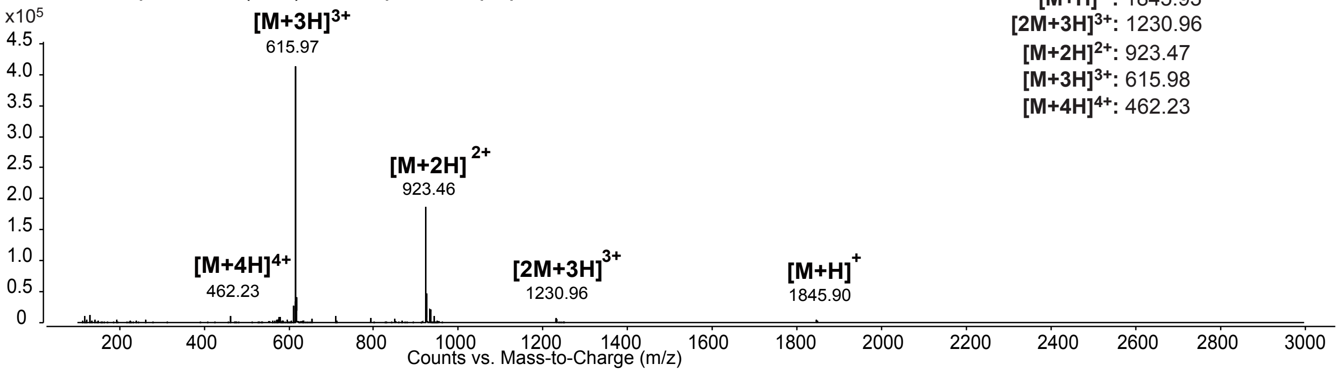

Characterization Data for ASP **18**

RP-HPLC trace of the purified peptide

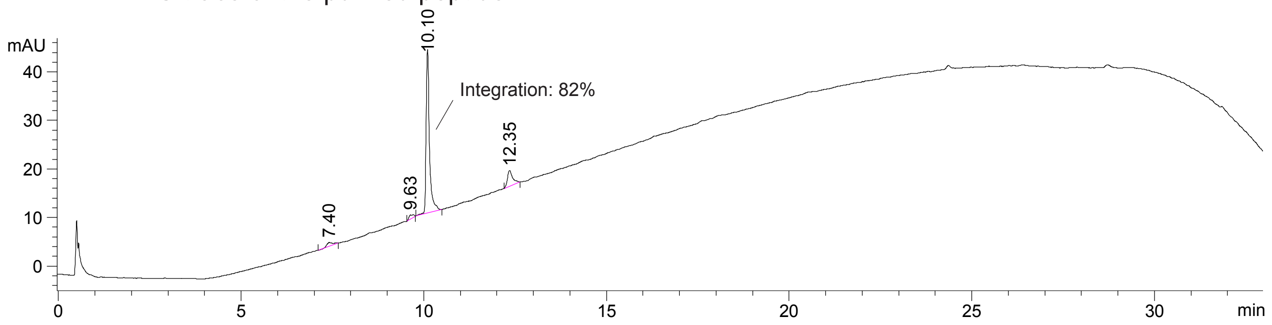

Calculated *m/z* for $C_{71}H_{122}N_{22}O_{24}$:

[M+H]$^+$: 1667.90
[M+2H]$^{2+}$: 834.46
[M+3H]$^{3+}$: 556.64

mass spectrum (ESI) of the purified peptide

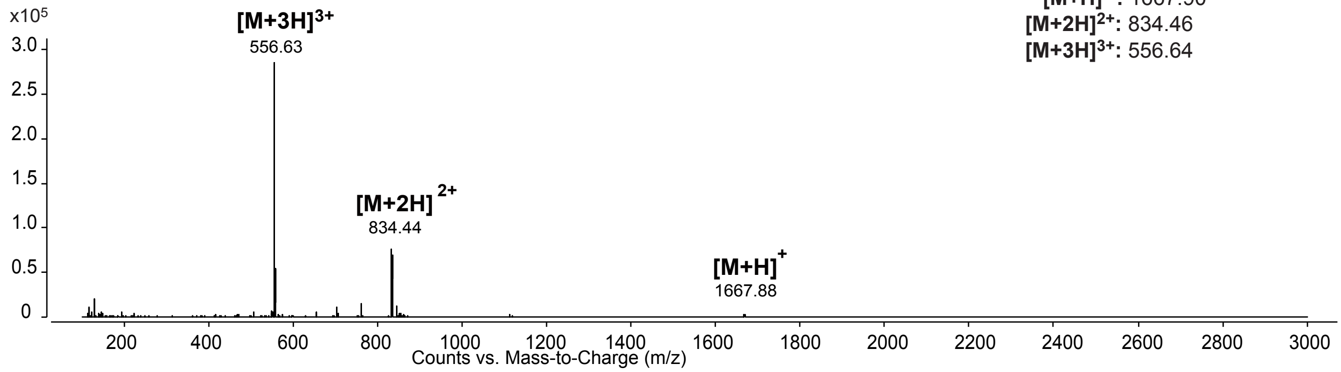

Characterization Data for ASP **19**

RP-HPLC trace of the purified peptide

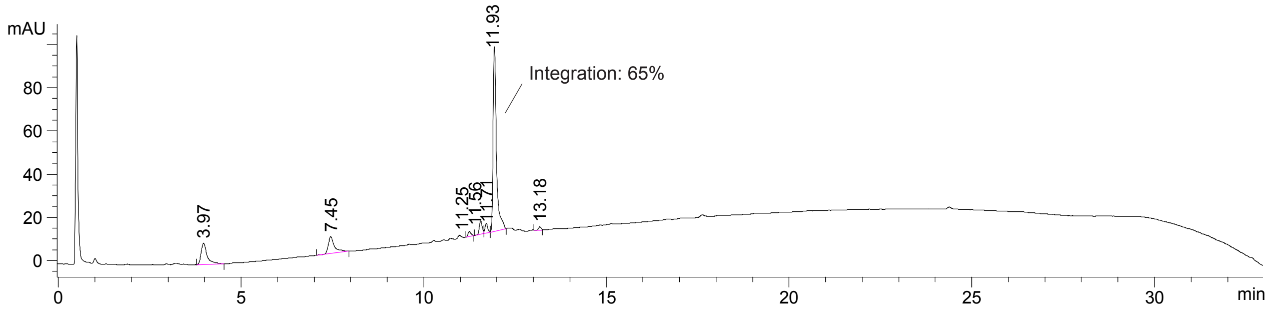

mass spectrum (ESI) of the purified peptide

Calculated *m/z* for $\mathbf{C_{71}H_{119}N_{19}O_{24}}$:

$[M+H]^{+}$: 1622.87
$[M+2H]^{2+}$: 811.94
$[M+3H]^{3+}$: 541.63

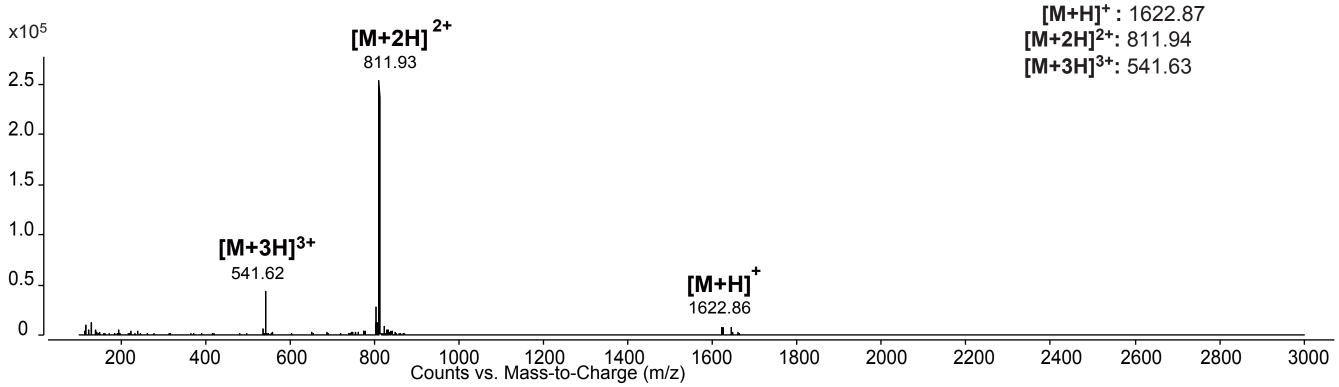

Characterization Data for ASP **20**

RP-HPLC trace of the purified peptide

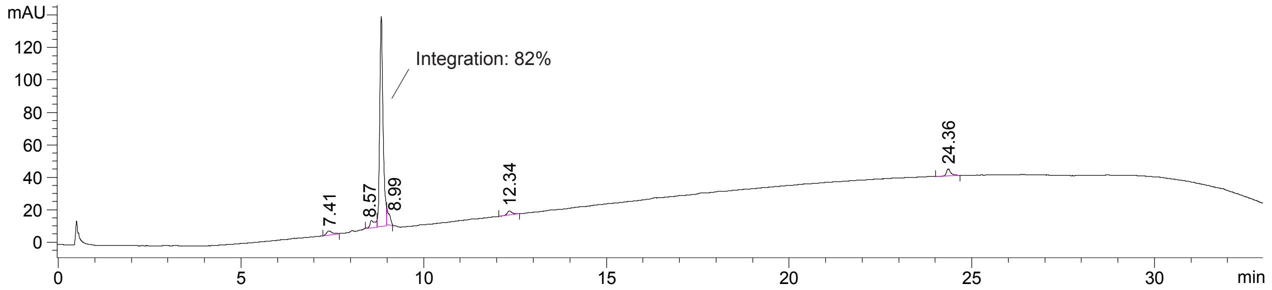

mass spectrum (ESI) of the purified peptide

Calculated *m/z* for $\mathbf{C_{63}H_{105}N_{17}O_{28}}$:

$[M+H]^{+}$: 1548.73
$[M+2H]^{2+}$: 774.87
$[M+3H]^{3+}$: 516.92

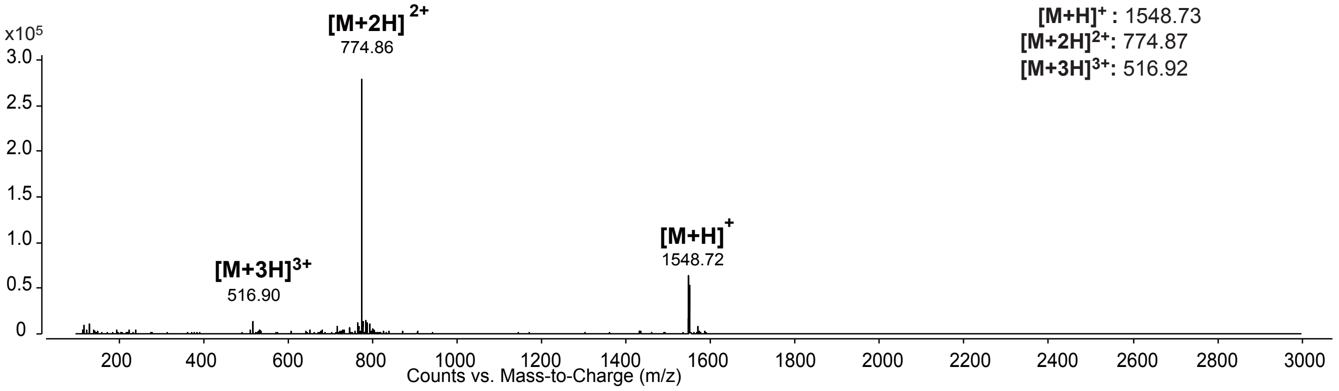

Characterization Data for ASP **21**

RP-HPLC trace of the purified peptide

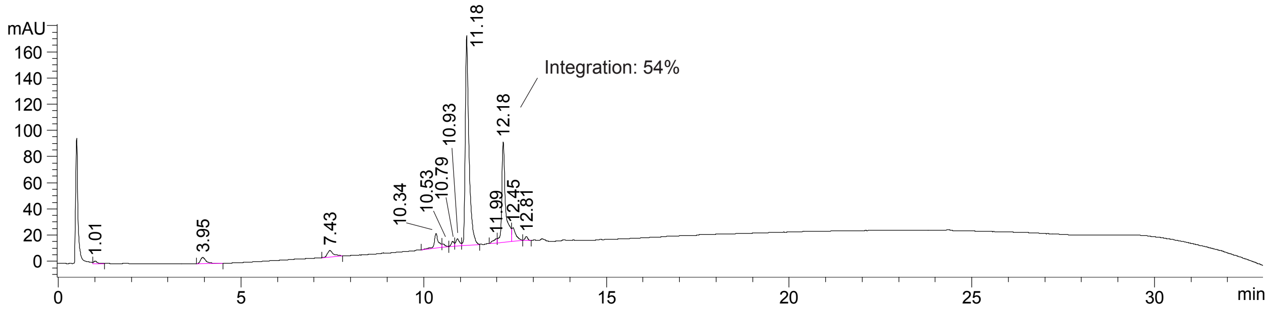

mass spectrum (ESI) of the purified peptide

Calculated *m/z* for $C_{74}H_{120}N_{22}O_{21}S_1$:

[M+H]$^+$: 1685.87
[M+2H]$^{2+}$: 843.44
[M+3H]$^{3+}$: 562.63
[M+4H]$^{4+}$: 422.22

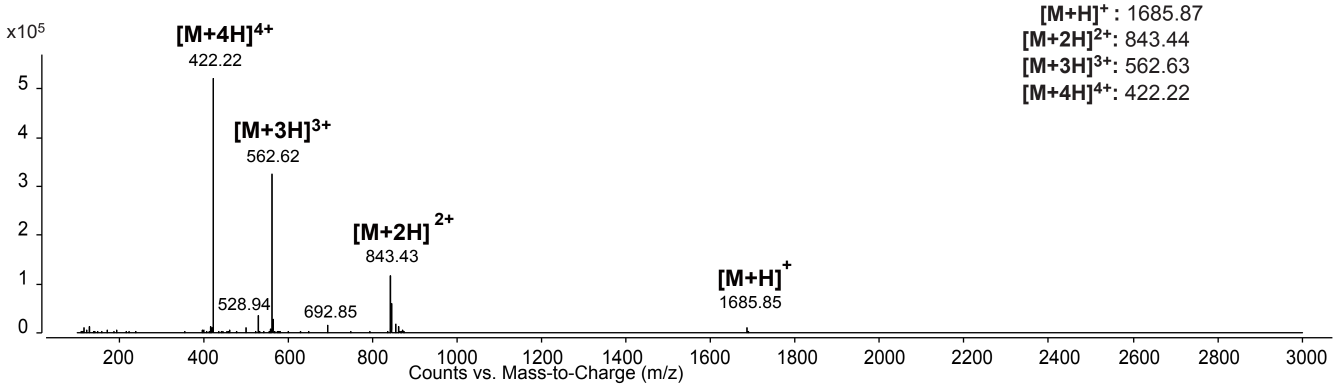

Characterization Data for ASP **22**

RP-HPLC trace of the purified peptide

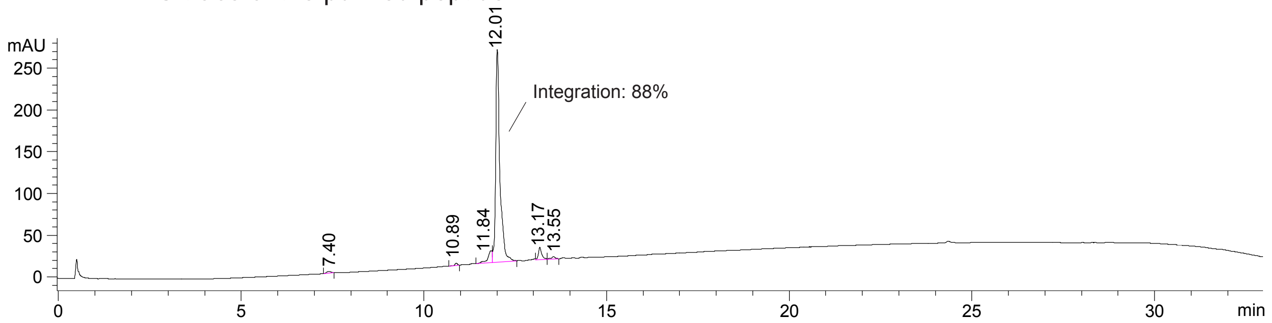

mass spectrum (ESI) of the purified peptide

Calculated *m/z* for $C_{85}H_{133}N_{29}O_{18}S_1$:

[M+H]$^+$: 1836.95
[M+2H]$^{2+}$: 941.01
[M+3H]$^{3+}$: 627.68
[M+4H]$^{4+}$: 471.01
[M+5H]$^{5+}$: 377.01

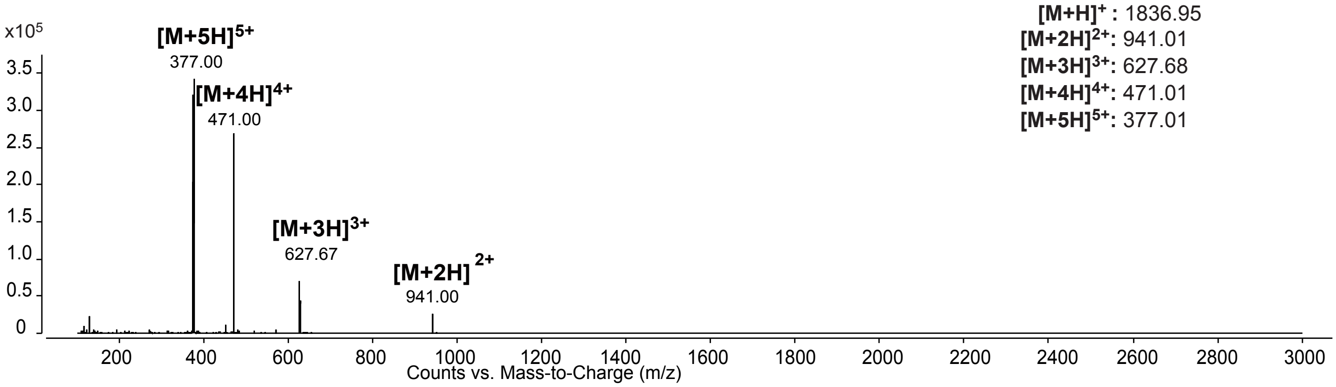

Characterization Data for ASP **23**

RP-HPLC trace of the purified peptide

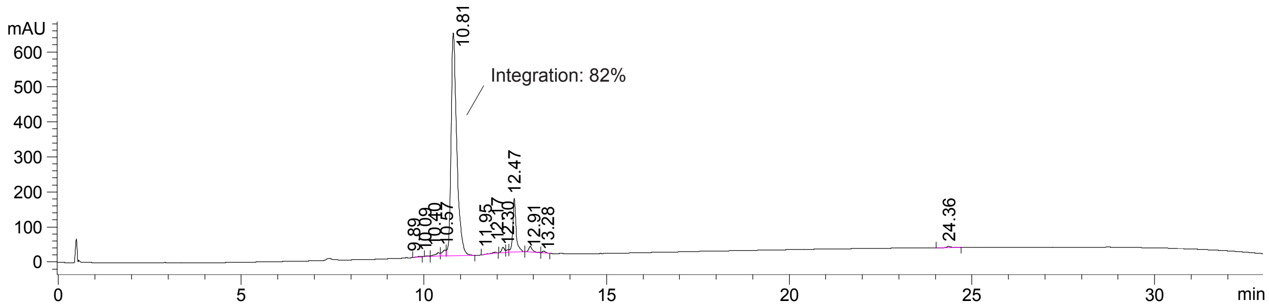

mass spectrum (ESI) of the purified peptide

Calculated *m/z* for $C_{81}H_{136}N_{30}O_{17}S_1$:

[M+H]$^+$: 1834.04
[M+2H]$^{2+}$: 917.51
[M+3H]$^{3+}$: 612.02
[M+4H]$^{4+}$: 459.27
[M+5H]$^{5+}$: 367.61
[M+6H]$^{6+}$: 306.51

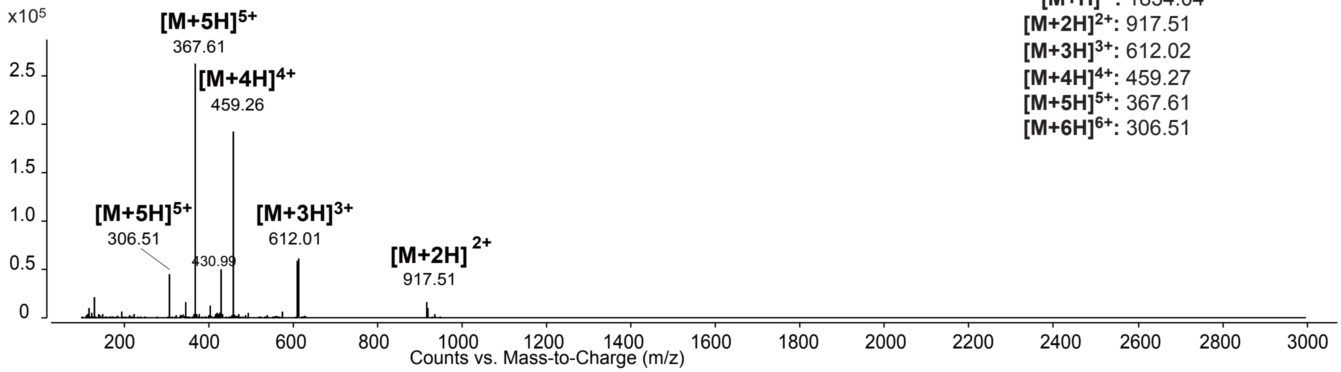

Characterization Data for ASP **24**

RP-HPLC trace of the purified peptide

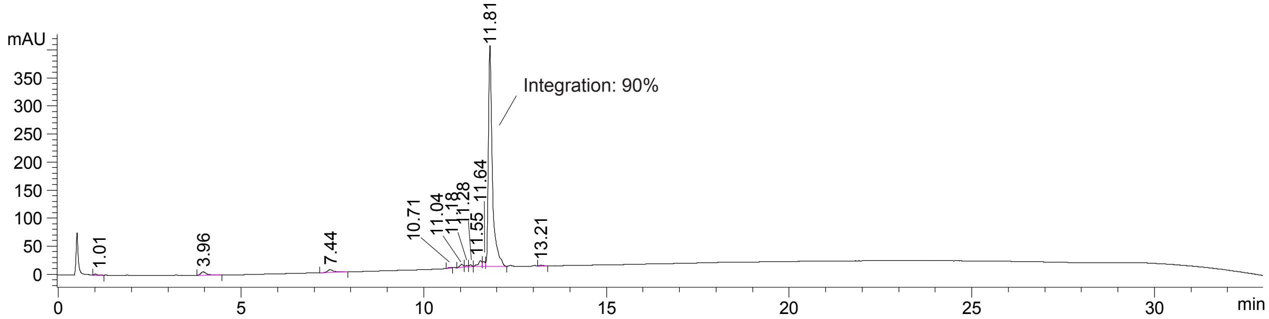

mass spectrum (ESI) of the purified peptide

Calculated *m/z* for $C_{91}H_{134}N_{26}O_{20}$:

[M+H]$^+$: 1912.03
[M+2H]$^{2+}$: 956.52
[M+3H]$^{3+}$: 638.02
[M+4H]$^{4+}$: 478.77
[M+5H]$^{5+}$: 383.21

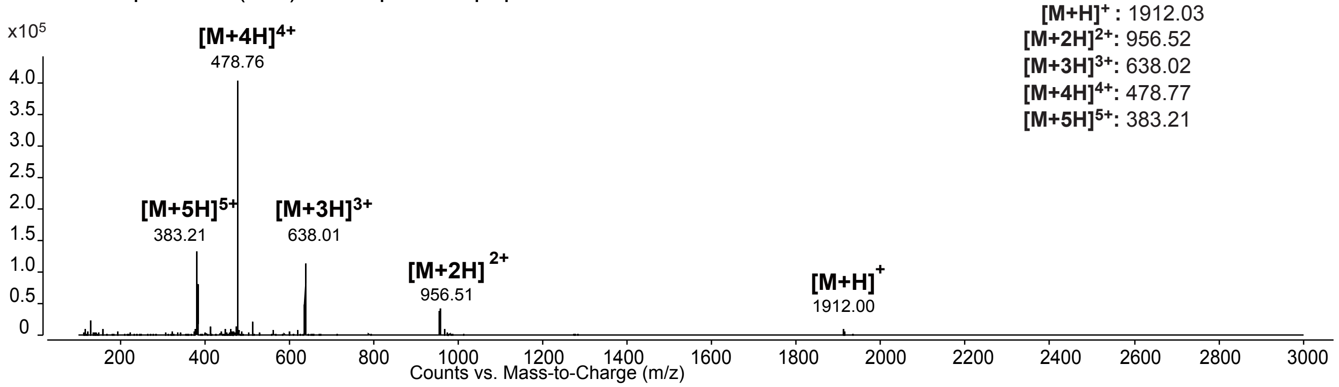

Characterization Data for ASP **25**

RP-HPLC trace of the purified peptide

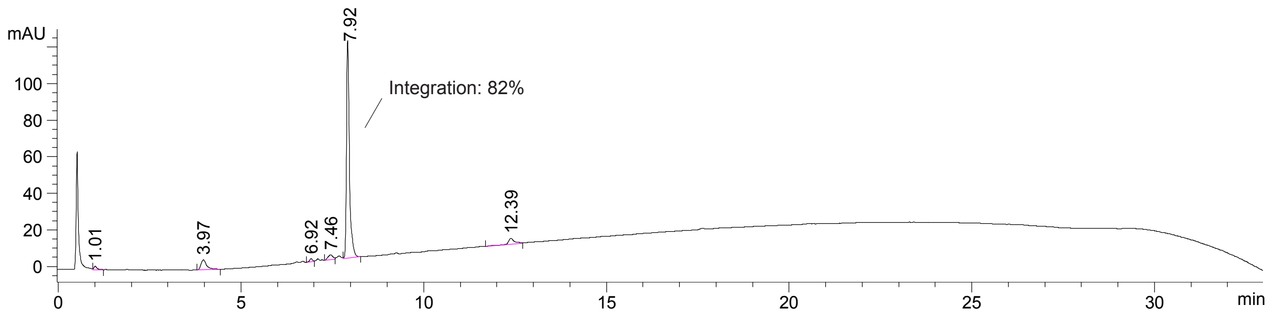

mass spectrum (ESI) of the purified peptide

Calculated *m/z* for $C_{86}H_{148}N_{32}O_{20}$:

[M+H]$^{+}$: 1949.15
[M+2H]$^{2+}$: 975.59
[M+3H]$^{3+}$: 650.73
[M+4H]$^{4+}$: 488.29
[M+5H]$^{5+}$: 390.83
[M+6H]$^{6+}$: 325.86

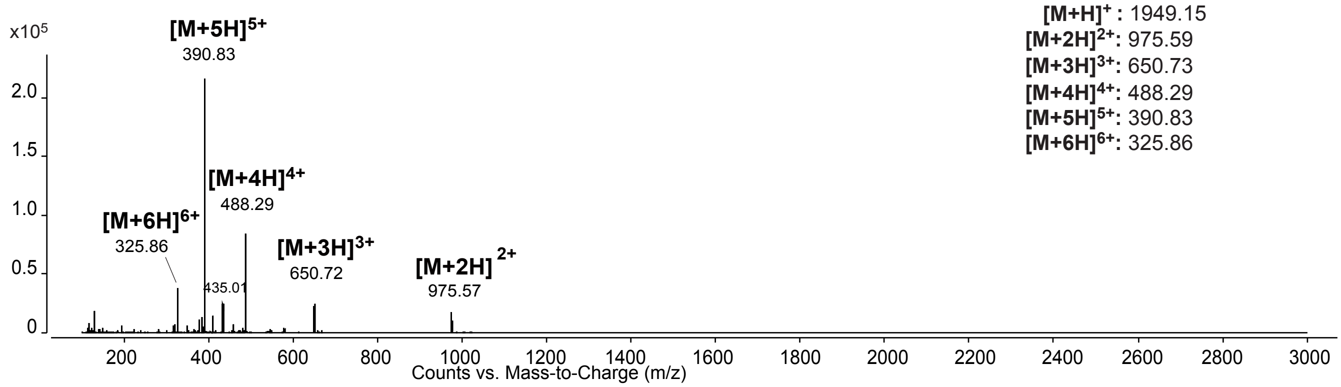

Characterization Data for ASP **26**

RP-HPLC trace of the purified peptide

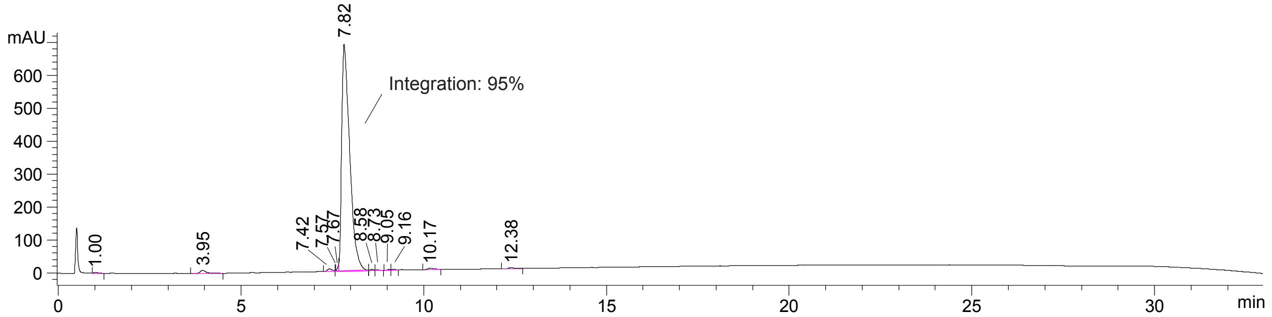

mass spectrum (ESI) of the purified peptide

Calculated *m/z* for $C_{80}H_{130}N_{26}O_{20}$:

[M+H]$^{+}$: 1776.00
[M+2H]$^{2+}$: 888.51
[M+3H]$^{3+}$: 592.67
[M+4H]$^{4+}$: 444.75
[M+5H]$^{5+}$: 356.00

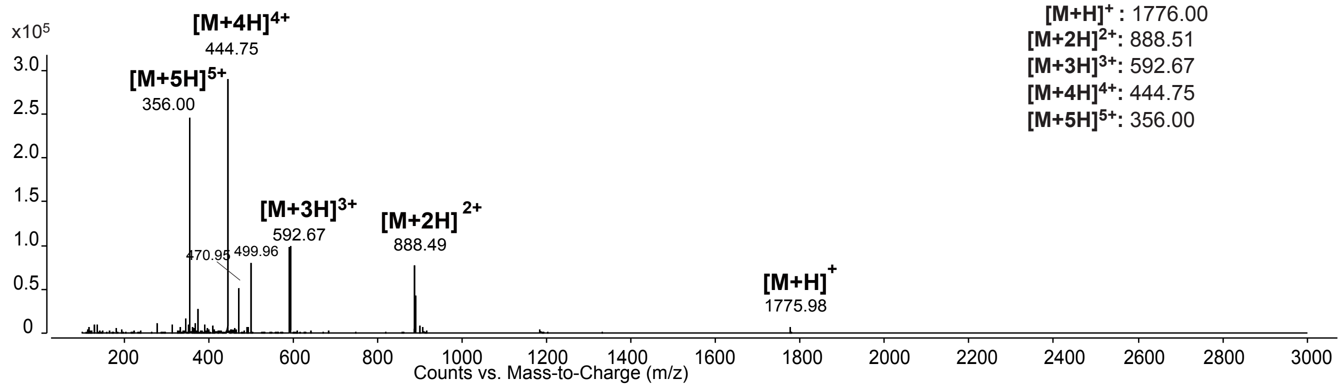

Characterization Data for ASP **27**

RP-HPLC trace of the purified peptide

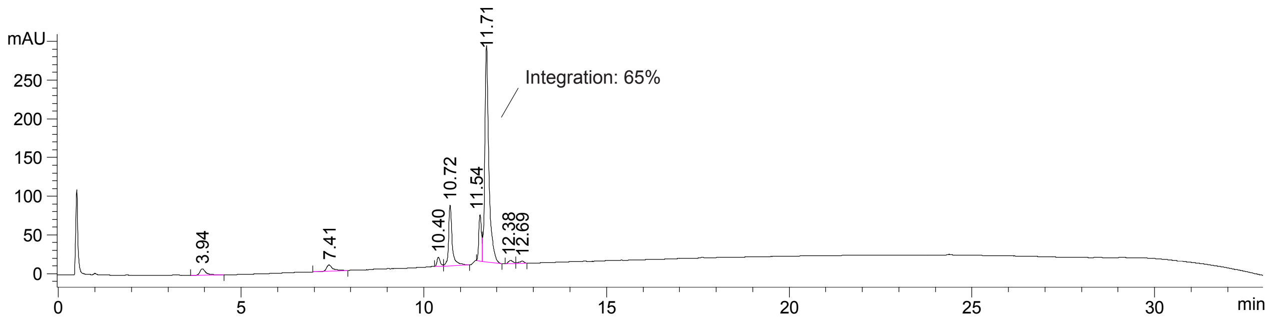

mass spectrum (ESI) of the purified peptide

Calculated *m/z* for $C_{82}H_{120}N_{18}O_{22}S_1$:

[M+H]⁺ : 1741.85
$[M+2H]^{2+}$: 871.44
$[M+3H]^{3+}$: 581.29

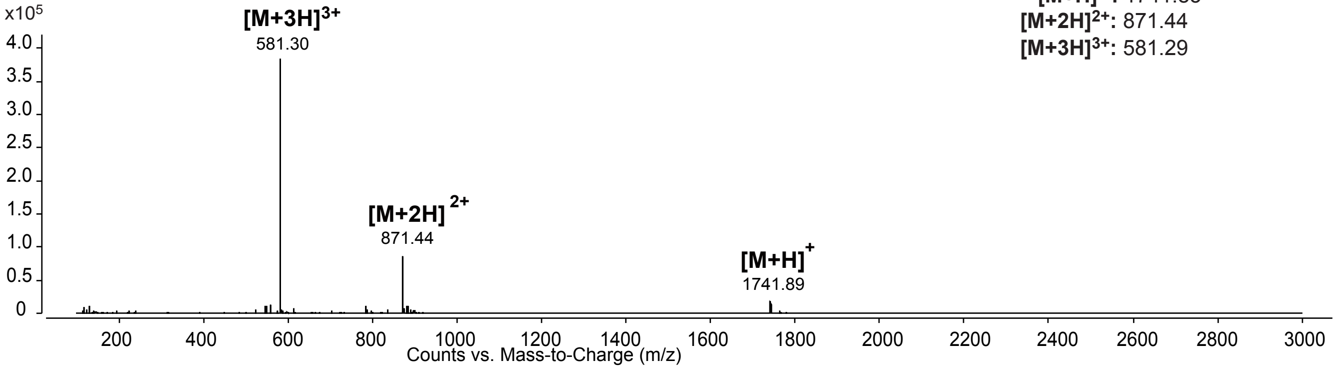

Characterization Data for ASP **28**

RP-HPLC trace of the purified peptide

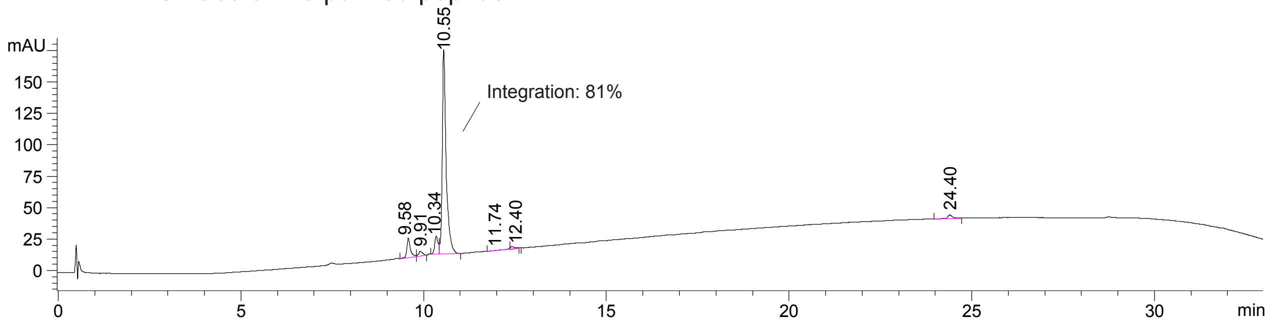

mass spectrum (ESI) of the purified peptide

Calculated *m/z* for $C_{79}H_{118}N_{18}O_{23}S_1$:

$[M+H]^+$: 1719.83
$[M+2H]^{2+}$: 860.42
$[M+3H]^{3+}$: 573.95

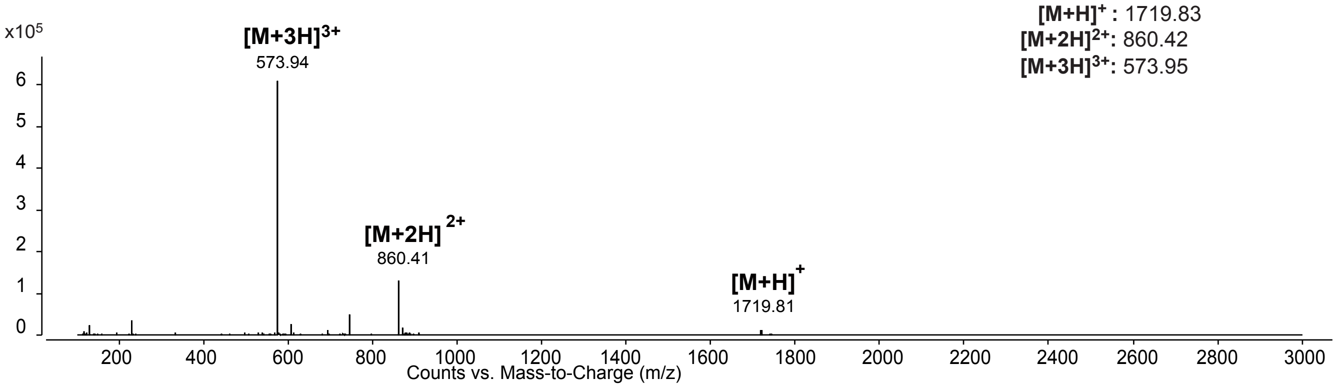

Characterization Data for ASP **29**

RP-HPLC trace of the purified peptide

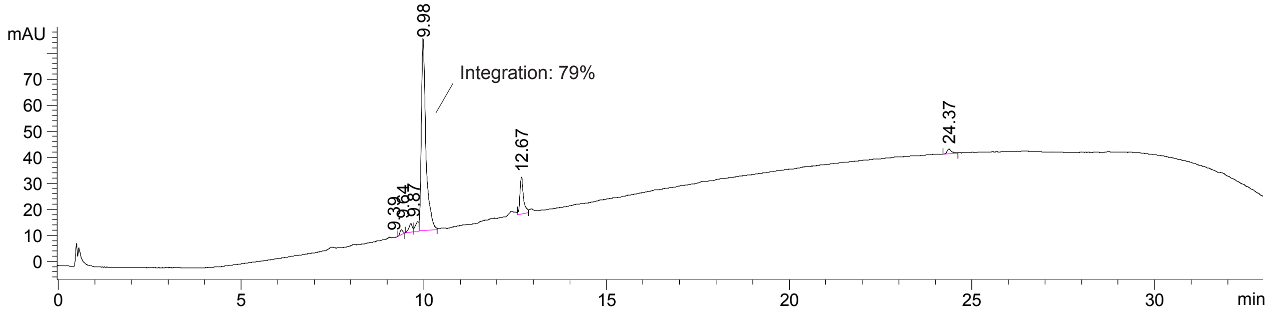

mass spectrum (ESI) of the purified peptide

Calculated *m/z* for $C_{76}H_{128}N_{28}O_{24}S_1$:

$[M+H]^+$: 1849.94
$[2M+3H]^{3+}$: 1233.63
$[M+2H]^{2+}$: 925.48
$[M+3H]^{3+}$: 617.32
$[M+4H]^{4+}$: 370.79

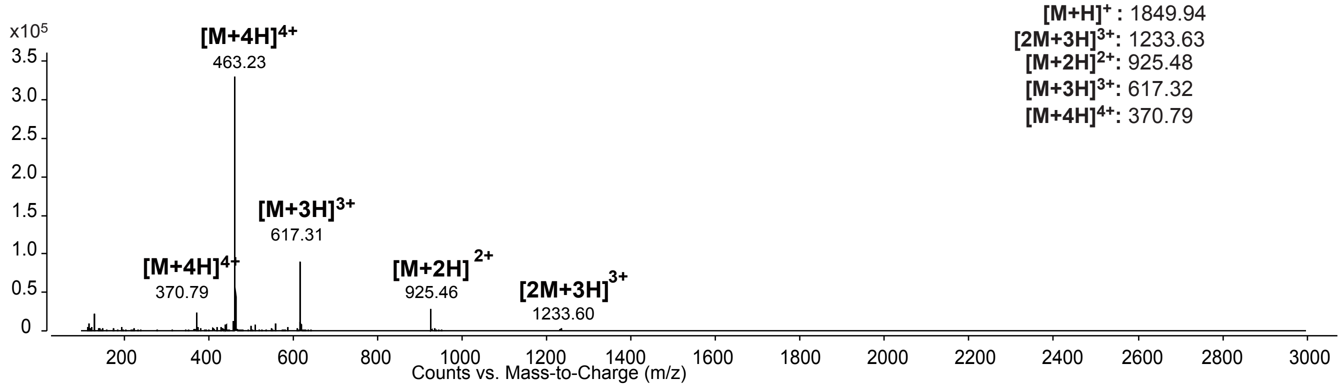

Characterization Data for ASP **30**

RP-HPLC trace of the purified peptide

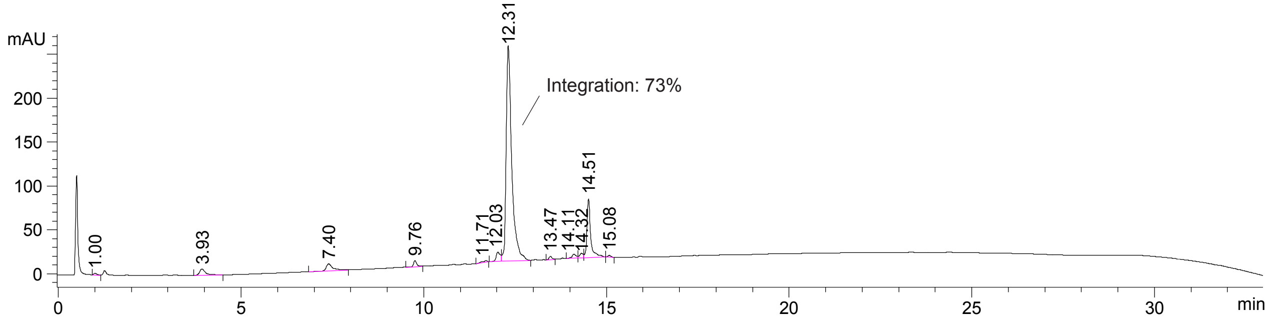

mass spectrum (ESI) of the purified peptide

Calculated *m/z* for $C_{81}H_{127}N_{25}O_{24}S_1$:

$[M+H]^+$: 1866.92
$[M+2H]^{2+}$: 933.97
$[M+3H]^{3+}$: 622.98
$[M+4H]^{4+}$: 467.48

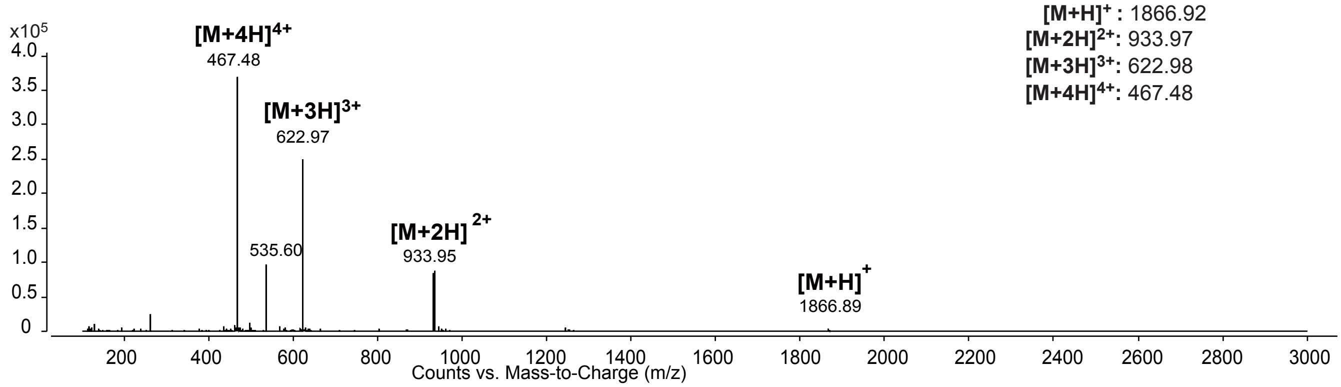

Characterization Data for ASP **31**

RP-HPLC trace of the purified peptide

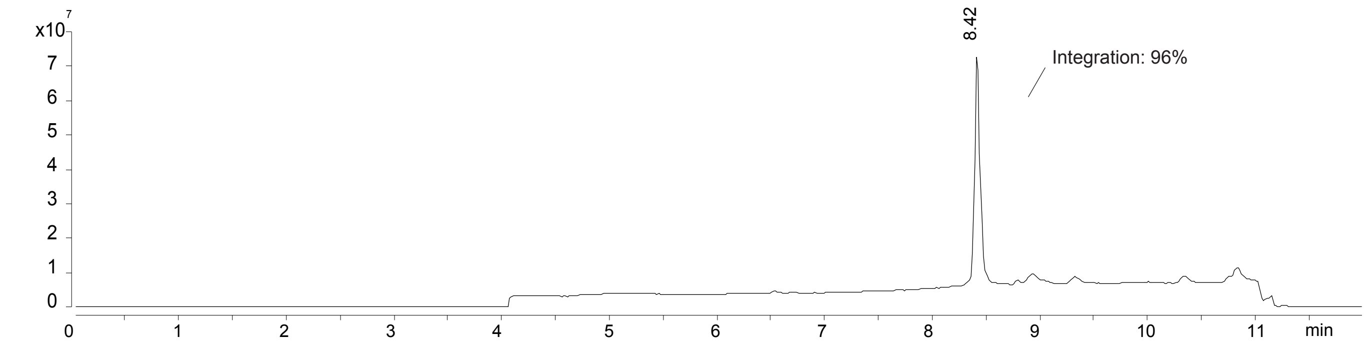

mass spectrum (ESI) of the purified peptide

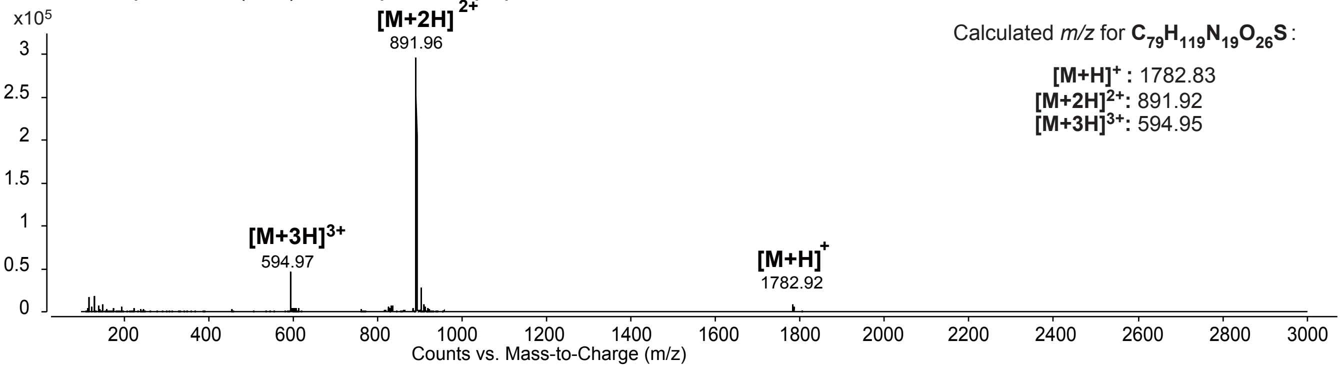

Characterization Data for ASP **32**

RP-HPLC trace of the purified peptide

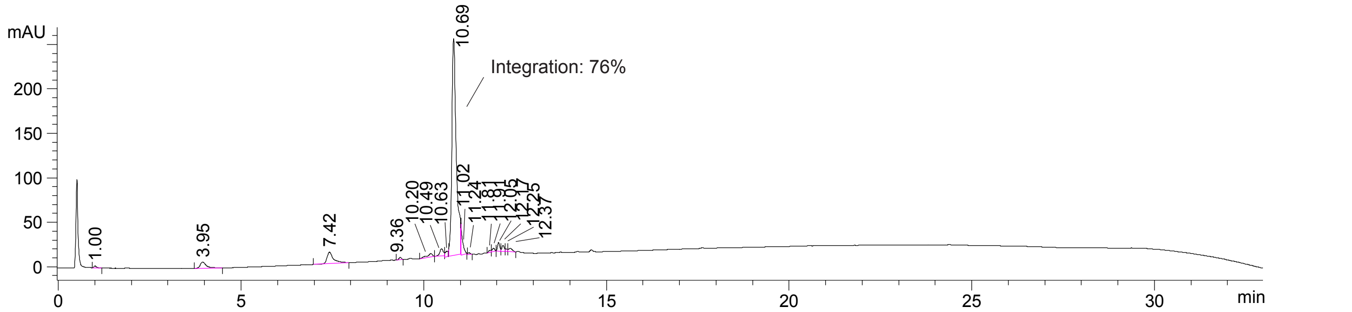

mass spectrum (ESI) of the purified peptide

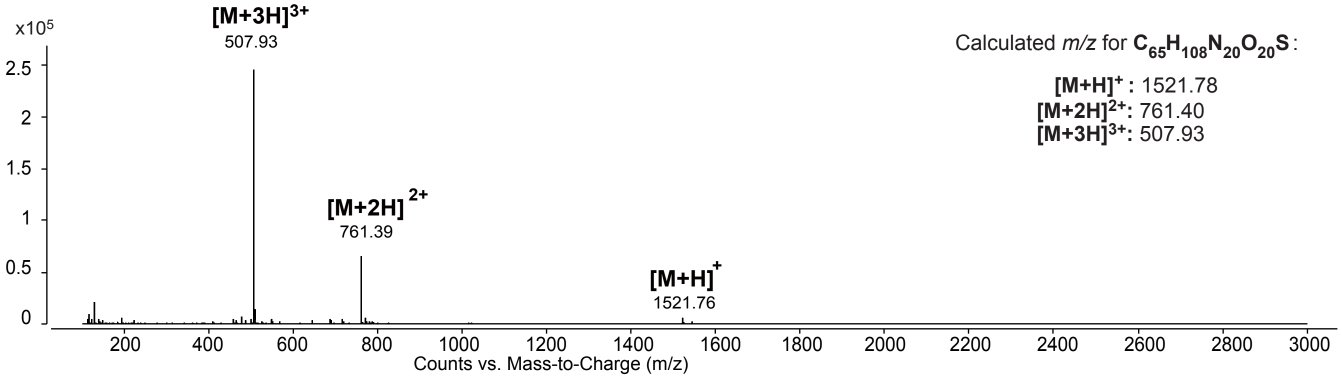

Characterization Data for ASP **33**

RP-HPLC trace of the purified peptide

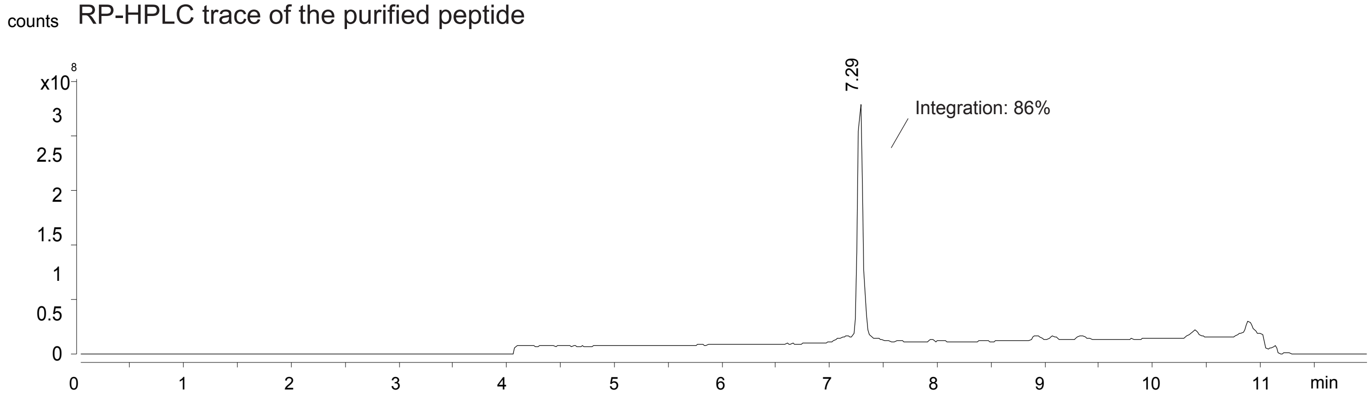

mass spectrum (ESI) of the purified peptide

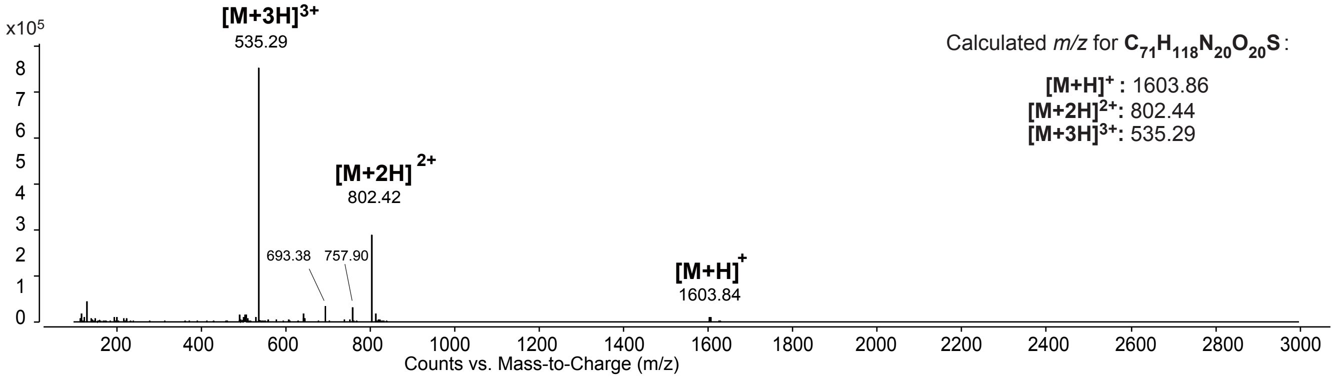

Characterization Data for ASP **34**

RP-HPLC trace of the purified peptide

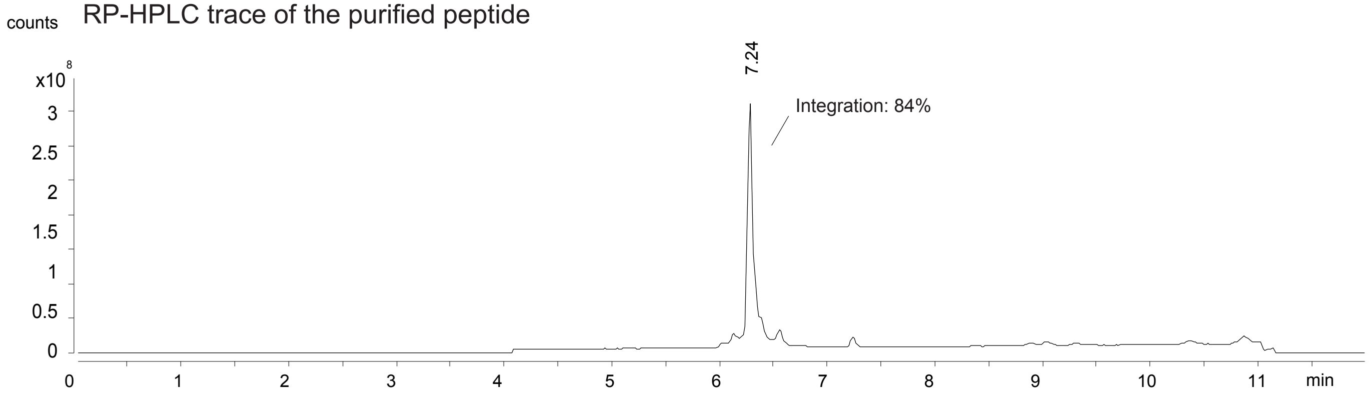

mass spectrum (ESI) of the purified peptide

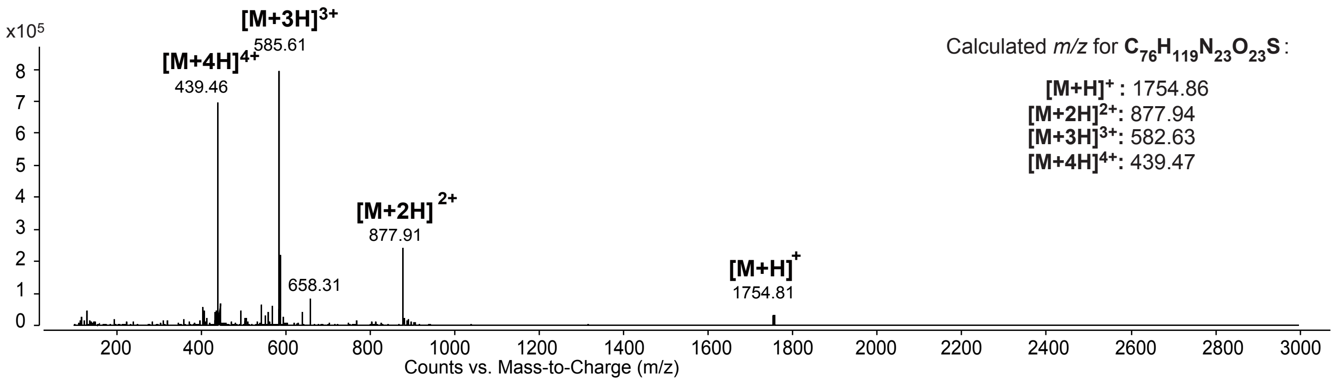

Characterization Data for ASP **35**

RP-HPLC trace of the purified peptide

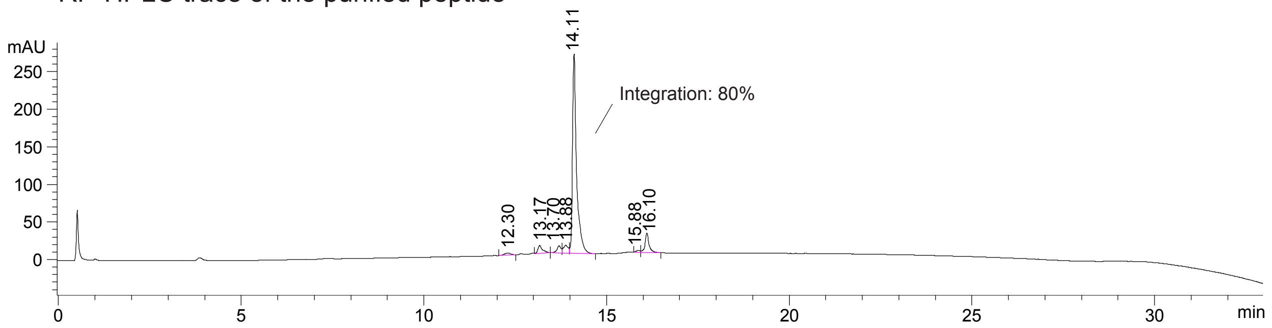

mass spectrum (ESI) of the purified peptide

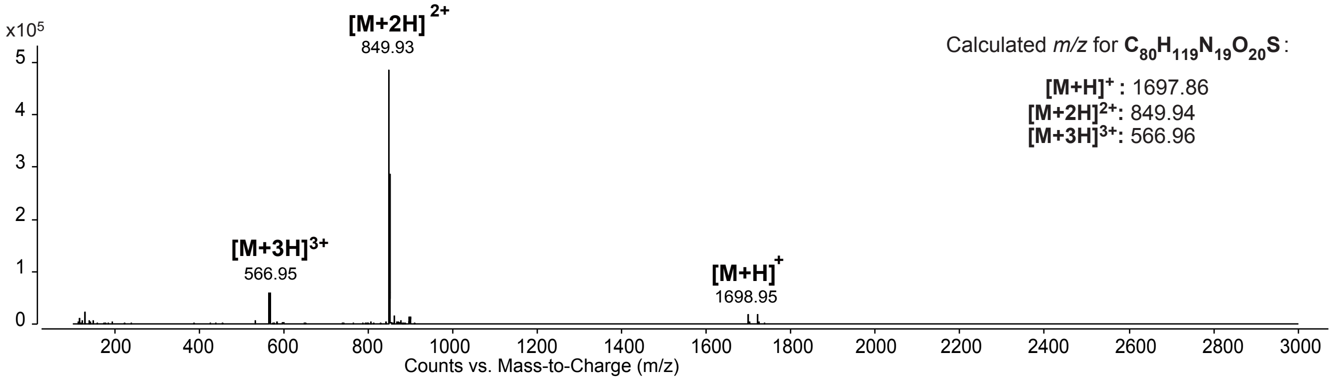

Characterization Data for ASP **36**

RP-HPLC trace of the purified peptide

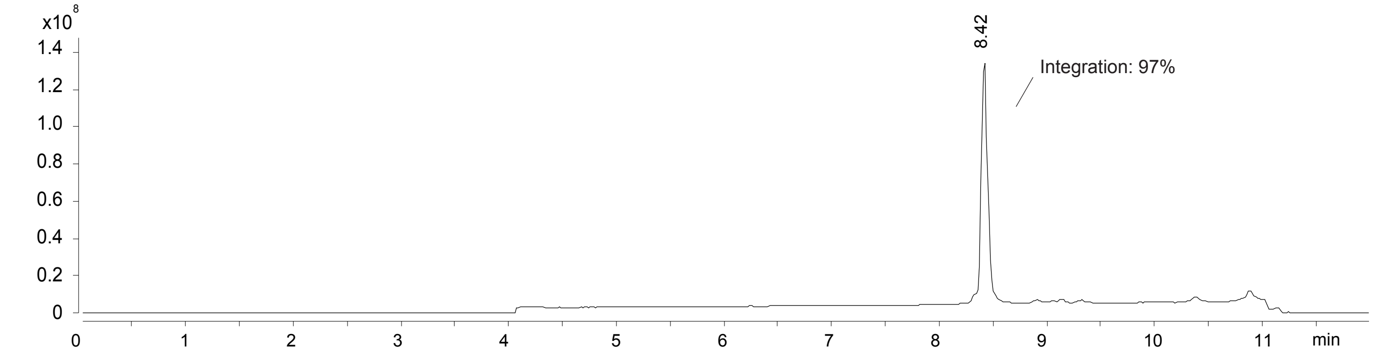

mass spectrum (ESI) of the purified peptide

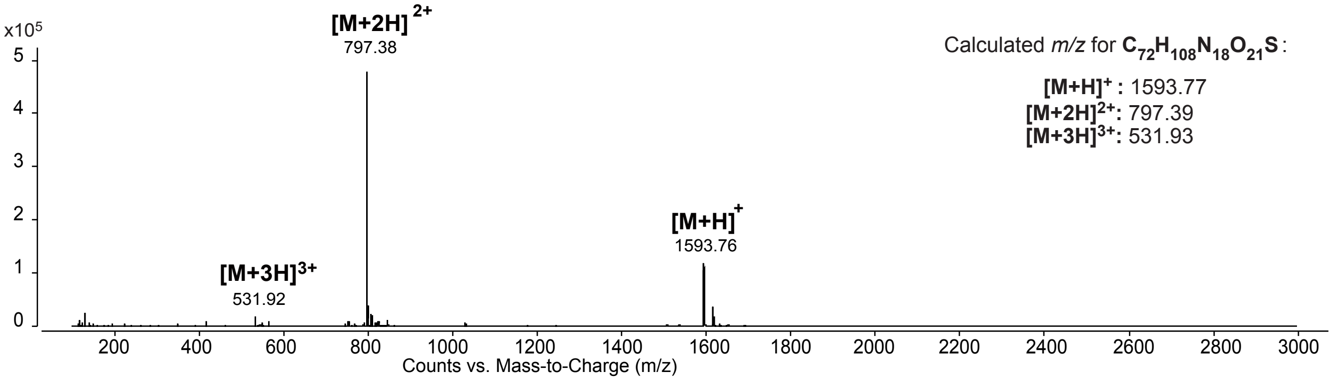

Characterization Data for ASP **37**

RP-HPLC trace of the purified peptide

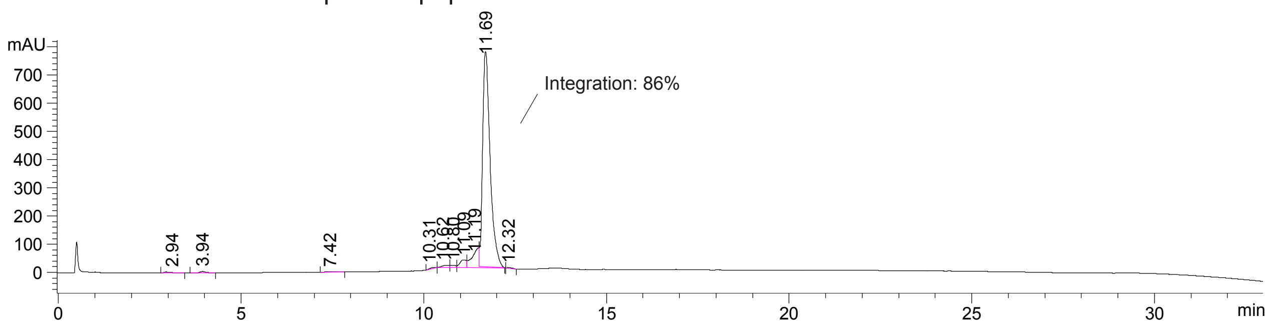

mass spectrum (ESI) of the purified peptide

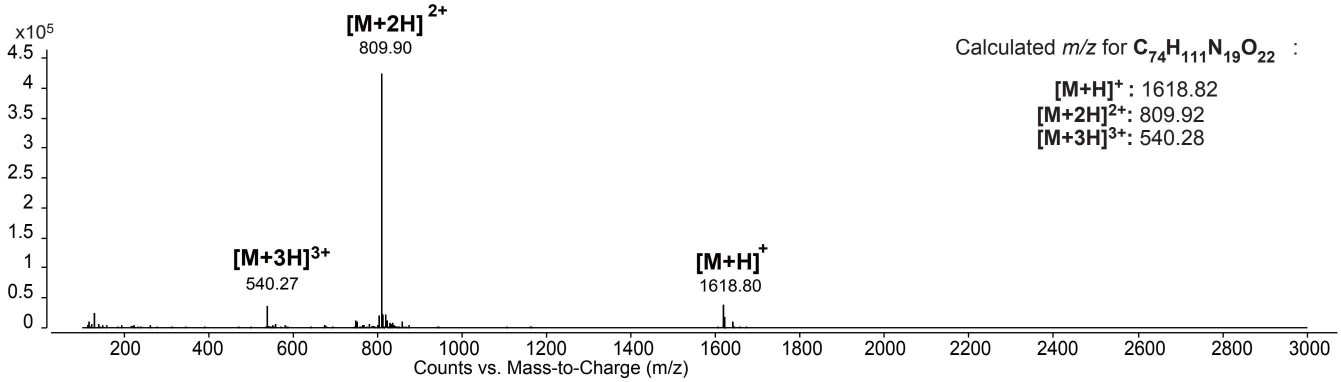

Characterization Data for ASP **38**

RP-HPLC trace of the purified peptide

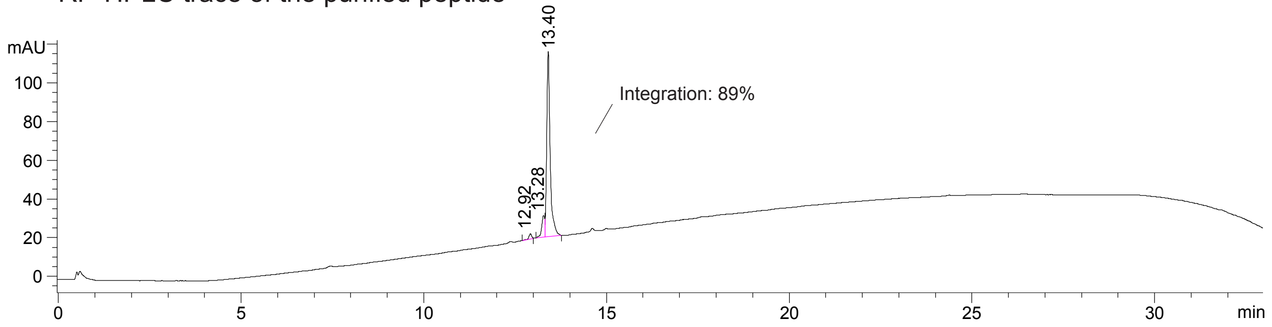

mass spectrum (ESI) of the purified peptide

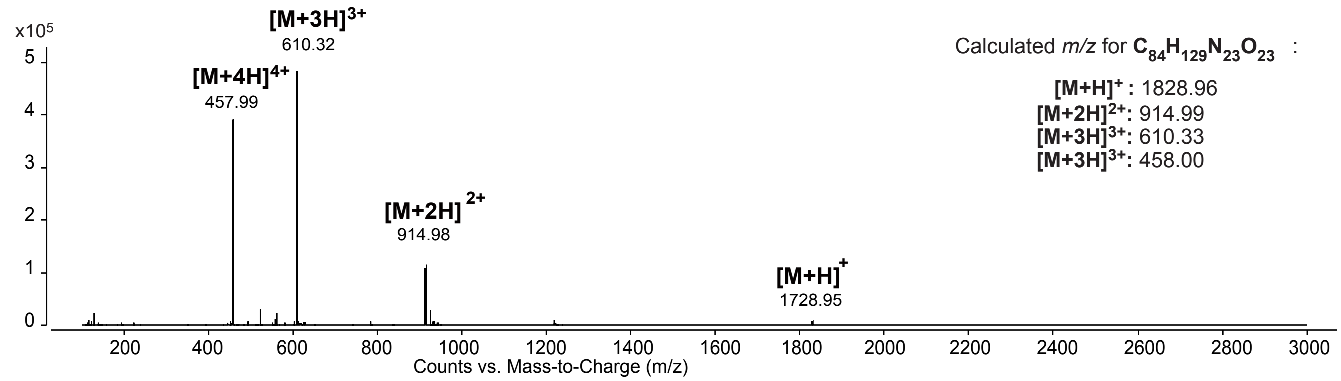

Characterization Data for ASP **39**

RP-HPLC trace of the purified peptide

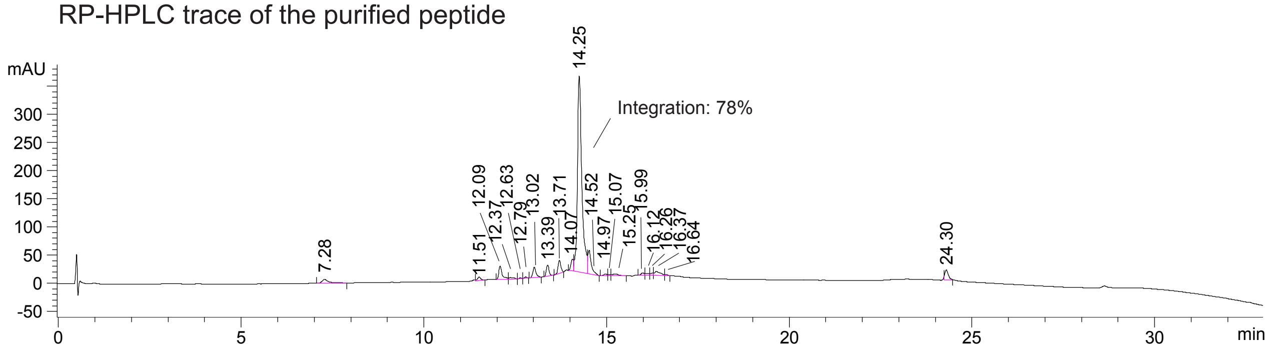

mass spectrum (ESI) of the purified peptide

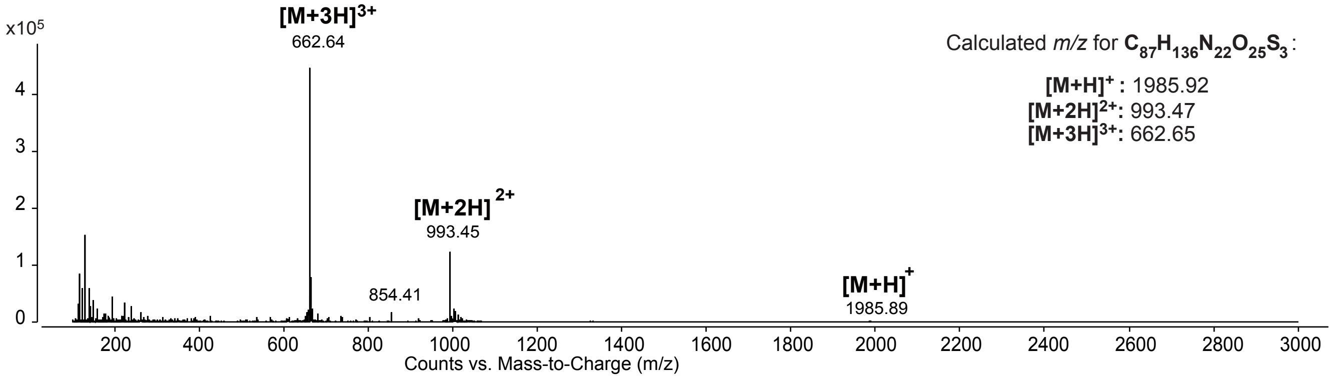

Characterization Data for ASP **40**

RP-HPLC trace of the purified peptide

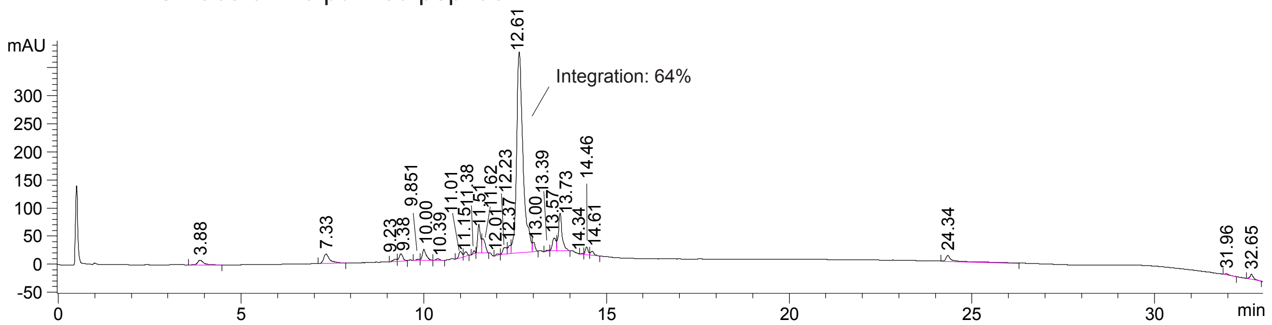

mass spectrum (ESI) of the purified peptide

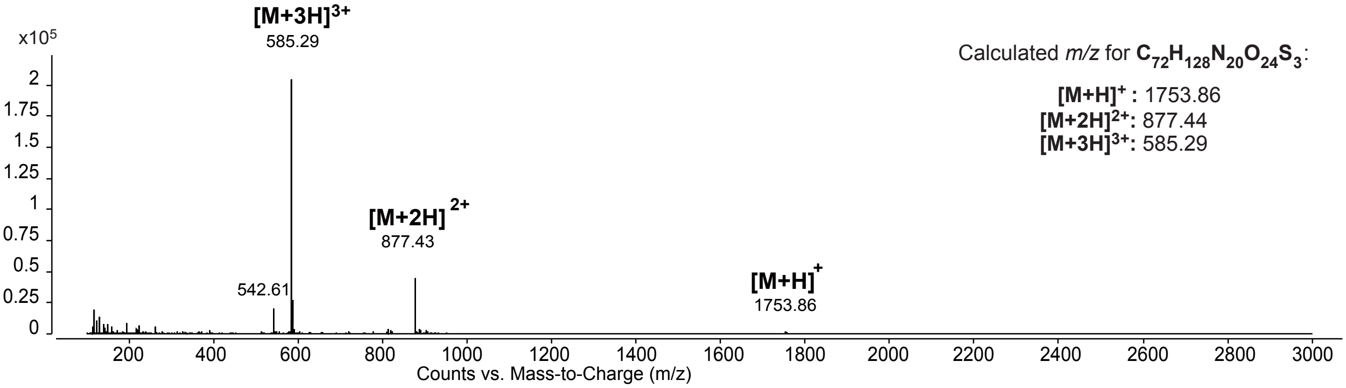

Characterization Data for ASP **41**

RP-HPLC trace of the purified peptide

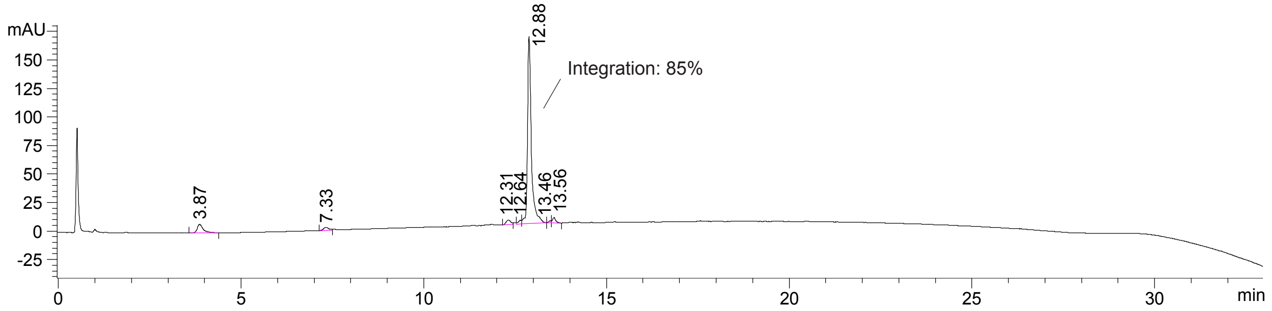

mass spectrum (ESI) of the purified peptide

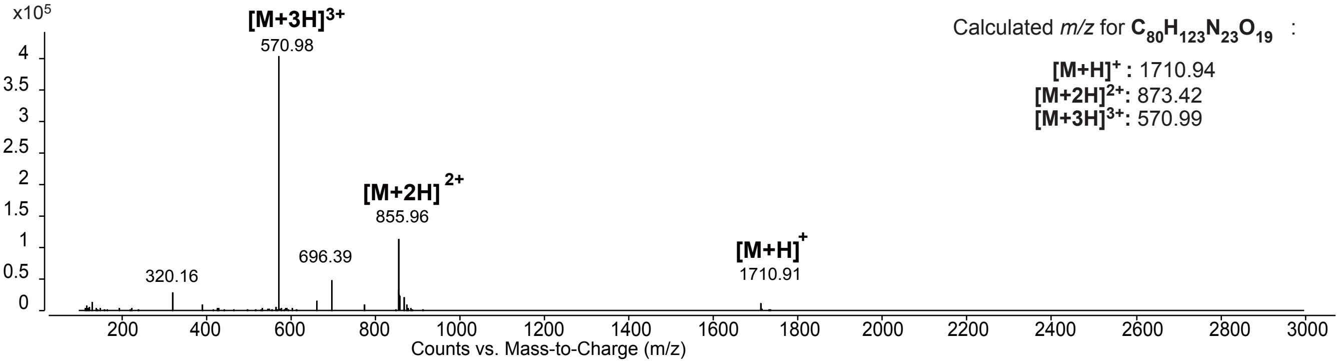

Characterization Data for ASP **42**

RP-HPLC trace of the purified peptide

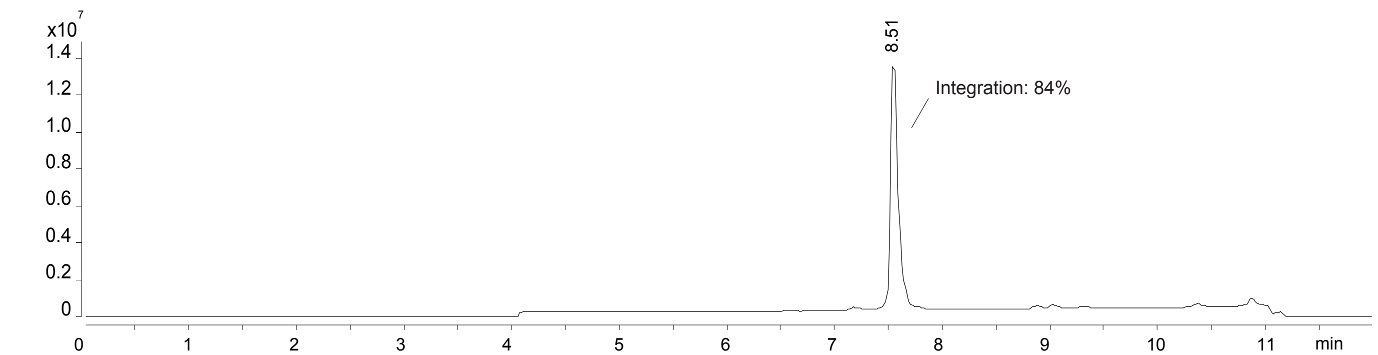

mass spectrum (ESI) of the purified peptide

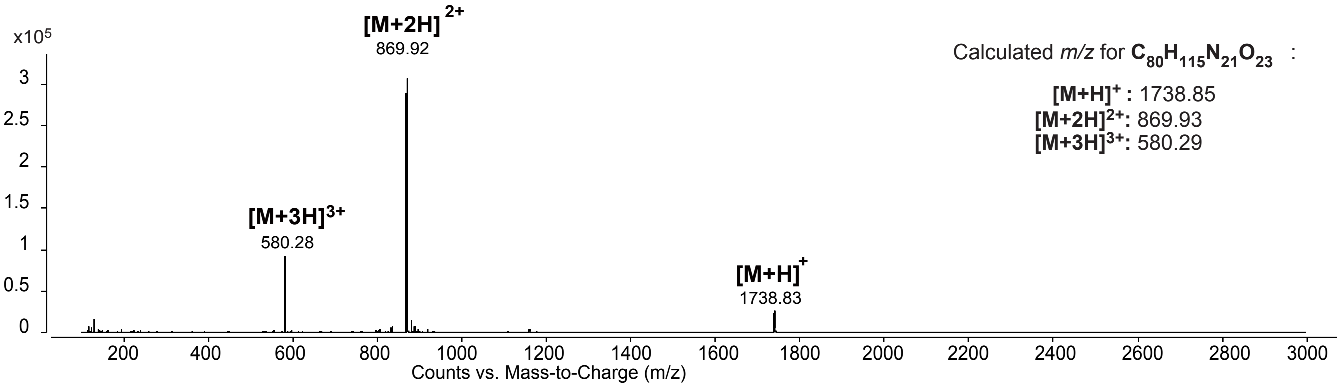

Characterization Data for ASP **43**

RP-HPLC trace of the purified peptide

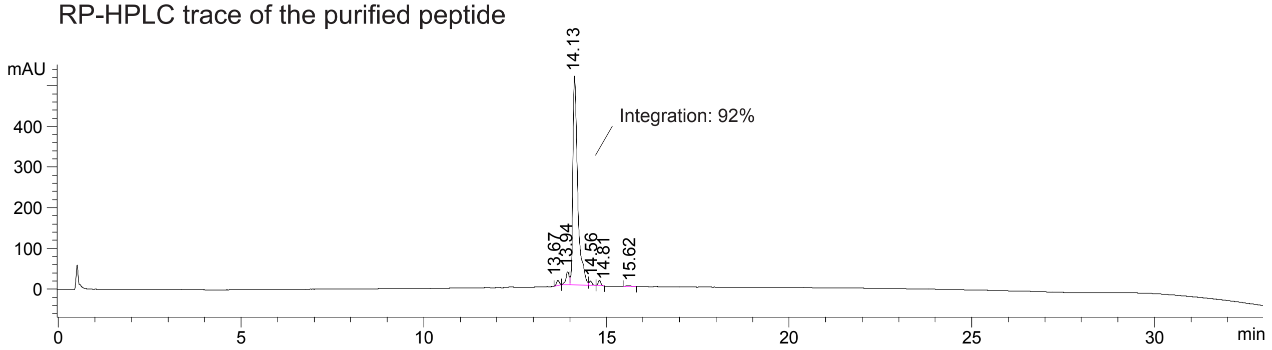

mass spectrum (ESI) of the purified peptide

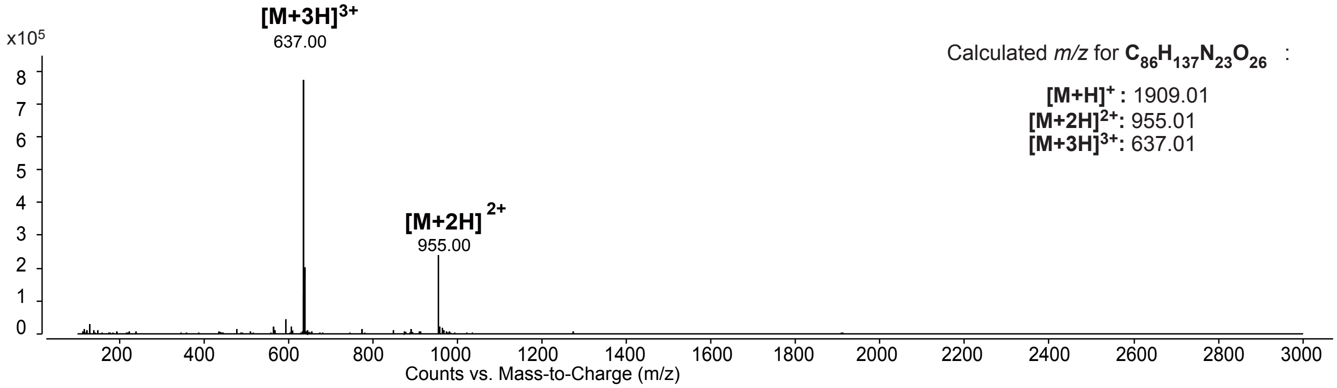

Calculated *m/z* for $C_{86}H_{137}N_{23}O_{26}$:

[M+H]$^+$: 1909.01
[M+2H]$^{2+}$: 955.01
[M+3H]$^{3+}$: 637.01

Characterization Data for ASP **44**

RP-HPLC trace of the purified peptide

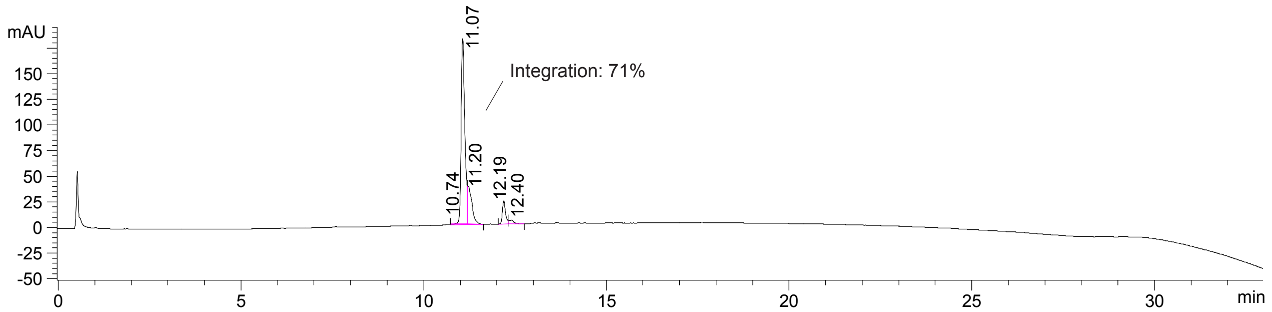

mass spectrum (ESI) of the purified peptide

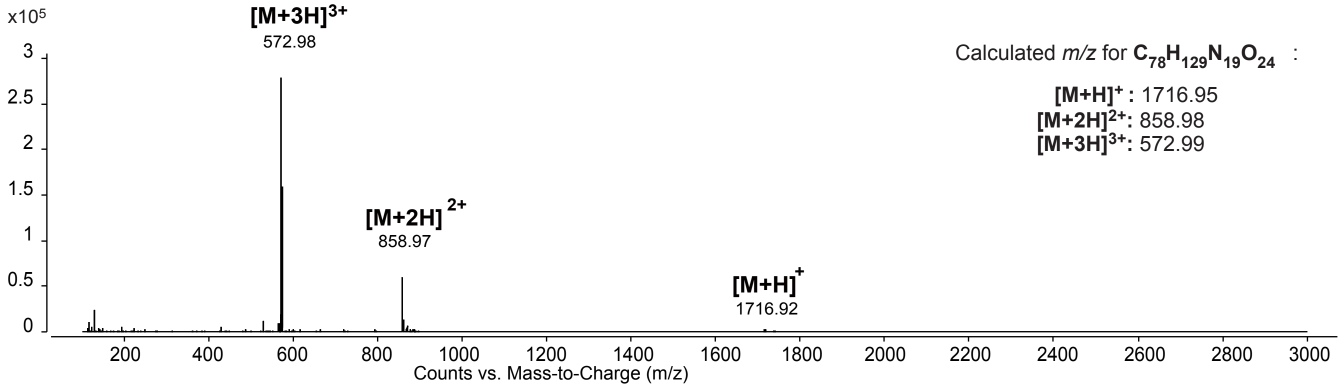

Calculated *m/z* for $C_{78}H_{129}N_{19}O_{24}$:

[M+H]$^+$: 1716.95
[M+2H]$^{2+}$: 858.98
[M+3H]$^{3+}$: 572.99

Characterization Data for ASP **47**

RP-HPLC trace of the purified peptide

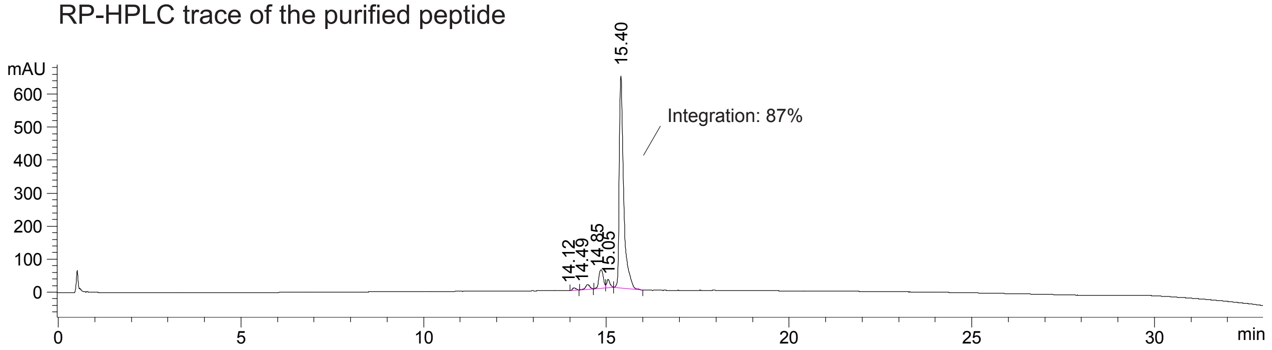

mass spectrum (ESI) of the purified peptide

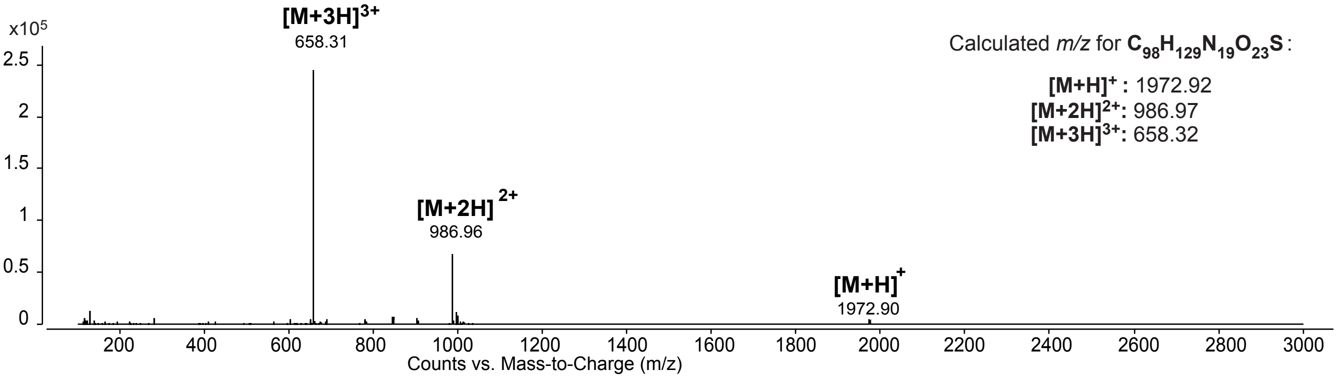

Characterization Data for ASP **48**

RP-HPLC trace of the purified peptide

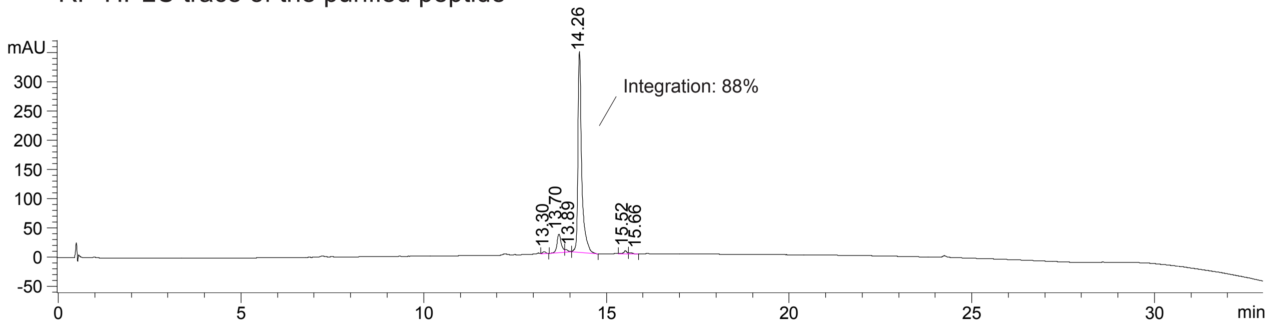

mass spectrum (ESI) of the purified peptide

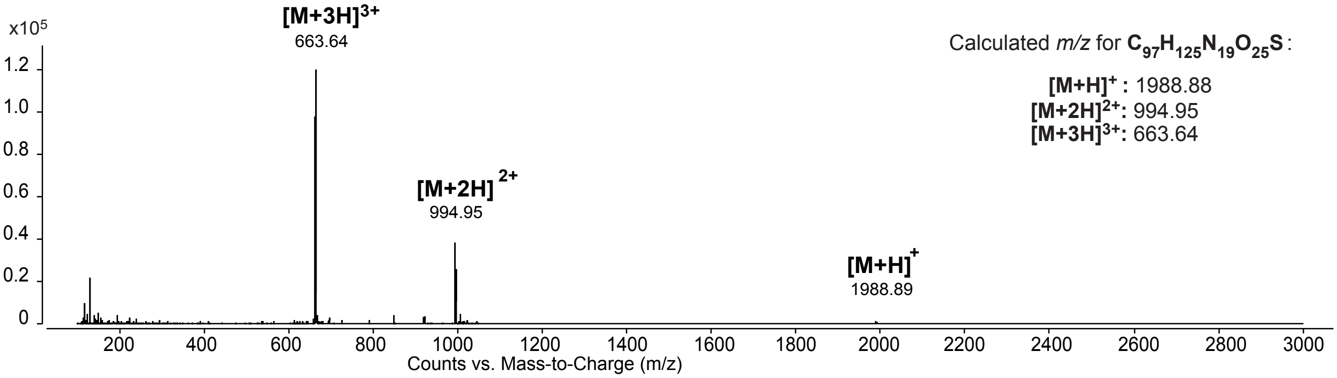

Supplement: Supplementary file 1 — Supplementary information. [file 41598_2019_56943_MOESM1_ESM.pdf]
